# Chemoselective formal β-functionalization of substituted aliphatic amides enabled by a facile stereoselective oxidation event†
†Electronic supplementary information (ESI) available. See DOI: 10.1039/c9sc03715b


**DOI:** 10.1039/c9sc03715b

**Published:** 2019-09-10

**Authors:** Adriano Bauer, Nuno Maulide

**Affiliations:** a Institute of Organic Chemistry , University of Vienna , Währinger Straße 38 , 1090 Vienna , Austria . Email: nuno.maulide@univie.ac.at

## Abstract

Aliphatic C–H functionalization is a topic of current intense interest in organic synthesis. Herein, we report that a facile and stereoselective dehydrogenation event enables the functionalization of aliphatic amides at different positions in a one-pot fashion. Derivatives of relevant pharmaceuticals were formally functionalized in the β-position in late-stage manner. A single-step synthesis of incrustoporine from a simple precursor further showcases the potential utility of this approach.

## Abstract 2

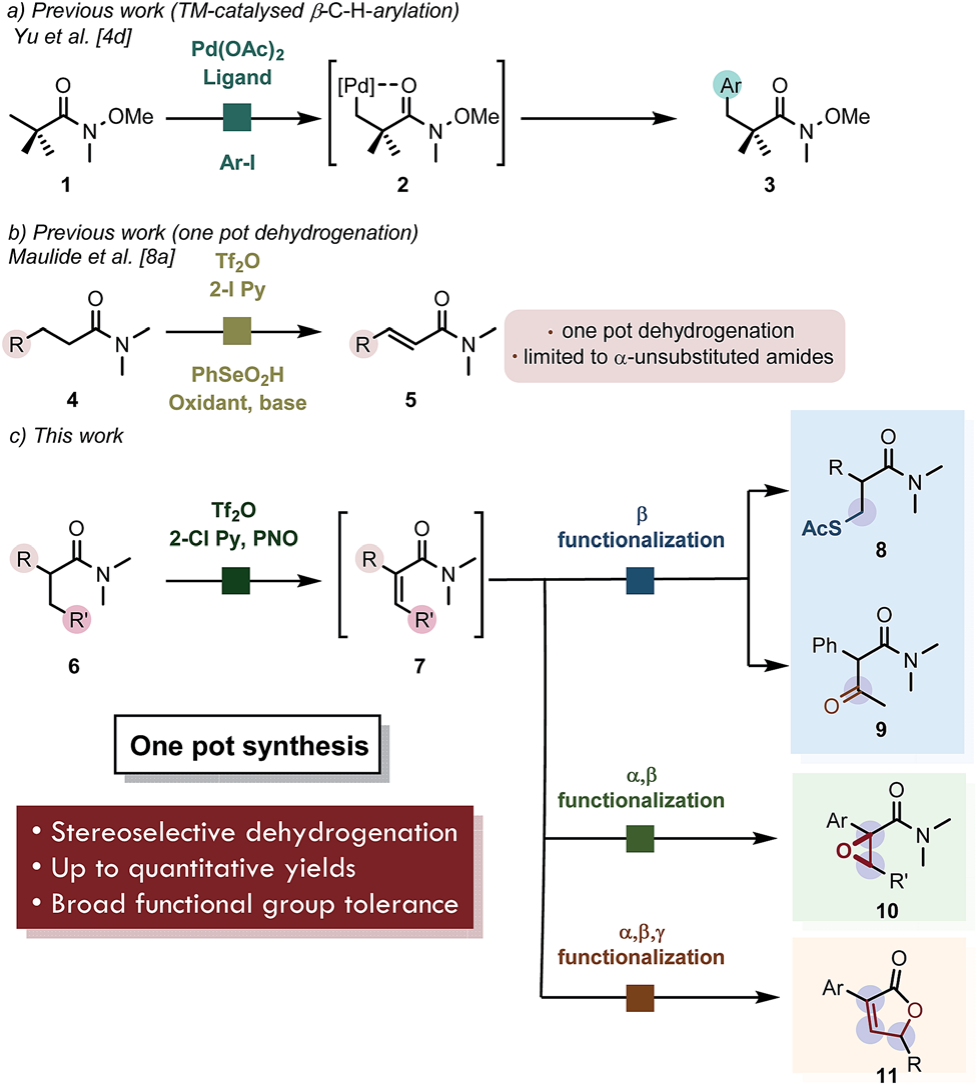
A facile dehydrogenation event enables the functionalization of aliphatic amides at different positions in a one-pot fashion.

## Introduction

**Scheme 1:**
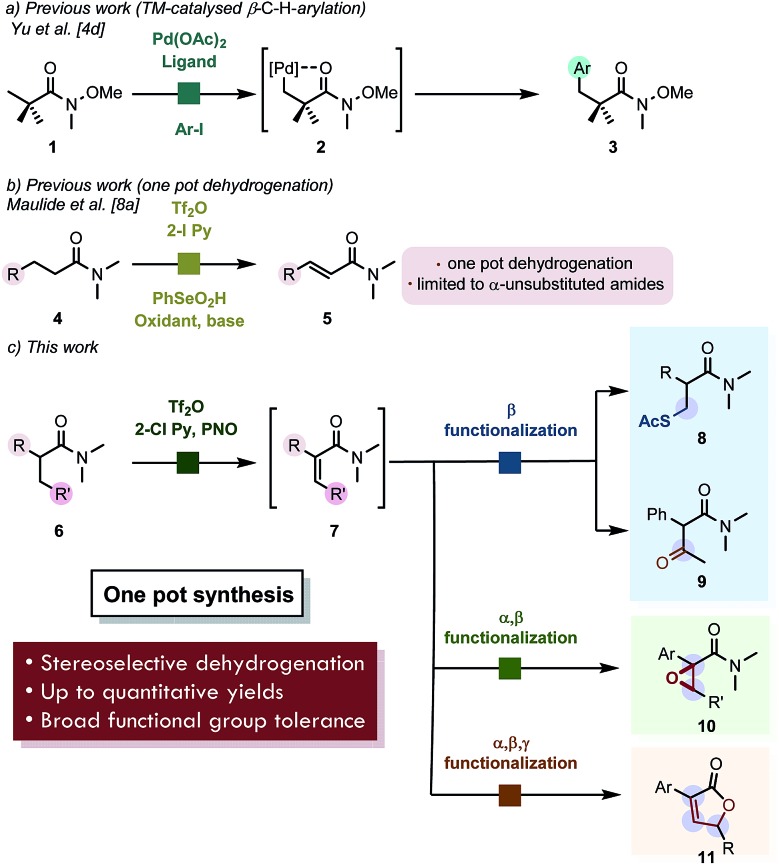
(a) Previous work on β-C–H functionalization of aliphatic, quaternary carboxamides. (b) Earlier report on dehydrogenation of carboxamides by electrophilic activation. (c) Chemoselective, formal remote functionalization of carboxamides enabled by stereoselective dehydrogenation.

The carbonyl functional group remains one of the most important synthetic handles in organic chemistry.^1^,^2^ While the α-carbon atom and the *ipso*-position of this ubiquitous structural motif can be modified in many ways,^2^ the direct modification of the β-position is more elusive. With the advent of C–H functionalization, transformation of molecular moieties which are traditionally perceived as unreactive became feasible.^1^–^3^ The past decades have witnessed an explosive wealth of accomplishments regarding this family of transformations.^3^ Relying on the transient generation of internally chelated, cyclopalladated intermediates such as **2** (Scheme 1a), this mode of C–H functionalization has been effectively exploited particularly for β-methyl carbonyl derivatives.^4^ The emerging field of photoredox catalysis has also proven to be a powerful tool for the β-functionalization of cyclic ketones.^5^

More conventional approaches make use of classical Michael addition strategies. However, the requisite α,β-unsaturated carbonyl compound is not always readily available. While the oxidative dehydrogenation of α-branched chloroenamines has been previously realized by Ghosez *et al.*8*b* (though no *E*/*Z* selectivity was described), we have recently disclosed a selenium-mediated, one-pot α,β-dehydrogenation of amides (*cf.* – **4** → **5**, Scheme 1b) in moderate to good yields. More recently a stepwise dehydrogenation using α-TEMPO (tetramethylpiperidinyloxyl) amides as intermediates for the dehydrogenation of linear amides has been reported.8*c*

This approach is predicated on the electrophilic activation of carboxamides, a reactivity mode that enables transformations ranging from the venerable [2 + 2] cycloaddition6a–d and *ipso* substitution6e–g reactions all the way to the functionalization of the α-position *via* rearrangement and Umpolung chemistry.^7^ However, the method depicted in Scheme 1b is limited to α-unsubstituted amides.8*a* In this manuscript, we report a stereoselective and mild α,β-dehydrogenation and how it enables not only the direct formal β-C(sp^3^)–H functionalization of simple amides under a metal-free regime, but also the functionalization of multiple carbon atoms in α,β and γ-positions.

## Results and discussion

### Preliminary results

**Scheme 2:**
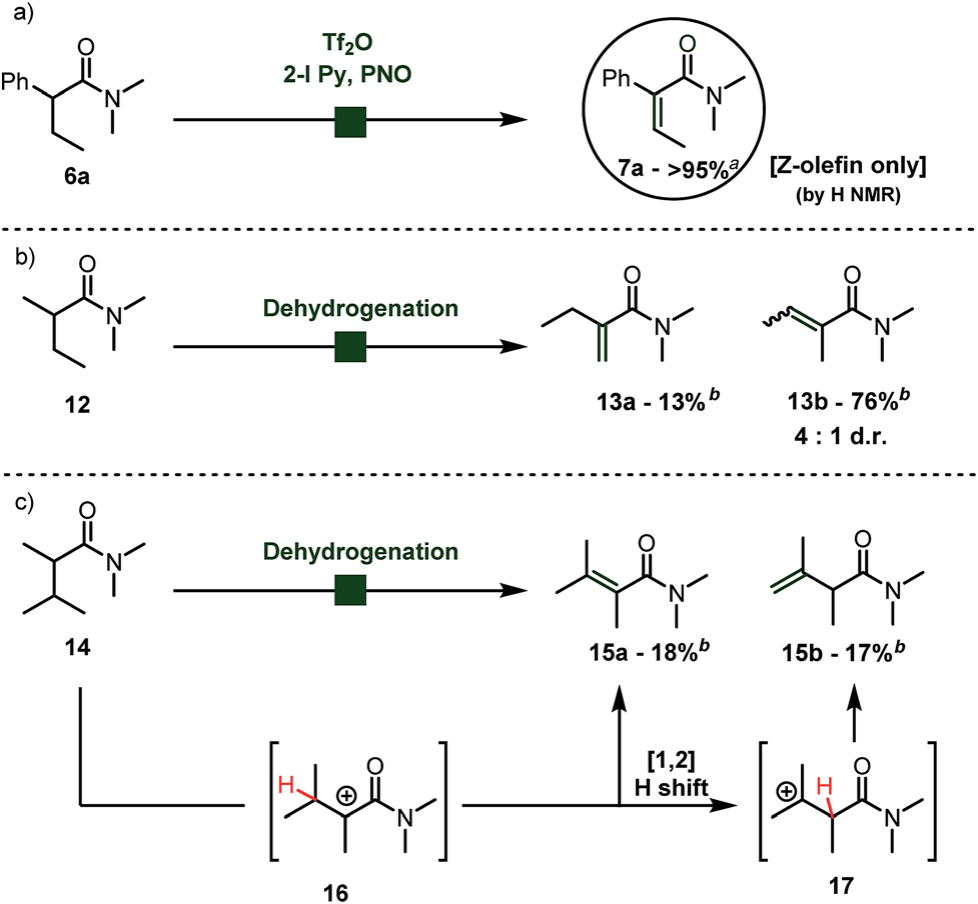
(a) Preliminary results. (b) Dehydrogenation of purely aliphatic α-branched amides. (c) Unexpected formation of a β,γ-dehydrogenated amide. ^*a*^ Yield refers to the pure isolated product. ^*b* 1^H NMR yield with mesitylene as the internal standard.

During our studies on the Umpolung functionalization of amides,^7^ we observed that α-branched amides deviate from Umpolung reactivity at the α-carbon. Instead, treatment of α-branched amides with trifluoromethanesulfonic anhydride (Tf_2_O), 2-iodo pyridine and pyridine *N*-oxide (PNO) resulted in clean and high-yielding α,β-dehydrogenation rather than the Umpolung reactivity we had previously observed.^9^ Moreover, α-aryl-substituted amides gave quantitative yield with the exclusive formation of one alkene-isomer. Importantly, careful analysis revealed that the dehydrogenation of α-aryl substituted amides gave exclusively the *Z*-olefin (by NMR), whose structure was confirmed by NOESY-NMR spectroscopy (*cf.*Scheme 2a, **6a** → **7a**). On the other hand, α-branched dialkyl amides typified by **12** afforded an inseparable mixture of regio- and stereoisomers (Scheme 2b) with moderate selectivity. In particular, when the isopropyl-substituted, α-branched amide **14** was submitted to the reaction conditions we observed (Scheme 2c) equal amounts of the expected tetrasubstituted alkene **15a** and the unexpected β,γ-dehydrogenated amide **15b** by NMR. The latter product is presumably formed by a Wagner–Meerwein rearrangement of the putative unstable carbocation **16** as shown (Scheme 2c). It is noteworthy that **14** reacted sluggishly.

The dehydrogenation reaction leads to very similar results regardless of whether 2-chloro- or 2-bromo pyridine is used instead of 2-iodo pyridine (a relevant piece of information for future developments, *vide infra*. See the ESI for further details†).

**Scheme 3:**
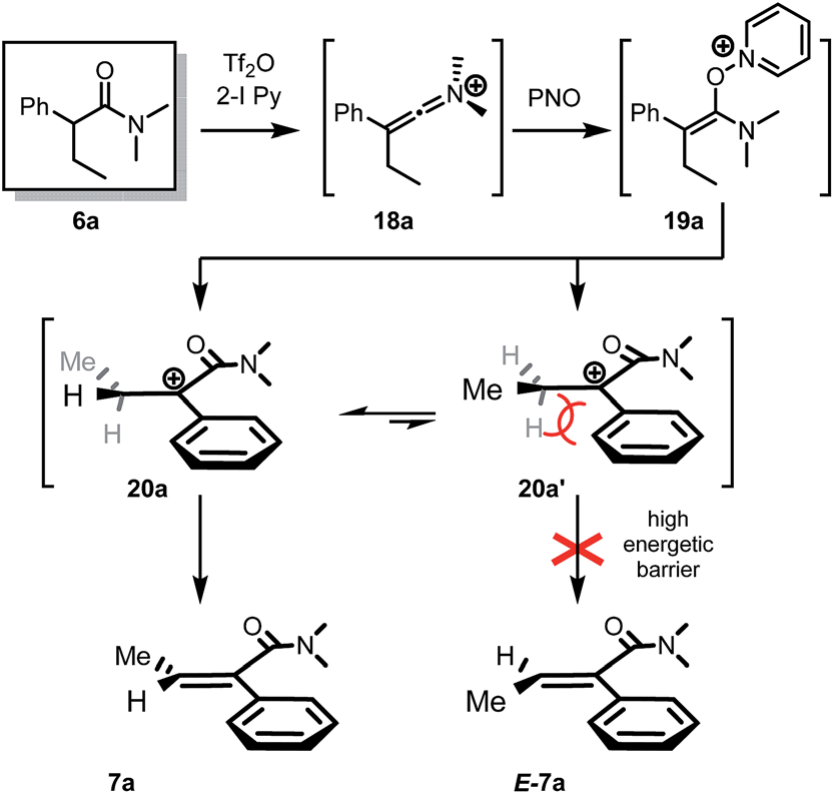
Proposed mechanism and stereochemical model for the observed high stereoselectivity of the dehydrogenation event.^14^

No reaction intermediates *en route* to dehydrogenation could be detected by NMR spectroscopy. We believe that the reaction proceeds by swift fragmentation of the enolonium species **19a** to an α-carbocationic amide **20** (Scheme 3).^14^ A rationale for the high *Z*/*E* selectivity is provided by the model shown in Scheme 3. Efficient mesomeric stabilization of the putative carbocationic intermediate mandates perpendicular arrangement of the adjacent aromatic moiety, resulting in restricted rotation of the arene substituent.^14^ This exacerbates steric hindrance considerations, disfavoring a scenario where even a methyl group in the β-position is on the same side as the arene ring. As steric congestion increases during formation of the double bond, it is likely that elimination is greatly favoured from the thermodynamically favored conformer **20a**.

### One pot α,β-epoxidation

**Scheme 4:**
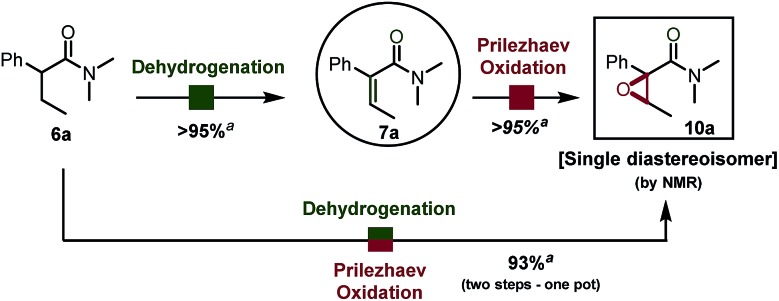
α,β-Epoxidation of a simple amide. See ESI† for detailed reaction conditions. ^*a*^ Yield refers to the pure isolated product.

This surprisingly stereoselective and efficient result (quantitative conversion within minutes at room temperature) encouraged us to leverage *in situ* oxidation in order to achieve formal remote functionalization reactions. In particular, we were looking to draw on the high diastereoselectivity of the dehydrogenation event. In our hands, Prilezhaev oxidation using *meta*-chloroperbenzoic acid (commercial grade *m*CPBA) proceeded smoothly in quantitative yield at room temperature (Scheme 4).^10^

When we investigated the possibility of a one pot procedure an unexpected hurdle was encountered, in that the original procedure using 2-iodo pyridine for the dehydrogenation step was not compatible with the Prilezhaev oxidation, most likely due to the easily oxidized iodine atom. This issue was readily circumvented by using 2-chloro pyridine instead. Pivotal to the one-pot procedure with 2-chloro pyridine was the addition of an acid quencher (water or an aqueous NaHCO_3_ solution) before adding *m*CPBA, owing to the high acidity of protonated 2-chloropyridine present in the solution (p*K*_a_ = 0.49 in H_2_O).^12^

**Scheme 5:**
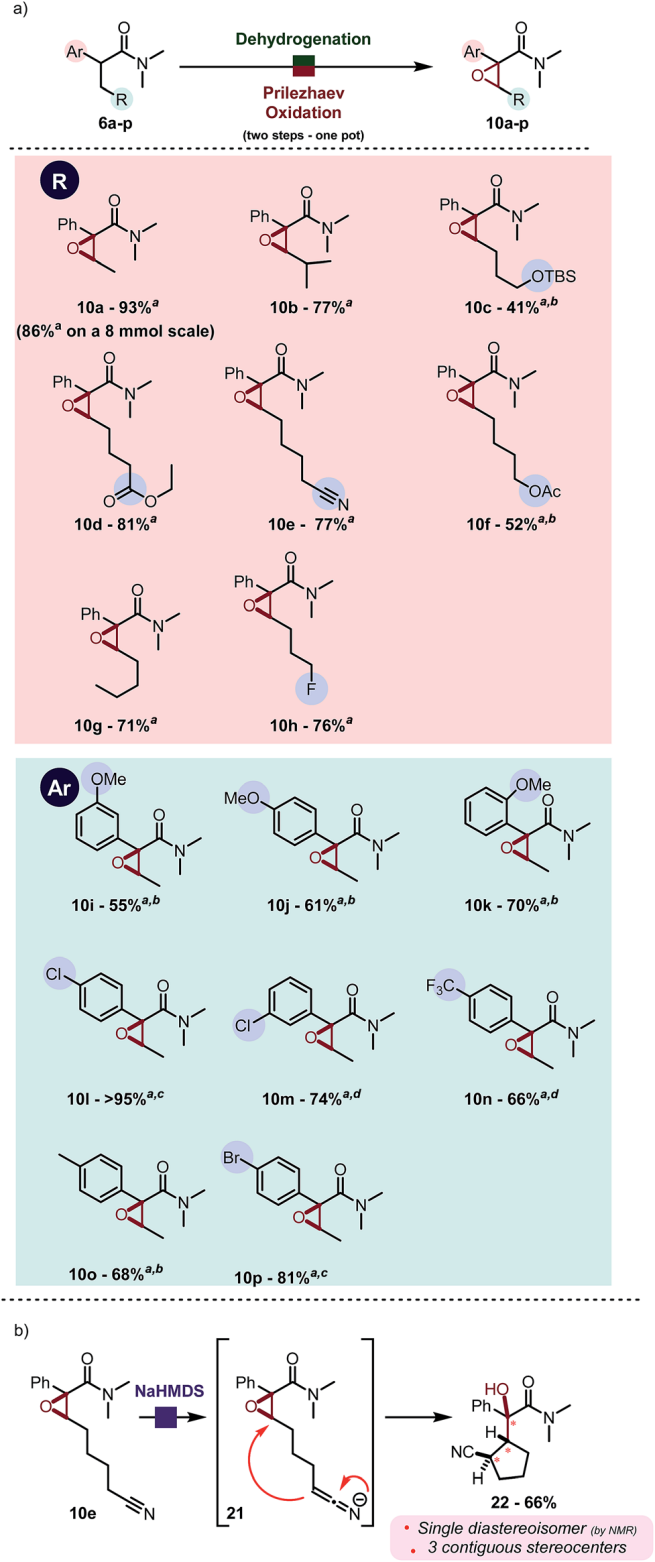
(a) Product scope of the one pot desaturation–epoxidation reaction. Reactions were carried out on a 0.2 mmol scale in DCM (0.2 M) with Tf_2_O (1.1 eq.), 2-Cl Py (2.2 eq.), PNO (1.3 eq.) at 0 °C then r.t., H_2_O and *m*CPBA (3.0 eq.) at r.t. ^*a*^ Yield refers to the pure isolated product. ^*b*^ Saturated aqueous NaHCO_3_ used instead of water. ^*c*^ Reaction was heated to 40 °C during epoxidation ^*d*^ Reaction was carried out in 1,2-DCE instead of DCM and heated to 60 °C during epoxidation. (b) Application of one of the products.

By use of this procedure, formal α,β-oxygenation of simple amide **6a** was achieved in 93% isolated yield in one pot. The reaction proceeds with a very similar yield on a gram scale. As shown in Scheme 5a, this transformation has some generality and tolerates a range of functional groups, such as esters (**10d**, **10f**), nitriles (**10e**) and silyl ethers (**10c**). Electron-withdrawing (such as pCF_3_, **10n**) and -donating groups (such as OMe, *cf.***10j**) are well tolerated at diverse positions of the aryl substituent (Scheme 5a). Heating to 40 °C was found to be generally beneficial during the epoxidation event to enhance conversion in cases were the aromatic ring is electronically impoverished.^11^ The use of aqueous NaHCO_3_ instead of water enables successful reaction for acid-sensitive substrates (*e.g.* TBS protected alcohol, *cf.***10c**) or sensitive products (electron rich aromatics in proximity to the formed epoxide, *cf.***10j**). Diastereoselectivity was found to be excellent in all cases: the only case in which the other diastereoisomer was detected in the crude reaction mixture (in traces by ^1^H NMR, d.r. > 25 : 1) is the sterically congested product **10b**. These epoxides can serve as precursors to more elaborated architectures as depicted in Scheme 5b. NaHMDS-mediated deprotonation of **10e**, which was obtained in good yield using the present protocol, led to diastereoselective intramolecular epoxide opening.^13^ The resulting tertiary alcohol **22**, which contains three contiguous stereogenic centers, was obtained as a single diastereoisomer.

### One pot β-oxidation and oxidative C–C bond cleavage

**Scheme 6:**
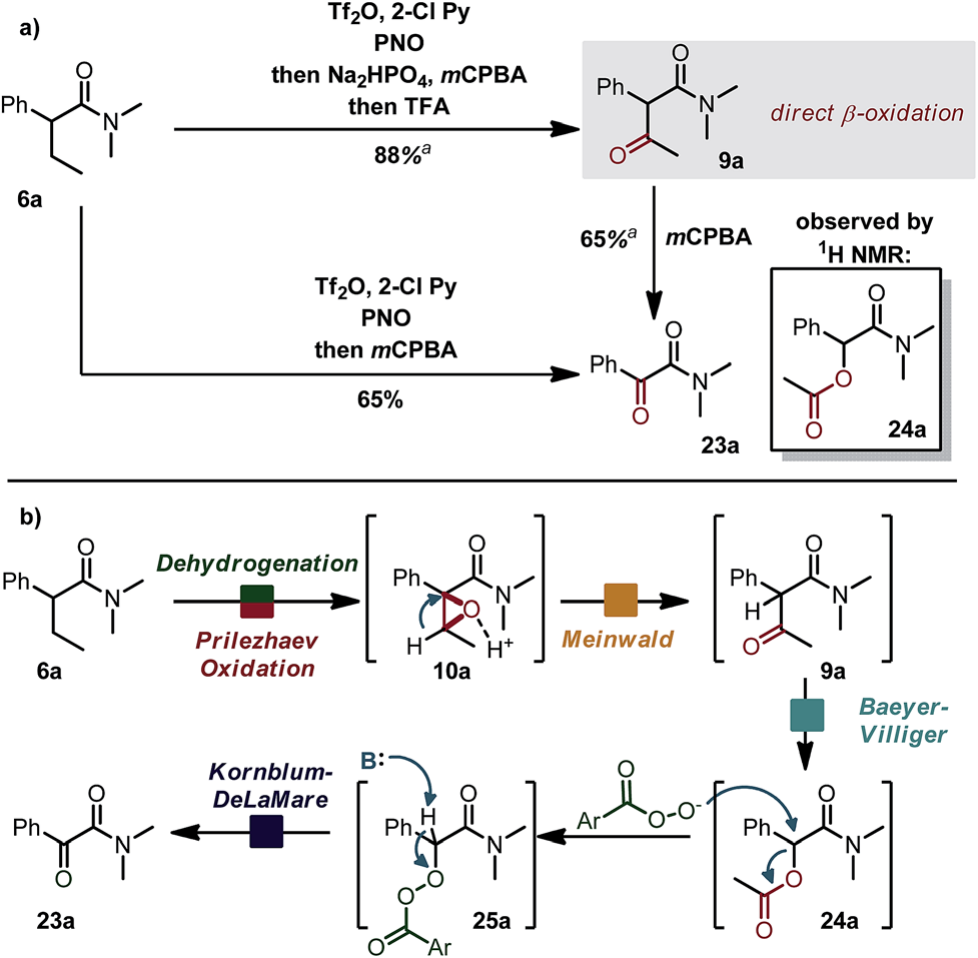
(a) Alternative pathway towards different ketoamides. See ESI† for detailed reaction conditions. (b) Proposed mechanism.

Interestingly (Scheme 6a), when the acid scavenger was omitted in the domino α,β-oxidation of amide **6a**, α-ketoamide **23a** was obtained as the major product, accompanied by trace amounts of α-acetoxyamide **24a** and β-ketoamide **9a**. Selectivity can be steered exclusively towards β-ketoamide **9a** by adding first a non-aqueous proton-scavenger (Na_2_HPO_4_) and then acid (trifluoroacetic acid, TFA).^15^ When the purified β-ketoamide **9a** (isolated in 88% yield) was treated with *m*CPBA in DCM, we observed α-ketoamide **23a** as the main product with the α-acetoxy derivative **24a** as a side product. We thus propose a mechanistic scenario (Scheme 6b) whereby acid-induced Meinwald rearrangement^15^ of epoxide **10a** delivers ketone **9a**. Baeyer–Villiger oxidation of the latter with *m*CPBA (preferential migration of the benzylic substituent)^16^ combined with nucleophilic attack of *m*CPBA anion/Kornblum–DeLaMare rearrangement^17^ accounts for the formation of products **23a** and **24a**. In particular, the β-oxidation of an amide to a β-ketoamide (**6a** → **9a**) is, to the best of our knowledge, an unknown transformation with potential synthetic utility.

### Synthesis of 2-furanones

**Scheme 7:**
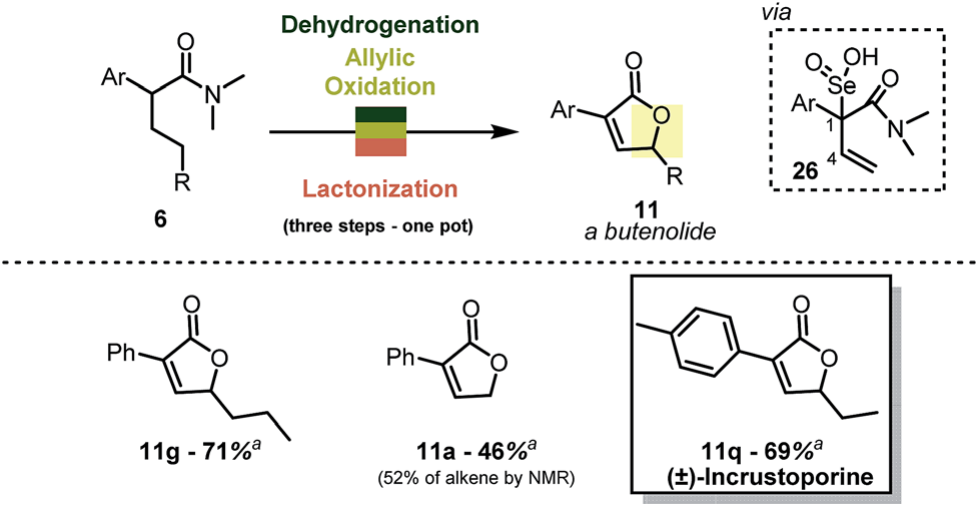
One-pot synthesis of butenolides. Reactions were carried out on a 0.2 mmol scale in 1,2-DCE (0.1 M) with Tf_2_O (1.1 eq.), 2-I Py (2.2 eq.), PNO (1.3 eq.) at 0 °C then r.t., 1,4-dioxane and SeO_2_ (2.0 eq.) at 80 °C. ^*a*^ Yield refers to the pure, isolated product.

When combined with allylic oxidation,^18^ the mild dehydrogenation chemistry described herein results in a one-step synthesis of butenolides from simple carboxamides (Scheme 7). The oxidation is most likely followed by nucleophilic displacement of the R–O–Se–OH functionality *via* the amide carbonyl.^19^ The stereoretention of the double bond geometry during oxidation is noteworthy, and the absence of products with inversion of the double bond configuration can be rationalised by the transition state of the [2,3] rearrangement of intermediate **26** (ref. 20). The reaction enables, for instance, the single-step preparation of the antifungal natural product incrustoporine **11q** (or analogues such as **11g**).^21^

### One pot β-thiolation

**Scheme 8:**
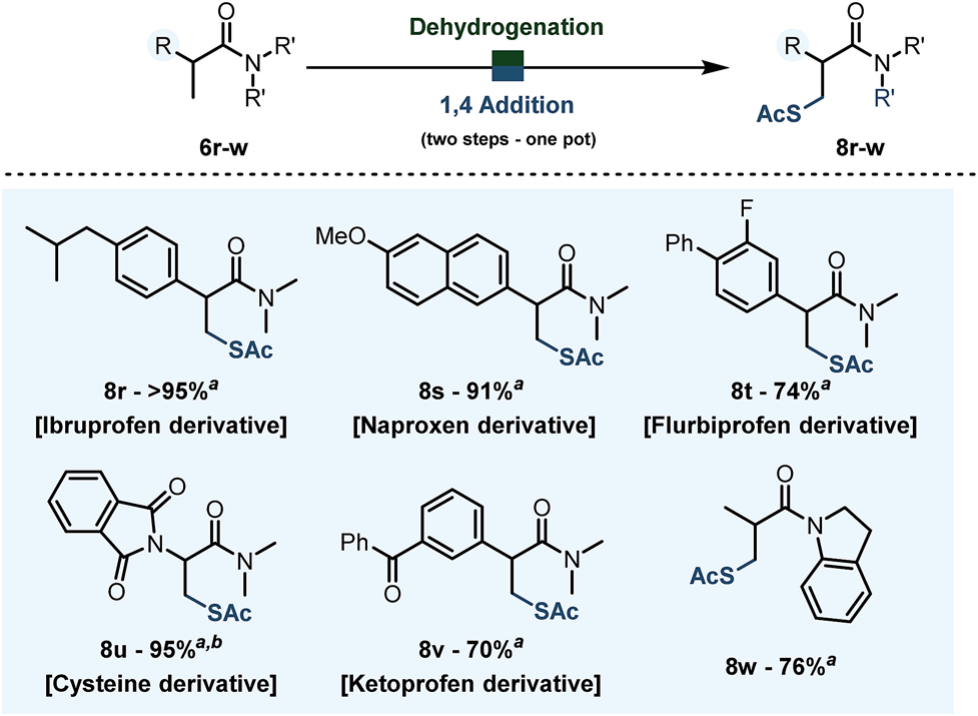
Product scope of the desaturation – 1,4 addition reaction on biologically relevant substrates. Reactions were carried out on a 0.2 mmol scale in 1,2-DCE (0.2 M) with Tf_2_O (1.1 eq.), 2-I Py (2.2 eq.), PNO (1.3 eq.) at 0 °C then 60 °C, AcSH (4.0 eq.), Et_3_N (2.0 eq.). ^*a*^ Yield refers to the isolated product. ^*b*^ Reaction was heated to 60 °C during desaturation.

Finally, when combined with the addition of thioacetic acid under basic conditions, the chemistry reported herein results in formal direct β-thiolation.^22^ As shown in Scheme 8, several derivatives of relevant drugs and biologically active compounds could be thus converted into their β-thiolated analogues. Namely, the dimethylamides of Ibruprofen, Naproxen, Flubriprofen and Ketoprofen all underwent β-thiolation in good to quantitative yields (*cf.*Scheme 6, **8r**,**s**,**t**,**v**). Moreover, this chemistry allows interconversion of proteogenic aminoacid derivatives: an amide derivative of alanine **6u** was converted into the corresponding cysteine derivative **8u** in 76% yield. The metal-free character of these transformations and the substrate complementarity to metal-catalysed processes are worthy of note.

## Conclusion

In conclusion, we have shown herein that a facile and chemoselective dehydrogenation event can be leveraged to achieve a plethora of one-pot oxidations/functionalisations of carboxamides. This results in formal direct α,β-epoxidation, direct conversion to α- or β-ketoamides at will and the one-step interconversion of a simple linear amide into butenolides by formal α,β,γ-oxidation. The ability to deploy this chemistry in a formal β-thiolation hints at its potential utility for late-stage derivatization.

## Conflicts of interest

There are no conflicts to declare.

## Supplementary Material

Supplementary information
