# Supplementary material for: Chemoselective formal β-functionalization of substituted aliphatic amides enabled by a facile stereoselective oxidation event
Source: Chem Sci. 2019 Sep 10;10(42):9836–40. doi: 10.1039/c9sc03715b (PMC6977552; doi:10.1039/c9sc03715b)
Supplement: Supplementary file 1 [file SC-010-C9SC03715B-s001.pdf]

Electronic Supplementary Material (ESI) for Chemical Science.
This journal is © The Royal Society of Chemistry 2019

Chemoselective β-functionalization of substituted amides enabled by a facile stereoselective oxidation event

Adriano Bauer,[a] and Nuno Maulide*[a]

[a] Institute of Organic Chemistry, University of Vienna, Währinger Straße 38, 1090 Vienna (Austria) , E-Mail: nuno.maulide@univie.ac.at, Homepage: http://maulide.univie.ac.at

Table of Contents

I. Preparation of amide starting materials 3
General procedure A 3
General procedure B 3
General procedure C 4
Synthesis of 1-(indolin-1-yl)-2-methylpropan-1-one (6w) 4
II. Desaturation experiments 4
III. Oxidations with *m*CPBA 6
Epoxidation - General procedure D 6
$\beta -$Ketoamide synthesis 6
$\alpha -$Ketoamide synthesis from $\beta -$Ketoamide 7
$\alpha -$Ketoamide synthesis from a simple amide 8
IV. 2-Furanone synthesis and Epoxynitrile cyclization 8
2-Furanone synthesis - General procedure E 8
Reaction of the epoxynitrile 9
V. β - Functionalization *via* 1,4 addition 10
β - Functionalization - General procedure F 10
VI. Characterization 11
VII. Spectra 25

General information

Unless otherwise stated, all glassware was flame-dried before use and all reactions were performed under an atmosphere of argon. All solvents were distilled from appropriate drying agents prior to use. All reagents were used as received from commercial suppliers unless otherwise stated. Trifluoromethanesulfonic anhydride was destilled over P_4O_{10} prior use. Reaction progress was monitored by thin layer chromatography (TLC) performed on aluminium plates coated with silica gel F254 with 0.2 mm thickness. Chromatograms were visualized by fluorescence quenching with UV light at 254 nm or by staining using potassium permanganate. Flash column chromatography was performed using silica gel 60 (230-400 mesh, Merck and co.). Neat infrared spectra were recorded using a Perkin-Elmer Spectrum 100 FT-IR spectrometer. Wavenumbers (ν_{max}) are reported in cm^{-1}. Mass spectra were obtained using a Finnigan MAT 8200 (70 eV) or an Agilent 5973 (70 eV) spectrometer, using electrospray ionization (ESI). All ^{1}H NMR and ^{13}C NMR spectra were recorded using a Bruker AV-400, AV-600 or AV-700 spectrometer at 300 K. Chemical shifts were given in parts per million (ppm, δ), referenced to the solvent peak of $CDCl_3$, defined at δ = 7.26 ppm (^{1}H NMR) and δ = 77.16 (^{13}C NMR). Coupling constants are quoted in Hz (*J*). ^{1}H NMR splitting patterns were designated as singlet (s), doublet (d), triplet (t), quartet (q). Splitting patterns that could not be interpreted or easily visualized were designated as multiplet (m) or broad (br).

I. Preparation of amide starting materials (6a-6w)

General procedure A

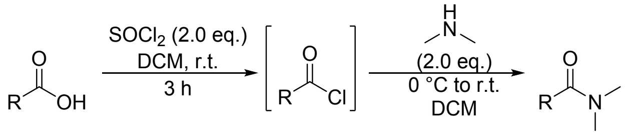

Scheme S 1

The reaction was carried out under air atmosphere. Typical scale was 5-10 mmol. The carboxylic acid was dissolved in DCM (*ca.* 0.3 M) and $SOCl_2$ (2.0 equiv.) was added. The reaction was stirred at room temperature for 3 h. Afterwards, the solvent and excess of $SOCl_2$ were removed under reduced pressure (rotary evaporator) to yield the crude acyl chloride. The crude acyl chloride was dissolved in DCM (*ca.* 0.3 M), cooled to 0 °C and dimethylamine in THF (1.0 M, 2.0 equiv.) was added dropwise. The mixture was stirred for another 2 h at room temperature. After quenching the reaction with an equal volume of aqueous saturated $NaHCO_3$ solution, the aqueous phase was extracted once with DCM. The combined organic layer was dried over Na_2SO_4, filtered and the solvent was removed under reduced pressure. The crude product was purified by column chromatography (Typical eluent was EtOAc: Heptane – 15 : 85 → 40 : 60).

General procedure B

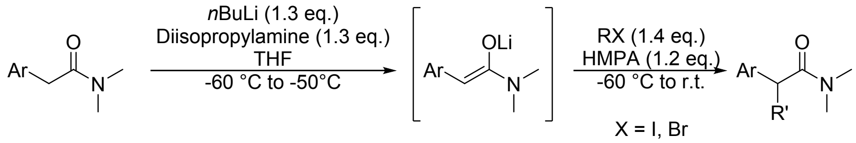

Scheme S 2

Typical scale was 2-5 mmol. Diisopropylamine (1.3 equiv.) was dissolved in THF (*ca.* 0.5 M) and cooled to 0 °C. *n*-BuLi (1.2 equiv., 2.5 M in hexanes) was added dropwise and the reaction was stirred for 15 min at the same temperature. The LDA solution was cooled to -60°C and hexamethylphosphoramide (1.2 equiv.) was added. A solution of the carboxamide (1.0 equiv.) in THF (*ca.* 0.8 M) was added dropwise to the mixture. The temperature was increased to -50 °C over 60 min and then cooled down again to -60°C. A solution of the alkylbromide or alkyliodide in THF (1.4 equiv., 1 M) was added dropwise. Afterwards, the reaction mixture was warmed up to r.t. and stirred for 16 h. After quenching with saturated aqueous NH_4Cl solution, the mixture was diluted with EtOAc. After extraction of the separated aqueous layer with more EtOAc, the combined organic layer was dried over Na_2SO_4, filtered and the solvent was removed under reduced pressure. The crude was purified by column chromatography. The addition of HMPA is generally not necessary when primary iodides are used. (Typical eluent was EtOAc: Heptane – 10 : 90 → 35 : 65).

General procedure C

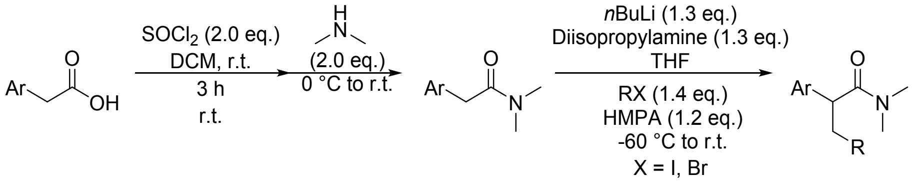

Scheme S 3

More sophisticated amides where synthesized from the acid in two steps, by combining General procedure A and B. (Typical eluent was EtOAc: Heptane – 10 : 90 → 35 : 65).

Synthesis of 1-(indolin-1-yl)-2-methylpropan-1-one (6w)

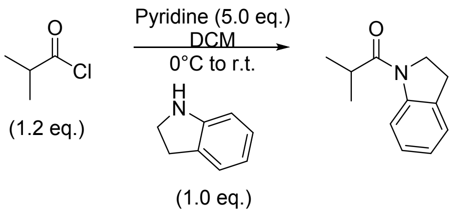

Scheme S 4

Indoline (1.40 mL, 1.0 equiv., 12.5 mmol) was dissolved in DCM (5 mL) and Pyridine (5.0 equiv., 5.1 mL, 62.5 mmol). Thereafter, isobutyryl chloride (1.57 mL, 1.2 equiv., 15 mmol) was added dropwise at 0 °C and the reaction was diluted with more DCM (5 mL). The reaction was stirred for 120 min at room temperature. Afterwards, the mixture was diluted with EtOAc (50 mL), washed with saturated aqueous $NaHCO_3$ (50 mL), aqueous HCl (50 mL) and water (20 mL). The organic layer was dried over Na_2SO_4, filtered and purified by column chromatography, to yield the desired compound as a brown solid (76% ≙ 2160 mg). (Eluent was EtOAc: Heptane – 20 : 80).

II. Desaturation experiments

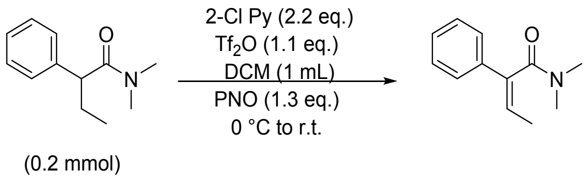

Scheme S 5

The carboxamide (38 mg, 1.0 equiv., 0.20 mmol) was dissolved in DCM (1 mL) and 2-Chloropyridine (42 µL, 2.2 equiv., 0.44 mmol) was added. The clear solution was cooled to 0 °C and under stirring trifluoromethane sulfonic anhydride (37 µL , 1.1 equiv., 0.22 mmol) was added dropwise. The reaction was stirred for 10 min at the same temperature. Then, a Pyridine-

N-Oxide solution in DCM (25 mg in 250 µL, 1.3 eq., 0.26 mmol) was added at once resulting in a strong change in color of the solution. The ice bath was removed and the reaction was stirred at room temperature for further 60 min. The progress of the reaction is readily trackable by TLC. The reaction was quenched with H_2O (*ca*. 5 mL), the aqueous phase was extracted with DCM (3 x 10 mL) and the combined organic layer was dried over Na_2SO_4, filtered and the solvent was removed under reduced pressure. The crude was purified by column chromatography to give a yellow oil (>95% ≙ 37 mg). The configuration of the double bond was determined by ^1H-^1H NOESY spectrometry of a purified sample of **7a**, where a distinct coupling between the vinylic and an aromatic proton was observed (see Figure S1).

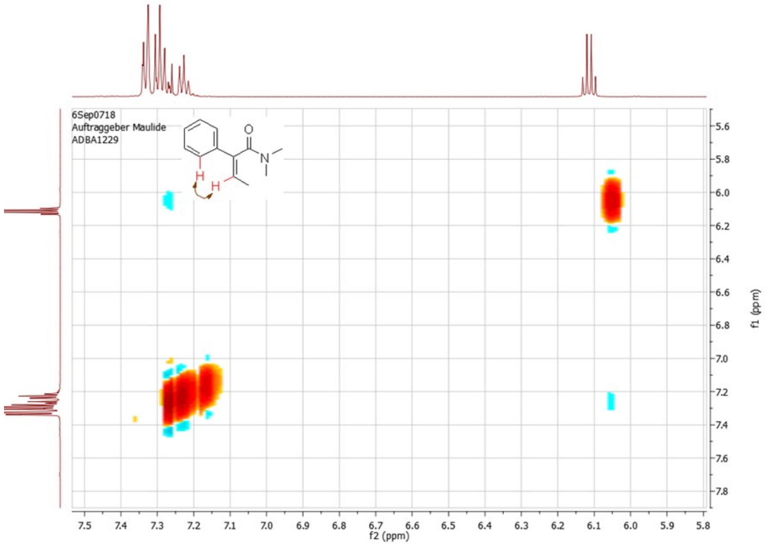

*Figure S 1 – Detailed coupling of two protons in the NOESY spectrum of the alkene product **7b***

The dehydrogenation of **14** to **15a** and **15b** lead to inseparable mixtures. The β-γ desaturated product was identified by comparison of the ^1H NMR chemical shifts which were reported previously: *Canadian Journal of Chemistry,* **2006**, 84, 1397-1410.

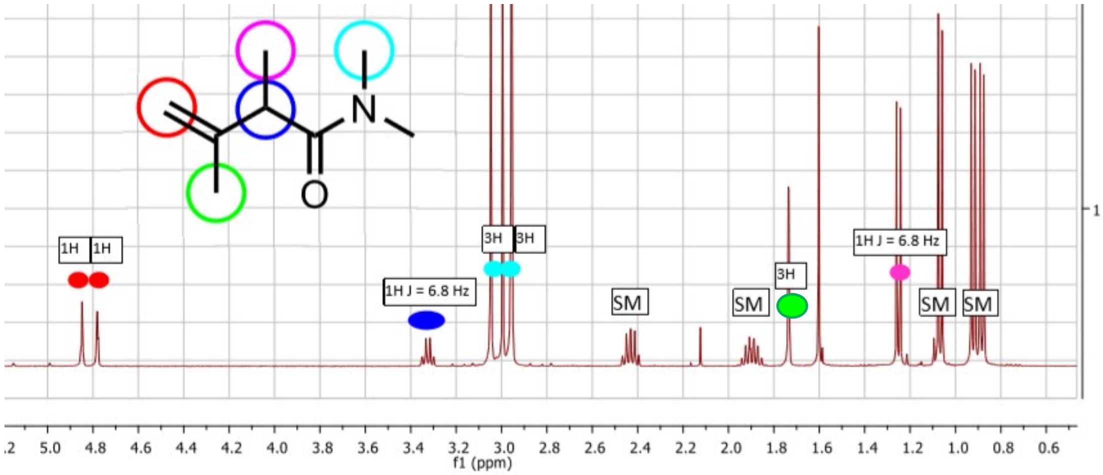

Figure S 2 – NMR spectrum of 15b with 14

III. Oxidations with *m*CPBA

Epoxidation - General procedure D

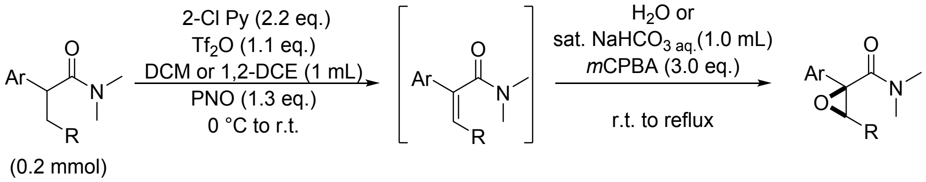

Scheme S 7

The carboxamide (1.0 equiv., 0.20 mmol) was dissolved in DCM or 1,2-Dichloroethane (1,2-DCE) (1 mL) and 2-Chloropyridine (42 µL, 2.2 equiv., 0.44 mmol) was added. The clear solution was cooled to 0 °C and under stirring trifluoromethane sulfonic anhydride (37 µL, 1.1 equiv., 0.22 mmol) was added dropwise. The reaction was stirred for 10 min at the same temperature. Then, a Pyridine-*N*-Oxide solution in DCM (25 mg in 250 µL, 1.3 eq., 0.26 mmol) was added at once resulting in a strong change in color of the solution. The ice bath was removed and the reaction was stirred at room temperature for further 60 min. The progress of the reaction is readily trackable by TLC. Water (1 mL) or saturated aqueous $NaHCO_3$ solution (1 mL) was added to the mixture as an acid scavenger, which was then vigorously stirred for 5 min. Thereafter, *m*CPBA (148 mg, 70%-77%, 3.0 equiv., 0.60 mmol) was added at once. The reaction mixture was stirred for 18 h at a given temperature. After cooling to room temperature, the reaction was diluted with DCM (3-4 mL) and quenched with saturated $Na_2S_2O_3$ solution (2-4mL). After separating the organic from the aqueous phase, the aqueous phase was extracted with DCM (10 mL) and the combined organic layer was washed once with aqueous saturated $NaHCO_3$ (10 mL). Thereafter, the organic phase was dried over Na_2SO_4, filtered and the solvent was removed under reduced pressure. The crude was purified by column chromatography. (Typical eluent was EtOAc: Heptane – 10 : 90 → 35 : 65). While the dehydrogenated amides are

visible under the UV lamp, the epoxides stain typically very well on TLC with "magic stain". (*Helv. Chim. Acta* **1987**, 70, 448-464, page 458.)

β – Ketoamide synthesis

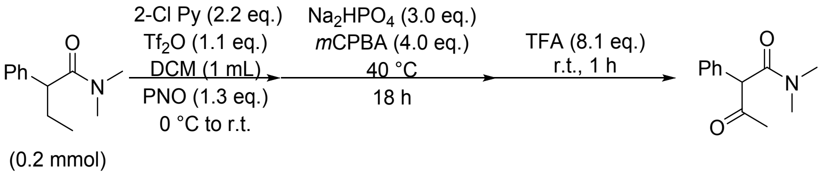

Scheme S 8

The carboxamide (38 mg, 1.0 equiv., 0.20 mmol) was dissolved in DCM (1 mL) and 2-Chloropyridine (42 µL, 2.2 equiv., 0.44 mmol) was added. The clear solution was cooled to 0 °C and under stirring trifluoromethane sulfonic anhydride (37 µL, 1.1 equiv., 0.22 mmol) was added dropwise. The reaction was stirred for 10 min at the same temperature. Then, a Pyridine-*N*-Oxide solution in DCM (25 mg in 250 µL, 1.3 eq.) was added at once resulting in a strong change in color of the solution. The ice bath was removed and the reaction was stirred at room temperature for further 60 min. Thereafter, Na_2HPO_4 (85 mg, 3.0 equiv., 0.6 mmol) was added and the mixture was stirred vigorously for 5 min. Then, *m*CPBA (197 mg, , 70%-77%, 4.0 equiv., 0.8 mmol) was added and the mixture was heated to 40 °C. After stirring for 18 h at the same temperature the reaction was cooled to room temperature and trifluoroacetic acid was added (120 µL , 8.1 equiv., 1.62 mmol,). After 1 h the reaction was quenched with saturated aqueous $Na_2S_2O_3$ solution (2-3 mL) and diluted with DCM (3-4 mL). The separated aqueous phase was extracted with DCM (5 mL) and the combined organic layer was washed with saturated aqueous $NaHCO_3$ (10 mL). The organic layer was dried over Na_2SO_4, filtered and the solvent was removed under reduced pressure. The crude was purified by column chromatography to yield a colorless oil (88% ≙ 36 mg). (Typical eluent was EtOAc: Heptane – 10 : 90 → 35 : 65).

α – Ketoamide synthesis from β – Ketoamide

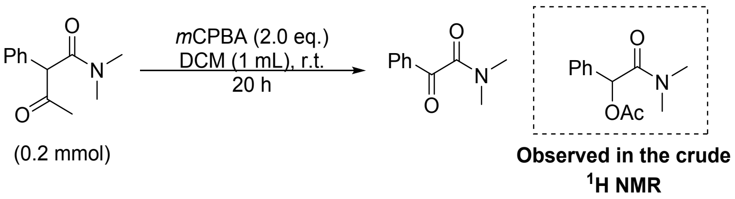

Scheme S 9

The ketoamide (41 mg, 1.0 equiv., 0.20 mmol,) was dissolved in DCM (1 mL) and *mCPBA* was added (98 mg, 70%-77%, 2.0 equiv., 0.4 mmol) and stirred for 20 h at room temperature. The mixture was quenched with saturated $Na_2S_2O_3$ solution (2-3 mL) and diluted with DCM (2-3 mL). After separating the organic from the aqueous phase, the aqueous phase was extracted with

DCM (5 mL) and the combined organic layer was washed once with saturated aqueous solution of $NaHCO_3$ (5 mL). Thereafter, the combined organic phase was dried over Na_2SO_4, filtered and the solvent was removed under reduced pressure. The crude was purified by column chromatography to yield the α-Ketoamide as a colorless oil (65% ≙ 23 mg). (Eluent was EtOAc: Heptane – 15 : 85).

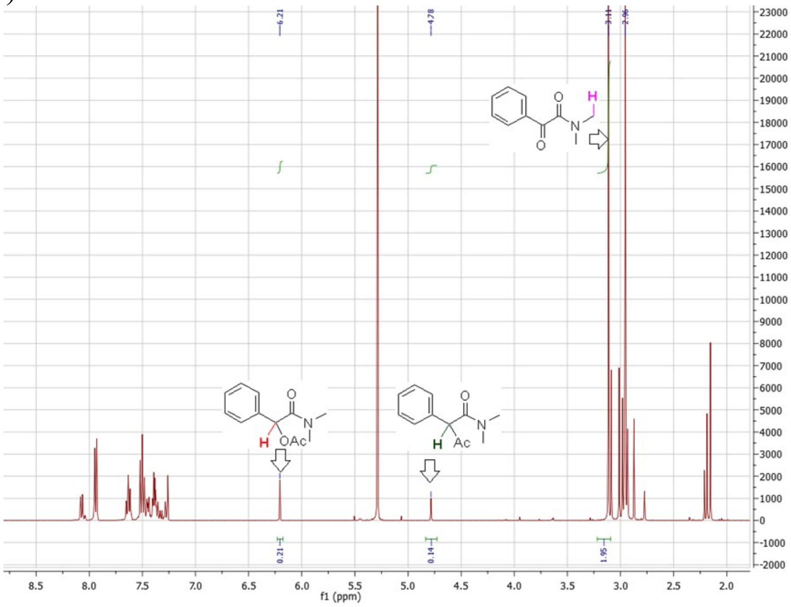

Figure S 3 - Crude ¹H NMR of the α Ketoamide reaction. The acetate peak is highlighted at 6.21 ppm. (See: Advanced Synthesis and Catalysis, **2017***, 359,* 3665 – 3673*)*

α – Ketoamide synthesis from a simple amide

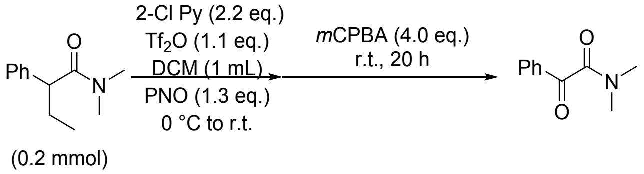

Scheme S 10

The carboxamide (38 mg, 1.0 equiv., 0.20 mmol) was dissolved in DCM (1 mL) and 2-Chloropyridine (42 µL, 2.2 equiv., 0.44 mmol) was added. The clear solution was cooled to 0 °C and under stirring trifluoromethane sulfonic anhydride (37 µL, 1.1 equiv., 0.22 mmol,) was added dropwise. The reaction was stirred for 10 min at the same temperature. Then a Pyridine-*N*-Oxide solution in DCM (25 mg in 250 µL, 1.3 eq.) was added at once resulting in a strong change in color of the solution. The ice bath was removed and the reaction was stirred at room temperature for further 60 min. Then *m*CBPA was added (196 mg, 70%-77%, 4.0 eq., 0.8 mmol) and the mixture was stirred for 20 h at room temperature. After quenching with saturated $Na_2S_2O_3$ solution (2-3 mL) the mixture was diluted with DCM (2-3 mL). After separating the organic from the aqueous phase, the aqueous phase was extracted with DCM (5 mL) and the combined organic layer was washed once with saturated aqueous solution of $NaHCO_3$ (5 mL). Thereafter, the combined organic phase was dried over Na_2SO_4, filtered and the solvent was

removed under reduced pressure. The crude was purified by column chromatography to yield the α-Ketoamide as a colorless oil (65% ≙ 23 mg). (Eluent was EtOAc: Heptane – 15 : 85).

IV. 2-Furanone synthesis and Epoxynitrile cyclization

2-Furanone synthesis - General procedure E

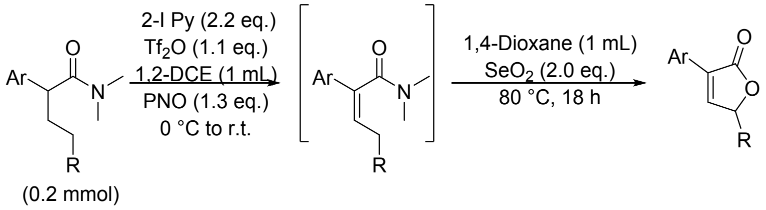

Scheme S 11

The carboxamide (1.0 equiv., 0.20 mmol) was dissolved in 1,2-Dichloroethane (1 mL) and 2-Iodopyridine (2.2 equiv., 0.44 mmol, 47 µL) was added. The clear solution was cooled to 0 °C and under stirring trifluoromethane sulfonic anhydride (1.1 equiv., 0.22 mmpl, 37 µL) was added dropwise. The reaction was stirred for 10 min at the same temperature. Then a Pyridine-*N*-Oxide solution in DCM (25 mg in 250 µL, 1.3 eq.) was added at once resulting in a strong change in color of the solution. The ice bath was removed and the reaction was stirred at room temperature for further 60 min. The mixture was diluted with 1,4-dioxane (1 mL), SeO_2 (44 mg, 2.0 equiv., 0.4 mmol) was added and the resulting suspension was stirred at 80 °C for 18 h. After cooling down to room temperature, the mixture was quenched with water (1 mL), diluted with DCM (5 mL) and filtered through a pad of Celite®. The organic phase was washed with water, dried over Na_2SO_4, filtered and the solvent was removed under reduced pressure. The crude was then purified by column chromatography. (Eluent was EtOAc: Heptane – 15 : 85).

Reaction of the epoxynitrile

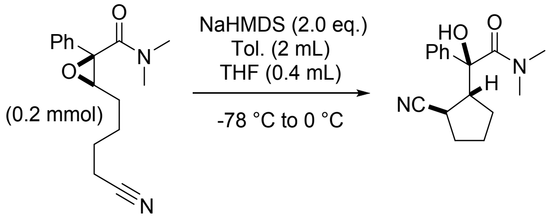

Scheme S 12

The epoxynitrile (55 mg, 1.0 equiv., 0.20 mmol) was dissolved in Toluene (2 mL) and THF (0.2 mL) and the solution was cooled to -78 °C. Then a solution of NaHDMS in THF (2.0 equiv., 2 M, 0.2 mL) was added dropwise. The reaction was stirred for 30 min at the same temperature, then warmed up to 0 °C and stirred for 2.5 h. The reaction was quenched with saturated aqueous NH_4Cl solution (3-4 mL), diluted with EtOAc (3-4 mL) and warmed to room temperature. The separated aqueous phase was extracted with EtOAc (3 x 5 mL). The combined organic layer was dried over Na_2SO_4, filtered and the solvent was removed under reduced pressure. The crude was

purified by column chromatography to yield a colorless oil (66% ≙ 36 mg). The crude ^{1}H NMR shows only one diastereoisomer, and also the purified compound's ^{13}C or ^{1}H NMR spectra provide a similar result, which implies the absence of another diastereoisomer. (Eluent was EtOAc: Heptane – 15 : 85).

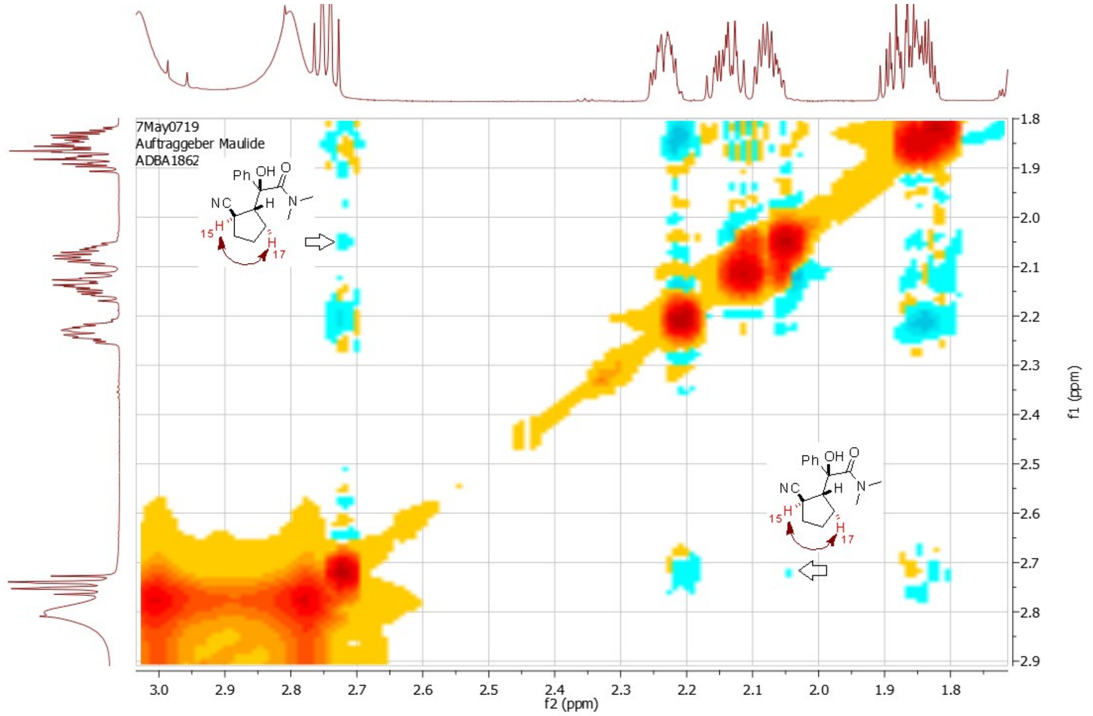

Scheme S 4 – NOESY coupling between 15H and 17H which are assumed to be located in the axial position of the 5-membered ring.

V. β - Functionalization *via* 1,4-addition

β - Functionalization - General procedure F

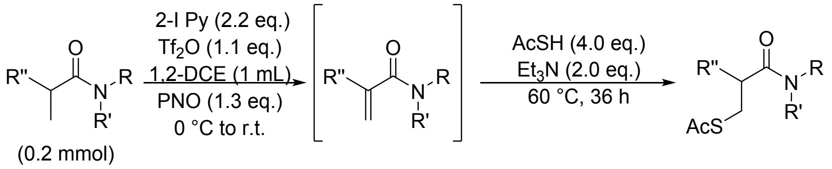

Scheme S 14

The carboxamide (38 mg, 1.0 equiv., 0.20 mmol) was dissolved in 1,2-Dichloroethane (1 mL) and 2-Iodopyridine (47 µL, 2.2 equiv., 0.44 mmol) was added. The clear solution was cooled to 0 °C and under stirring trifluoromethane sulfonic anhydride (37 µL, 1.1 equiv., 0.22 mmol) was added dropwise. The reaction was stirred for 10 min at the same temperature. Then a Pyridine-*N*-Oxide solution in DCM (25 mg in 250 µL, 1.3 eq.) was added at once resulting in a strong

change in color of the solution. The ice bath was removed and the reaction was stirred at room temperature for further 60 min. Then Et_3N (56 µL, 2.0 equiv., 0.44 mmol) and AcSH (57 µL, 0.8 mmol, 4.0 equiv.) was added and the mixture was stirred at 60 °C for 36 h. The reaction was quenched with water (3-4 mL), diluted with DCM (3-4 mL) and the separated aqueous phase was extracted with DCM (3 x 5 mL). The combined organic layer was dried over Na_2SO_4, filtered and the solvent was removed under reduced pressure. The crude was then purified by column chromatography. (Typical eluent was EtOAc: Heptane – 10 : 90 → 35 : 65).
The synthesis of S-(3-(indolin-1-yl)-2-methyl-3-oxopropyl) ethanethioate (**8w**) only proceeds without the use of Et_3N.

VI. Characterization

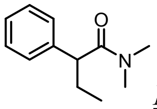
N,N-dimethyl-2-phenylbutanamide (**6a**)

Synthesized from the corresponding commercially available carboxylic acid (General procedure A), 68% ≙ 1874 mg . Yellowish solid.
^{1}H NMR (400 MHz, CDCl$_3$) δ 7.35 – 7.27 (m, 4H), 7.27 – 7.17 (m, 1H), 3.61 (t, *J* = 7.3 Hz, 1H), 2.92 – 2.95 (s, 6H), 2.18 – 2.04 (m, 1H), 1.83 – 1.68 (m, 1H), 0.87 (t, *J* = 7.3 Hz, 3H). **^{13}C NMR** (101 MHz, CDCl$_3$) δ 173.22, 140.28, 128.70, 128.02, 126.82, 50.81, 37.20, 35.91, 28.24, 12.50. **HRMS** (ESI) m/z calculated for [M+Na]$^+$ 214.1208 found 214.1205. **ATR-FTIR** (cm^{-1}): 3027, 3005, 2964, 2929, 2873, 2358, 1637, 1602, 1583, 1490, 1453, 1393, 1334, 1275, 1261, 1146, 1097, 1068, 1031, 964, 906, 850, 764, 749, 700, 634, 607.

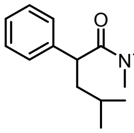
N,N,4-trimethyl-2-phenylpentanamide (**6b**)

Synthesized starting from Phenylacetyl chloride (General procedure C). Alkyliodide used for the alkylation. 60% ≙ 331 mg over 2 steps. White solid.
^{1}H NMR (400 MHz, CDCl$_3$) δ 7.34 – 7.27 (m, 4H), 7.24 – 7.16 (m, 1H), 3.82 (t, *J* = 7.1 Hz, 1H), 2.96 – 2.92 (s, 6H), 2.07 – 1.93 (m, 1H), 1.61 – 1.53 (m, 1H), 1.52 – 1.38 (m, 1H), 0.91 (d, *J* = 6.5 Hz, 3H), 0.87 (d, *J* = 6.6 Hz, 3H). **^{13}C NMR** (101 MHz, CDCl$_3$) δ 173.34, 140.59, 128.78, 128.05, 126.83, 46.51, 44.31, 37.27, 36.07, 25.83, 22.81, 22.71. **HRMS** (ESI) m/z calculated for [M+Na]$^+$ 242.1521 found 242.1519. **ATR-FTIR** (cm^{-1}): 2953, 2933, 2906, 2869, 2855, 1631, 1602, 1584, 1493, 1457, 1412, 1393, 1262, 1171, 1142, 1109, 1072, 750, 722, 703, 633.

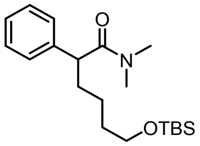
6-((tert-butyldimethylsilyl)oxy)-*N,N*-dimethyl-2-phenylhexanamide (**6c**)

Synthesized starting from Phenylacetyl chloride (General procedure C). Alkyliodide used for the alkylation. 69% ≙ 605 mg over 2 steps. Yellow oil.
^{1}H NMR (600 MHz, CDCl$_3$) δ 7.32 – 7.27 (m, 4H), 7.24 – 7.19 (m, 1H), 3.69 (t, *J* = 7.3 Hz, 1H), 3.60 – 3.52 (m, 2H), 2.93 (s, 6H), 2.12 – 2.00 (m, 1H), 1.78 – 1.67 (m, 1H), 1.58 – 1.40 (m, 2H), 1.36 – 1.14 (m, 2H), 0.86 (s, 9H), 0.01 (d, *J* = 1.2 Hz, 6H). **^{13}C NMR** (151 MHz, CDCl$_3$) δ 173.29, 140.39, 128.81, 128.07, 126.91, 63.22, 49.06, 37.30, 36.04, 35.00, 32.94, 26.12, 24.18, 18.50, -5.13. **HRMS** (ESI) m/z calculated for [M+Na]$^+$ 372.2335 found 372.2231. **ATR-FTIR** (cm^{-1}): 2929, 2857, 1643, 1603, 1493, 1471, 1462, 1393, 1361, 1275, 1258, 1140, 1094, 1032, 1006, 976, 939, 903, 833, 814, 765, 749, 700, 663, 633, 588.

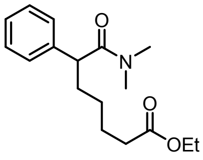
Ethyl 7-(dimethylamino)-7-oxo-6-phenylheptanoate (**6d**)

Synthesized starting from Phenylacetyl chloride (General procedure C). Alkylbromide used for the alkylation. 37% ≙ 270 mg over 2 steps. Yellow oil.

^{1}H NMR (600 MHz, $CDCl_3$) δ 7.31 – 7.25 (m, 4H), 7.23 – 7.19 (m, 1H), 4.08 (q, *J* = 7.1 Hz, 2H), 3.68 (t, *J* = 7.3 Hz, 1H), 2.92 – 2.91 (m, 6H), 2.25 (t, *J* = 7.5 Hz, 2H), 2.12 – 2.05 (m, 1H), 1.73 – 1.66 (m, 1H), 1.65 – 1.56 (m, 2H), 1.36 – 1.27 (m, 1H), 1.25 – 1.14 (m, 4H). **^{13}C NMR** (151 MHz, $CDCl_3$) δ 173.87, 173.10, 140.24, 128.83, 127.98, 126.95, 60.28, 48.86, 37.26, 36.02, 34.84, 34.28, 27.41, 24.98, 14.34. **HRMS** (ESI) m/z calculated for $[M+Na]^+$ 314.1732 found 314.1725. **ATR-FTIR** (cm^{-1}): 3026, 2933, 2864, 1730, 1642, 1493, 1454, 1395, 1374, 1348, 1304, 1260, 1181, 1151, 1091, 1066, 1031, 922, 855, 757, 735, 702, 633, 607, 587, 578, 537.

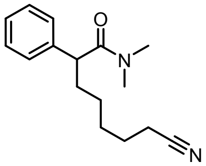

7-cyano-*N,N*-dimethyl-2-phenylheptanamide (**6e**)

Synthesized starting from Phenylacetyl chloride (General procedure C). Alkylbromide used for the alkylation. 61% ≙ 390 mg over 2 steps. Yellow oil.

^{1}H NMR (400 MHz, $CDCl_3$) δ 7.34 – 7.19 (m, 5H), 3.67 (m 1H), 2.93 – 2.91 (m, 6H), 2.29 (t, *J* = 7.0 Hz, 2H), 2.13 – 2.04 (m, 1H), 1.73 – 1.58 (m, 3H), 1.51 – 1.15 (m, 4H). **^{13}C NMR** (101 MHz, $CDCl_3$) δ 172.96, 140.20, 128.89, 127.92, 127.02, 119.87, 49.00, 37.25, 36.03, 34.85, 28.58, 27.01, 25.19, 17.09. **HRMS** (ESI) m/z calculated for $[M+Na]^+$ 281.1630 found 281.1628. **ATR-FTIR** (cm^{-1}): 3007, 2932, 2861, 2244, 1638, 1062, 1583, 1492, 1453, 1394, 1329, 1276, 1261, 1132, 1093, 1066, 1031, 916, 850, 765, 754, 745, 701, 634, 617, 607, 591.

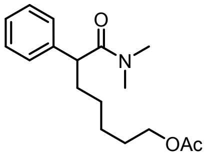

7-(dimethylamino)-7-oxo-6-phenylheptyl acetate (**6f**)

Synthesized starting from Phenylacetyl chloride (General procedure C). Alkylbromide used for the alkylation. 28% ≙ 200 mg over 2 steps. Yellow oil.

^{1}H NMR (700 MHz, $CDCl_3$) δ 7.30 – 7.24 (m, 4H), 7.23 – 7.19 (m, 1H), 4.00 (t, *J* = 6.7 Hz, 2H), 3.66 (t, *J* = 7.2 Hz, 1H), 2.92 – 2.91 (m, 6H), 2.12 – 2.04 (m, 1H), 2.00 (s, 3H), 1.72 – 1.66 (m, 1H), 1.57 (p, *J* = 7.0 Hz, 2H), 1.38 – 1.15 (m, 4H). **^{13}C NMR** (101 MHz, $CDCl_3$) δ 173.17, 171.33, 140.39, 128.85, 128.00, 126.95, 64.63, 49.12, 37.28, 36.05, 35.18, 28.57, 27.65, 26.06, 21.12. **HRMS** (ESI) m/z calculated for $[M+Na]^+$ 314.1732 found 314.1729. **ATR-FTIR** (cm^{-1}): 3026, 2935, 2858, 2366, 2335, 1733, 1640, 1602, 1583, 1492, 1454, 1392, 1365, 1328, 1233, 1141, 1104, 1033, 968, 800, 750, 729, 700, 634, 608.

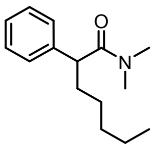

N,N-dimethyl-2-phenylheptanamide (**6g**)

Synthesized starting from Phenylacetyl chloride (General procedure C). Alkyliodide used for the alkylation. 47% ≙ 275 mg over 2 steps. Yellow oil.

^{1}H NMR (600 MHz, $CDCl_3$) δ 7.33 – 7.26 (m, 4H), 7.24 – 7.19 (m, 1H), 3.68 (d, *J* = 7.3 Hz, 1H), 2.93 (s, 6H), 2.14 – 2.03 (m, 1H), 1.75 – 1.65 (m, 1H), 1.36 – 1.12 (m, 6H), 0.85 (t, *J* = 6.9 Hz, 3H). **^{13}C NMR**

(151 MHz, $CDCl_3$) δ 173.38, 140.59, 128.78, 128.06, 126.86, 49.09, 37.30, 36.04, 35.26, 31.95, 27.70, 22.67, 14.20. **HRMS** (ESI) m/z calculated for $[M+Na]^+$ 256.1677 found 256.1682. **ATR-FTIR** (cm^{-1}): 2953, 2925, 2857, 2357, 1640, 1602, 1583, 1493, 1453, 1393, 1327, 1275, 1261, 1143, 1103, 1071, 1058, 1032, 763, 751, 726, 700, 633, 617, 606, 587.

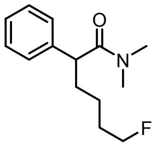

6-fluoro-N,N-dimethyl-2-phenylhexanamide (**6h**)

Synthesized starting from Phenylacetyl chloride (General procedure C). Alkylbromide used for the alkylation. 76% ≙ 448 mg over 2 steps. Colorless oil.

^{1}H NMR (600 MHz, $CDCl_3$) δ 7.33 – 7.27 (m, 4H), 7.25 – 7.19 (m, 1H), 4.40 (dt, *J* = 47.3, 6.1 Hz, 2H), 3.69 (t, *J* = 7.2 Hz, 1H), 2.93 – 2.92 (m, 6H), 2.17 – 2.07 (m, 1H), 1.80 – 1.61 (m, 3H), 1.45 – 1.36 (m, 1H), 1.34 – 1.23 (m, 1H). **^{13}C NMR** (151 MHz, $CDCl_3$) δ 173.02, 140.18, 128.89, 127.98, 127.02, 84.09 (d, *J* = 164.2 Hz), 49.13, 37.26, 36.03, 34.89, 30.55 (d, *J* = 19.6 Hz), 23.71 (d, *J* = 5.5 Hz). **HRMS** (ESI) m/z calculated for $[M+Na]^+$ 260.1427 found 260.1422. **ATR-FTIR** (cm^{-1}): 2939, 1637, 1602, 1493, 1454, 1394, 1252, 1218, 1141, 1101, 1058, 1031, 1003, 980, 938, 852, 754, 700, 665, 633, 593.

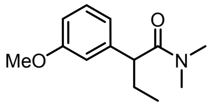

2-(3-methoxyphenyl)-*N,N*-dimethylbutanamide (**6i**)

Synthesized starting from *m*-Methoxyphenylacetic acid (General procedure C). Ethyliodide used for the alkylation. 92% ≙ 510 mg over 2 steps. Yellowish oil.

^{1}H NMR (600 MHz, $CDCl_3$) δ 7.21 (t, *J* = 7.9 Hz, 1H), 6.88 – 6.82 (m, 2H), 6.77 (m, 1H), 3.79 (s, 3H), 3.56 (t, *J* = 7.3 Hz, 1H), 2.93 (s, 6H), 2.12 – 2.03 (m, 1H), 1.78 – 1.70 (m, 1H), 0.86 (t, *J* = 7.4 Hz, 3H). **^{13}C NMR** (151 MHz, $CDCl_3$) δ 173.13, 159.98, 141.91, 129.66, 120.61, 113.59, 112.29, 55.34, 50.92, 37.31, 36.03, 28.23, 12.59. **HRMS** (ESI) m/z calculated for $[M+Na]^+$ 244.1313 found 244.1307. **ATR-FTIR** (cm^{-1}): 2962, 2931, 2874, 1637, 1598, 1583, 1485, 1455, 1436, 1393, 1320, 1296, 1258, 1143, 1087, 1047, 996, 939, 867, 847, 780, 754, 702, 627, 572, 551, 532.

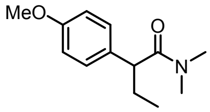

2-(4-methoxyphenyl)-*N,N*-dimethylbutanamide (**6j**)

Synthesized starting from *p*-Methoxyphenylacetic acid (General procedure C). Ethyliodide used for the alkylation. 68% ≙ 422 mg over 2 steps. Yellowish oil.

^{1}H NMR (600 MHz, $CDCl_3$) δ 7.21 – 7.16 (m, 2H), 6.88 – 6.80 (m, 2H), 3.78 (s, 3H), 3.54 (t, *J* = 7.3 Hz, 1H), 2.94 – 2.92 (s, 3H), 2.11 – 2.00 (m, 1H), 1.71 – 1.64 (m, 1H), 0.87 – 0.81 (m, 3H). **^{13}C NMR** (151 MHz, $CDCl_3$) δ 173.66, 158.54, 132.40, 129.07, 114.14, 55.36, 49.92, 37.28, 35.98, 28.28, 12.51. **HRMS** (ESI) m/z calculated for $[M+Na]^+$ 244.1313 found 244.1308. **ATR-FTIR** (cm^{-1}): 3006, 2963, 2932, 2873, 1634, 1583, 1510, 1461, 1394, 1337, 1301, 1275, 1257, 1178, 1147, 1112, 1087, 1033, 910, 850, 823, 788, 763, 750, 728, 645, 619, 559, 529.

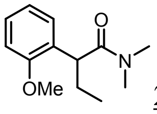

2-(2-methoxyphenyl)-*N,N*-dimethylbutanamide (**6k**)

Synthesized starting from *o*-Methoxyphenylacetic acid (General procedure C). Ethyliodide used for the alkylation. 40% ≙ 220 mg over 2 steps. Yellowish oil.
^{1}H NMR (600 MHz, $CDCl_3$) δ 7.30 (dd, *J* = 7.5, 1.7 Hz, 1H), 7.19 (ddd, *J* = 8.2, 7.5, 1.7 Hz, 1H), 6.91 (td, *J* = 7.5, 1.0 Hz, 1H), 6.86 (d, *J* = 8.2 Hz, 1H), 4.18 (dd, *J* = 7.7, 6.8 Hz, 1H), 3.85 (s, 3H), 2.91 – 2.87 (m, 6H), 2.07 – 1.99 (m, 1H), 1.69 – 1.59 (m, 1H), 0.86 (t, *J* = 7.4 Hz, 3H). **^{13}C NMR** (151 MHz, $CDCl_3$) δ 173.90, 156.06, 128.79, 127.95, 127.62, 121.10, 110.15, 55.42, 41.64, 36.62, 35.66, 27.29, 12.30. **HRMS** (ESI) m/z calculated for $[M+Na]^+$ 244.1313 found 244.1310. **ATR-FTIR** (cm^{-1}): 3006, 2964, 2931, 2873, 1639, 1599, 1491, 1462, 1439, 1394, 1329, 1276, 1261, 1241, 1172, 1150, 1107, 1082, 1052, 1026, 914, 792, 760, 746, 701, 616.

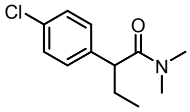

2-(4-chlorophenyl)-*N,N*-dimethylbutanamide (**6l**)

Synthesized starting from *p*-Chlorophenylacetic acid (General procedure C). Ethyliodide used for the alkylation. 66% ≙ 561 mg over 2 steps. Yellowish oil.
^{1}H NMR (600 MHz, $CDCl_3$) δ 7.27 (d, *J* = 8.5 Hz, 2H), 7.22 (d, *J* = 8.4 Hz, 2H), 3.58 (t, *J* = 7.3 Hz, 1H), 2.93 (s, 6H), 2.16 – 2.01 (m, 1H), 1.84 – 1.64 (m, 1H), 0.85 (t, *J* = 7.4 Hz, 3H). **^{13}C NMR** (151 MHz, $CDCl_3$) δ 172.92, 138.77, 132.77, 129.48, 128.93, 50.11, 37.31, 36.05, 28.25, 12.48. **HRMS** (ESI) m/z calculated for $[M+Na]^+$ 248.0812 found 248.0818. **ATR-FTIR** (cm^{-1}): 2966, 2932, 2875, 1636, 1490, 1461, 1395, 1336, 1263, 1216, 1190, 1147, 1089, 1059, 1015, 965, 914, 848, 816.

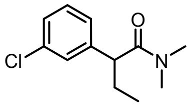

2-(3-chlorophenyl)-*N,N*-dimethylbutanamide (**6m**)

Synthesized starting from *m*-Chlorophenylacetic acid (General procedure C). Ethyliodide used for the alkylation. 52% ≙ 486 mg over 2 steps. Yellowish oil.
^{1}H NMR (600 MHz, $CDCl_3$) δ 7.31 – 7.27 (m, 1H), 7.25 – 7.15 (m, 3H), 3.58 (t, *J* = 7.3 Hz, 1H), 2.94 (s, 6H), 2.15 – 2.03 (m, 1H), 1.86 – 1.66 (m, 1H), 0.86 (t, *J* = 7.4 Hz, 3H). **^{13}C NMR** (151 MHz, $CDCl_3$) δ 172.62, 142.32, 134.57, 130.02, 128.21, 127.20, 126.32, 50.43, 37.34, 36.07, 28.26, 12.51. **HRMS** (ESI) m/z calculated for $[M+Na]^+$ 248.0818 found 248.0813. **ATR-FTIR** (cm^{-1}): 3005, 2966, 2932, 2874, 1637, 1594, 1572, 1473, 1430, 1395, 1336, 1276, 1261, 1217, 1192, 1147, 1100, 1085, 890, 786, 747, 696, 677, 665, 634, 617.

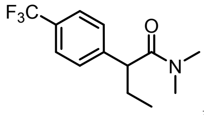

N,N-dimethyl-2-(4-(trifluoromethyl)phenyl)butanamide (**6n**)

Synthesized starting from *p*-(trifluoromethyl)phenylacetic acid (General procedure C). Ethyliodide used for the alkylation. 76% ≙ 490 mg over 2 steps. Yellowish oil.
^{1}H NMR (400 MHz, $CDCl_3$) δ 7.56 (d, *J* = 8.1 Hz, 2H), 7.42 (d, *J* = 8.1 Hz, 2H), 3.68 (t, *J* = 7.3 Hz, 1H), 2.95 (s, 6H), 2.21 – 2.01 (m, 1H), 1.84 – 1.68 (m, 1H), 0.87 (t, *J* = 7.4 Hz, 3H). **^{13}C NMR** (101 MHz, $CDCl_3$) δ 172.53, 144.36, 129.32 (d, *J* = 32.5 Hz), 128.50, 125.74 (q, *J* = 3.8 Hz), 122.97, 50.58, 37.32, 36.09, 28.28, 12.49. **HRMS** (ESI) m/z calculated for $[M+Na]^+$ 282.1082 found 282.1076. **ATR-FTIR** (cm^{-1}): 2967, 2935, 1641, 1618, 1493, 1460, 1396, 1322, 1276, 1261, 1161, 1112, 1087, 1066, 1018, 966, 859, 826, 764, 750, 711, 642, 630, 600.

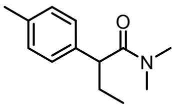
N,N-dimethyl-2-(p-tolyl)butanamide (**6o**)

Synthesized starting from *p*-methylphenylacetic acid (General procedure C). Ethyliodide used for the alkylation. 28% ≙ 210 mg over 2 steps. Yellowish oil.
^{1}H NMR (400 MHz, $CDCl_3$) δ 7.16 (d, *J* = 8.0 Hz, 2H), 7.10 (d, *J* = 8.0 Hz, 2H), 3.55 (t, *J* = 7.3 Hz, 1H), 2.92 (s, 6H), 2.31 (s, 3H), 2.14 – 2.02 (m, 1H), 1.78 – 1.66 (m, 1H), 0.85 (t, *J* = 7.4 Hz, 3H). **^{13}C NMR** (101 MHz, $CDCl_3$) δ 173.48, 137.29, 136.43, 129.44, 127.94, 50.46, 37.26, 35.96, 28.27, 21.14, 12.55. **HRMS** (ESI) m/z calculated for $[M+Na]^+$ 228.1364 found 228.1363. **ATR-FTIR** (cm^{-1}): 3006, 2964, 2925, 2873, 1638, 1512, 1490, 1457, 1393, 1276, 1261, 1146, 1113, 1089, 1056, 810, 764, 748, 722, 688.

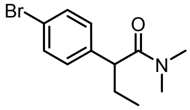
2-(4-bromophenyl)-N,N-dimethylbutanamide (**6p**)

Synthesized starting from *p*-Bromophenylacetic acid (General procedure C). Ethyliodide used for the alkylation. 64% ≙ 435 mg over 2 steps. Yellowish oil.
^{1}H NMR (700 MHz, $CDCl_3$) δ 7.45 – 7.41 (m, 2H), 7.19 – 7.14 (m, 2H), 3.57 (t, *J* = 7.3 Hz, 1H), 2.93 (s, 6H), 2.10 – 2.01 (m, 1H), 1.75 – 1.67 (m, 1H), 0.85 (t, *J* = 7.4 Hz, 3H). **^{13}C NMR** (176 MHz, $CDCl_3$) δ 172.83, 139.27, 131.88, 129.85, 120.84, 50.18, 37.31, 36.06, 28.20, 12.48. **HRMS** (ESI) m/z calculated for $[M+Na]^+$ 292.0313 found 292.0312. **ATR-FTIR** (cm^{-1}): 2963, 2930, 2873, 1736, 1639, 1589, 1487, 1462, 1393, 1373, 1325, 1240, 1189, 1146, 1106, 1083, 0173, 1046, 1010, 846, 813, 751, 724, 712, 637, 626.

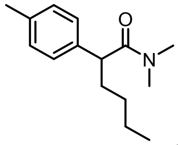
N,N-dimethyl-2-(p-tolyl)hexanamide (**6q**)

Synthesized starting from pTolylacetic acid (General procedure C). Alkylbromide used for the alkylation. >95% ≙ 325 mg over 2 steps. Yellow oil.
^{1}H NMR (600 MHz, $CDCl_3$) δ 7.17 (d, *J* = 7.8 Hz, 2H), 7.11 (d, *J* = 7.8 Hz, 2H), 3.64 (t, *J* = 7.2 Hz, 1H), 2.94 – 2.92 (m, 6H), 2.31 (s, 3H), 2.11 – 2.01 (m, 1H), 1.74 – 1.62 (m, 1H), 1.35 – 1.21 (m, 3H), 1.20 – 1.11 (m, 1H), 0.85 (t, *J* = 7.0 Hz, 3H). **^{13}C NMR** (151 MHz, $CDCl_3$) δ 173.57, 137.53, 136.41, 129.46, 127.91, 48.63, 37.28, 36.01, 35.01, 30.20, 22.83, 21.16, 14.12. **HRMS** (ESI) m/z calculated for $[M+Na]^+$ 256.1677 found 256.1678. **ATR-FTIR** (cm^{-1}): 2954, 2926, 2859, 1640, 1511, 1491, 1457, 1393, 1341, 1263, 1144, 1113, 1096, 1057, 1023, 811.

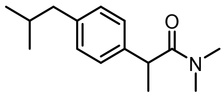
2-(4-isobutylphenyl)-N,N-dimethylpropanamide (**6r**)

Synthesized from the corresponding acid (General Procedure A). >95% ≙ 1167 mg. Yellow oil.
^{1}H NMR (600 MHz, $CDCl_3$) δ 7.15 (d, *J* = 8.0 Hz, 2H), 7.07 (d, *J* = 8.0 Hz, 2H), 3.85 (q, *J* = 6.9 Hz, 1H), 2.95 – 2.89 (m, 6H), 2.43 (d, *J* = 7.2 Hz, 2H), 1.89 – 1.79 (m, 1H), 1.42 (d, *J* = 6.9 Hz, 3H), 0.89 (d, *J* = 6.6 Hz, 6H). **^{13}C NMR** (151 MHz, $CDCl_3$) δ 174.04, 140.22, 139.25, 129.64, 127.15, 45.17, 43.00, 37.30, 36.04, 30.32, 22.54, 22.52, 20.89. **HRMS** (ESI) m/z calculated for $[M+H]^+$ 234.1858 found

234.1854. **ATR-FTIR** (cm^{-1}): 2954, 2927, 2868, 2359, 1643, 1509, 1462, 1393, 1368, 1308, 1275, 1262, 1146, 1119, 1061, 1022, 1001, 848, 805, 765, 750, 695, 645, 612, 588.

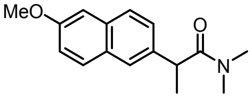
2-(6-methoxynaphthalen-2-yl)-*N,N*-dimethylpropanamide (**6s**)

Synthesized from the corresponding acid (General Procedure A). >95% ≙ 830 mg. Yellow solid.
^{1}H NMR (600 MHz, $CDCl_3$) δ 7.69 (t, *J* = 8.4 Hz, 2H), 7.61 (s, 1H), 7.38 (dd, *J* = 8.4, 1.7 Hz, 1H), 7.16 – 7.09 (m, 2H), 4.00 (q, *J* = 6.9 Hz, 1H), 3.90 (s, 3H), 2.97 – 2.90 (m, 6H), 1.50 (d, *J* = 6.9 Hz, 3H). **^{13}C NMR** (151 MHz, $CDCl_3$) δ 173.87, 157.67, 137.21, 133.56, 129.28, 129.20, 127.55, 126.38, 125.67, 119.09, 105.72, 55.43, 43.36, 37.30, 36.05, 20.91. **HRMS** (ESI) m/z calculated for $[M+Na]^+$ 280.1313 found 280.1303. **ATR-FTIR** (cm^{-1}): 2934, 1638, 1606, 1505, 1485, 1464, 1393, 1264, 1229, 1213, 1195, 1151, 1122, 1075, 1060, 1032, 922, 893, 855, 813.

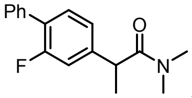
2-(2-fluoro-[1,1'-biphenyl]-4-yl)-*N,N*-dimethylpropanamide (**6t**)

Synthesized from the corresponding acid (General Procedure A). 92% ≙ 820 mg. Yellow oil
^{1}H NMR (600 MHz, $CDCl_3$) δ 7.57 – 7.49 (m, 2H), 7.43 (t, *J* = 7.7 Hz, 2H), 7.41 – 7.33 (m, 2H), 7.15 – 7.08 (m, 2H), 3.94 (q, *J* = 6.9 Hz, 1H), 2.98 – 2.97 (m, 6H), 1.48 (d, *J* = 6.9 Hz, 3H). **^{13}C NMR** (151 MHz, $CDCl_3$) δ 173.23, 159.99 (d, *J* = 248.5 Hz), 143.44 (d, *J* = 7.5 Hz), 135.66, 131.14 (d, *J* = 3.9 Hz), 129.07 (d, *J* = 2.9 Hz), 128.58, 127.77, 127.58 (d, *J* = 13.5 Hz), 123.49 (d, *J* = 3.2 Hz), 115.22 (d, *J* = 23.4 Hz), 42.73, 37.39, 36.15, 20.62. **HRMS** (ESI) m/z calculated for $[M+Na]^+$ 294.1270 found 294.1254. **ATR-FTIR** (cm^{-1}): 3034, 2976, 2933, 1645, 1581, 1562, 1484, 1451, 1417, 1396, 1266, 1223, 1141, 1074, 1011, 949, 914, 878, 834, 766, 752, 724, 699, 643, 618, 595.

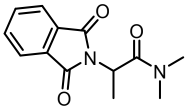
2-(1,3-dioxoisoindolin-2-yl)-*N,N*-dimethylpropanamide (**6u**)

Synthesized from the corresponding acid (General Procedure A). 92% ≙ 1501 mg. White solid.
^{1}H NMR (600 MHz, $CDCl_3$) δ 7.91 – 7.81 (m, 2H), 7.76 – 7.69 (m, 2H), 5.13 (q, *J* = 7.2 Hz, 1H), 2.97 – 2.96 (m, 6H), 1.71 (d, *J* = 7.2 Hz, 3H). **^{13}C NMR** (151 MHz, $CDCl_3$) δ 169.14, 167.87, 134.25, 131.92, 123.57, 47.31, 37.11, 36.36, 15.57. **HRMS** (ESI) m/z calculated for $[M+Na]^+$ 269.0902 found 269.0888. **ATR-FTIR** (cm^{-1}): 2938, 2363, 2343, 1777, 1713, 1657, 1611, 1500, 1468, 1386, 1361, 1260, 1174, 1130, 1084, 1052, 1018, 896, 881, 776, 721.

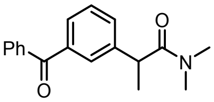
2-(3-benzoylphenyl)-*N,N*-dimethylpropanamide (**6v**)

Synthesized from the corresponding acid (General Procedure A). 87% ≙ 810 mg. Yellow oil.
^{1}H NMR (600 MHz, $CDCl_3$) δ 7.81 – 7.77 (m, 2H), 7.71 (s, 1H), 7.64 (d, *J* = 7.6 Hz, 1H), 7.59 (t, *J* = 7.4 Hz, 1H), 7.55 (d, *J* = 7.8 Hz, 1H), 7.48 (t, *J* = 7.7 Hz, 2H), 7.43 (dd, *J* = 9.6, 5.8 Hz, 1H), 3.99 (q, *J* = 6.9 Hz, 1H), 2.96 – 2.94 (m, 6H), 1.48 (d, *J* = 6.9 Hz, 3H). **^{13}C NMR** (151 MHz, $CDCl_3$) δ 196.72, 173.32, 142.40, 138.19, 137.67, 132.69, 131.42, 130.22, 129.22, 128.96, 128.89, 128.46, 43.08, 37.40, 36.13, 20.70. **HRMS** (ESI) m/z calculated for $[M+Na]^+$ 304.1313 found 304.1303. **ATR-FTIR** (cm^{-1}): 2976, 2932, 1641, 1597, 1579, 1482, 1447, 1395, 1372, 1317, 1280, 1198, 1178, 1149, 1073, 999, 958, 939, 825, 783, 720, 699, 646, 604.

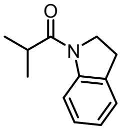

1-(indolin-1-yl)-2-methylpropan-1-one (**6w**)

Synthesized from isobutyryl chloride. 76% ≙ 2160 mg. Brown solid.

^{1}H NMR (600 MHz, $CDCl_3$) δ 8.27 (d, *J* = 7.8 Hz, 1H), 7.22 – 7.15 (m, 2H), 7.00 (t, *J* = 7.4 Hz, 1H), 4.12 (t, *J* = 8.5 Hz, 2H), 3.19 (t, *J* = 8.2 Hz, 2H), 2.73 –2.83 (m, 1H), 1.23 (d, *J* = 6.7 Hz, 6H). **^{13}C NMR** (151 MHz, $CDCl_3$) δ 175.75, 143.39, 131.28, 127.63, 124.56, 123.62, 117.42, 47.92, 33.54, 28.19, 19.26. **HRMS** (ESI) m/z calculated for $[M+Na]^+$ 212.1051 found 212.1053. **ATR-FTIR** (cm^{-1}): 2968, 2934, 2875, 2352, 1732, 1656, 1599, 1481, 1462, 1407, 1363, 1337, 1308, 1266, 1228, 1164, 1105, 1081, 926.

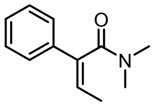

(*Z*)-*N,N*-dimethyl-2-phenylbut-2-enamiden (**7a**)

Synthesized using the desaturation procedure. >95% ≙ 37 mg. Yellow oil.

^{1}H NMR (600 MHz, $CDCl_3$) δ 7.36 – 7.33 (m, 2H), 7.33 – 7.28 (m, 2H), 7.26 – 7.22 (m, 1H), 6.13 (q, *J* = 7.0 Hz, 1H), 3.10 (s, 3H), 2.91 (s, 3H), 1.82 (d, *J* = 7.0 Hz, 3H). **^{13}C NMR** (151 MHz, $CDCl_3$) δ 170.32, 138.52, 136.36, 128.83, 127.73, 125.45, 124.09, 37.70, 34.35, 15.44. **HRMS** (ESI) m/z calculated for $[M+H]^+$ 190.1228 found 190.1226. **ATR-FTIR** (cm^{-1}): 2988, 2925, 1622, 1496, 1441, 1395, 1333, 1275, 1261, 1147, 1113, 1076, 1057, 1033, 980, 872, 751, 744, 709, 693, 664, 623.

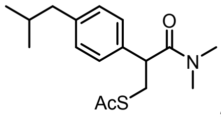

S-(3-(dimethylamino)-2-(4-isobutylphenyl)-3-oxopropyl) ethanethioate (**8r**)

Synthesized by using the procedure for β-functionalization (General procedure F). >95% ≙ 61 mg. Brown solid.

^{1}H NMR (600 MHz, $CDCl_3$) δ 7.19 (d, *J* = 8.0 Hz, 2H), 7.09 (d, *J* = 8.0 Hz, 2H), 3.93 (dd, *J* = 9.5, 5.1 Hz, 1H), 3.33 (dd, *J* = 13.3, 9.5 Hz, 1H), 3.26 (dd, *J* = 13.3, 5.1 Hz, 1H), 2.95 (s, 3H), 2.82 (s, 3H), 2.43 (d, *J* = 7.2 Hz, 2H), 2.30 (s, 3H), 1.83 (hept, *J* = 6.6 Hz, 1H), 0.88 (dd, *J* = 6.6, 0.7 Hz, 6H). **^{13}C NMR** (151 MHz, $CDCl_3$) δ 196.74, 171.74, 141.14, 135.57, 129.79, 127.65, 49.27, 45.14, 37.17, 35.99, 33.80, 30.70, 30.27, 22.48, 22.47. **HRMS** (ESI) m/z calculated for $[M+Na]^+$ 330.1504 found 330.1497. **ATR-FTIR** (cm^{-1}): 3007, 2989, 2955, 1686, 1639, 1507, 1464, 1398, 1354, 1276, 1261, 1135, 968, 908, 764, 751, 727, 629, 558, 540.

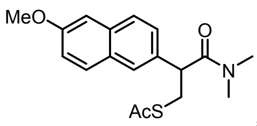

S-(3-(dimethylamino)-2-(6-methoxynaphthalen-2-yl)-3-oxopropyl) ethanethioate (**8s**)

Synthesized by using the procedure for β-functionalization (General procedure F). 91% ≙ 60 mg. Brown solid.

^{1}H NMR (600 MHz, $CDCl_3$) δ 7.73 – 7.69 (m, 2H), 7.66 (s, 1H), 7.42 (dd, *J* = 8.4, 1.8 Hz, 1H), 7.15 (dd, *J* = 8.9, 2.5 Hz, 1H), 7.11 (d, *J* = 2.5 Hz, 1H), 4.10 (dd, *J* = 9.4, 5.2 Hz, 1H), 3.91 (s, 3H), 3.42 (dd, *J* = 13.4, 9.4 Hz, 1H), 3.34 (dd, *J* = 13.4, 5.2 Hz, 1H), 2.98 (s, 3H), 2.84 (s, 3H), 2.33 (s, 3H). **^{13}C NMR** (151 MHz, $CDCl_3$) δ 196.84, 171.68, 157.97, 133.99, 133.49, 129.45, 129.10, 127.78, 126.58, 126.47, 119.33, 105.72, 55.46, 49.58, 37.23, 36.04, 33.84, 30.76. **HRMS** (ESI) m/z calculated for $[M+Na]^+$ 354.1140

found 354.1134. **ATR-FTIR** (cm^{-1}): 2938, 1683, 1636, 1604, 1503, 1483, 1461, 1392, 1352, 1264, 1231, 1213, 1172, 1134, 1053, 1029, 962, 926, 894, 853, 813, 764, 732, 701, 671, 628, 575, 525.

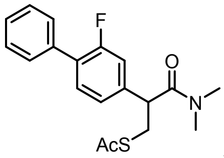

S-(3-(dimethylamino)-2-(2-fluoro-[1,1'-biphenyl]-4-yl)-3-oxopropyl) ethanethioate (**8t**)

Synthesized by using the procedure for β-functionalization (General procedure F). 74% ≙ 51 mg. Brown oil.

^{1}H NMR (600 MHz, $CDCl_3$) δ 7.55 – 7.51 (m, 2H), 7.46 – 7.39 (m, 3H), 7.38 – 7.35 (m, 1H), 7.16 (ddd, *J* = 12.9, 9.6, 1.7 Hz, 2H), 4.03 (dd, *J* = 9.3, 5.3 Hz, 1H), 3.40 (dd, *J* = 13.4, 9.3 Hz, 1H), 3.30 (dd, *J* = 13.4, 5.3 Hz, 1H), 3.00 (s, 3H), 2.91 (s, 3H), 2.34 (s, 3H). **^{13}C NMR** (151 MHz, $CDCl_3$) δ 196.70, 171.04, 160.00 (d, *J* = 249.3 Hz), 139.65 (d, *J* = 7.6 Hz), 135.41, 131.35 (d, *J* = 3.9 Hz), 129.08, 129.06, 128.62, 128.46 (d, *J* = 13.6 Hz), 127.93, 124.08 (d, *J* = 3.3 Hz), 115.72 (d, *J* = 23.8 Hz), 48.88, 37.32, 36.15, 33.68, 30.79. **HRMS** (ESI) m/z calculated for $[M+Na]^+$ 368.1096 found 368.1081. **ATR-FTIR** (cm^{-1}): 2935, 1687, 1643, 1581, 1562, 1483, 1451,1416.

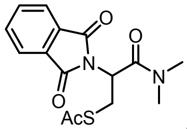

S-(3-(dimethylamino)-2-(1,3-dioxoisoindolin-2-yl)-3-oxopropyl) ethanethioate (**8u**)

Synthesized by using the procedure for β-functionalization (General procedure F). >95% ≙ 64 mg. Brown solid.

^{1}H NMR (600 MHz, $CDCl_3$) δ 7.85 – 7.82 (m, 2H), 7.74 – 7.71 (m, 2H), 5.19 (dd, *J* = 10.4, 5.1 Hz, 1H), 3.79 (dd, *J* = 14.2, 10.4 Hz, 1H), 3.66 (dd, *J* = 14.2, 5.1 Hz, 1H), 3.10 (s, 3H), 2.95 (d, *J* = 5.5 Hz, 3H), 2.28 (s, 3H). **^{13}C NMR** (151 MHz, $CDCl_3$) δ 195.97, 167.91, 167.23, 134.38, 131.62, 123.69, 123.52, 51.39, 37.15, 37.06, 36.34, 30.52, 28.47. **HRMS** (ESI) m/z calculated for $[M+Na]^+$ 343.0728 found 343.0718. **ATR-FTIR** (cm^{-1}): 1773, 1714, 1691, 1655, 1613, 1495, 1468, 1379, 1356, 1300, 1275, 1261, 1134, 1099, 1086, 1063, 960, 940, 912, 890, 764, 749, 716, 699, 646, 621.

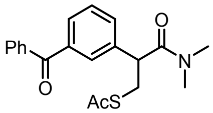

S-(2-(3-benzoylphenyl)-3-(dimethylamino)-3-oxopropyl) ethanethioate (**8v**)

Synthesized by using the procedure for β-functionalization (General procedure F). 70% ≙ 50 mg. Brown oil.

^{1}H NMR (600 MHz, $CDCl_3$) δ 7.79 – 7.75 (m, 2H), 7.72 – 7.69 (m, 1H), 7.69 – 7.65 (m, 1H), 7.60 – 7.54 (m, 2H), 7.49 – 7.41 (m, 3H), 4.05 (dd, *J* = 8.8, 5.7 Hz, 1H), 3.40 (dd, *J* = 13.4, 8.8 Hz, 1H), 3.28 (dd, *J* = 13.4, 5.7 Hz, 1H), 2.95 (s, 3H), 2.86 (s, 3H), 2.30 (s, 3H). **^{13}C NMR** (151 MHz, $CDCl_3$) δ 196.34, 196.33, 171.05, 138.60, 138.34, 137.39, 132.76, 131.76, 130.17, 129.67, 129.45, 129.12, 128.43, 49.07, 37.25, 36.05, 33.64, 30.67. **HRMS** (ESI) m/z calculated for $[M+Na]^+$ 378.1140 found 378.1140. **ATR-FTIR** (cm^{-1}): 2933, 2249, 1687, 1640, 1597, 1579, 1493, 1447, 1398, 1354, 1318, 1354, 1318, 1279, 1261, 1223, 1197, 1133, 1055, 1001, 963, 950, 908, 867, 819, 717, 699, 646, 628.

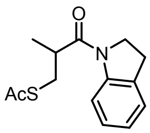

S-(3-(indolin-1-yl)-2-methyl-3-oxopropyl) ethanethioate (**8w**)

Synthesized by using the procedure for β-functionalization (General procedure F), without using triethylamine. 76%. Brown oil.
^{1}H NMR (600 MHz, $CDCl_3$) δ 8.26 (d, *J* = 8.0 Hz, 1H), 7.19 (t, *J* = 8.0 Hz, 2H), 7.02 (t, *J* = 7.4 Hz, 1H), 4.20 (td, *J* = 10.0, 7.1 Hz, 1H), 4.05 (td, *J* = 10.0, 7.1 Hz, 1H), 3.27 – 3.15 (m, 3H), 3.00 (dd, *J* = 13.5, 6.5 Hz, 1H), 2.99 – 2.90 (m, 1H), 2.33 (s, 3H), 1.29 (d, *J* = 6.8 Hz, 3H). **^{13}C NMR** (151 MHz, $CDCl_3$) δ 196.43, 173.09, 143.04, 131.49, 127.67, 124.66, 124.01, 117.53, 48.11, 39.63, 32.78, 30.77, 28.14, 17.25. **HRMS** (ESI) m/z calculated for $[M+Na]^+$ 286.0878 found 286.0883. **ATR-FTIR** (cm^{-1}): 2970, 1688, 1651, 1598, 1481, 1461, 1411, 1372, 1338, 1313, 1283, 1136, 1103, 1025, 957.

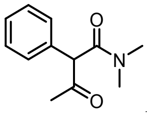

N,N-dimethyl-3-oxo-2-phenylbutanamide (**9a**)
Synthesized following the procedure for the β–ketoamide synthesis. 88% ≙ 36 mg. Colorless oil.
^{1}H NMR (600 MHz, $CDCl_3$) δ 7.40 – 7.35 (m, 2H), 7.35 – 7.30 (m, 1H), 7.30 – 7.27 (m, 2H), 4.78 (s, 1H), 2.99 (s, 3H), 2.88 (s, 3H), 2.19 (s, 3H). **^{13}C NMR** (151 MHz, $CDCl_3$) δ 203.69, 168.95, 133.77, 129.25, 128.99, 128.16, 64.13, 37.74, 35.84, 29.07. **HRMS** (ESI) m/z calculated for $[M+Na]^+$ 220.1000 found 220.0990. **ATR-FTIR** (cm^{-1}): 3006, 2936, 2360, 2342, 1727, 1713, 1634, 1584, 1496, 1454, 1396, 1354, 1276, 1261, 1161, 1134, 1077, 1060, 1033, 763, 750, 701, 700, 633.

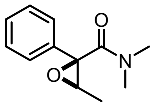

(2*R**,3*R**)-*N,N*,3-trimethyl-2-phenyloxirane-2-carboxamide (**10a**)
Synthesized following the procedure for the epoxidation (General procedure D) in DCM with water as the acid quencher. Epoxidation at room temperature. 93% ≙ 38 mg. Colorless solid. The gram scale synthesis follows the same procedure and yields the product in the same appearance. 86% ≙ 1410 mg.
^{1}H NMR (400 MHz, $CDCl_3$) δ 7.46 – 7.41 (m, 2H), 7.37 – 7.28 (m, 3H), 3.15 (q, *J* = 5.3 Hz, 1H), 2.99 (s, 3H), 2.98 (s, 3H), 1.45 (d, *J* = 5.4 Hz, 3H). **^{13}C NMR** (151 MHz, $CDCl_3$) δ 167.11, 136.28, 128.75, 128.40, 125.14, 64.51, 37.02, 35.22, 16.85. **HRMS** (ESI) m/z calculated for $[M+Na]^+$ 228.1000 found 228.0993. **ATR-FTIR** (cm^{-1}): 2930, 2243, 1639, 1495, 1448, 1402, 1270, 1172, 1126, 1043, 1029, 980, 956, 907, 857, 797, 756, 727, 697, 646, 626, 583, 553.

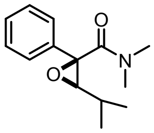

(2*R**,3*R**)-3-isopropyl-*N,N*-dimethyl-2-phenyloxirane-2-carboxamide (**10b**)
Synthesized following the procedure for the epoxidation (General procedure D) in DCM with water as the acid quencher. Epoxidation at room temperature. 77% ≙ 36 mg. Yellow solid.
^{1}H NMR (400 MHz, $CDCl_3$) δ 7.41 – 7.34 (m, 2H), 7.31 – 7.17 (m, 3H), 2.91 – 2.89 (m, 6H), 2.59 (d, *J* = 9.1 Hz, 1H), 1.54 – 1.34 (m, 1H), 1.06 (d, *J* = 6.7 Hz, 3H), 1.02 (d, *J* = 6.7 Hz, 3H). **^{13}C NMR** (101 MHz, $CDCl_3$) δ 167.13, 136.79, 128.71, 128.25, 125.05, 73.87, 65.43, 37.01, 35.31, 30.68, 19.90, 18.50. **HRMS** (ESI) m/z calculated for $[M+Na]^+$ 256.1313 found 256.1305. **ATR-FTIR** (cm^{-1}): 2961, 2869, 1643, 1495, 1471, 1448, 1400, 1383, 1364, 1266, 1244, 1161, 1125, 1093, 1076, 1061, 1032, 986, 953, 934, 908, 886, 867, 827, 760, 733, 698, 628, 611.

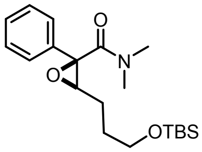

(2*R**,3*R**)-3-(3-((tert-butyldimethylsilyl)oxy)propyl)-*N,N*-dimethyl-2-phenyloxirane-2-carboxamide (**10c**) Synthesized following the procedure for the epoxidation (General procedure D) in DCM with aqueous $NaHCO_3$ as the acid quencher. Epoxidation at room temperature. 41% ≙ 31 mg. Yellow oil.

^{1}H NMR (400 MHz, $CDCl_3$) δ 7.46 – 7.41 (m, 2H), 7.37 – 7.28 (m, 3H), 3.67 (t, *J* = 6.3 Hz, 2H), 3.11 – 3.02 (m, 1H), 2.98 – 2.97 (m, 6H), 1.96 – 1.85 (m, 1H), 1.84 – 1.72 (m, 2H), 1.60 – 1.41 (m, 1H), 0.92 – 0.83 (m, 9H), 0.04 (d, *J* = 1.5 Hz, 6H). **^{13}C NMR** (101 MHz, $CDCl_3$) δ 167.00, 136.33, 128.59, 128.21, 125.02, 68.31, 64.48, 62.65, 36.88, 35.11, 29.49, 27.94, 25.92, 18.27, -5.31. **HRMS** (ESI) m/z calculated for $[M+Na]^+$ 386.2127 found 386.2124 **ATR-FTIR** (cm^{-1}): 2952, 2928, 2894, 2856, 2365, 1649, 1496, 1471, 1462, 1450, 1400, 1361, 1252, 1157, 1094, 1031, 1006, 979, 957, 936, 907, 834, 809, 774, 733, 698, 687, 662, 627, 614.

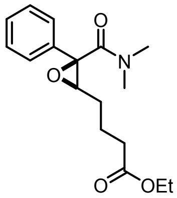

Ethyl 4-((2*R**,3*R**)-(3-(dimethylcarbamoyl)-3-phenyloxiran-2-yl)butanoate (**10d**) Synthesized following the procedure for the epoxidation (General procedure D) in DCM with water as the acid quencher. Epoxidation at room temperature. 81% ≙ 49 mg. Yellow oil.

^{1}H NMR (400 MHz, $CDCl_3$) δ 7.45 – 7.40 (m, 2H), 7.38 – 7.28 (m, 3H), 4.12 (q, *J* = 7.1 Hz, 2H), 3.02 – 2.99 (m, 1H), 2.98 – 2.96 (m, 6H), 2.43 – 2.37 (m, 2H), 1.98 – 1.83 (m, 3H), 1.52 – 1.40 (m, 1H), 1.24 (t, *J* = 7.1 Hz, 3H). **^{13}C NMR** (101 MHz, $CDCl_3$) δ 173.37, 167.03, 136.26, 128.78, 128.43, 125.09, 68.11, 64.44, 60.43, 37.00, 35.27, 34.05, 30.80, 21.95, 14.37. **HRMS** (ESI) m/z calculated for $[M+Na]^+$ 328.1525 found 328.1509. **ATR-FTIR** (cm^{-1}): 2934, 1730, 1643, 1496, 1449, 1400, 1375, 1300, 1248, 1165, 1113, 1060, 1029, 971, 935, 906, 852, 800, 760, 732, 699, 626, 578, 528.

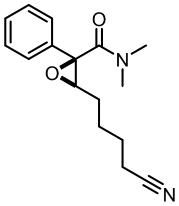

(2*R**,3*R**)-3-(4-cyanobutyl)-*N,N*-dimethyl-2-phenyloxirane-2-carboxamide (**10e**) Synthesized following the procedure for the epoxidation (General procedure D) in DCM with water as the acid quencher. Epoxidation at room temperature. 77% ≙ 42 mg. Yellow oil.

^{1}H NMR (400 MHz, $CDCl_3$) δ 7.43 – 7.39 (m, 2H), 7.37 – 7.27 (m, 3H), 2.99 – 2.98 (m, 1H), 2.97 – 2.96 (m, 6H), 2.39 – 2.33 (m, 2H), 1.94 – 1.84 (m, 1H), 1.81 – 1.67 (m, 4H), 1.54 – 1.44 (m, 1H). **^{13}C NMR** (101 MHz, $CDCl_3$) δ 166.94, 136.07, 128.79, 128.47, 124.97, 119.63, 68.01, 64.43, 36.97, 35.25, 30.46, 25.57, 25.21, 17.12. **HRMS** (ESI) m/z calculated for $[M+Na]^+$ 295.1422 found 295.1419. **ATR-FTIR** (cm^{-1}): 2946, 1643, 1496, 1449, 1402, 1265, 1154, 1115, 1061, 979, 935, 908, 730, 699, 648, 626.

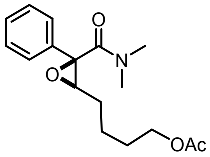

4-((2R*,3R*)-(3-(dimethylcarbamoyl)-3-phenyloxiran-2-yl)butyl acetate (**10f**)

Synthesized following the procedure for the epoxidation (General procedure D) in DCM with aqueous $NaHCO_3$ as the acid quencher. Epoxidation at room temperature. 52% ≙ 32 mg. Yellow oil.
^{1}H NMR (600 MHz, $CDCl_3$) δ 7.45 – 7.40 (m, 2H), 7.37 – 7.27 (m, 3H), 4.08 (t, J = 6.5 Hz, 2H), 3.01 – 2.99 (m, 1H), 2.98 (s, 3H), 2.97 (s, 3H), 2.04 (s, 3H), 1.96 – 1.89 (m, 1H), 1.75 – 1.68 (m, 2H), 1.67 – 1.59 (m, 2H), 1.49 – 1.41 (m, 1H). **^{13}C NMR** (151 MHz, $CDCl_3$) δ 171.34, 167.06, 136.30, 128.78, 128.42, 125.06, 68.43, 64.46, 64.40, 37.02, 35.27, 31.04, 28.49, 23.04, 21.13. **HRMS** (ESI) m/z calculated for $[M+Na]^+$ 328.1525 found 328.1522. **ATR-FTIR** (cm^{-1}): 2938, 1734, 1643, 1496, 1450, 1400, 1239, 1157, 1118, 1034, 975, 934, 908, 840, 763, 730, 699, 646, 627, 609, 583, 534.

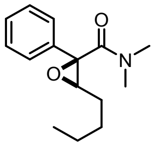

(2R*,3R*)-3-butyl-N,N-dimethyl-2-phenyloxirane-2-carboxamide (**10g**)

Synthesized following the procedure for the epoxidation (General procedure D) in DCM with water as the acid quencher. Epoxidation at room temperature. 71% ≙ 35 mg. Yellow oil.
^{1}H NMR (400 MHz, $CDCl_3$) δ 7.47 – 7.39 (m, 2H), 7.37 – 7.27 (m, 3H), 3.02 – 2.98 (m, 1H), 2.97 (s, 6H), 1.94 – 1.84 (m, 1H), 1.59 – 1.48 (m, 2H), 1.39 (td, J = 14.6, 7.3 Hz, 3H), 0.92 (t, J = 7.3 Hz, 3H). **^{13}C NMR** (101 MHz, $CDCl_3$) δ 167.19, 136.53, 128.72, 128.31, 125.11, 68.72, 64.47, 37.00, 35.25, 31.11, 28.67, 22.63, 14.08. **HRMS** (ESI) m/z calculated for $[M+Na]^+$ 270.1470 found 270.1465. **ATR-FTIR** (cm^{-1}): 2958, 2932, 2872, 2366, 1643, 1495, 1450, 1400, 1267, 1158, 1124, 1092, 1062, 1031, 973, 937, 906, 760, 733, 698, 687, 627, 612.

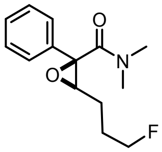

(2R*,3R*)-3-(3-fluoropropyl)-N,N-dimethyl-2-phenyloxirane-2-carboxamide (**10h**)

Synthesized following the procedure for the epoxidation (General procedure D) in DCM with water as the acid quencher. Epoxidation at room temperature. 76% ≙ 38 mg. Colorless oil.
^{1}H NMR (600 MHz, $CDCl_3$) δ 7.47 – 7.39 (m, 2H), 7.37 – 7.28 (m, 3H), 4.51 (dt, J = 47.0, 5.7 Hz, 2H), 3.05 – 3.03 (m, 1H), 2.98 (s, 3H), 2.97 (s, 3H), 2.07 – 1.87 (m, 3H), 1.65 – 1.53 (m, 1H). **^{13}C NMR** (151 MHz, $CDCl_3$) δ 166.93, 136.16, 128.78, 128.46, 125.06, 83.67 (d, J = 165.2 Hz), 67.89, 64.62, 36.98, 35.25, 27.50 (d, J = 5.2 Hz), 27.32 (d, J = 20.0 Hz). **HRMS** (ESI) m/z calculated for $[M+Na]^+$ 274.1219 found 274.1214. **ATR-FTIR** (cm^{-1}): 3007, 2988, 1642, 1497, 1449, 1399, 1276, 1261, 1157, 1117, 1062, 1041, 996, 975, 936, 908, 750, 699, 626.

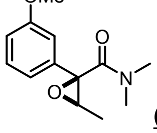

(2R*,3R*)-2-(3-methoxyphenyl)-N,N,3-trimethyloxirane-2-carboxamide (**10i**)

Synthesized following the procedure for the epoxidation (General procedure D) in DCM with aqueous $NaHCO_3$ as the acid quencher. Epoxidation at room temperature. 55% ≙ 26 mg. Yellow oil. Decomposes at room temperature.
^{1}H NMR (400 MHz, $CDCl_3$) δ 7.18 (t, *J* = 7.8 Hz, 1H), 7.00 – 6.94 (m, 1H), 6.93 – 6.90 (m, 1H), 6.77 (ddd, *J* = 8.2, 2.6, 0.9 Hz, 1H), 3.73 (s, 3H), 3.06 (q, *J* = 5.4 Hz, 1H), 2.91 (s, 6H), 1.37 (d, *J* = 5.4 Hz, 3H). **^{13}C NMR** (101 MHz, $CDCl_3$) δ 167.00, 160.09, 137.98, 129.82, 117.55, 114.14, 110.37, 64.44, 55.40, 55.39, 36.99, 35.22, 16.81. **HRMS** (ESI) m/z calculated for $[M+Na]^+$ 258.1106 found 258.1105. **ATR-FTIR** (cm^{-1}): 2934, 1642, 1601, 1583, 1487, 1455, 1434, 1400, 1318, 1285, 1257, 1218, 1184, 1157, 1124, 1035, 958, 929, 861, 812, 782, 750, 725, 696, 628, 567, 555, 534.

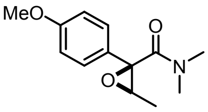
(2*R**,3*R**)-2-(4-methoxyphenyl)-*N*,*N*,3-trimethyloxirane-2-carboxamide (**10j**)

Synthesized following the procedure for the epoxidation (General procedure D) in DCM with aqueous $NaHCO_3$ as the acid quencher. Epoxidation at room temperature. 61% ≙ 29 mg. Yellow oil. Decomposes at room temperature.
^{1}H NMR (400 MHz, $CDCl_3$) δ 7.33 (d, *J* = 8.7 Hz, 2H), 6.86 (d, *J* = 8.7 Hz, 2H), 3.79 (s, 3H), 3.13 (q, *J* = 5.4 Hz, 1H), 2.99 – 2.97 (m, 6H), 1.42 (d, *J* = 5.4 Hz, 3H). **^{13}C NMR** (176 MHz, $CDCl_3$) δ 167.34, 159.78, 128.22, 126.52, 114.16, 64.40, 64.16, 55.42, 37.02, 35.20, 16.77.. **HRMS** (ESI) m/z calculated for $[M+Na]^+$ 258.1106 found 258.1104. **ATR-FTIR** (cm^{-1}): 2933, 1640, 1612, 1580, 1511, 1460, 1400, 1303, 1275, 1247, 1169, 1125, 1030, 981, 912, 829, 793, 750, 729, 675, 646, 591, 573.

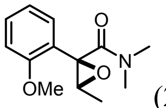
(2*R**,3*R**)-2-(2-methoxyphenyl)-*N*,*N*,3-trimethyloxirane-2-carboxamide (**10k**)

Synthesized following the procedure for the epoxidation (General procedure D) in DCM with aqueous $NaHCO_3$ as the acid quencher. Epoxidation at room temperature. 70% ≙ 33 mg. Yellow oil. Decomposes at room temperature.
^{1}H NMR (600 MHz, $CDCl_3$) δ 7.43 (dd, *J* = 7.6, 1.6 Hz, 1H), 7.31 – 7.27 (m, 1H), 6.96 – 6.91 (m, 1H), 6.87 (d, *J* = 8.3 Hz, 1H), 3.83 (s, 3H), 3.54 (q, *J* = 5.4 Hz, 1H), 3.16 (s, 3H), 2.93 (s, 3H), 1.42 (d, *J* = 5.4 Hz, 3H). **^{13}C NMR** (151 MHz, $CDCl_3$) δ 168.10, 157.85, 130.19, 130.12, 125.15, 120.93, 111.36, 63.07, 60.02, 55.72, 37.08, 35.84, 15.47. **HRMS** (ESI) m/z calculated for $[M+Na]^+$ 258.1106 found 258.1102. **ATR-FTIR** (cm^{-1}): 2936, 2361, 2243, 2335, 1639, 1602, 1585, 1495, 1461, 1438, 1398, 1253, 1168, 1117, 1055, 1036, 1025, 982, 917, 756, 679.

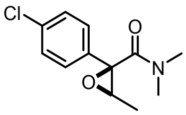
(2*R**,3*R**)-2-(4-chlorophenyl)-*N*,*N*,3-trimethyloxirane-2-carboxamide (**10l**)

Synthesized following the procedure for the epoxidation (General procedure D) in 1,2-DCE with water as the acid quencher. Epoxidation at 40 °C. >95% ≙ 46 mg. Yellow oil.
^{1}H NMR (600 MHz, $CDCl_3$) δ 7.40 – 7.37 (m, 2H), 7.33 – 7.29 (m, 2H), 3.09 (q, *J* = 5.3 Hz, 1H), 2.98 – 2.97 (m, 6H), 1.43 (d, *J* = 5.3 Hz, 3H). **^{13}C NMR** (151 MHz, $CDCl_3$) δ 166.72, 134.94, 134.36, 128.97, 126.63, 64.64, 64.12, 36.98, 35.28, 16.82. **HRMS** (ESI) m/z calculated for $[M+Na]^+$ 262.0611 found 262.0608. **ATR-FTIR** (cm^{-1}): 2360, 2343, 2335, 1648, 1492, 1461, 1420, 1399, 1126, 1091, 1040, 1015, 984, 918, 824, 750, 661.

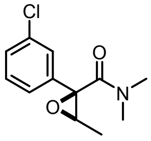

(2*R**,3*R**)-2-(3-chlorophenyl)-*N,N*,3-trimethyloxirane-2-carboxamide (**10m**)

Synthesized following the procedure for the epoxidation (General procedure D) in 1,2-DCE with water as the acid quencher. Epoxidation at 40 °C. 74% ≙ 35 mg. Yellow oil.
^{1}H NMR (400 MHz, $CDCl_3$) δ 7.39 – 7.36 (m, 1H), 7.31 – 7.26 (m, 1H), 7.23 – 7.17 (m, 2H), 3.03 (q, *J* = 5.3 Hz, 1H), 2.92 (s, 3H), 2.92 (s, 3H), 1.37 (d, *J* = 5.3 Hz, 3H). **^{13}C NMR** (151 MHz, $CDCl_3$) δ 166.52, 138.54, 134.93, 130.07, 128.63, 125.24, 123.47, 64.72, 63.99, 37.00, 35.30, 16.81. **HRMS** (ESI) m/z calculated for $[M+Na]^+$ 262.0611 found 262.0609. **ATR-FTIR** (cm^{-1}): 2930, 1642, 1597, 1572, 1503, 1474, 1400, 1244, 1172, 1126, 1079, 1043, 985, 931, 891, 864, 786, 749, 706, 691, 674, 629.

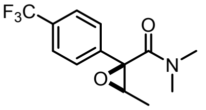

(2*R**,3*R**)-*N,N*,3-trimethyl-2-(4-(trifluoromethyl)phenyl)oxirane-2-carboxamide (**10n**)

Synthesized following the procedure for the epoxidation (General procedure D) in 1,2-DCE with water as the acid quencher. Epoxidation at 60 °C. 66% ≙ 36 mg. Colorless oil.
^{1}H NMR (600 MHz, $CDCl_3$) δ 7.63 – 7.53 (m, 4H), 3.10 (q, *J* = 5.3 Hz, 1H), 2.99 – 2.97 (m, 6H), 1.44 (d, *J* = 5.3 Hz, 3H). **^{13}C NMR** (151 MHz, $CDCl_3$) δ 166.43, 140.45, 130.61 (q, *J* = 32.5 Hz), 125.74 (dd, *J* = 7.2 Hz, 3.5 Hz) 125.59, 124.09 (q, *J* = 272.3 Hz), 64.87, 64.12, 36.94, 35.30, 16.82. **HRMS** (ESI) m/z calculated for $[M+Na]^+$ 296.0874 found 296.0873. **ATR-FTIR** (cm^{-1}): 2934, 1644, 1504, 1456, 1403, 1323, 1274, 1164, 1121, 1109, 1067, 1039, 1017, 986, 959, 921, 865, 834, 794, 764, 750, 727, 690, 649, 606, 574.

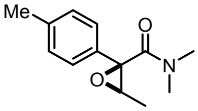

(2*R**,3*R**)-*N,N*,3-trimethyl-2-(*p*-tolyl)oxirane-2-carboxamide (**10o**)

Synthesized following the procedure for the epoxidation (General procedure D) in DCM with aqueous $NaHCO_3$ as the acid quencher. Epoxidation at room temperature. 68% ≙ 30 mg. Yellow oil. Decomposes at room temperature.
^{1}H NMR (400 MHz, $CDCl_3$) δ 7.31 (d, *J* = 8.2 Hz, 2H), 7.15 (d, *J* = 8.2 Hz, 2H), 3.13 (q, *J* = 5.4 Hz, 1H), 2.98 (s, 3H), 2.97 – 2.33 (m, 6H), 1.43 (d, *J* = 5.4 Hz, 3H). **^{13}C NMR** (101 MHz, $CDCl_3$) δ 167.26, 138.21, 133.30, 129.44, 125.10, 64.53, 64.31, 37.01, 35.19, 21.23, 16.81. **HRMS** (ESI) m/z calculated for $[M+Na]^+$ 242.1157 found 242.1153 **ATR-FTIR** (cm^{-1}): 2928, 2873, 2363, 2341, 1642, 1511, 1450, 1419, 1399, 1268, 1251, 1171, 1124, 1040, 1022, 981, 954, 917, 862, 815, 728, 712, 675, 591.

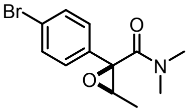

(2*R**,3*R**)-2-(4-bromophenyl)-*N,N*,3-trimethyloxirane-2-carboxamide (**10p**)

Synthesized following the procedure for the epoxidation (General procedure D) in 1,2-DCE with water as the acid quencher. Epoxidation at 40 °C. 81% ≙ 46 mg. Yellow oil.
^{1}H NMR (400 MHz, $CDCl_3$) δ 7.47 – 7.43 (m, 2H), 7.34 – 7.28 (m, 2H), 3.07 (q, *J* = 5.4 Hz, 1H), 2.96 – 2.95 (m, 6H), 1.41 (d, *J* = 5.4 Hz, 3H). **^{13}C NMR** (101 MHz, $CDCl_3$) δ 166.65, 135.46, 131.87, 126.92, 122.47, 64.54, 64.11, 36.93, 35.25, 16.76. **HRMS** (ESI) m/z calculated for $[M+Na]^+$ 306.0106 found 306.0101. **ATR-FTIR** (cm^{-1}): 2933, 1644, 1488, 1397, 1262, 1125, 1072, 1040, 1011, 984, 918, 821, 757.

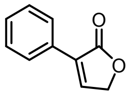 3-phenylfuran-2(5H)-one (**11a**)

Synthesized following the procedure for the 2-furanone synthesis (General procedure E). 46% ≙ 15 mg. Colorless oil. Unreacted alkene intermediate (7a) can be recovered in similar quantities.
Spectroscopical data of 11a is in accordance with reported characterization: *Org. Lett.*, **2019**, *21*, 2231–2235.

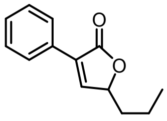 3-phenyl-5-propylfuran-2(5H)-one (**11g**)

Synthesized following the procedure for the 2-furanone synthesis (General procedure E). 71% ≙ 29 mg. Colorless oil.
^{1}H NMR (400 MHz, $CDCl_3$) δ 7.90 – 7.78 (m, 2H), 7.55 (d, J = 1.8 Hz, 1H), 7.47 – 7.30 (m, 3H), 5.05 (m, 1H), 1.89 – 1.65 (m, 2H), 1.61 – 1.40 (m, 2H), 1.00 (t, J = 7.4 Hz, 3H). **^{13}C NMR** (151 MHz, $CDCl_3$) δ 171.88, 148.13, 131.68, 129.74, 129.39, 128.76, 127.15, 80.48, 35.72, 18.62, 13.98. **HRMS** (ESI) m/z calculated for $[M+Na]^+$ 225.0891 found 225.0892. **ATR-FTIR** (cm^{-1}): 3072, 2961, 2934, 2874, 2349, 1745, 1689, 1598, 1493, 1449, 1381, 1328, 1304, 1246, 1183, 1117, 1069, 1028, 965, 937, 901, 865.

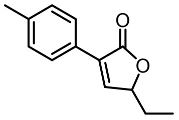 Incrustoporine (**11q**)

Synthesized following the procedure for the 2-furanone synthesis (General procedure E). 69% ≙ 28 mg. Colorless oil.
Spectroscopical data of **11q** is in accordance with reported characterization: *Eur. J. Org. Chem.* **2015**, 2012–2022.

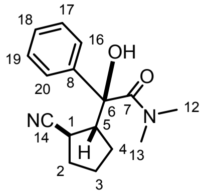

(*S**)-2-((1*R**,2*R**)-2-cyanocyclopentyl)-2-hydroxy-N,N-dimethyl-2-phenylacetamide (**22**)

Synthesized following the procedure for the epoxynitrile cyclization. 66% ≙ 36 mg. Colorless oil.
^{1}H NMR (700 MHz, $CDCl_3$) δ 7.41 – 7.32 (m, 4H), 7.31 – 7.28 (m, 1H), 5.95 (s, 1H), 3.40 – 3.33 (m, 1H), 3.12 – 2.66 (m, 7H), 2.26 – 2.17 (m, 1H), 2.15 – 2.09 (m, 1H), 2.08 – 2.03 (m, 1H), 1.90 – 1.78 (m, 2H), 1.72 – 1.62 (m, 1H). **^{13}C NMR** (176 MHz, $CDCl_3$) δ 173.21 (C7), 141.86 (C8), 128.80 (C17/19), 128.06 (C18), 127.18 (C20/16), 122.72 (C14), 78.46 (C6), 47.24 (C5), 37.82 (C13/12), 33.07 (C4), 29.46 (C1), 29.12 (C2), 26.61 (C3). **HRMS** (ESI) m/z calculated for $[M+Na]^+$ 295.1422 found 295.1413. **ATR-FTIR** (cm^{-1}): 3345, 2957, 2872, 2236, 1632, 1495, 1449, 1368, 1256, 1145, 1070, 1036, 964, 915.
.

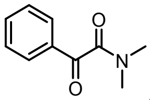 *N,N*-dimethyl-2-oxo-2-phenylacetamide (**23a**)

Synthesized following the procedure for the syntheses of the α–ketoamide. Colorless oil (65% ≙ 23 mg).

Spectroscopical data of 15a is in accordance with reported characterization: *Chemical Communications* **2012**, *48,* 10117–10119. **^{1}H NMR** (400 MHz, $CDCl_3$) δ 7.97 – 7.92 (m, 2H), 7.67 – 7.61 (m, 1H), 7.55 – 7.48 (m, 2H), 3.12 (s, 3H), 2.96 (s, 3H).

VII. Spectra

Compound **6a**

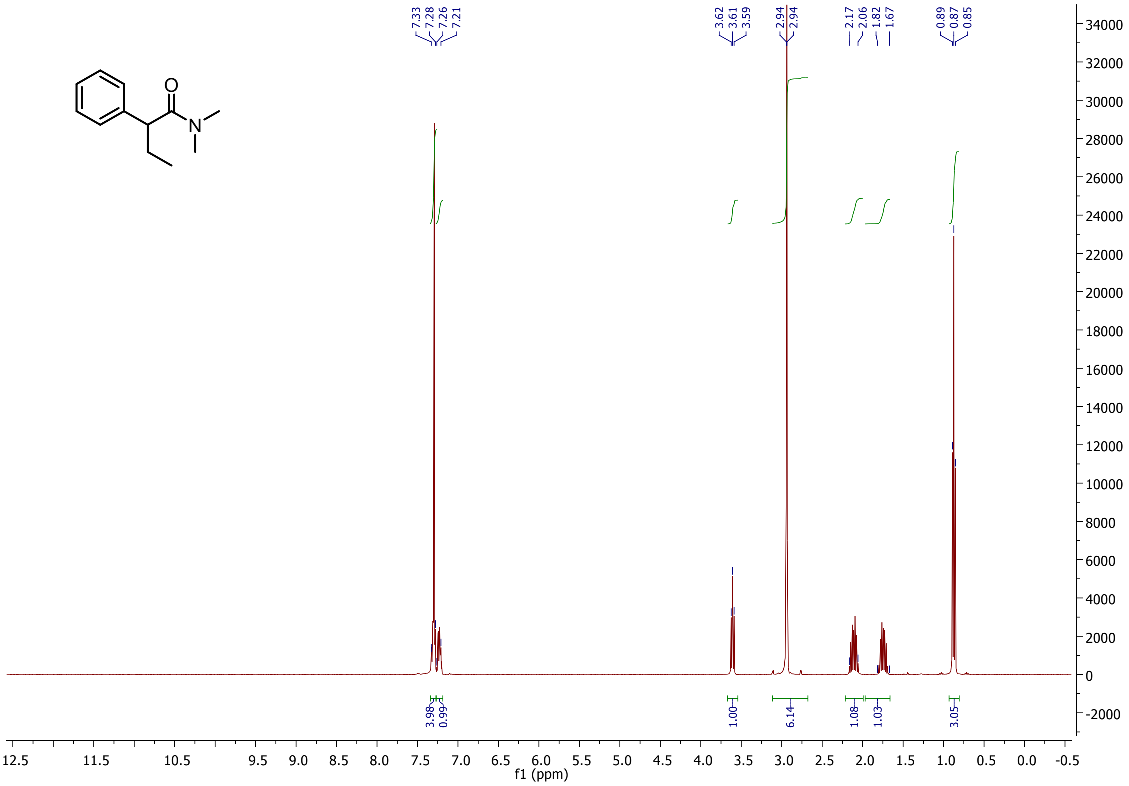

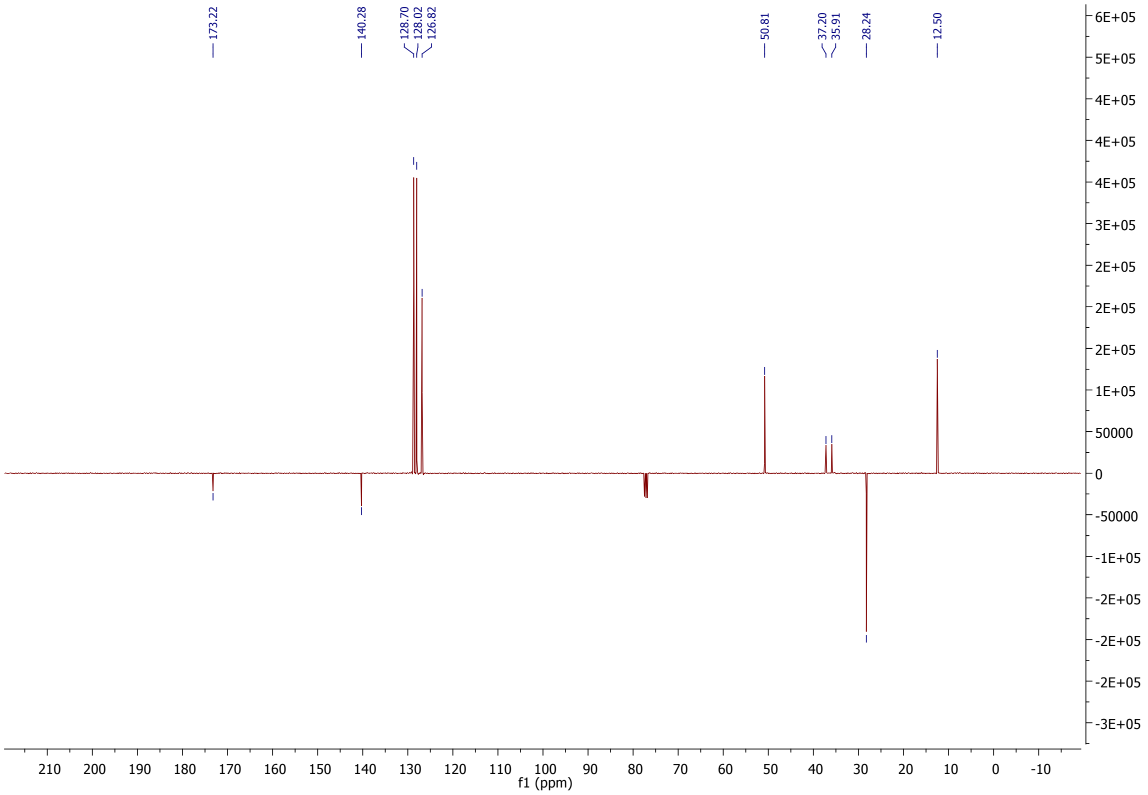
173.22
140.28
128.70
128.02
126.82
50.81
37.20
35.91
28.24
12.50
6E+05
5E+05
4E+05
4E+05
4E+05
3E+05
2E+05
2E+05
2E+05
1E+05
50000
0
-50000
-1E+05
-2E+05
-2E+05
-2E+05
-3E+05
210 200 190 180 170 160 150 140 130 120 110 100 90 80 70 60 50 40 30 20 10 0 -10
f1 (ppm)

Compound **6b**

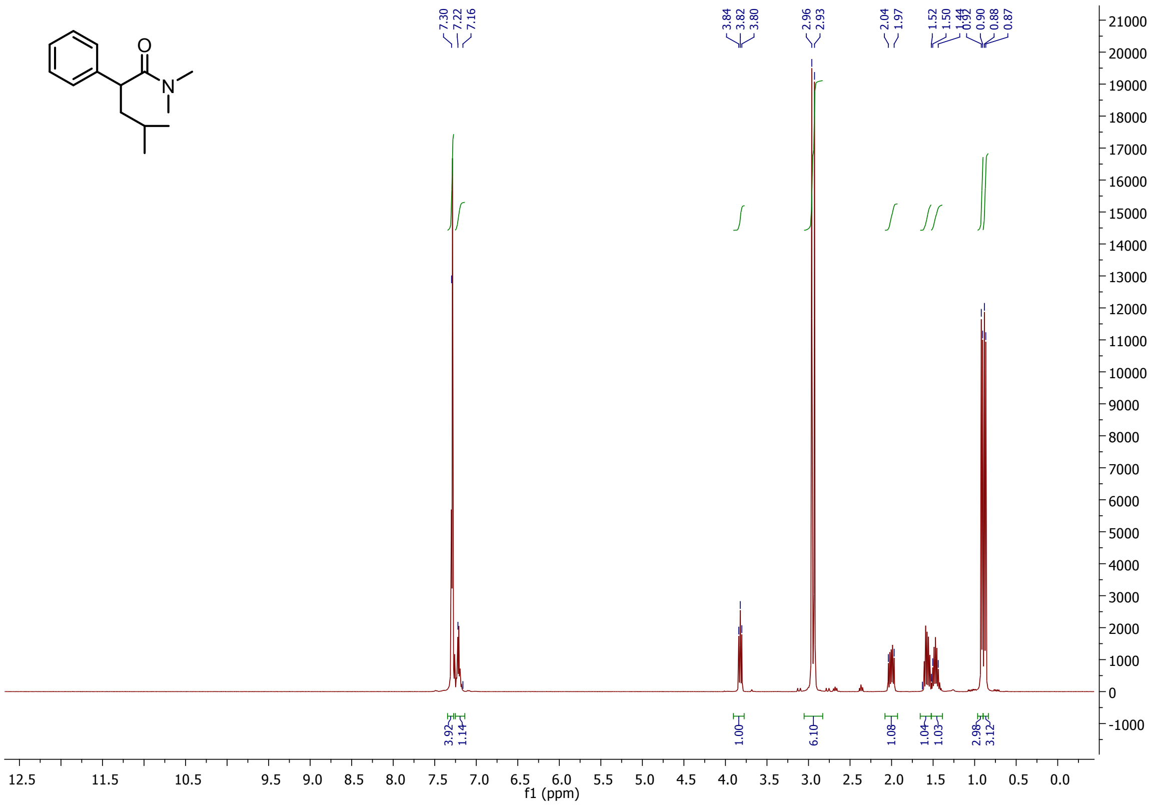
O
N
7.30
7.22
7.16
3.84
3.82
3.80
2.96
2.93
2.04
1.97
1.52
1.50
1.44
0.92
0.90
0.88
0.87
3.92
1.14
1.00
6.10
1.08
1.04
1.03
2.98
3.12
f1 (ppm)

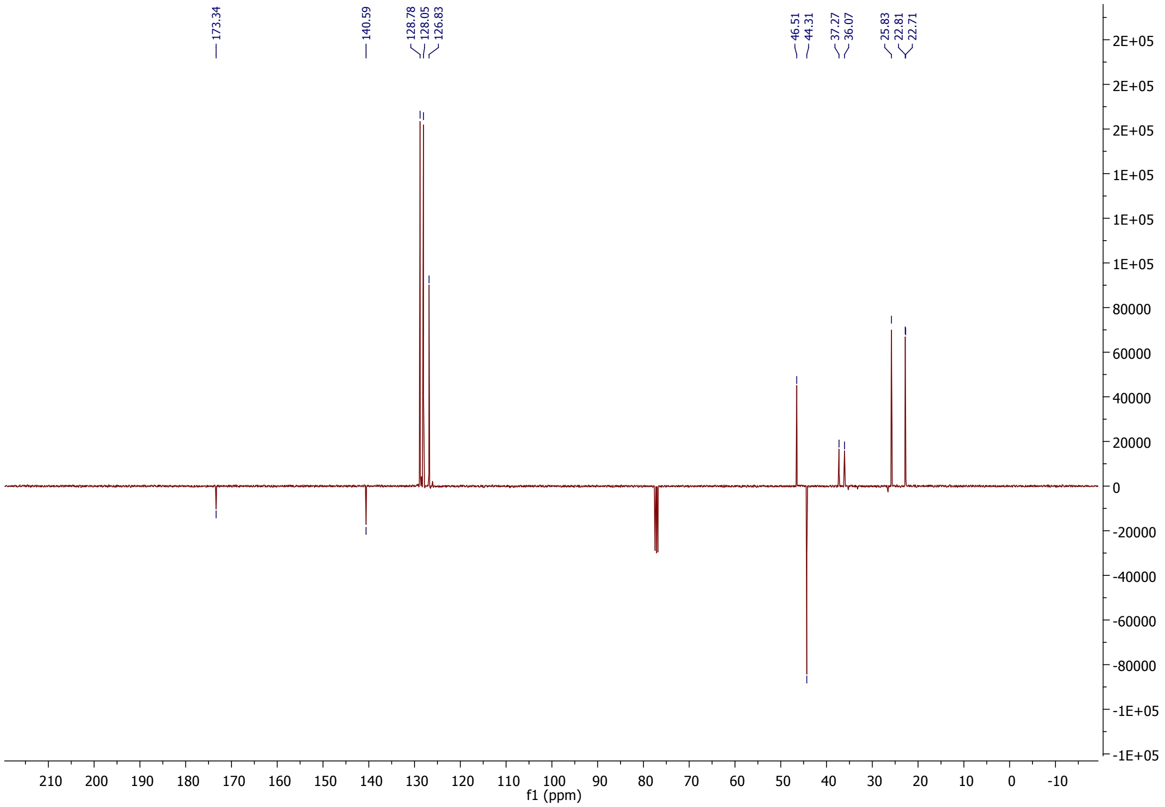
173.34
140.59
128.78
128.05
126.83
46.51
44.31
37.27
36.07
25.83
22.81
22.71
f1 (ppm)

Compound **6c**

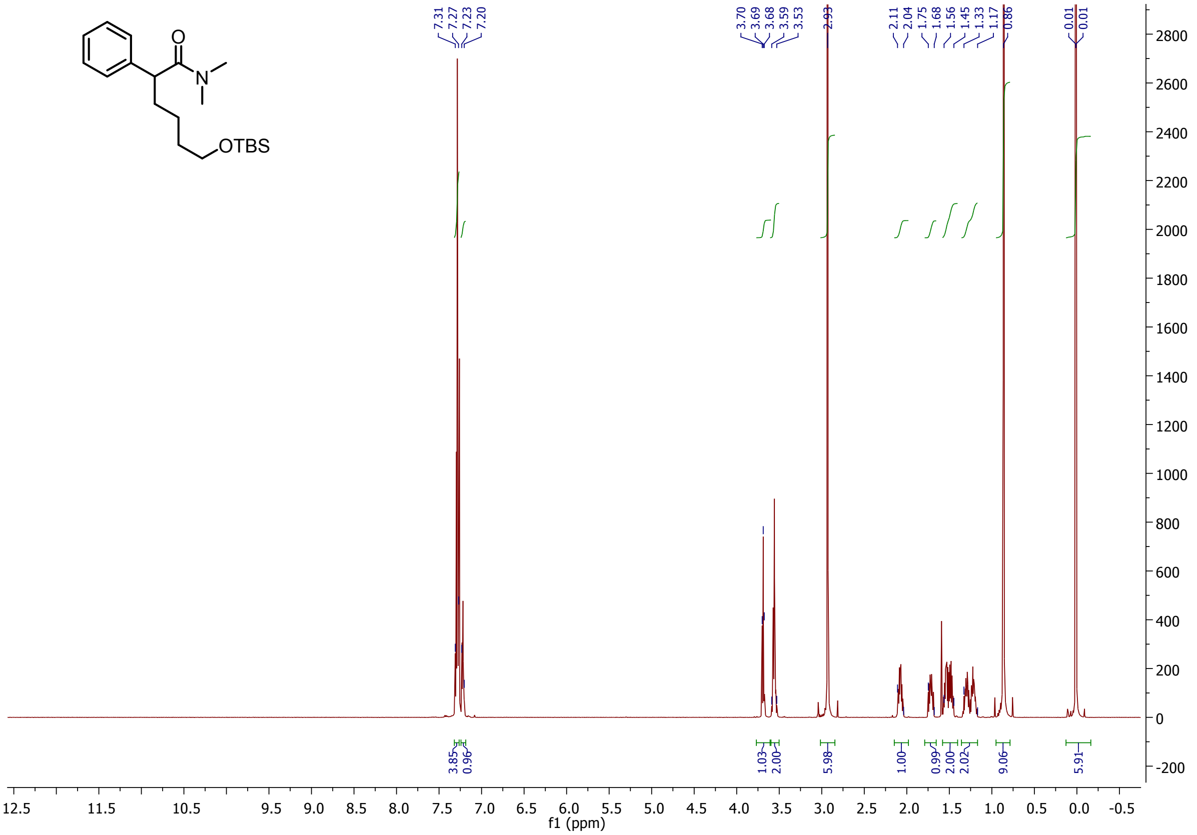
O
N
OTBS
7.31
7.27
7.23
7.20
3.70
3.69
3.68
3.59
3.53
2.93
2.11
2.04
1.75
1.68
1.56
1.45
1.33
1.17
0.86
0.01
0.01
3.85
0.96
1.03
2.00
5.98
1.00
0.99
2.00
2.02
9.06
5.91
f1 (ppm)

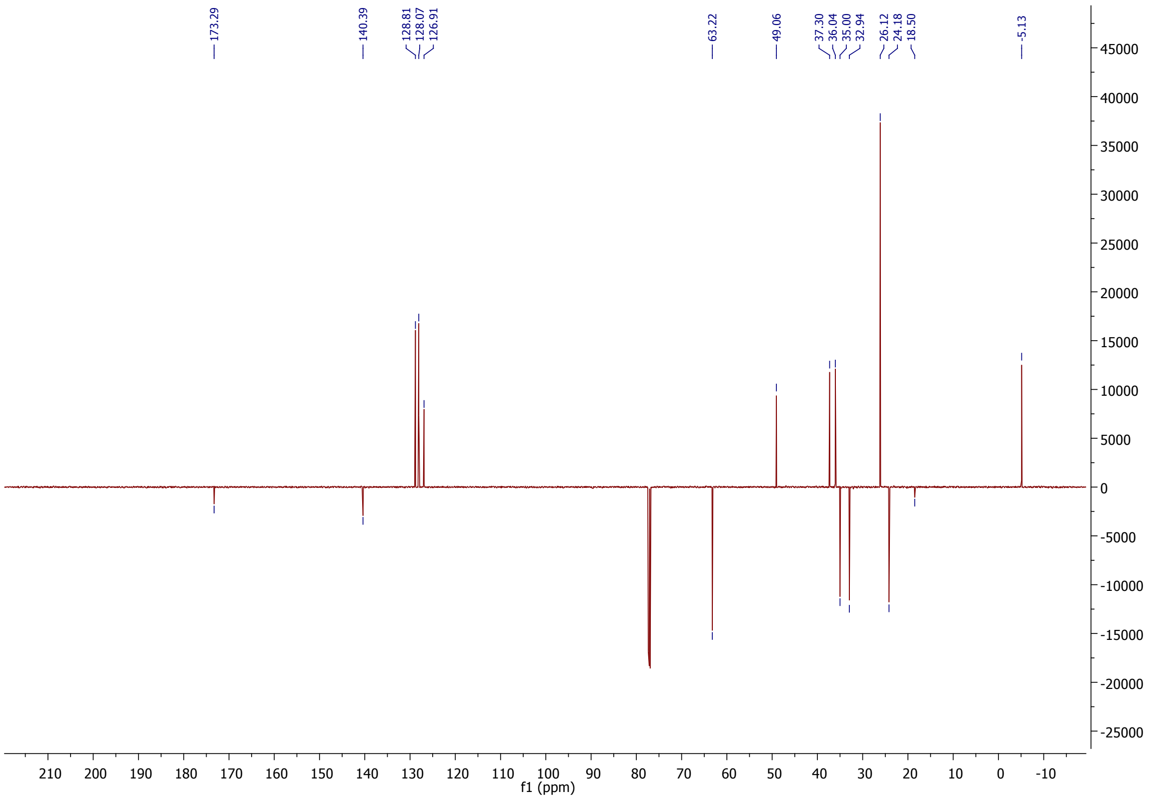
173.29
140.39
128.81
128.07
126.91
63.22
49.06
37.30
36.04
35.00
32.94
26.12
24.18
18.50
-5.13
45000
40000
35000
30000
25000
20000
15000
10000
5000
0
-5000
-10000
-15000
-20000
-25000
210 200 190 180 170 160 150 140 130 120 110 100 90 80 70 60 50 40 30 20 10 0 -10
f1 (ppm)

Compound **6d**

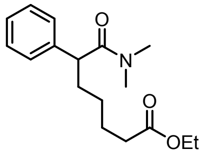

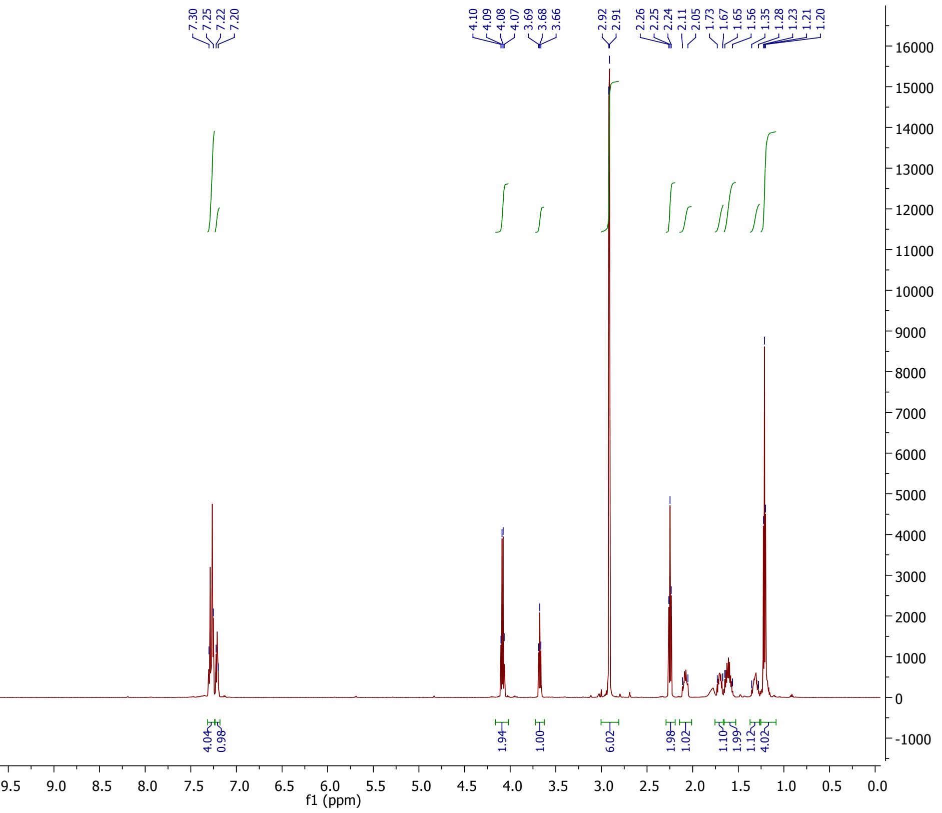
7.30
7.25
7.22
7.20
4.10
4.09
4.08
4.07
3.69
3.68
3.66
2.92
2.91
2.26
2.25
2.24
2.11
2.05
1.73
1.67
1.65
1.56
1.35
1.28
1.23
1.21
1.20
4.04
0.98
1.94
1.00
6.02
1.98
1.02
1.10
1.99
1.12
4.02
f1 (ppm)

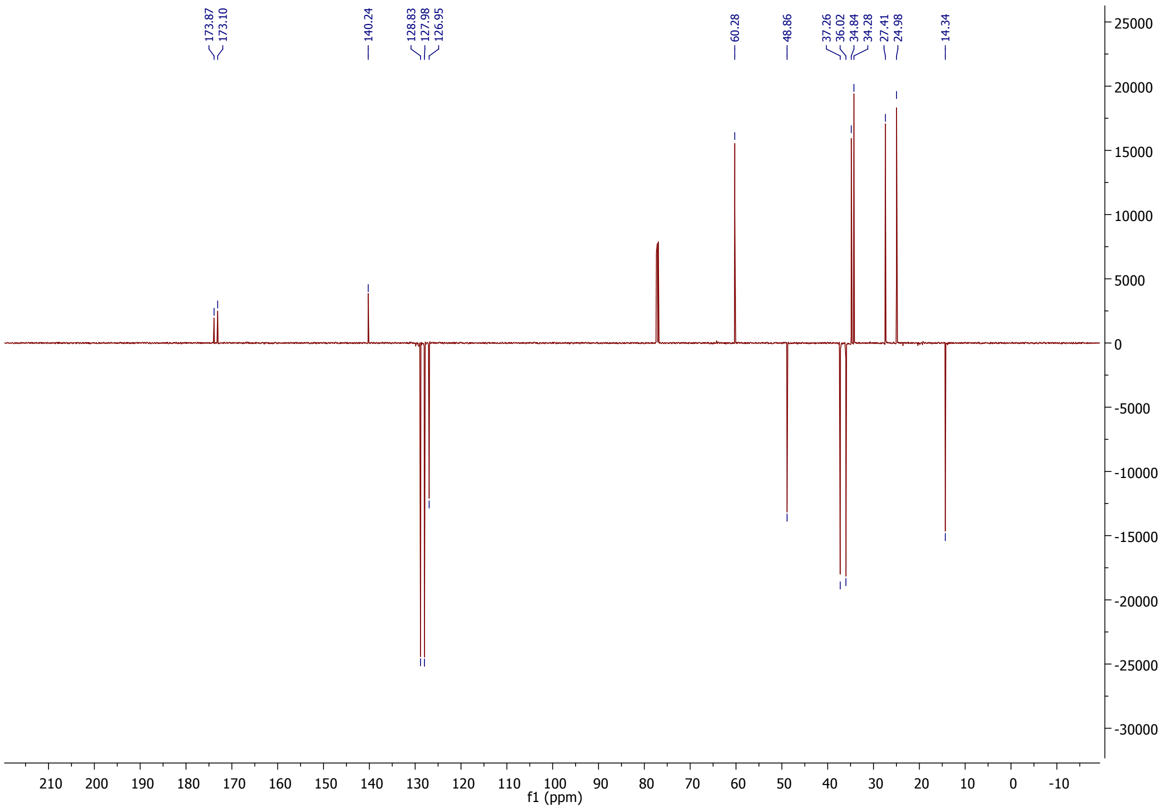
173.87
173.10
140.24
128.83
127.98
126.95
60.28
48.86
37.26
36.02
34.84
34.28
27.41
24.98
14.34
25000
20000
15000
10000
5000
0
-5000
-10000
-15000
-20000
-25000
-30000
210 200 190 180 170 160 150 140 130 120 110 100 90 80 70 60 50 40 30 20 10 0 -10
f1 (ppm)

Compound **6e**

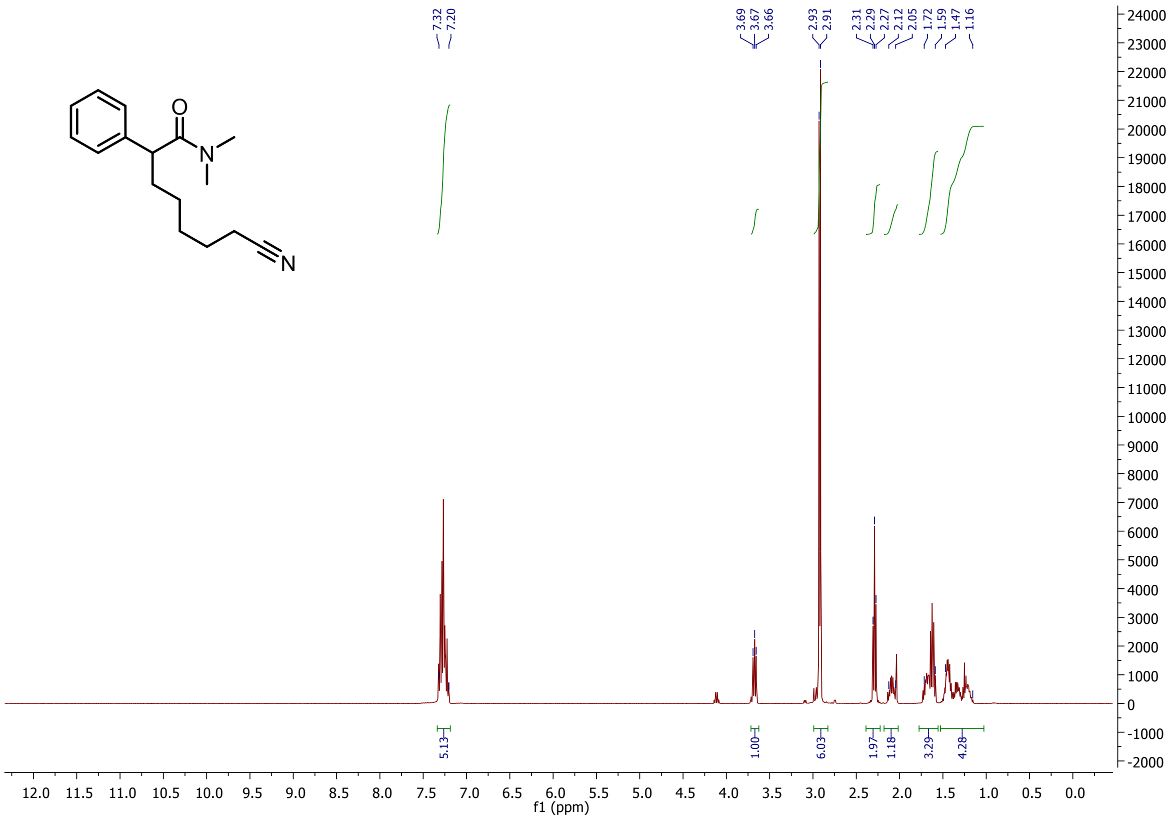
7.32
7.20
3.69
3.67
3.66
2.93
2.91
2.31
2.29
2.27
2.12
2.05
1.72
1.59
1.47
1.16
5.13
1.00
6.03
1.97
1.18
3.29
4.28
f1 (ppm)

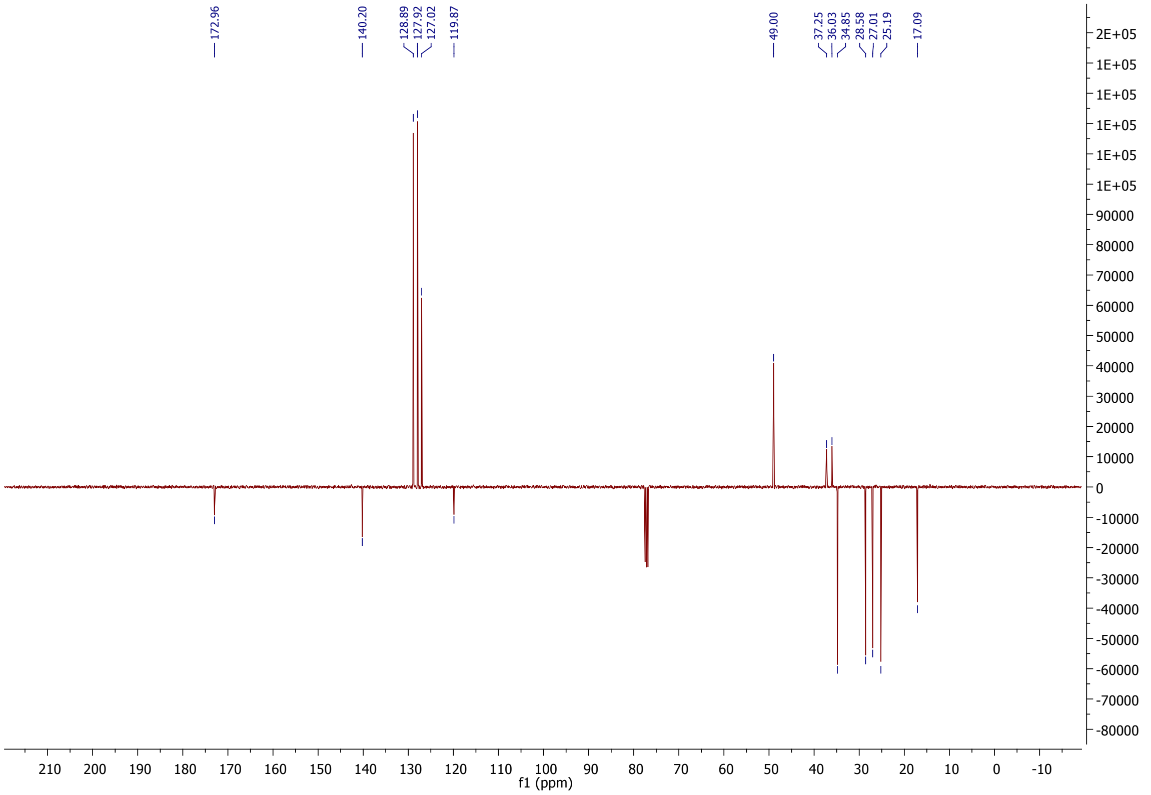
172.96
140.20
128.89
127.92
127.02
119.87
49.00
37.25
36.03
34.85
28.58
27.01
25.19
17.09
f1 (ppm)

Compound **6f**

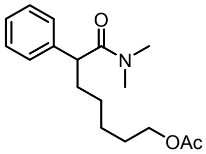
O
N
OAc

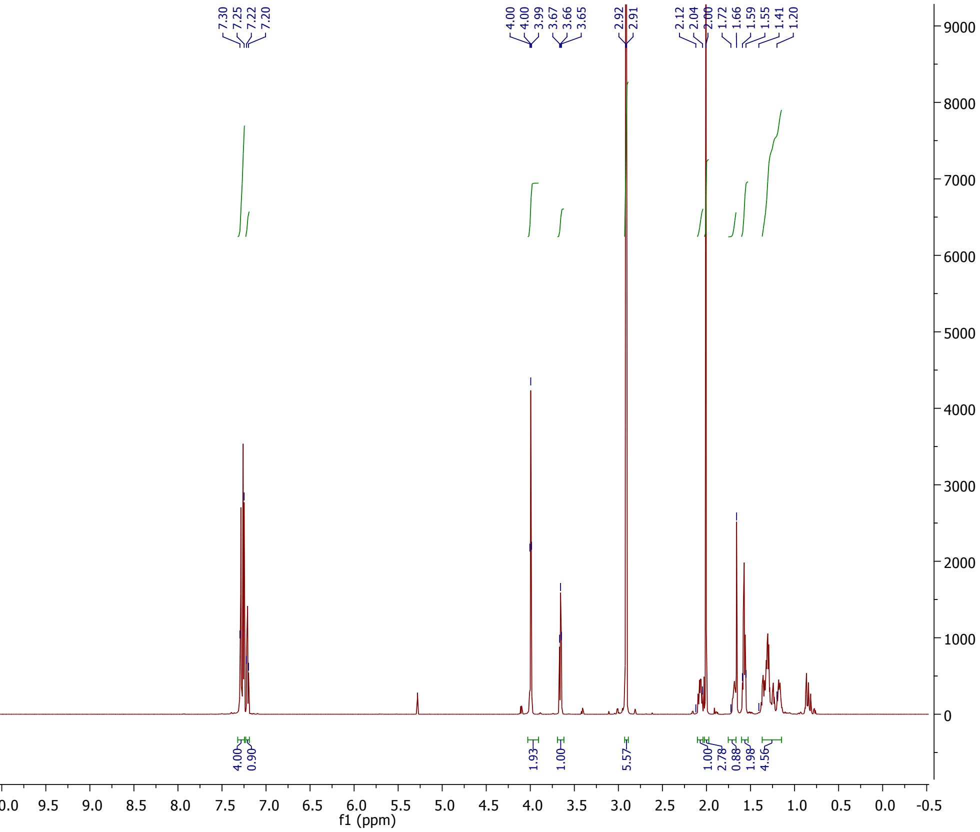
7.30
7.25
7.22
7.20
4.00
4.00
3.99
3.67
3.66
3.65
2.92
2.91
2.12
2.04
2.00
1.72
1.66
1.59
1.55
1.41
1.20
4.00
0.90
1.93
1.00
5.57
1.00
2.78
0.88
1.98
4.56
f1 (ppm)

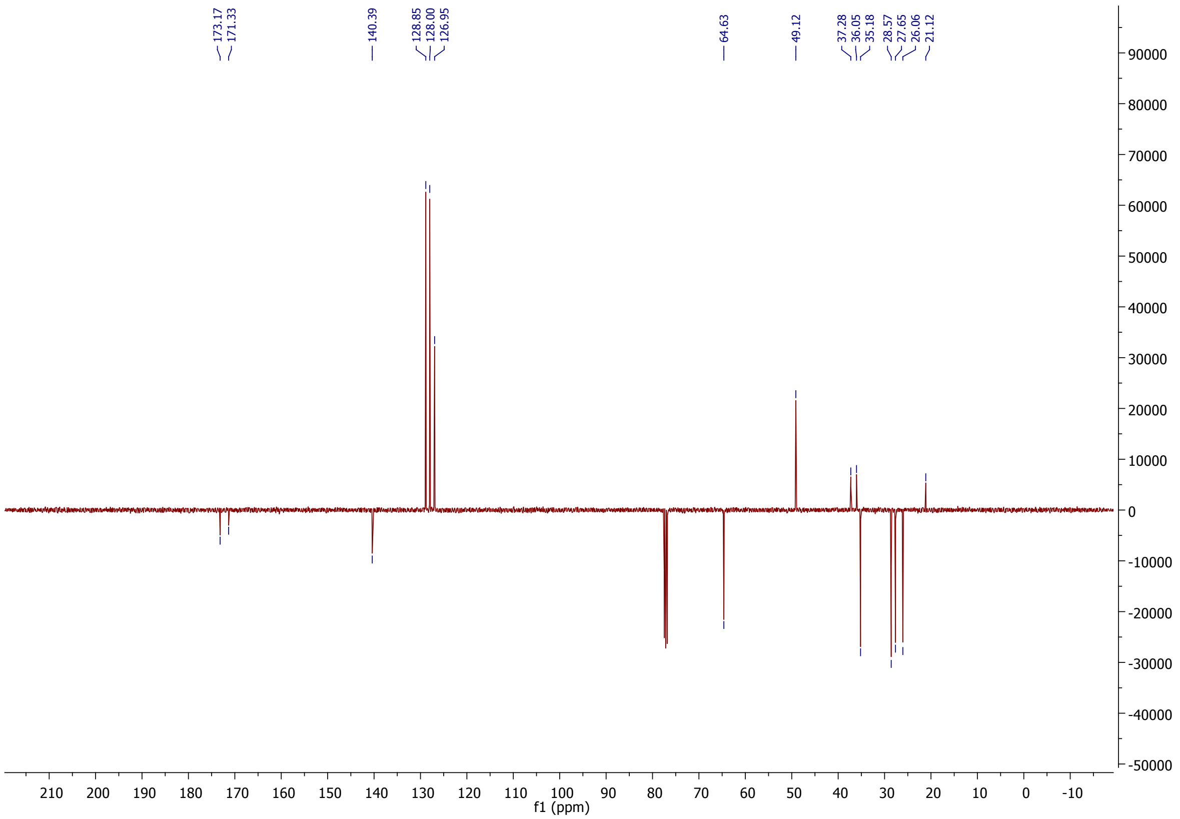
173.17
171.33
140.39
128.85
128.00
126.95
64.63
49.12
37.28
36.05
35.18
28.57
27.65
26.06
21.12
90000
80000
70000
60000
50000
40000
30000
20000
10000
0
-10000
-20000
-30000
-40000
-50000
210 200 190 180 170 160 150 140 130 120 110 100 90 80 70 60 50 40 30 20 10 0 -10
f1 (ppm)

Compound **6g**

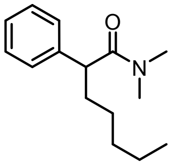

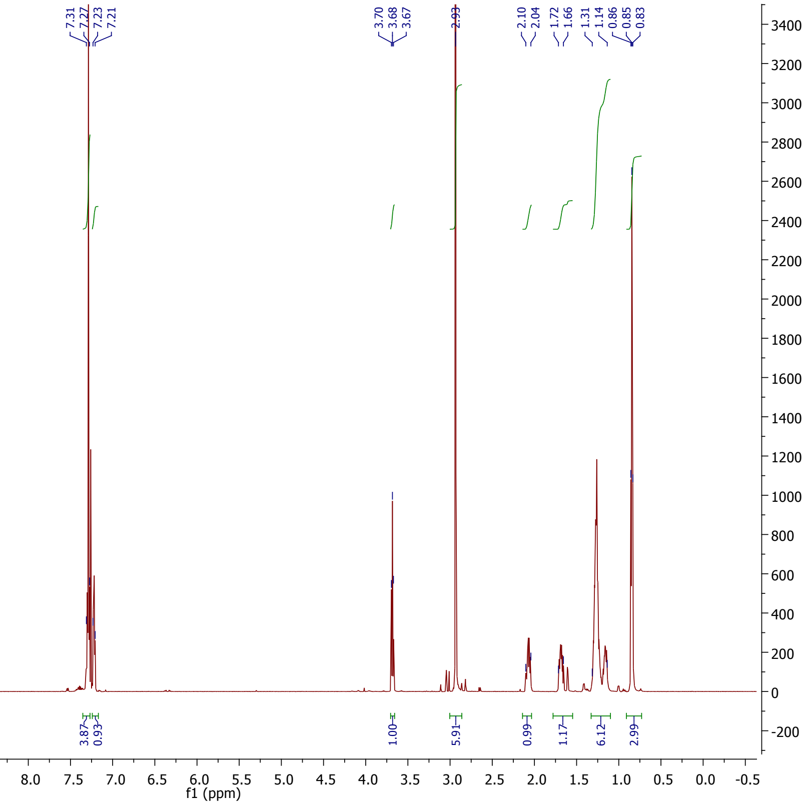
7.31
7.22
7.23
7.21
3.70
3.68
3.67
2.93
2.10
2.04
1.72
1.66
1.31
1.14
0.86
0.85
0.83
3.87
0.93
1.00
5.91
0.99
1.17
6.12
2.99
f1 (ppm)

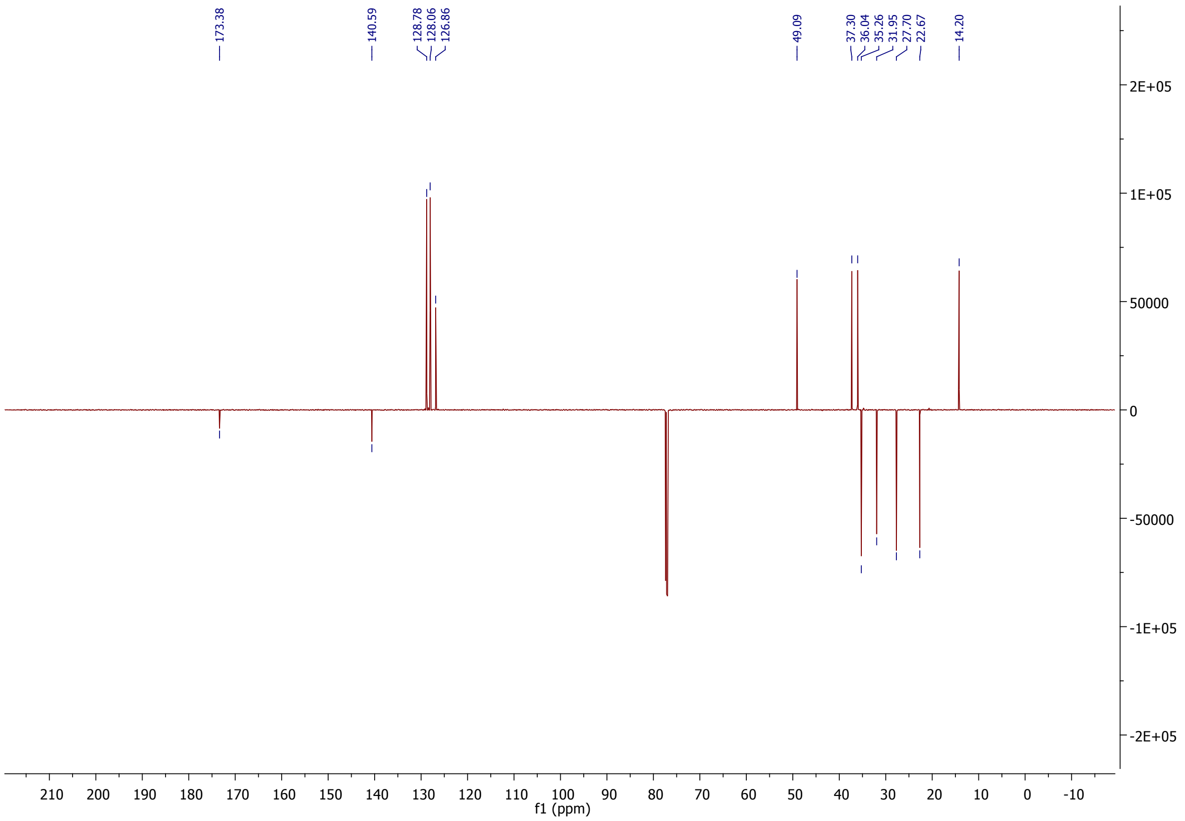

173.38
140.59
128.78
128.06
126.86
49.09
37.30
36.04
35.26
31.95
27.70
22.67
14.20
2E+05
1E+05
50000
0
-50000
-1E+05
-2E+05
210 200 190 180 170 160 150 140 130 120 110 100 90 80 70 60 50 40 30 20 10 0 -10
f1 (ppm)

Compound **6h**

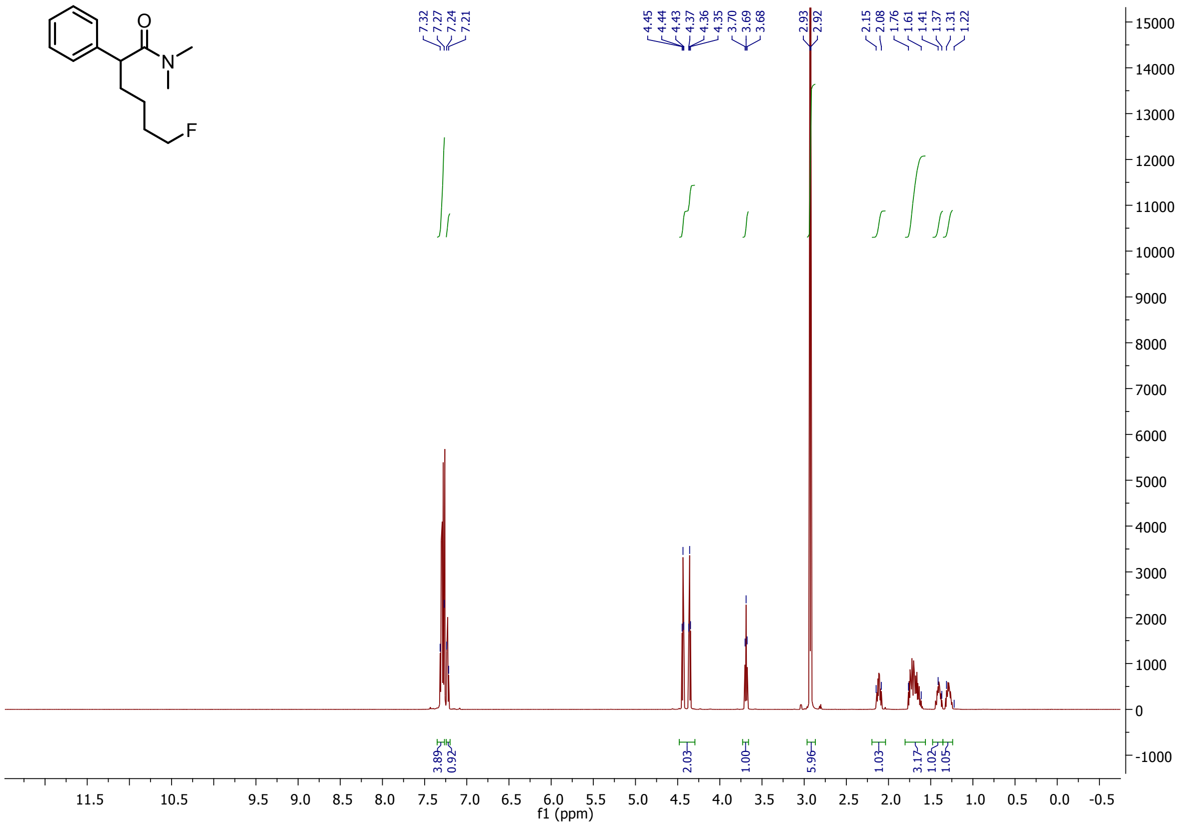
O
N
F
7.32
7.27
7.24
7.21
4.45
4.44
4.43
4.37
4.36
4.35
3.70
3.69
3.68
2.93
2.92
2.15
2.08
1.76
1.61
1.41
1.37
1.31
1.22
3.89
0.92
2.03
1.00
5.96
1.03
3.17
1.02
1.05
f1 (ppm)

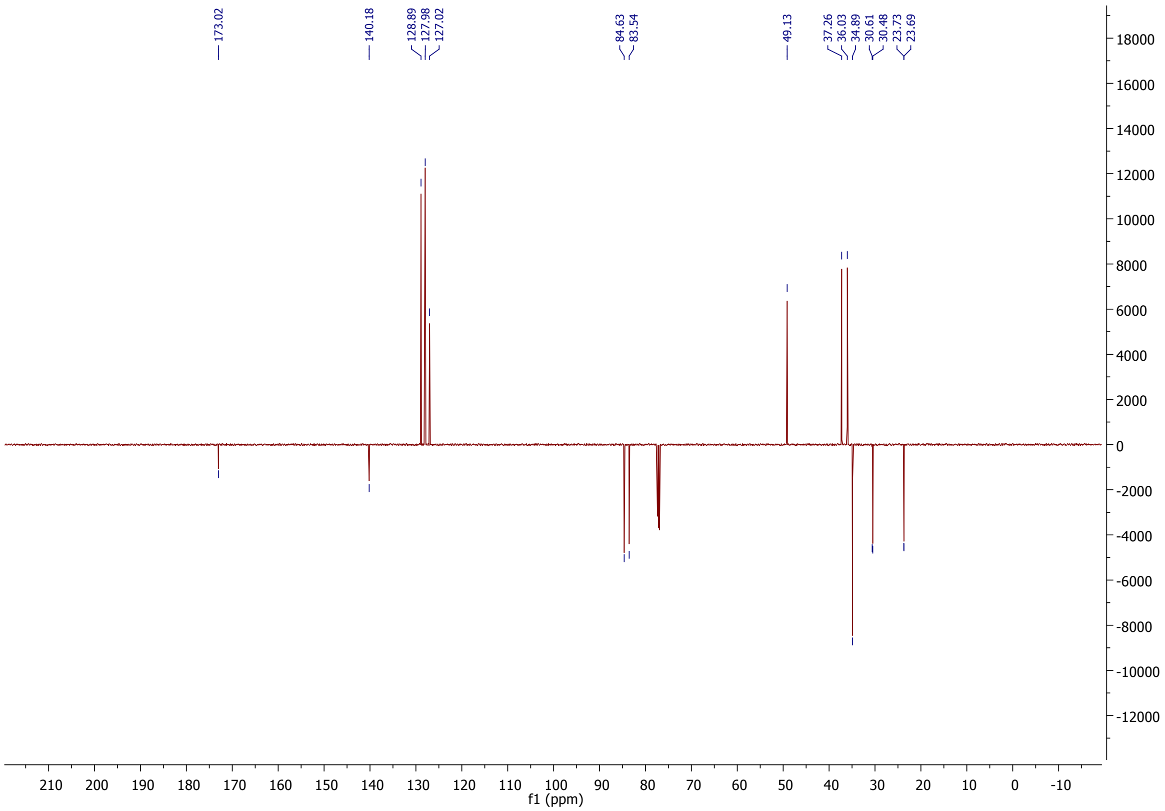
173.02
140.18
128.89
127.98
127.02
84.63
83.54
49.13
37.26
36.03
34.89
30.61
30.48
23.73
23.69
f1 (ppm)

Compound **6i**

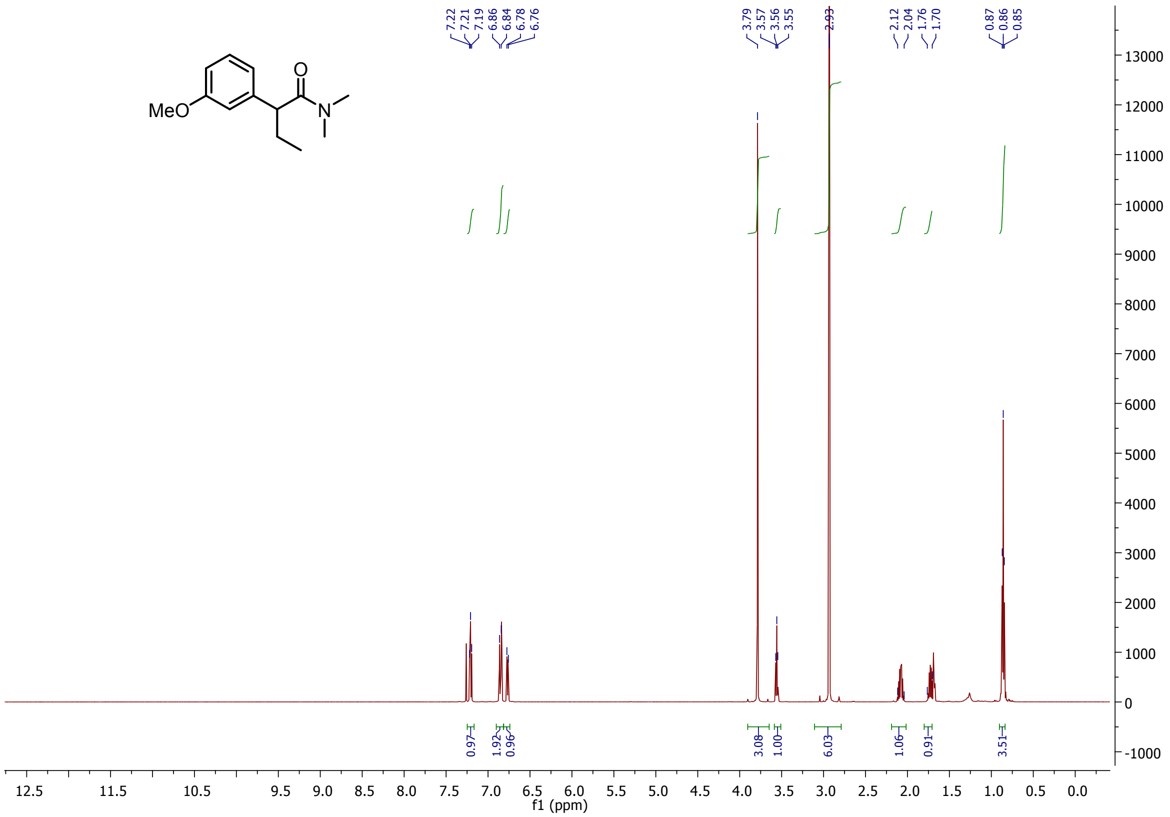

MeO
O
N
7.22
7.21
7.19
6.86
6.84
6.78
6.76
3.79
3.57
3.56
3.55
2.93
2.12
2.04
1.76
1.70
0.87
0.86
0.85
0.97
1.92
0.96
3.08
1.00
6.03
1.06
0.91
3.51
13000
12000
11000
10000
9000
8000
7000
6000
5000
4000
3000
2000
1000
0
-1000
12.5
11.5
10.5
9.5
9.0
8.5
8.0
7.5
7.0
6.5
6.0
5.5
5.0
4.5
4.0
3.5
3.0
2.5
2.0
1.5
1.0
0.5
0.0
f1 (ppm)

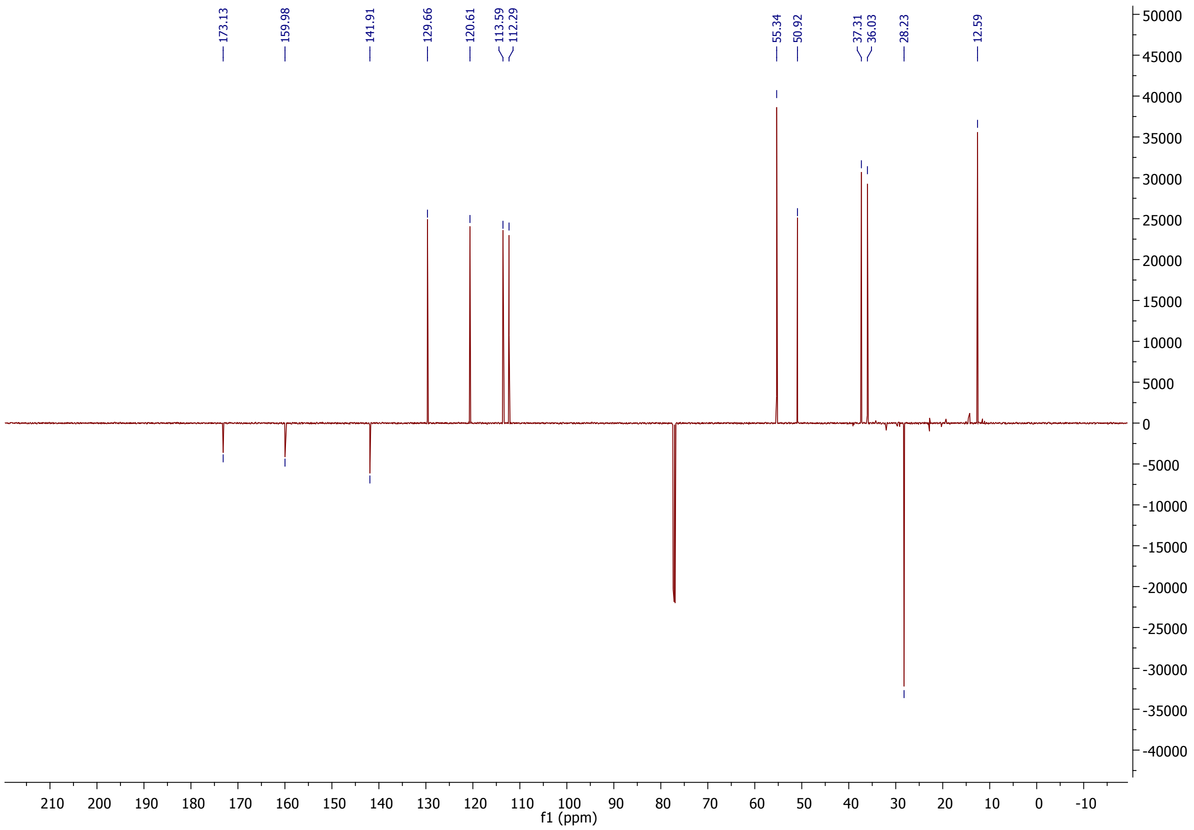
173.13
159.98
141.91
129.66
120.61
113.59
112.29
55.34
50.92
37.31
36.03
28.23
12.59
50000
45000
40000
35000
30000
25000
20000
15000
10000
5000
0
-5000
-10000
-15000
-20000
-25000
-30000
-35000
-40000
210 200 190 180 170 160 150 140 130 120 110 100 90 80 70 60 50 40 30 20 10 0 -10
f1 (ppm)

Compound **6j**

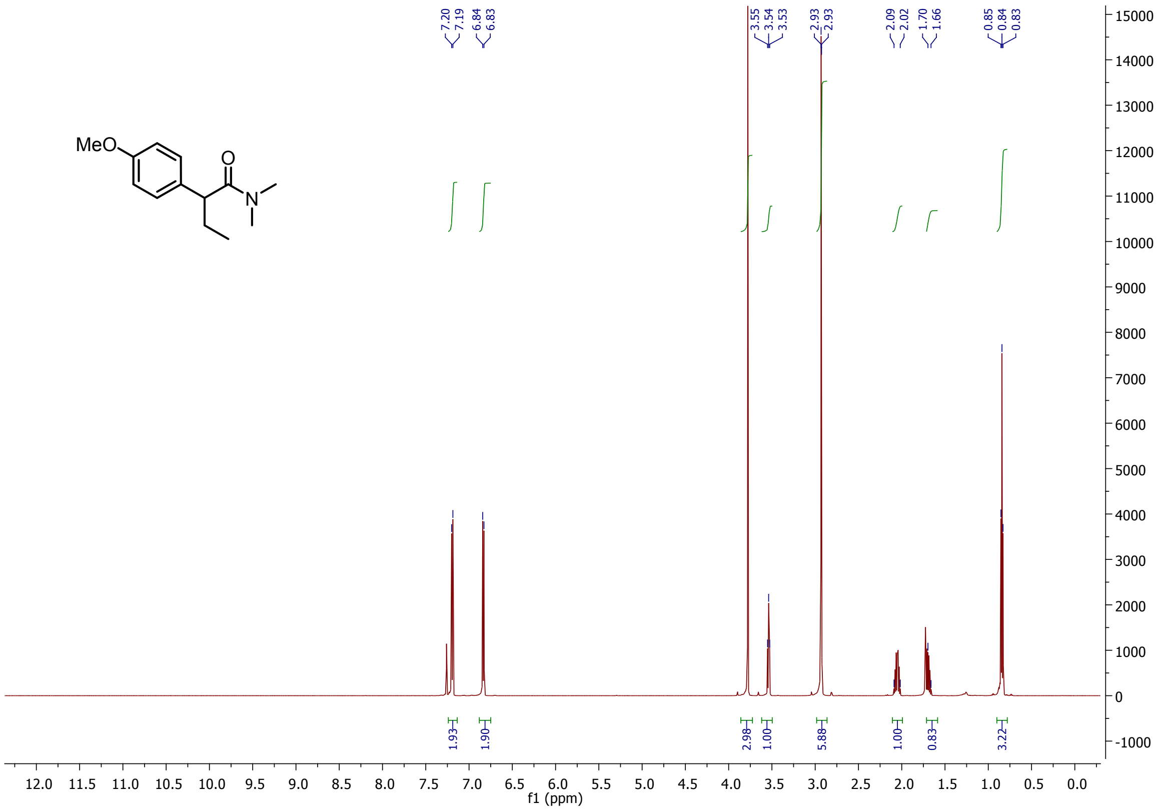
MeO
O
N
7.20
7.19
6.84
6.83
3.55
3.54
3.53
2.93
2.93
2.09
2.02
1.70
1.66
0.85
0.84
0.83
1.93
1.90
2.98
1.00
5.88
1.00
0.83
3.22
f1 (ppm)

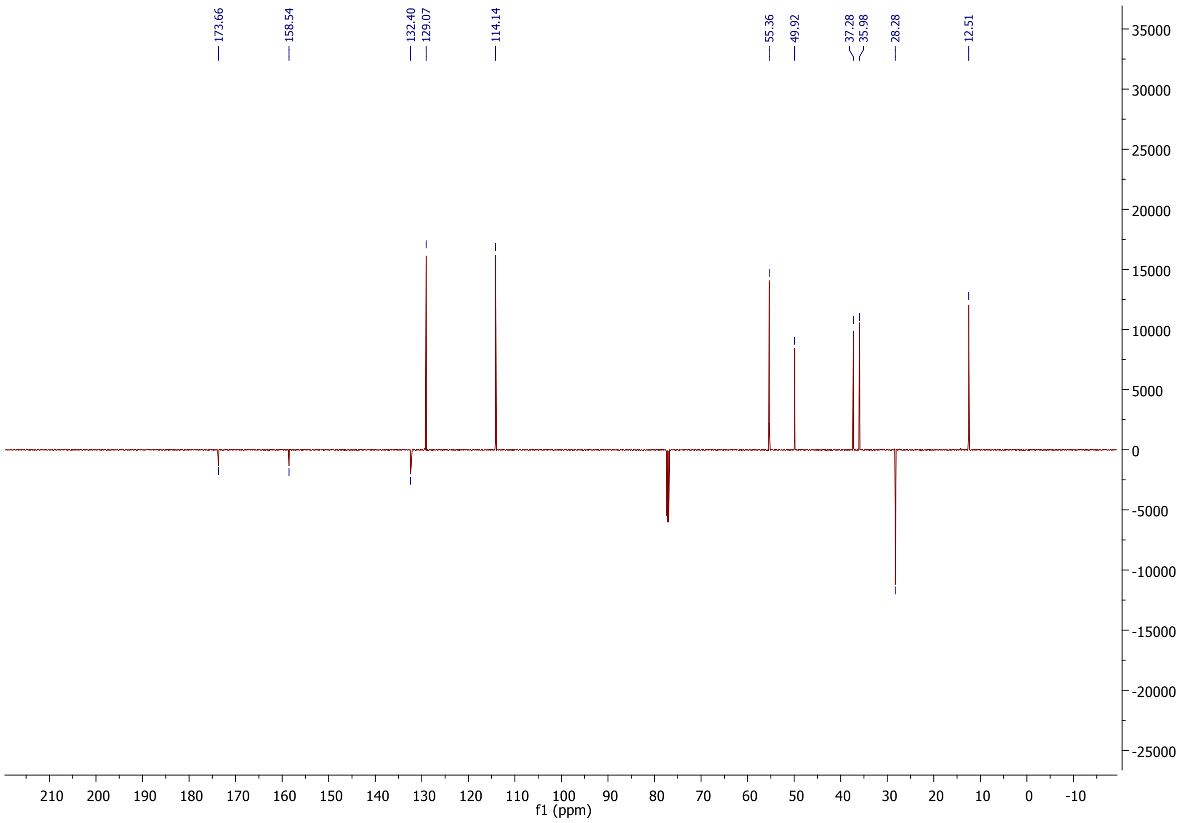
173.66
158.54
132.40
129.07
114.14
55.36
49.92
37.28
35.98
28.28
12.51
35000
30000
25000
20000
15000
10000
5000
0
-5000
-10000
-15000
-20000
-25000
210 200 190 180 170 160 150 140 130 120 110 100 90 80 70 60 50 40 30 20 10 0 -10
f1 (ppm)

Compound **6k**

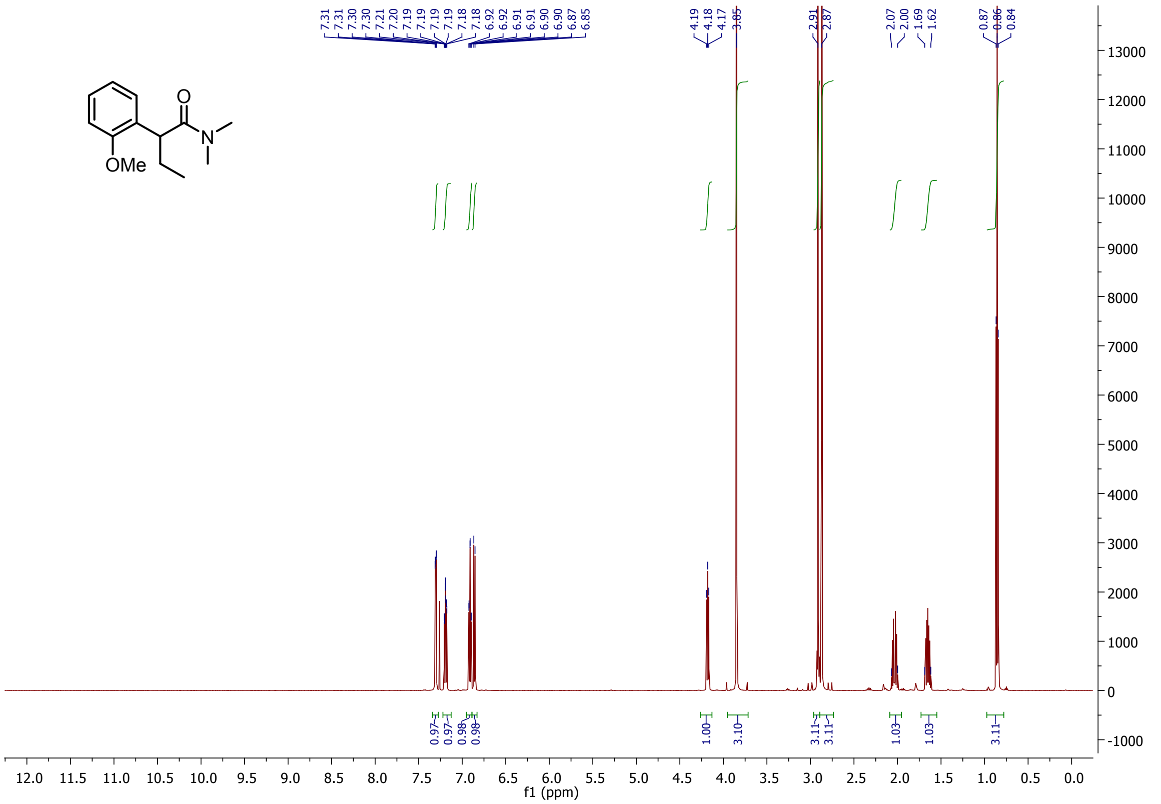
OMe
O
N
7.31
7.31
7.30
7.30
7.21
7.20
7.19
7.19
7.19
7.19
7.18
7.18
6.92
6.92
6.91
6.91
6.90
6.90
6.87
6.85
4.19
4.18
4.17
3.85
2.91
2.87
2.07
2.00
1.69
1.62
0.87
0.86
0.84
0.97
0.97
0.98
0.98
1.00
3.10
3.11
3.11
1.03
1.03
3.11
f1 (ppm)

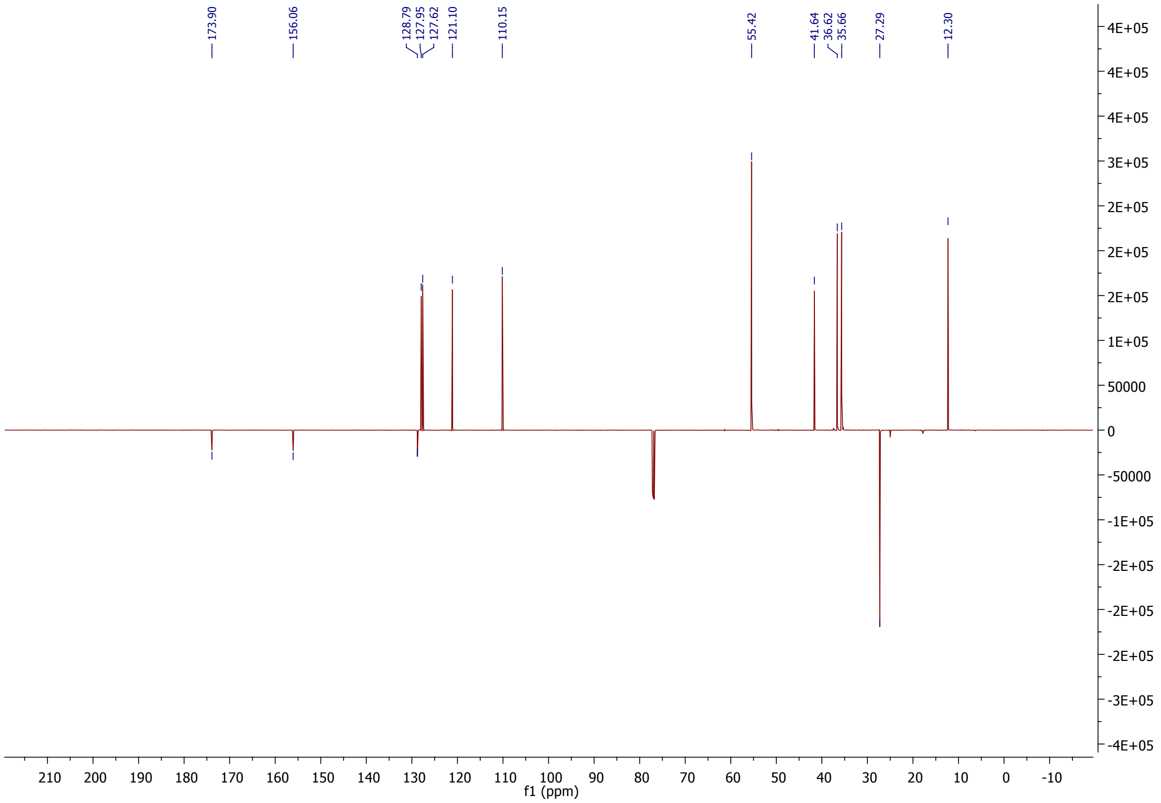
173.90
156.06
128.79
127.95
127.62
121.10
110.15
55.42
41.64
36.62
35.66
27.29
12.30
f1 (ppm)

Compound **6l**

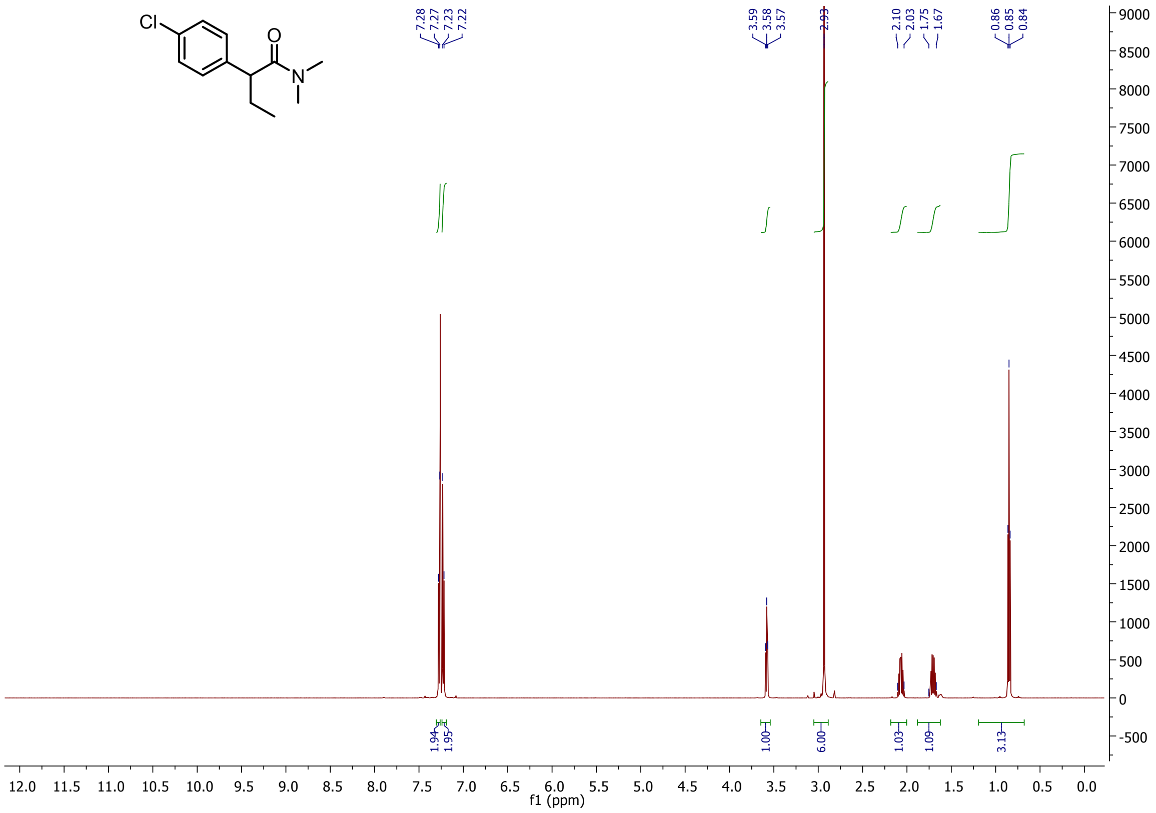
Cl
O
N
7.28
7.27
7.23
7.22
3.59
3.58
3.57
2.93
2.10
2.03
1.75
1.67
0.86
0.85
0.84
1.94
1.95
1.00
6.00
1.03
1.09
3.13
f1 (ppm)

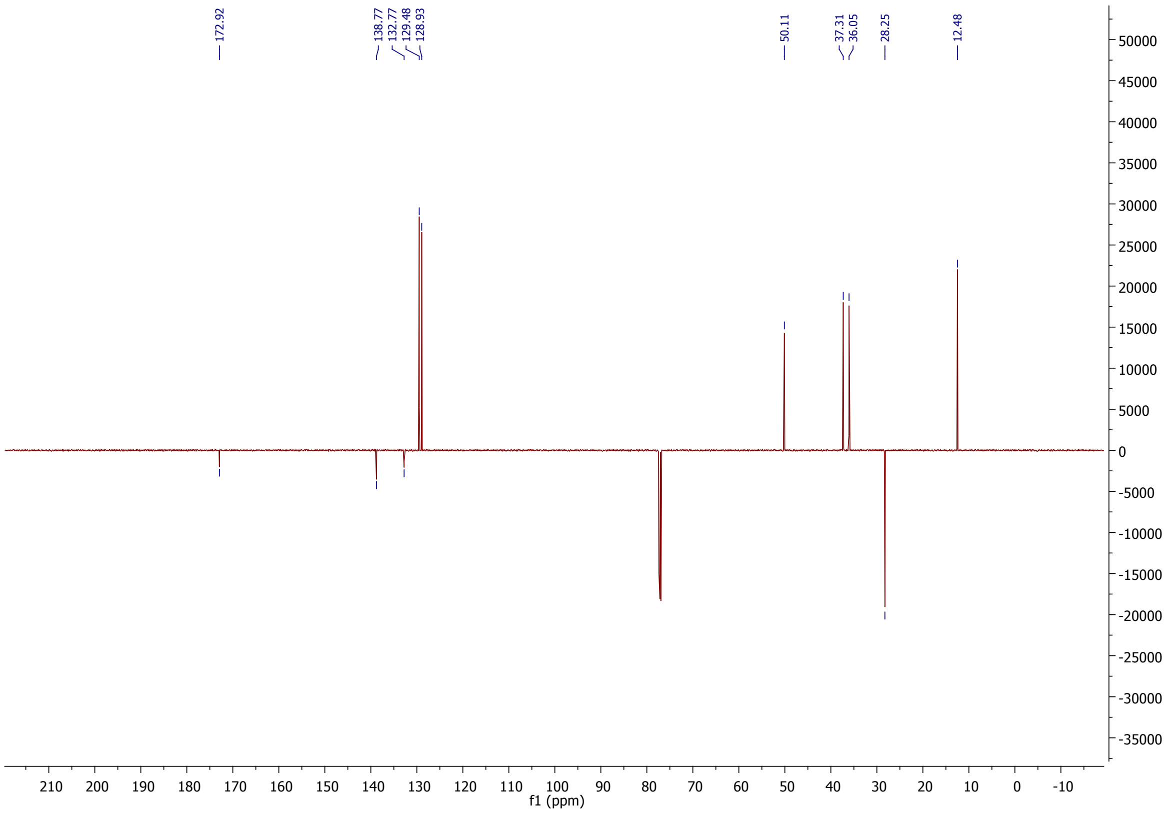
172.92
138.77
132.77
129.48
128.93
50.11
37.31
36.05
28.25
12.48
f1 (ppm)

Compound **6m**

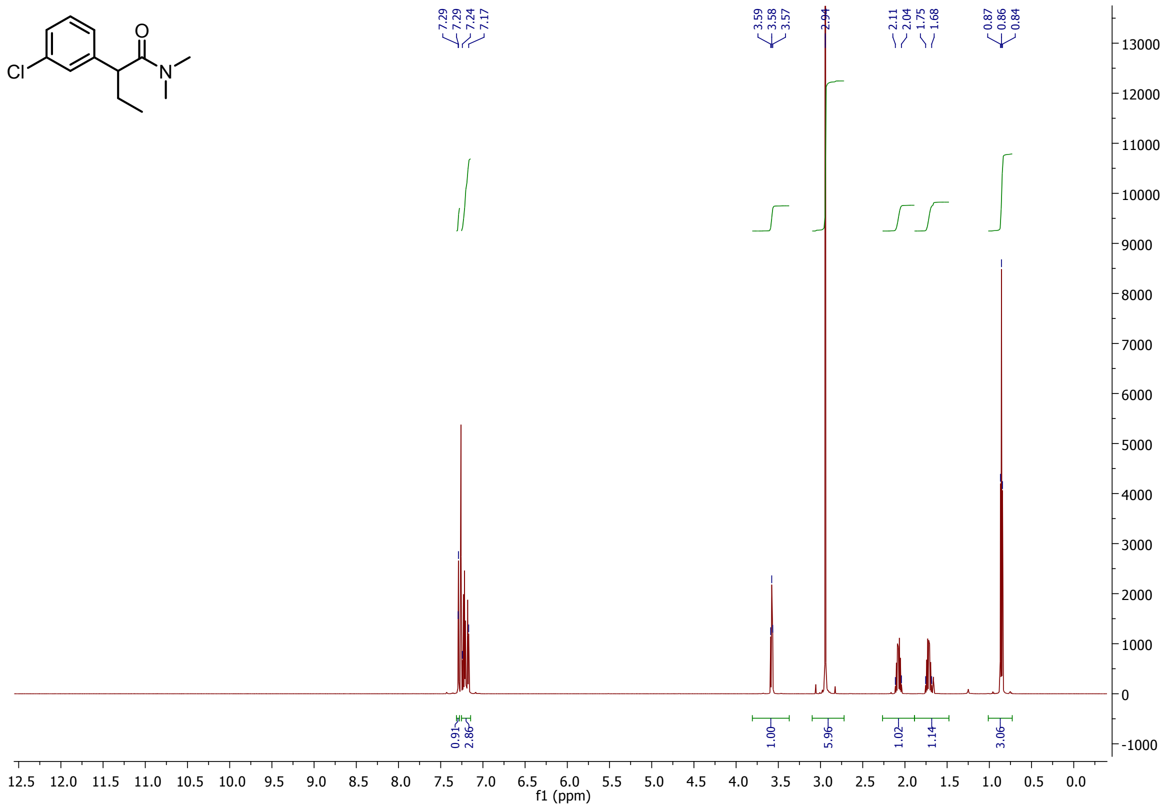
Cl
O
N
7.29
7.29
7.24
7.17
3.59
3.58
3.57
2.94
2.11
2.04
1.75
1.68
0.87
0.86
0.84
0.91
2.86
1.00
5.96
1.02
1.14
3.06
f1 (ppm)

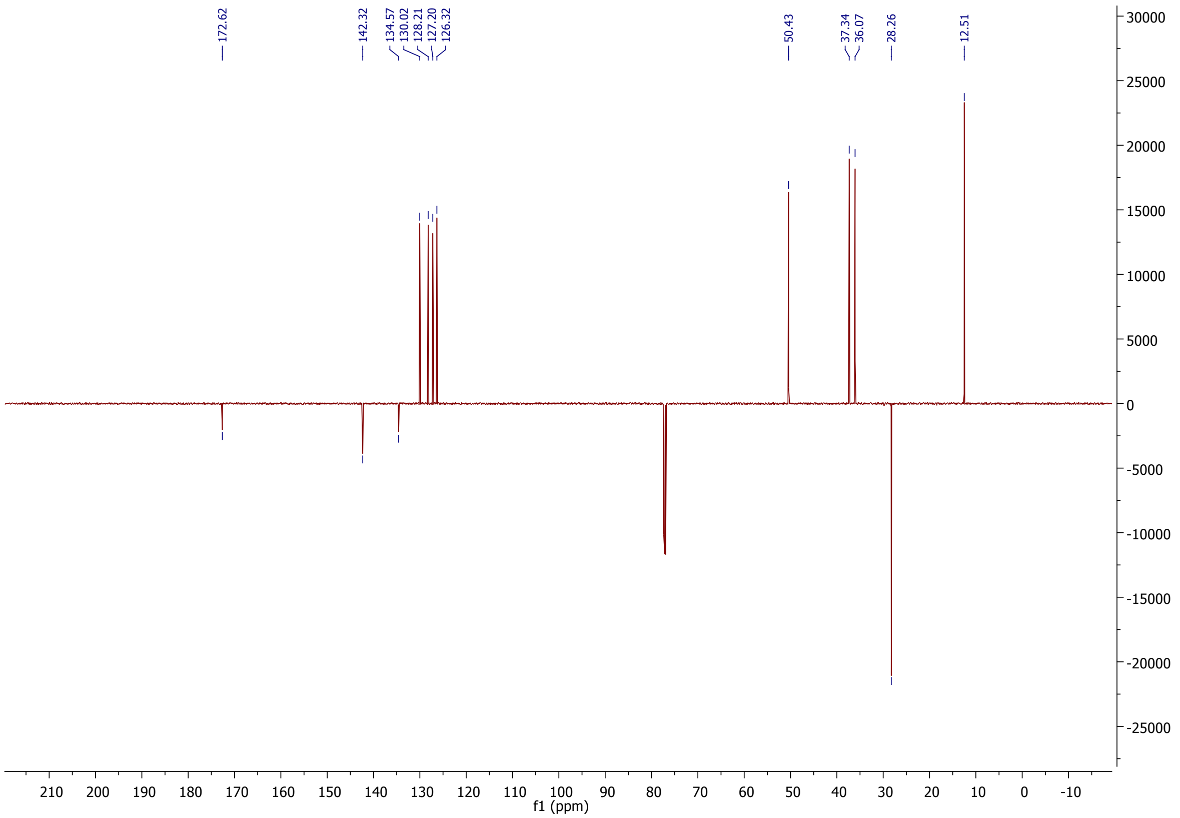
172.62
142.32
134.57
130.02
128.21
127.20
126.32
50.43
37.34
36.07
28.26
12.51
f1 (ppm)

Compound **6n**

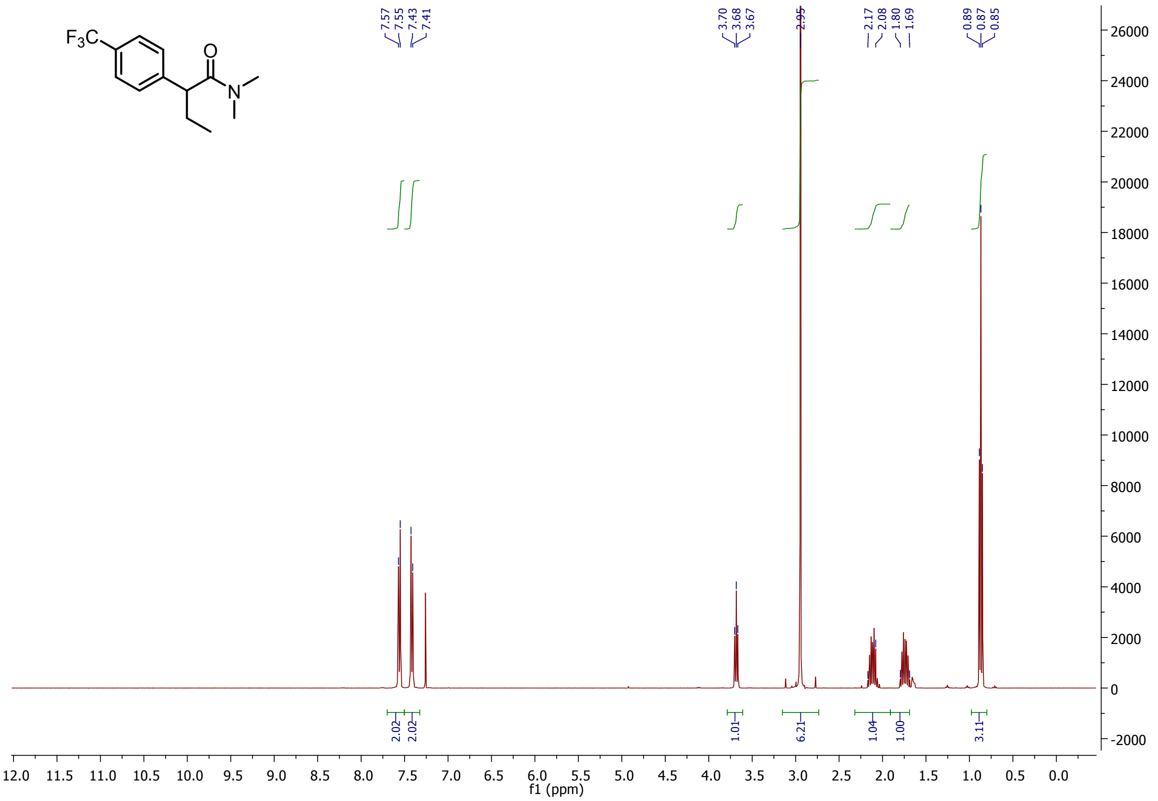

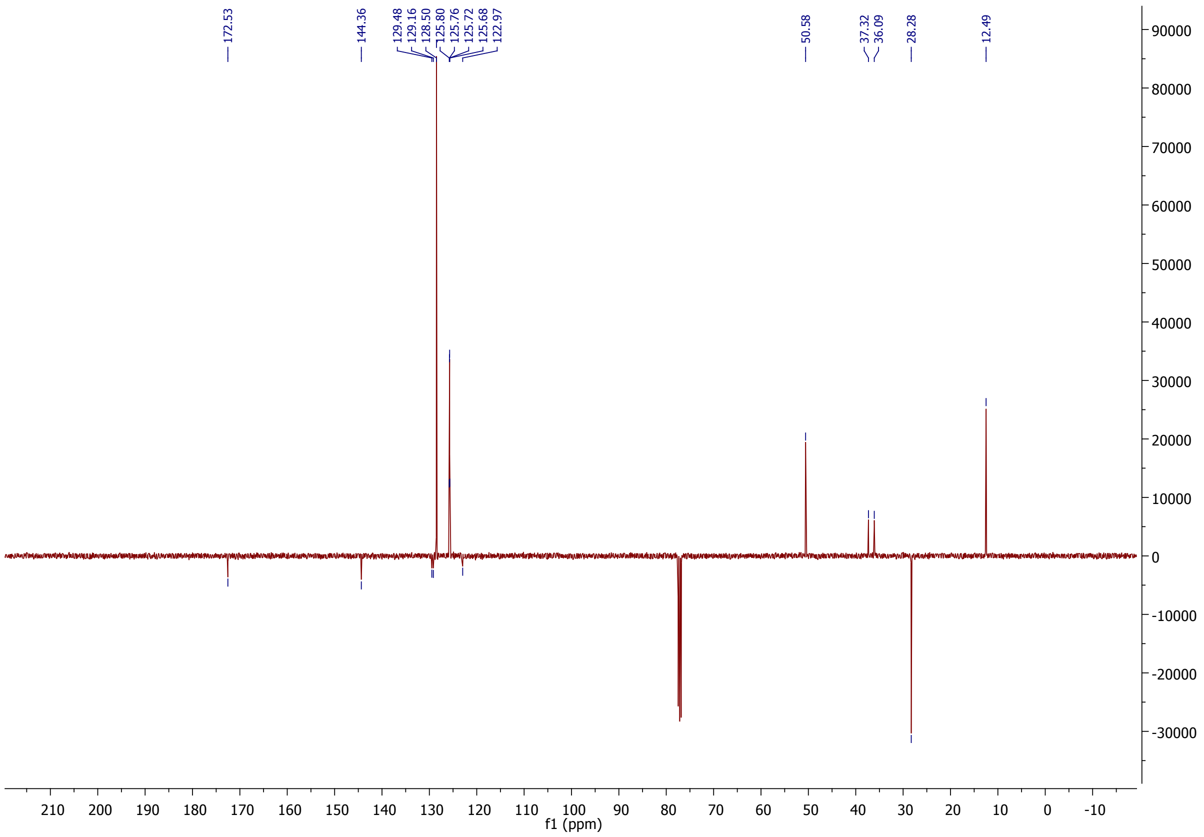
172.53
144.36
129.48
129.16
128.50
125.80
125.76
125.72
125.68
122.97
50.58
37.32
36.09
28.28
12.49
f1 (ppm)

Compound **6o**

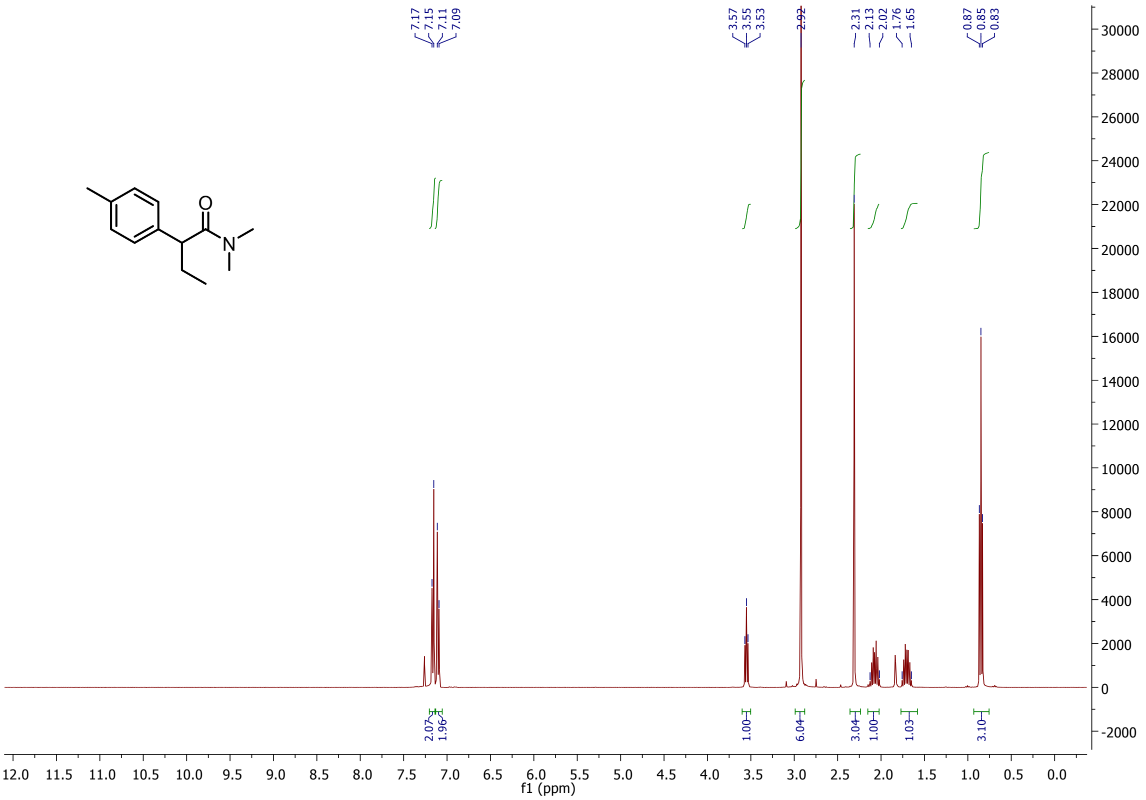
7.17
7.15
7.11
7.09
3.57
3.55
3.53
2.92
2.31
2.13
2.02
1.76
1.65
0.87
0.85
0.83
2.07
1.96
1.00
6.04
3.04
1.00
1.03
3.10
f1 (ppm)

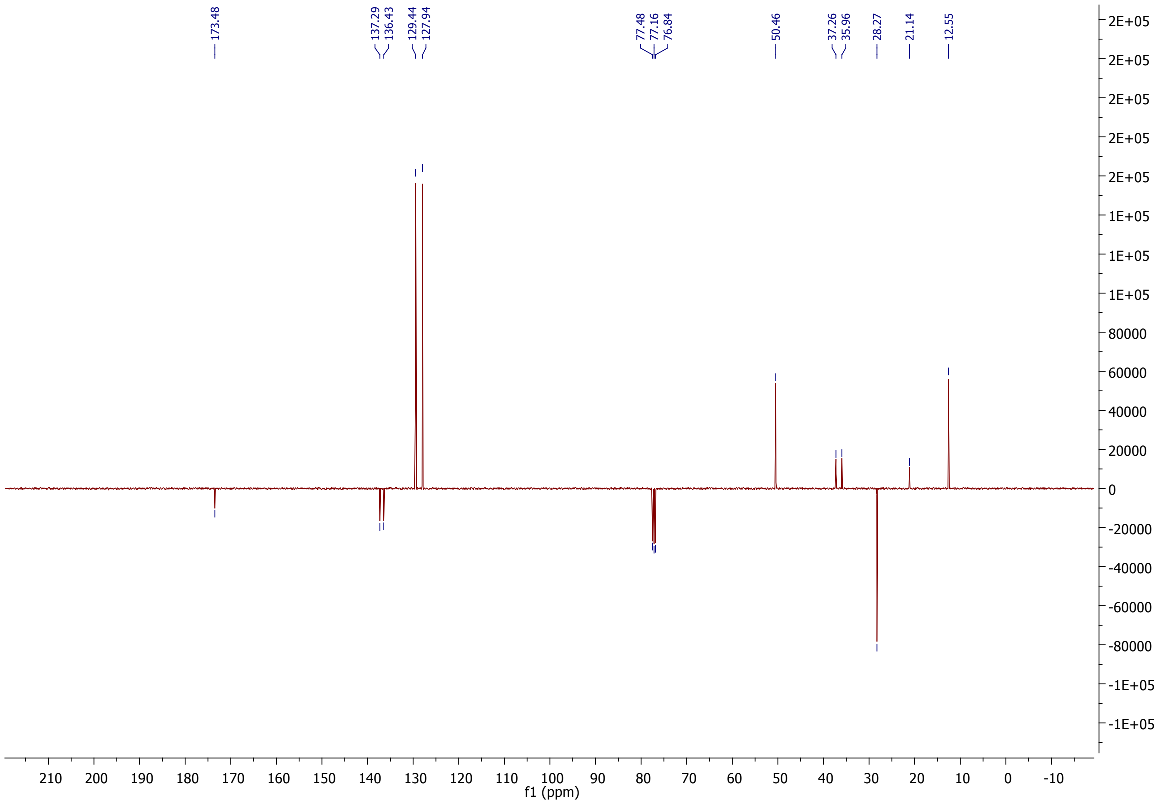
173.48
137.29
136.43
129.44
127.94
77.48
77.16
76.84
50.46
37.26
35.96
28.27
21.14
12.55
f1 (ppm)

Compound **6p**

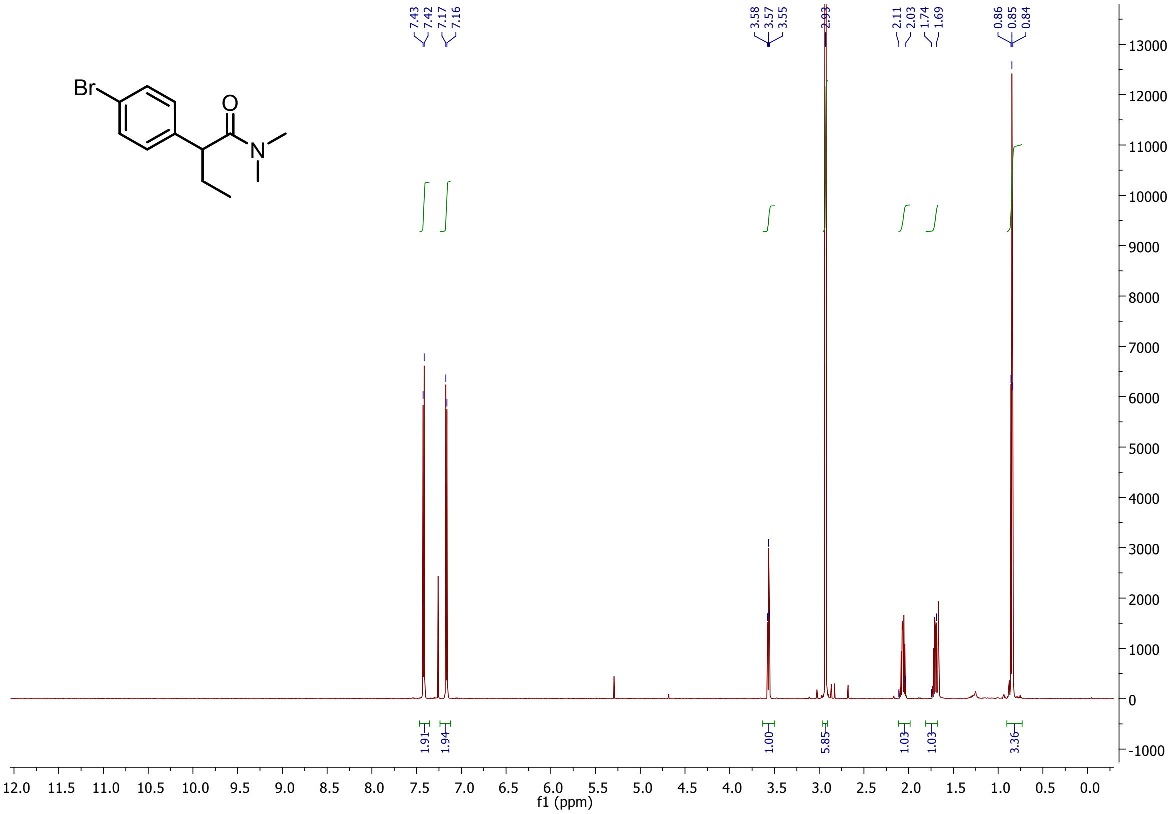
7.43
7.42
7.17
7.16
3.58
3.57
3.55
2.93
2.11
2.03
1.74
1.69
0.86
0.85
0.84
1.91
1.94
1.00
5.85
1.03
1.03
3.36
f1 (ppm)

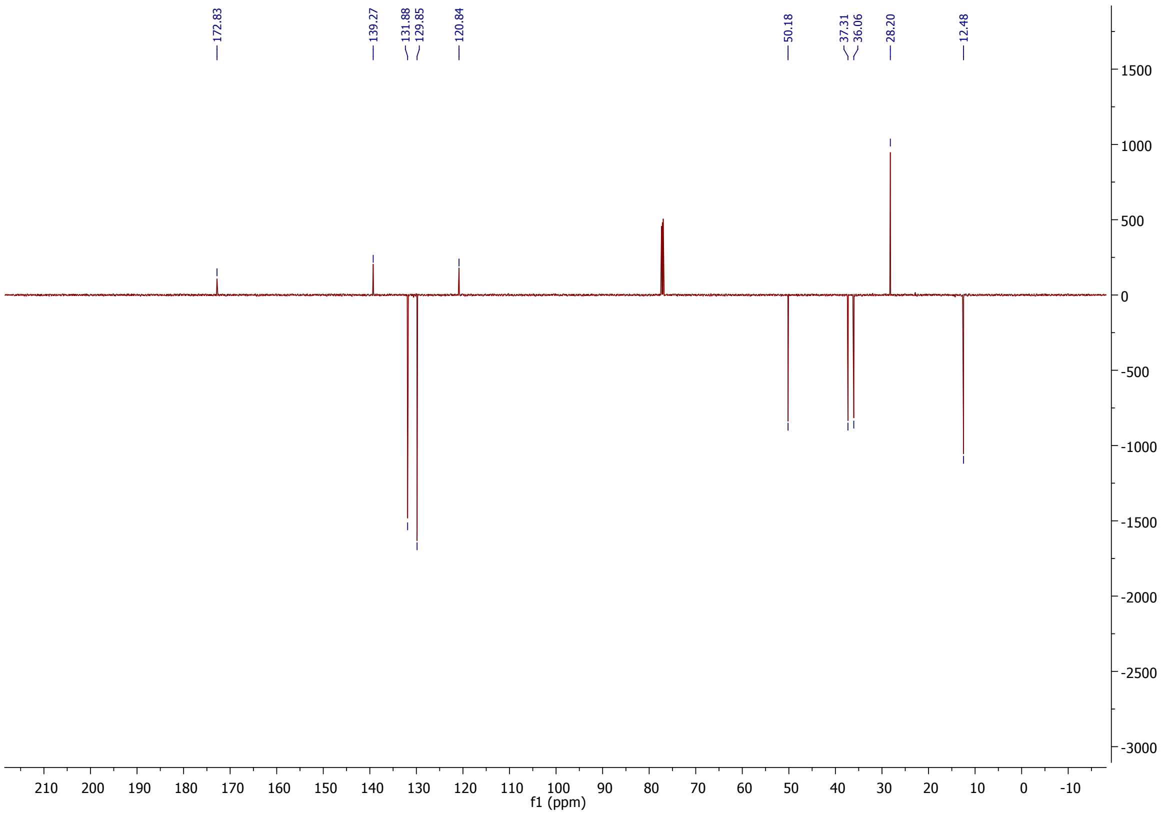

172.83
139.27
131.88
129.85
120.84
50.18
37.31
36.06
28.20
12.48
1500
1000
500
0
-500
-1000
-1500
-2000
-2500
-3000
210 200 190 180 170 160 150 140 130 120 110 100 90 80 70 60 50 40 30 20 10 0 -10
f1 (ppm)

Compound **6q**

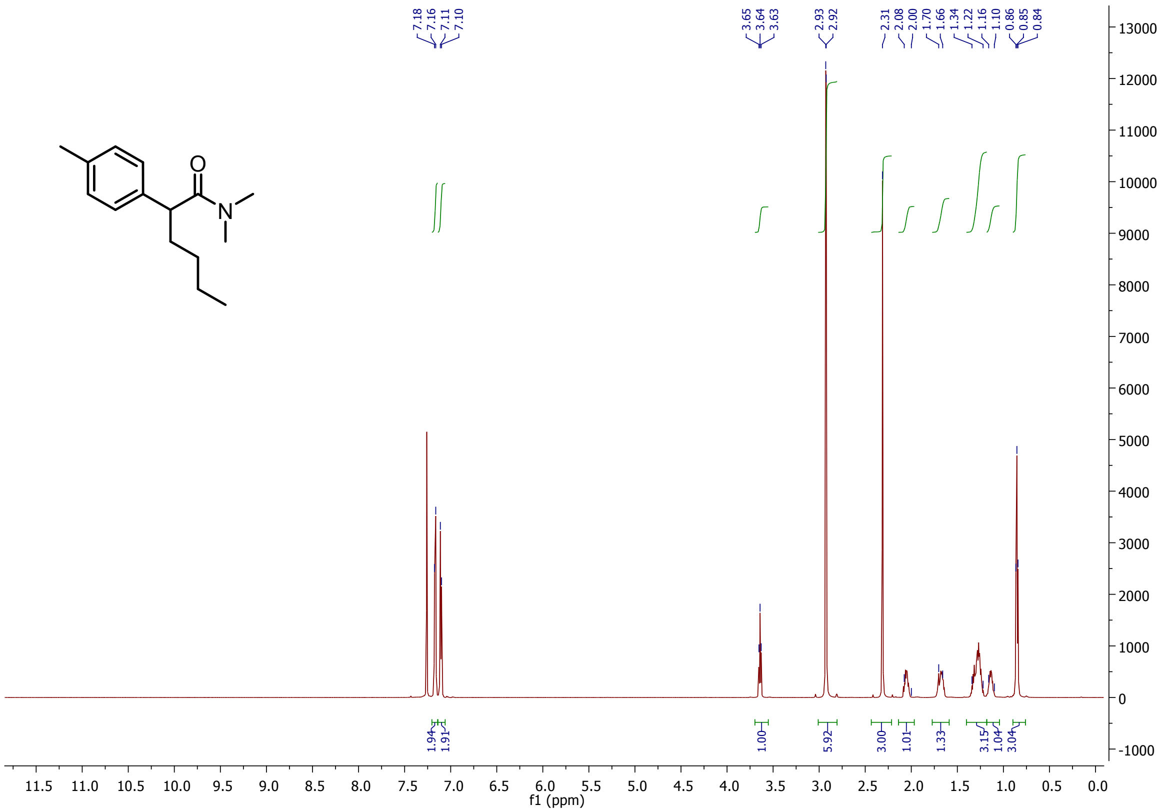
f1 (ppm)

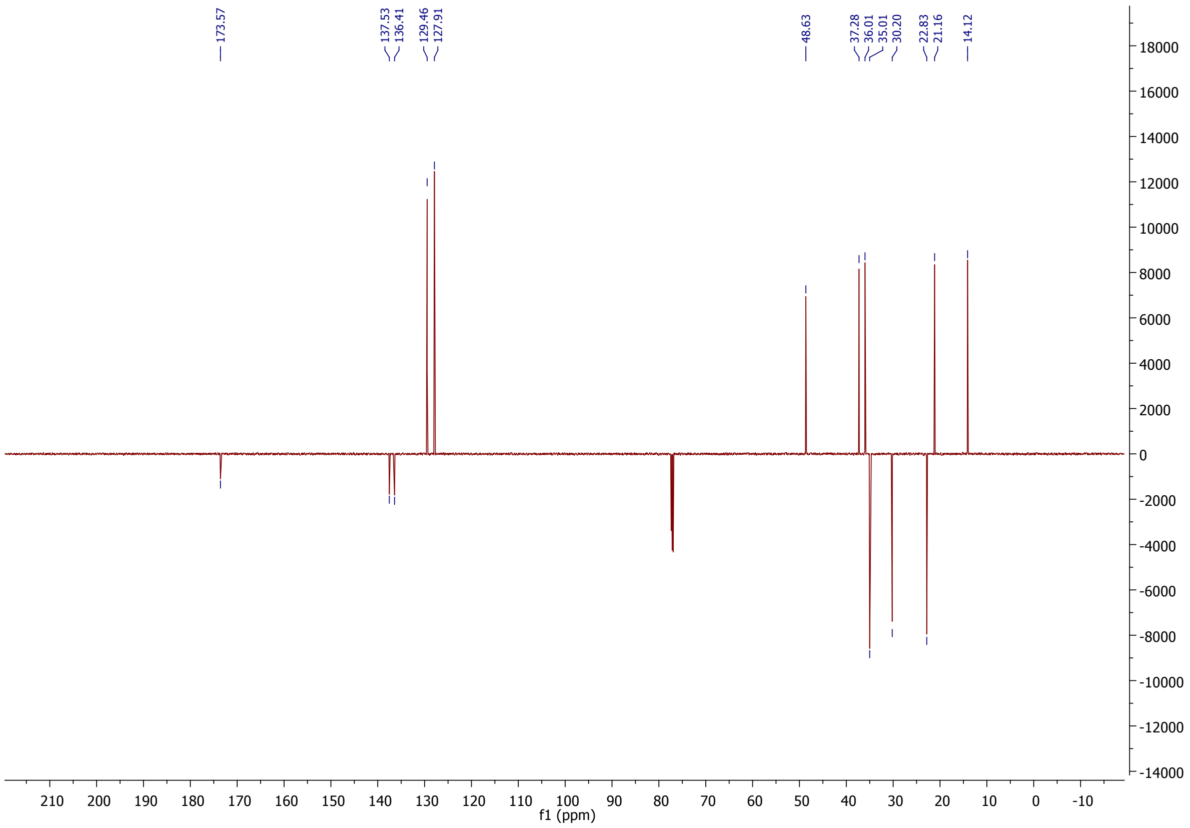
173.57
137.53
136.41
129.46
127.91
48.63
37.28
36.01
35.01
30.20
22.83
21.16
14.12
18000
16000
14000
12000
10000
8000
6000
4000
2000
0
-2000
-4000
-6000
-8000
-10000
-12000
-14000
210
200
190
180
170
160
150
140
130
120
110
100
90
80
70
60
50
40
30
20
10
0
-10
f1 (ppm)

Compound **6r**

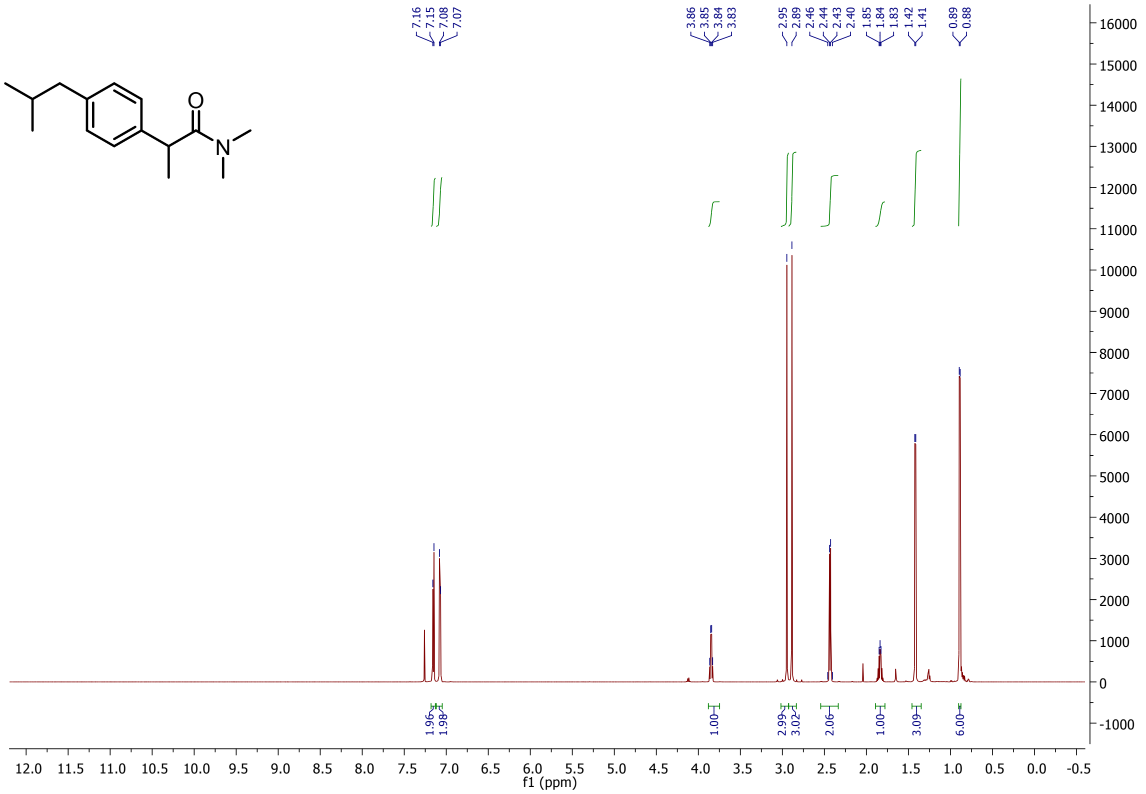
O
N
7.16
7.15
7.08
7.07
3.86
3.85
3.84
3.83
2.95
2.89
2.46
2.44
2.43
2.40
1.85
1.84
1.83
1.42
1.41
0.89
0.88
1.96
1.98
1.00
2.99
3.02
2.06
1.00
3.09
6.00
f1 (ppm)

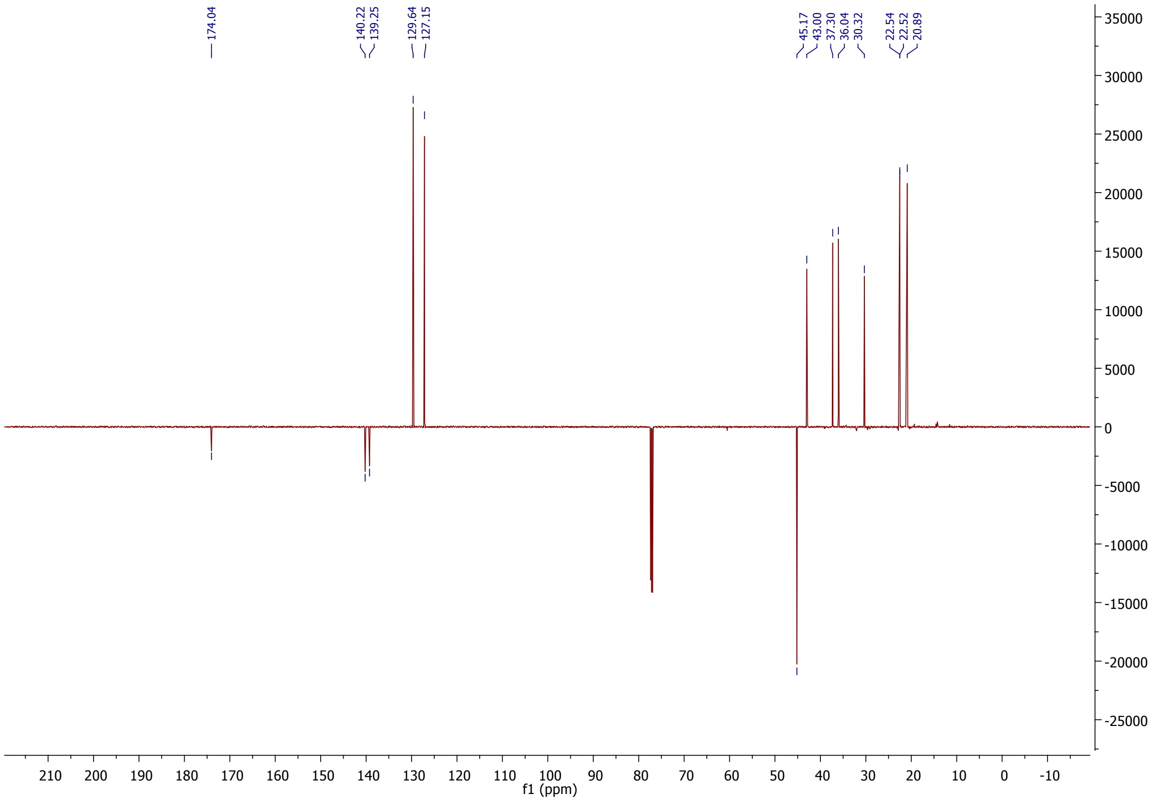
174.04
140.22
139.25
129.64
127.15
45.17
43.00
37.30
36.04
30.32
22.54
22.52
20.89
f1 (ppm)

Compound **6s**

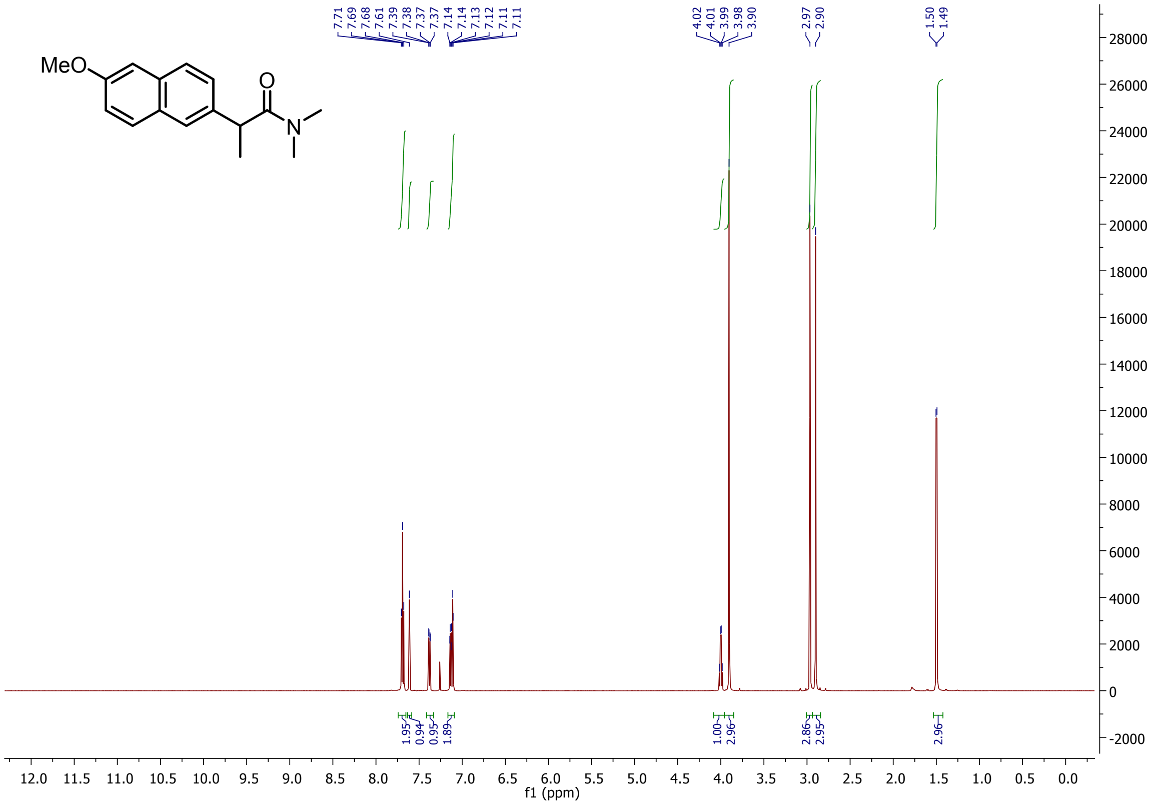
MeO
O
N
7.71
7.69
7.68
7.61
7.39
7.38
7.37
7.37
7.14
7.14
7.13
7.12
7.11
7.11
4.02
4.01
3.99
3.98
3.90
2.97
2.90
1.50
1.49
1.95
0.94
0.95
1.89
1.00
2.96
2.86
2.95
2.96
f1 (ppm)

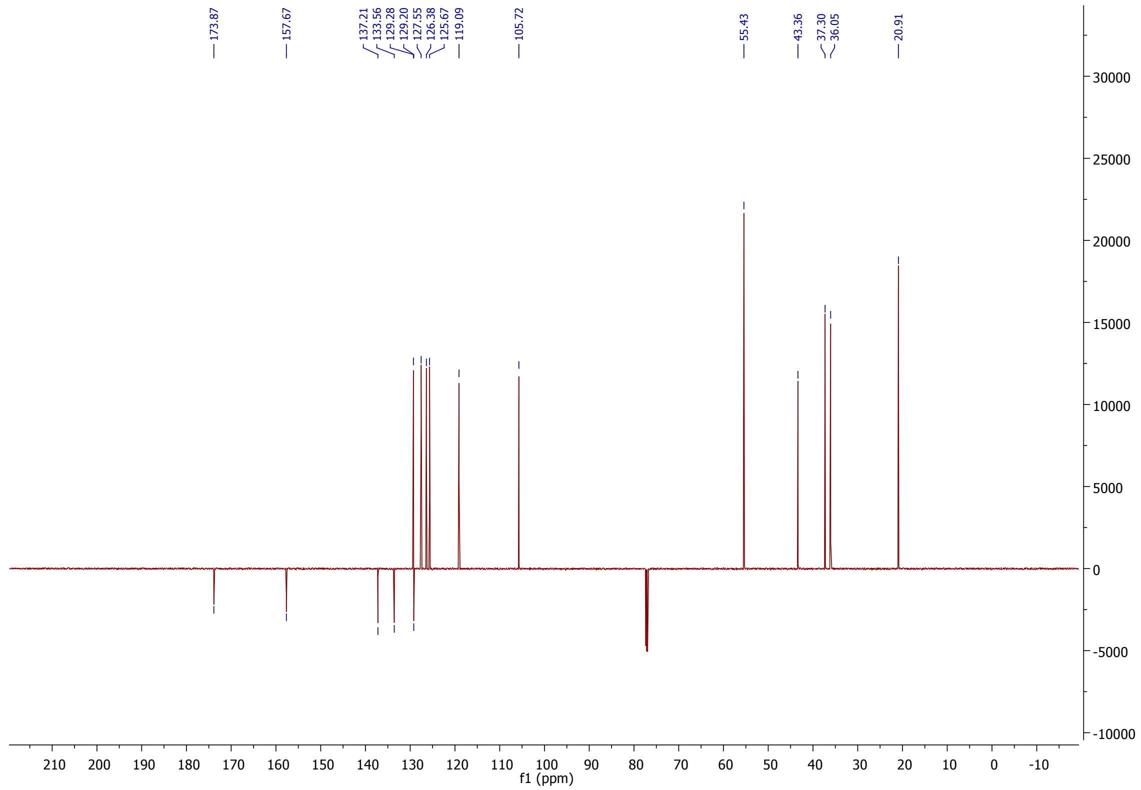
173.87
157.67
137.21
133.56
129.28
129.20
127.55
126.38
125.67
119.09
105.72
55.43
43.36
37.30
36.05
20.91
30000
25000
20000
15000
10000
5000
0
-5000
-10000
210 200 190 180 170 160 150 140 130 120 110 100 90 80 70 60 50 40 30 20 10 0 -10
f1 (ppm)

Compound **6t**

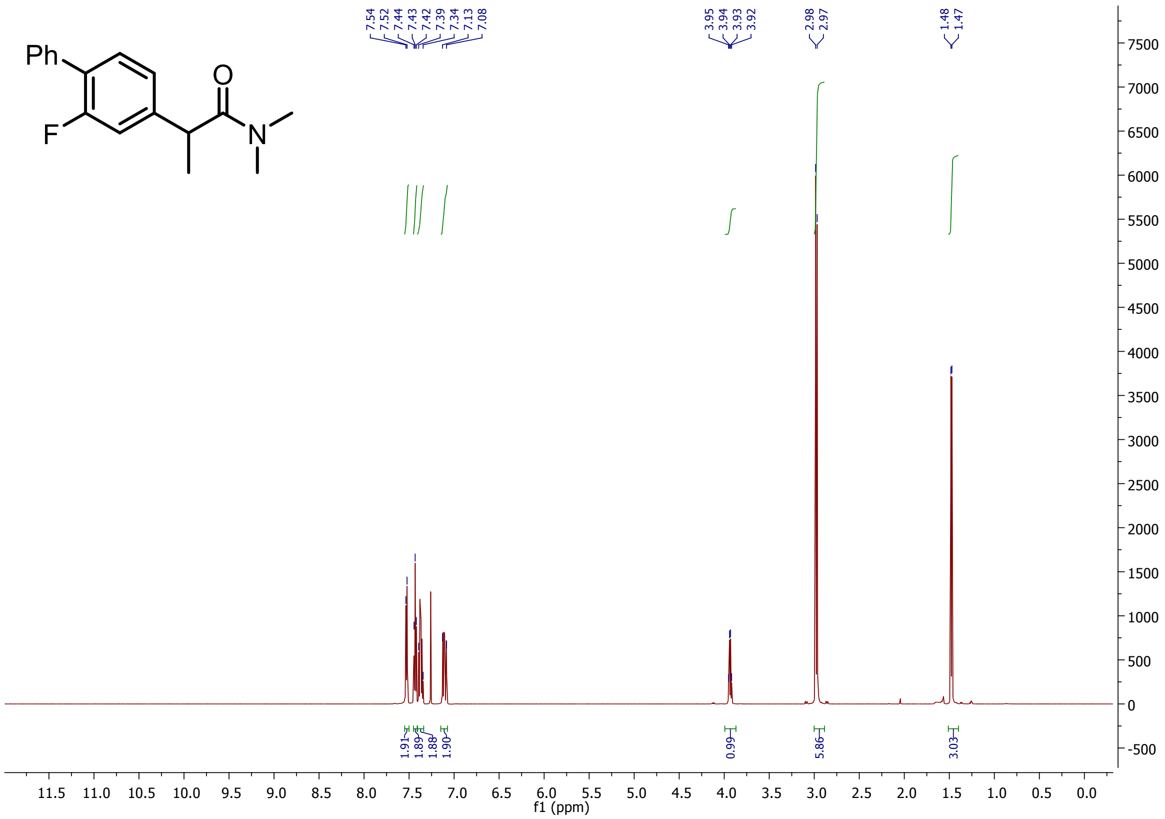
Ph
F
O
N
7.54
7.52
7.44
7.43
7.42
7.39
7.34
7.13
7.08
3.95
3.94
3.93
3.92
2.98
2.97
1.48
1.47
1.91
1.89
1.88
1.90
0.99
5.86
3.03
f1 (ppm)

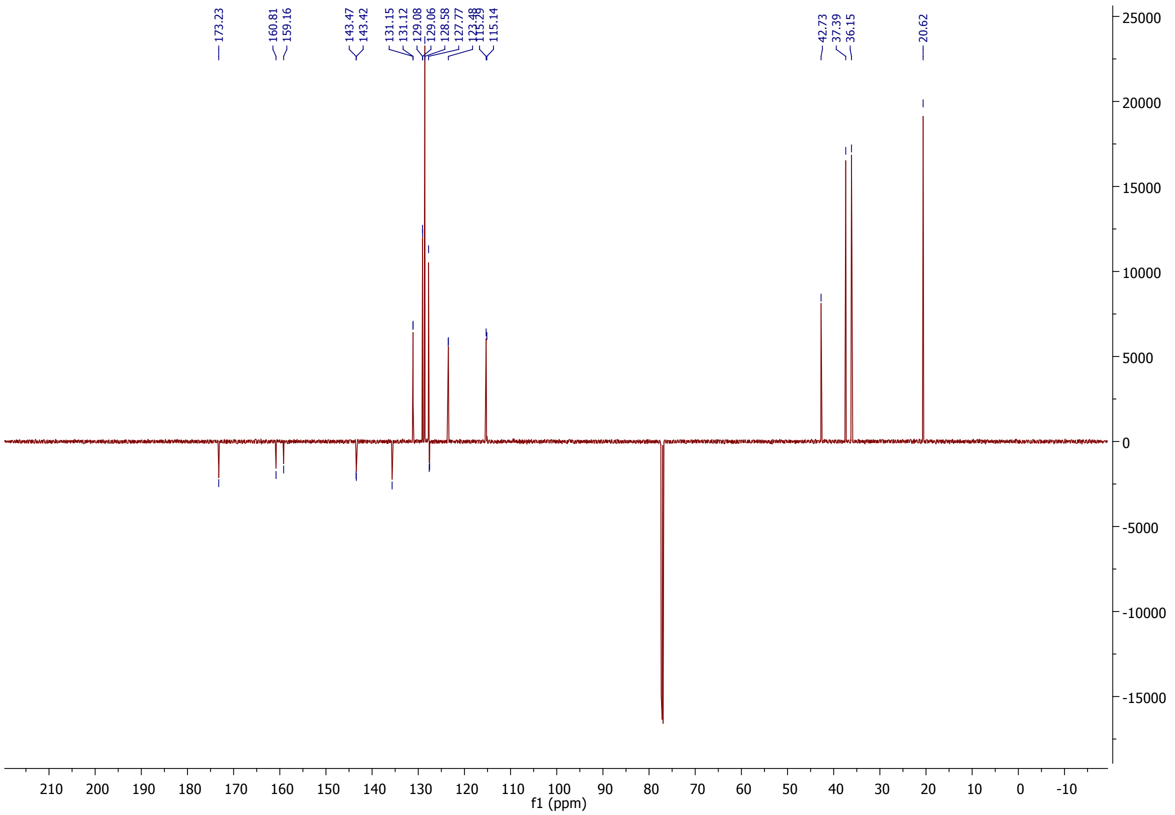
173.23
160.81
159.16
143.47
143.42
131.15
131.12
129.08
129.06
128.58
127.77
123.48
115.29
115.14
42.73
37.39
36.15
20.62
25000
20000
15000
10000
5000
0
-5000
-10000
-15000
210 200 190 180 170 160 150 140 130 120 110 100 90 80 70 60 50 40 30 20 10 0 -10
f1 (ppm)

Compound **6u**

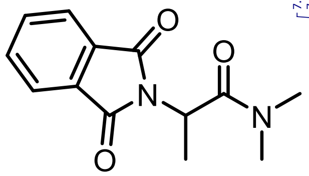

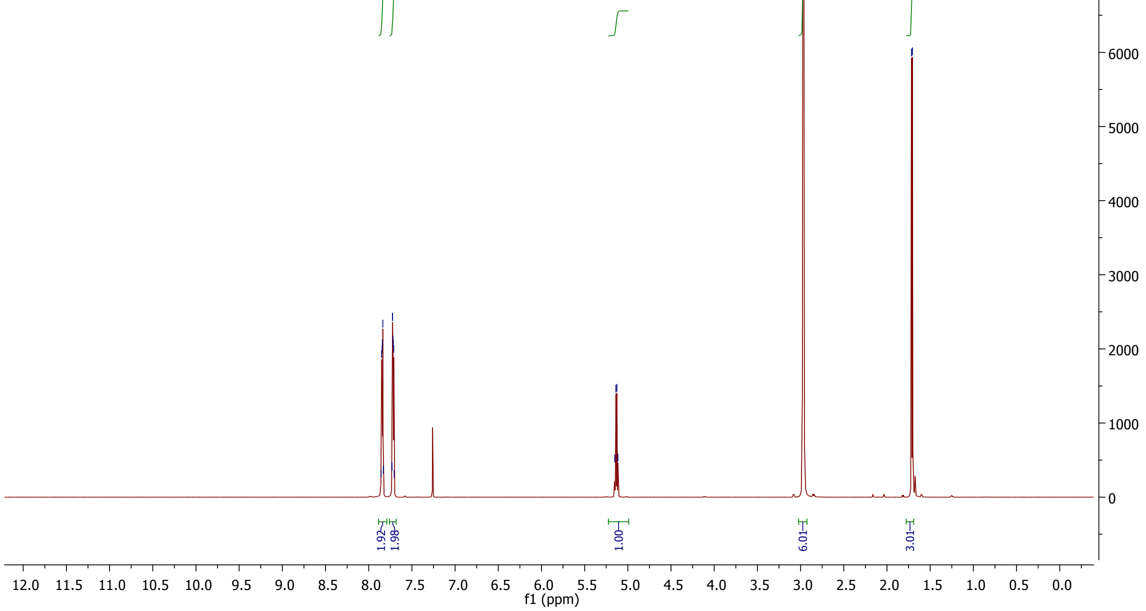
7.86
7.85
7.85
7.84
7.84
7.83
7.83
7.73
7.73
7.72
7.72
7.72
7.72
7.71
7.70
5.15
5.14
5.13
5.11
2.97
2.96
1.72
1.70
1.92
1.98
1.00
6.01
3.01
12.0 11.5 11.0 10.5 10.0 9.5 9.0 8.5 8.0 7.5 7.0 6.5 6.0 5.5 5.0 4.5 4.0 3.5 3.0 2.5 2.0 1.5 1.0 0.5 0.0
f1 (ppm)
9000
8000
7000
6000
5000
4000
3000
2000
1000
0

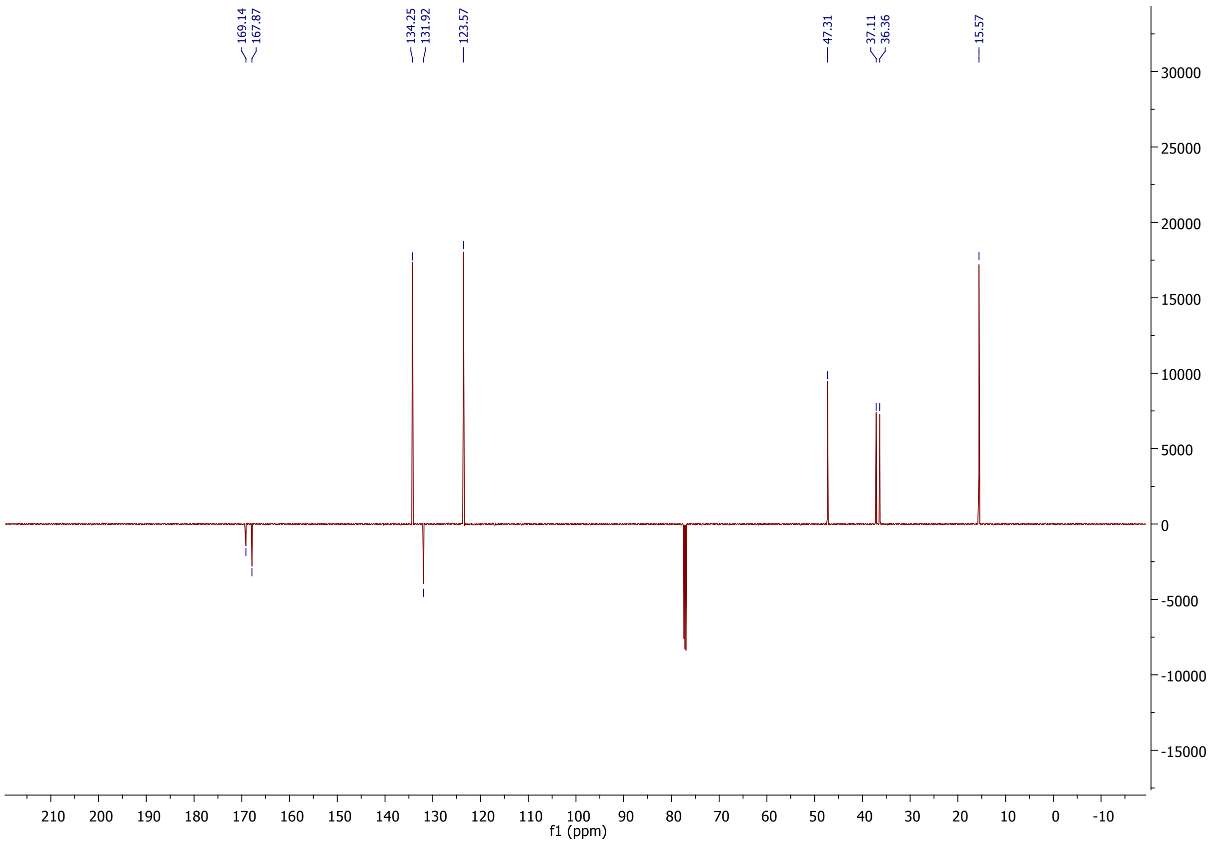
169.14
167.87
134.25
131.92
123.57
47.31
37.11
36.36
15.57
30000
25000
20000
15000
10000
5000
0
-5000
-10000
-15000
210 200 190 180 170 160 150 140 130 120 110 100 90 80 70 60 50 40 30 20 10 0 -10
f1 (ppm)

Compound **6v**

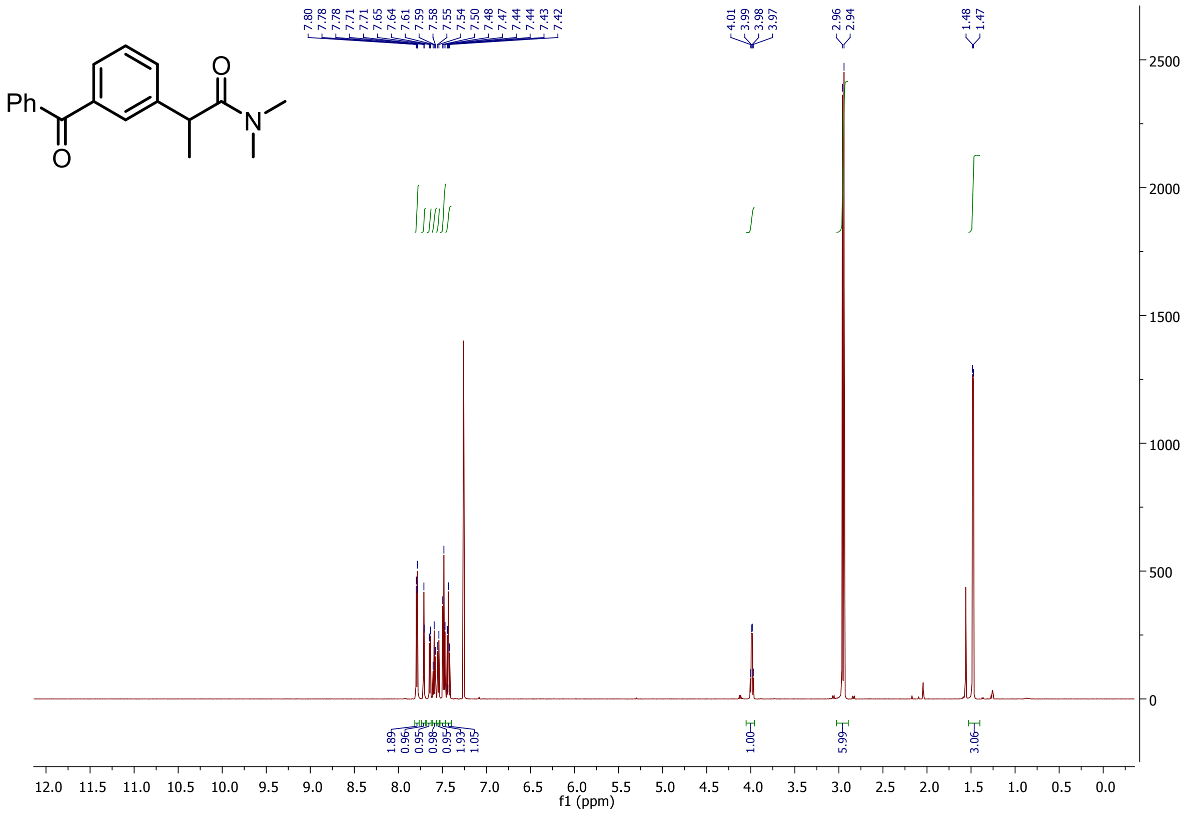
Ph
O
O
N
7.80
7.78
7.78
7.71
7.71
7.65
7.64
7.61
7.59
7.58
7.55
7.54
7.50
7.48
7.47
7.44
7.44
7.43
7.42
4.01
3.99
3.98
3.97
2.96
2.94
1.48
1.47
2500
2000
1500
1000
500
0
1.89
0.96
0.95
0.98
0.95
1.93
1.05
1.00
5.99
3.06
12.0 11.5 11.0 10.5 10.0 9.5 9.0 8.5 8.0 7.5 7.0 6.5 6.0 5.5 5.0 4.5 4.0 3.5 3.0 2.5 2.0 1.5 1.0 0.5 0.0
f1 (ppm)

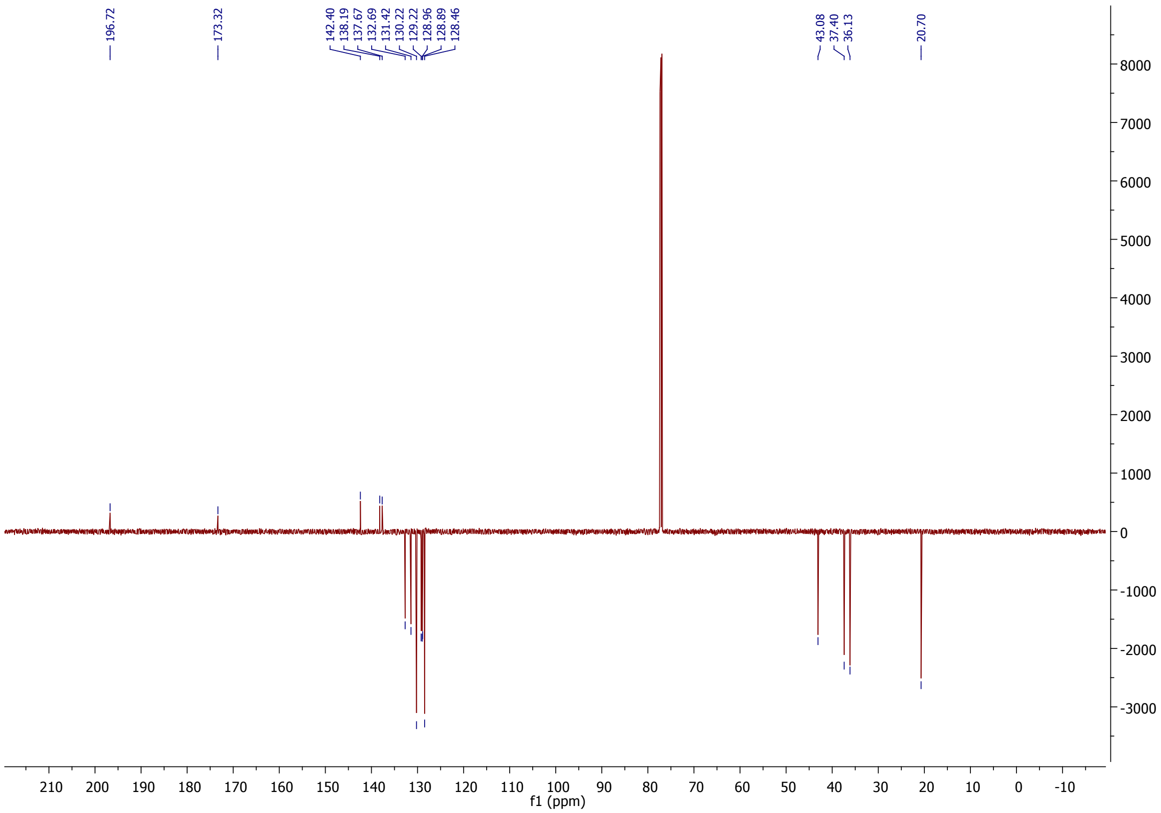
196.72
173.32
142.40
138.19
137.67
132.69
131.42
130.22
129.22
128.96
128.89
128.46
43.08
37.40
36.13
20.70
8000
7000
6000
5000
4000
3000
2000
1000
0
-1000
-2000
-3000
210 200 190 180 170 160 150 140 130 120 110 100 90 80 70 60 50 40 30 20 10 0 -10
f1 (ppm)

Compound **6w**

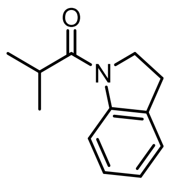

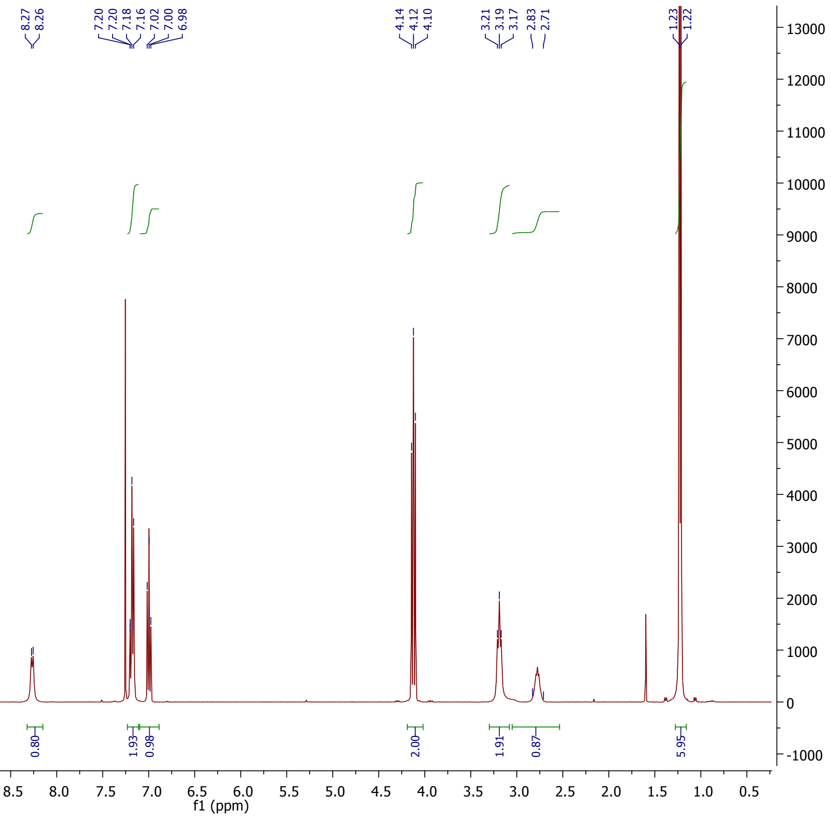
8.27
8.26
7.20
7.20
7.18
7.16
7.02
7.00
6.98
4.14
4.12
4.10
3.21
3.19
3.17
2.83
2.71
1.23
1.22
0.80
1.93
0.98
2.00
1.91
0.87
5.95
f1 (ppm)

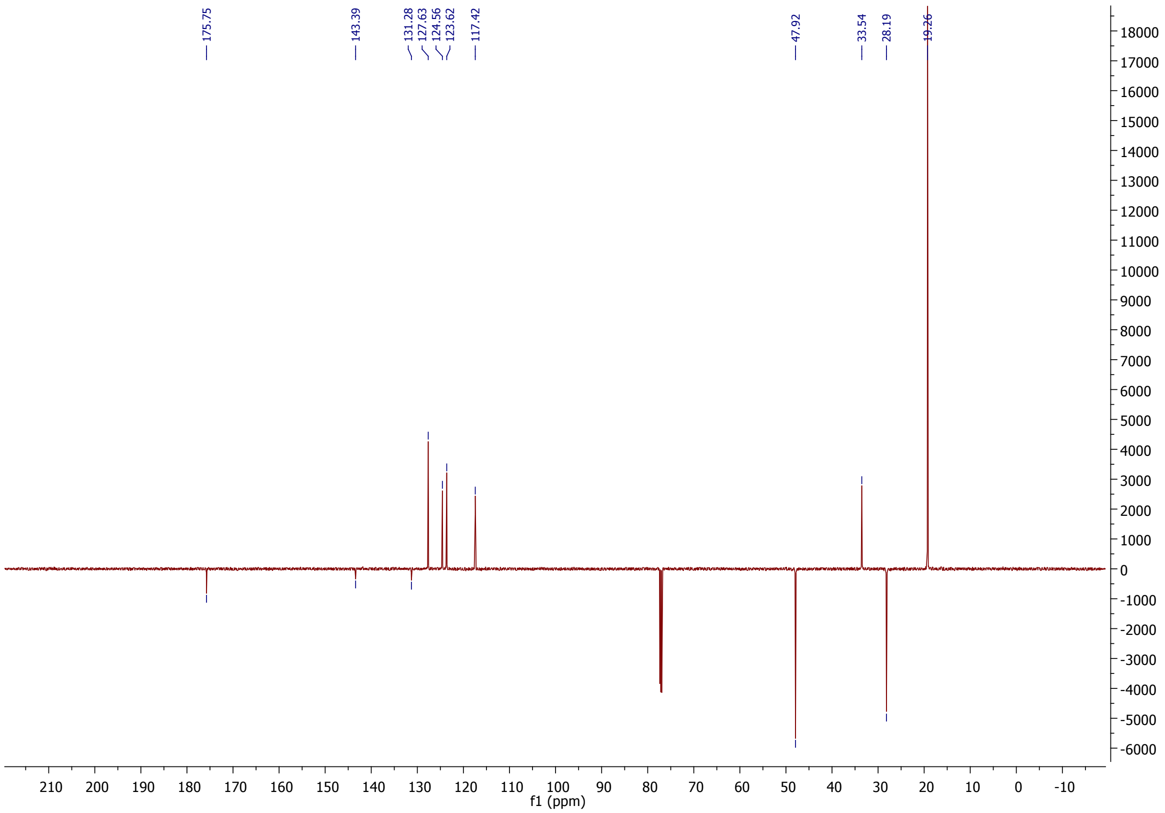
175.75
143.39
131.28
127.63
124.56
123.62
117.42
47.92
33.54
28.19
19.26
f1 (ppm)

Compound **7a**

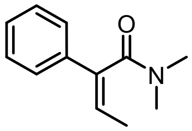

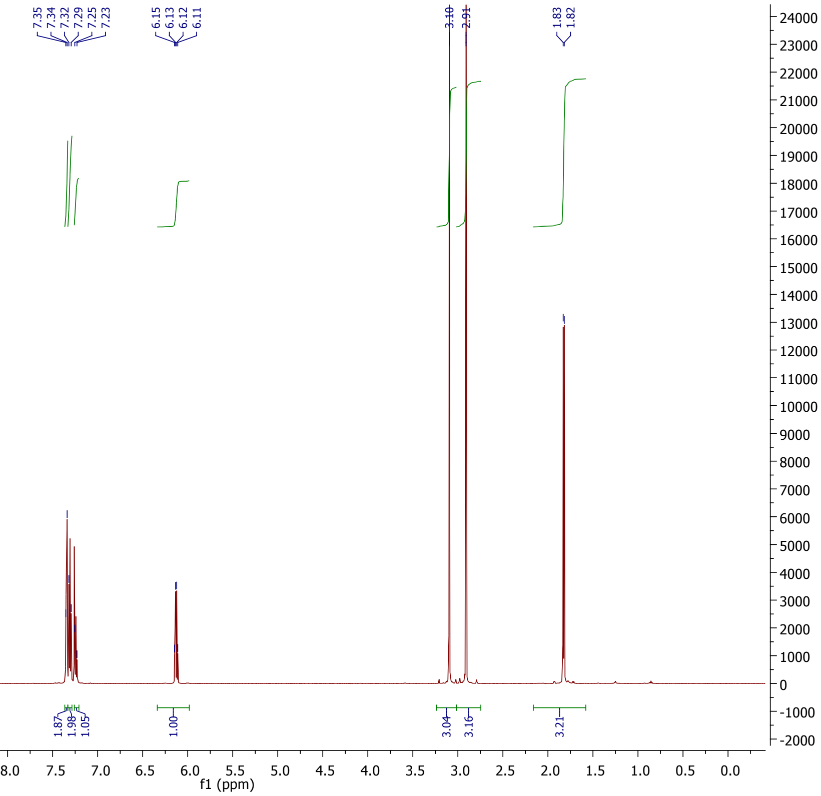
7.35
7.34
7.32
7.29
7.25
7.23
6.15
6.13
6.12
6.11
3.10
2.91
1.83
1.82
1.87
1.98
1.05
1.00
3.04
3.16
3.21
f1 (ppm)

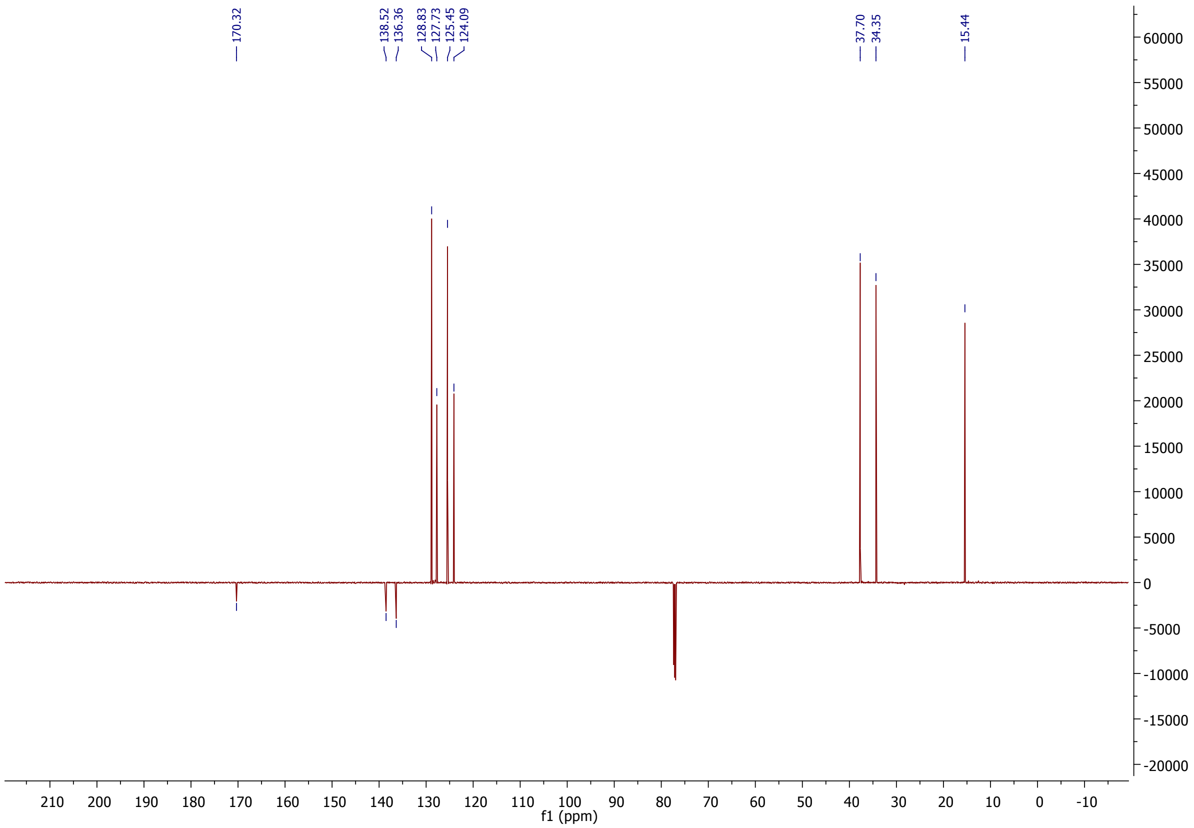
170.32
138.52
136.36
128.83
127.73
125.45
124.09
37.70
34.35
15.44
f1 (ppm)

Compound **8r**

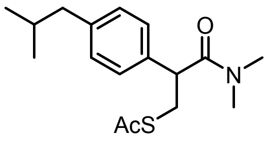
O
N
AcS

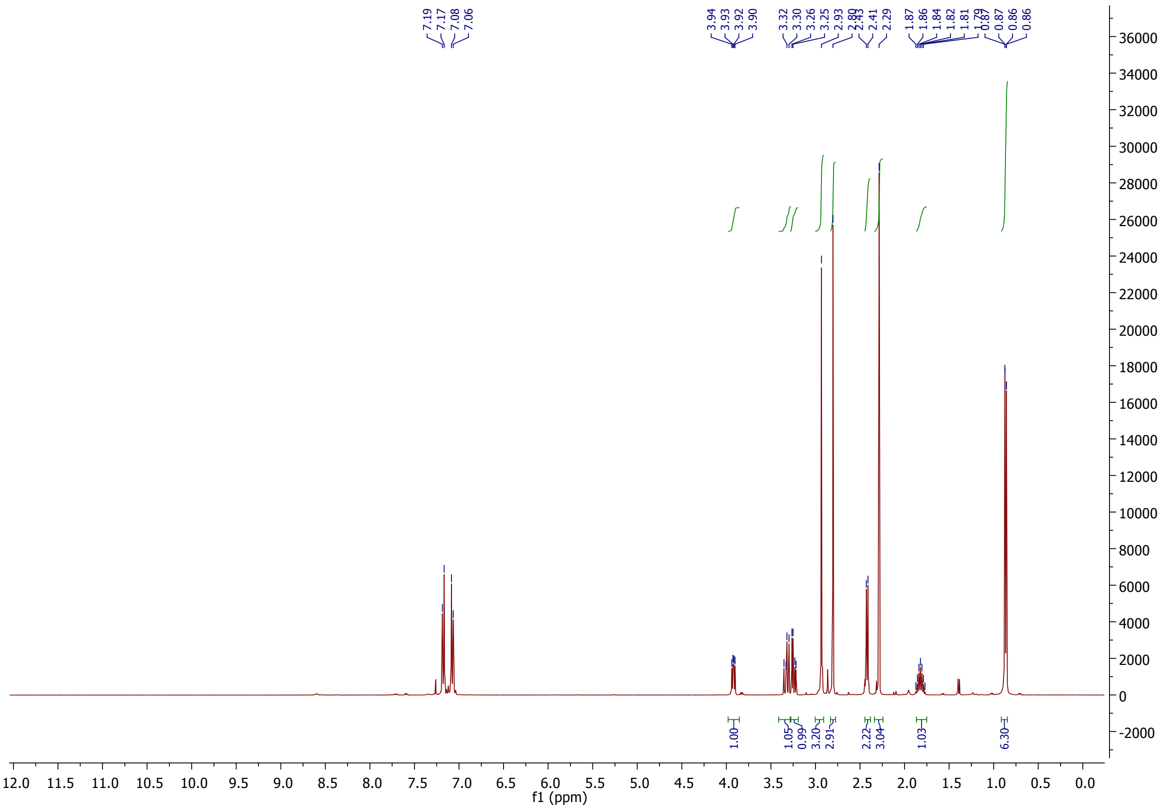

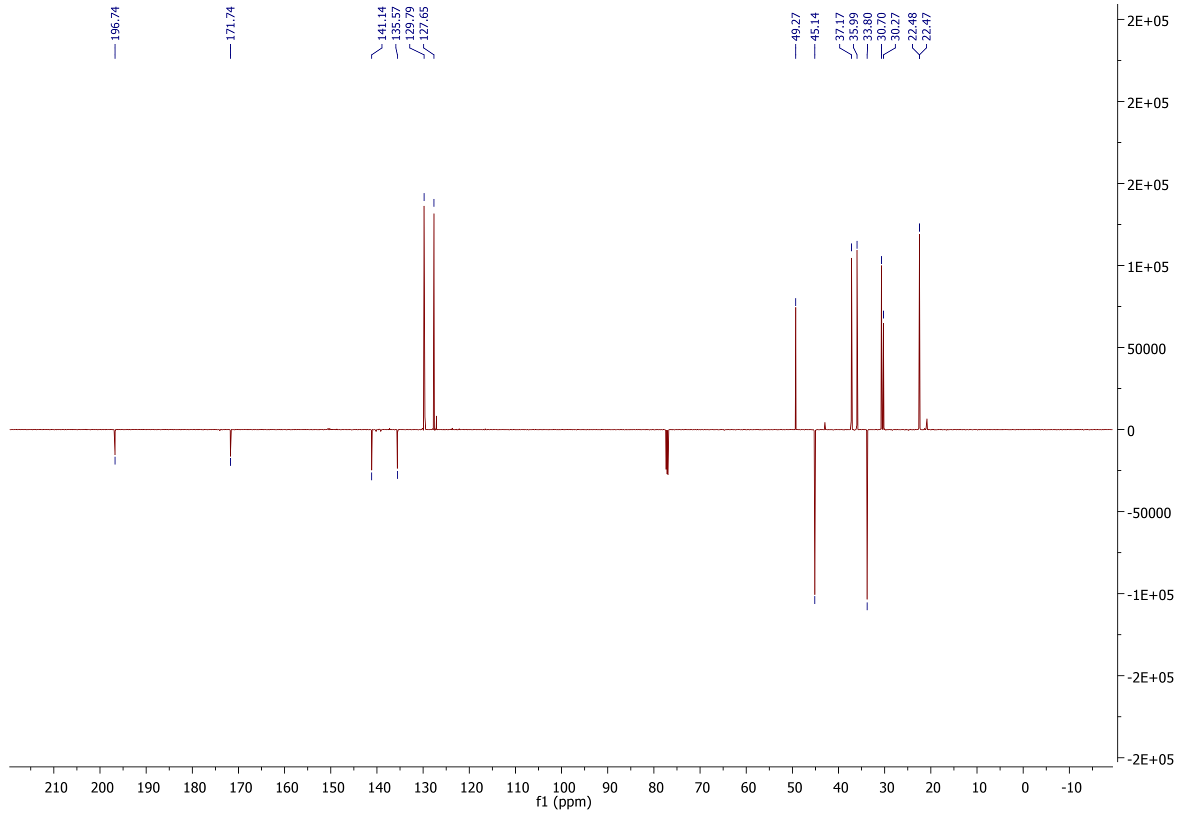

Compound **8s**

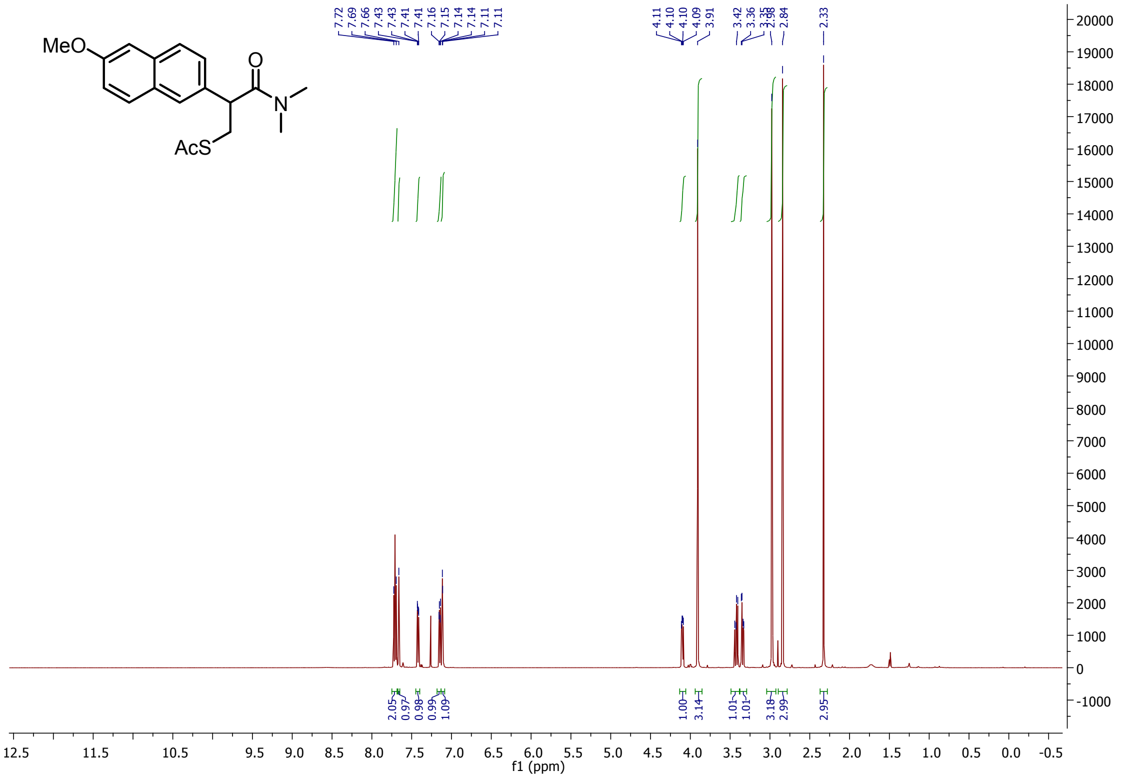

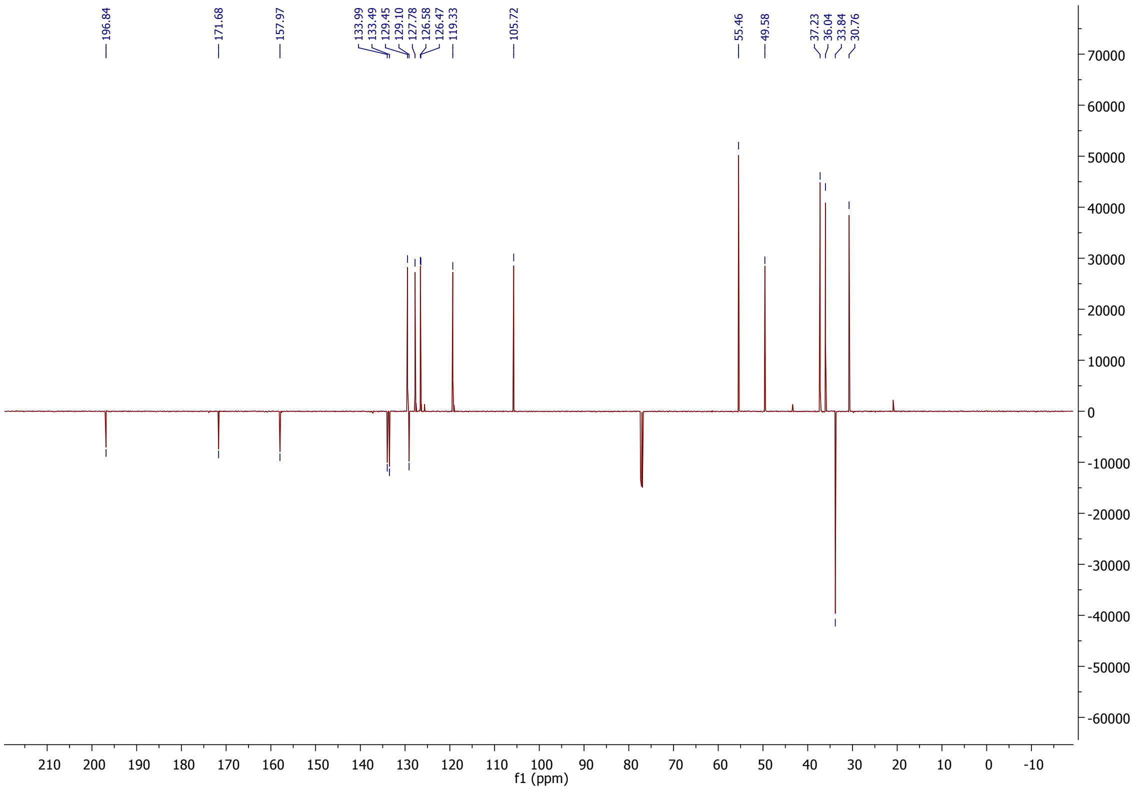
196.84
171.68
157.97
133.99
133.49
129.45
129.10
127.78
126.58
126.47
119.33
105.72
55.46
49.58
37.23
36.04
33.84
30.76
f1 (ppm)

Compound **8t**

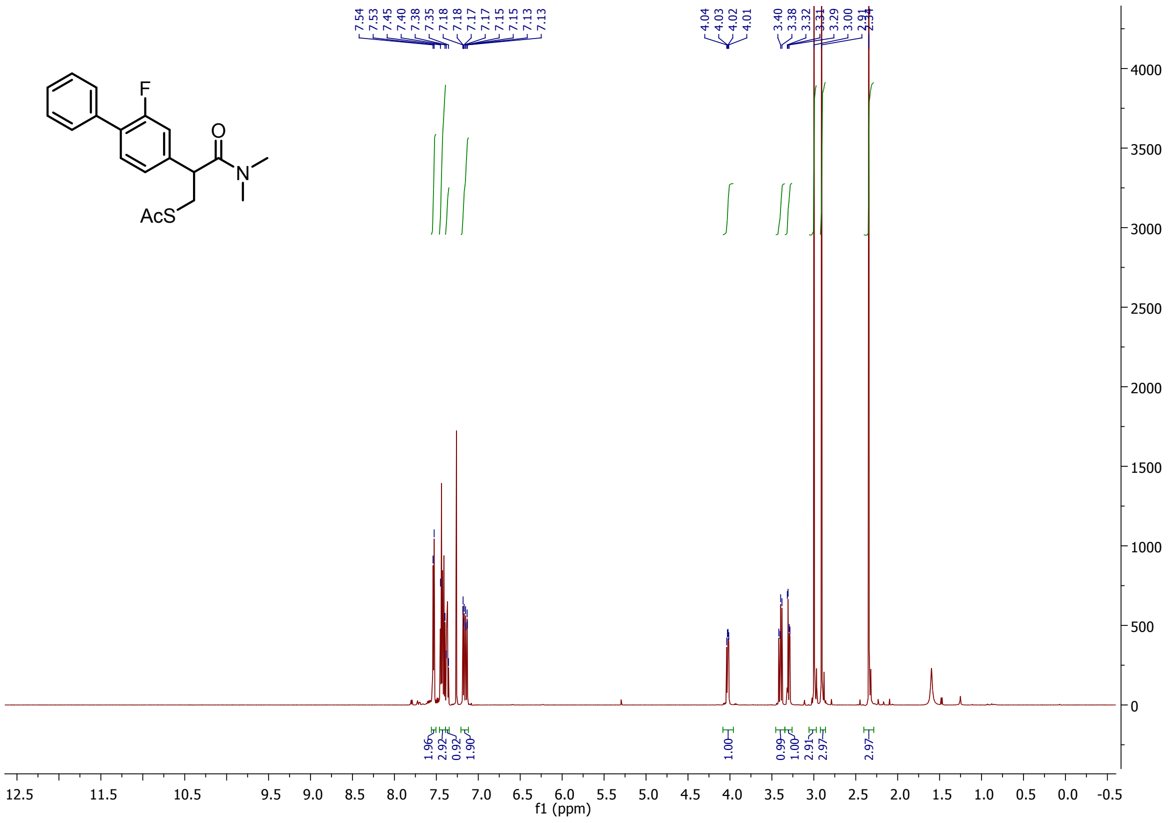

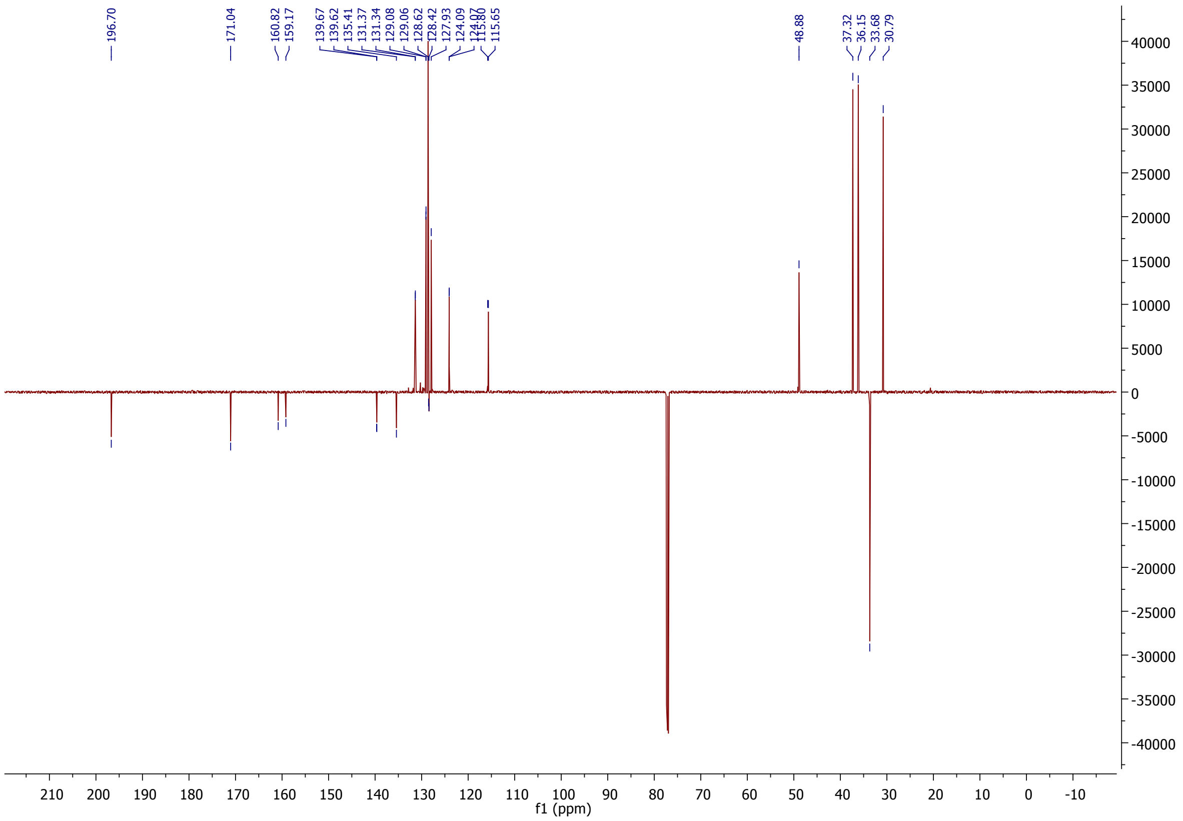
196.70
171.04
160.82
159.17
139.67
139.62
135.41
131.37
131.34
129.08
129.06
128.62
128.42
127.93
124.09
124.07
115.80
115.65
48.88
37.32
36.15
33.68
30.79
f1 (ppm)

Compound **8u**

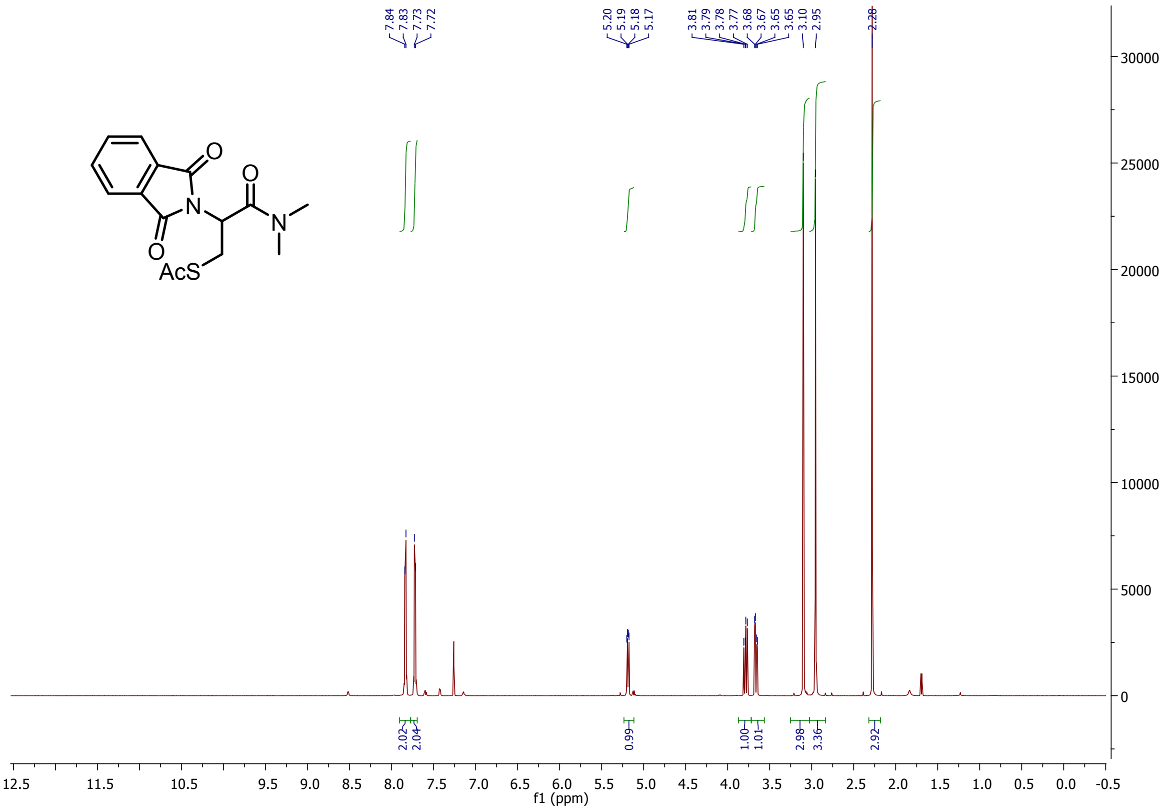
AcS
7.84
7.83
7.73
7.72
5.20
5.19
5.18
5.17
3.81
3.79
3.78
3.77
3.68
3.67
3.65
3.65
3.10
2.95
2.28
2.02
2.04
0.99
1.00
1.01
2.98
3.36
2.92
f1 (ppm)

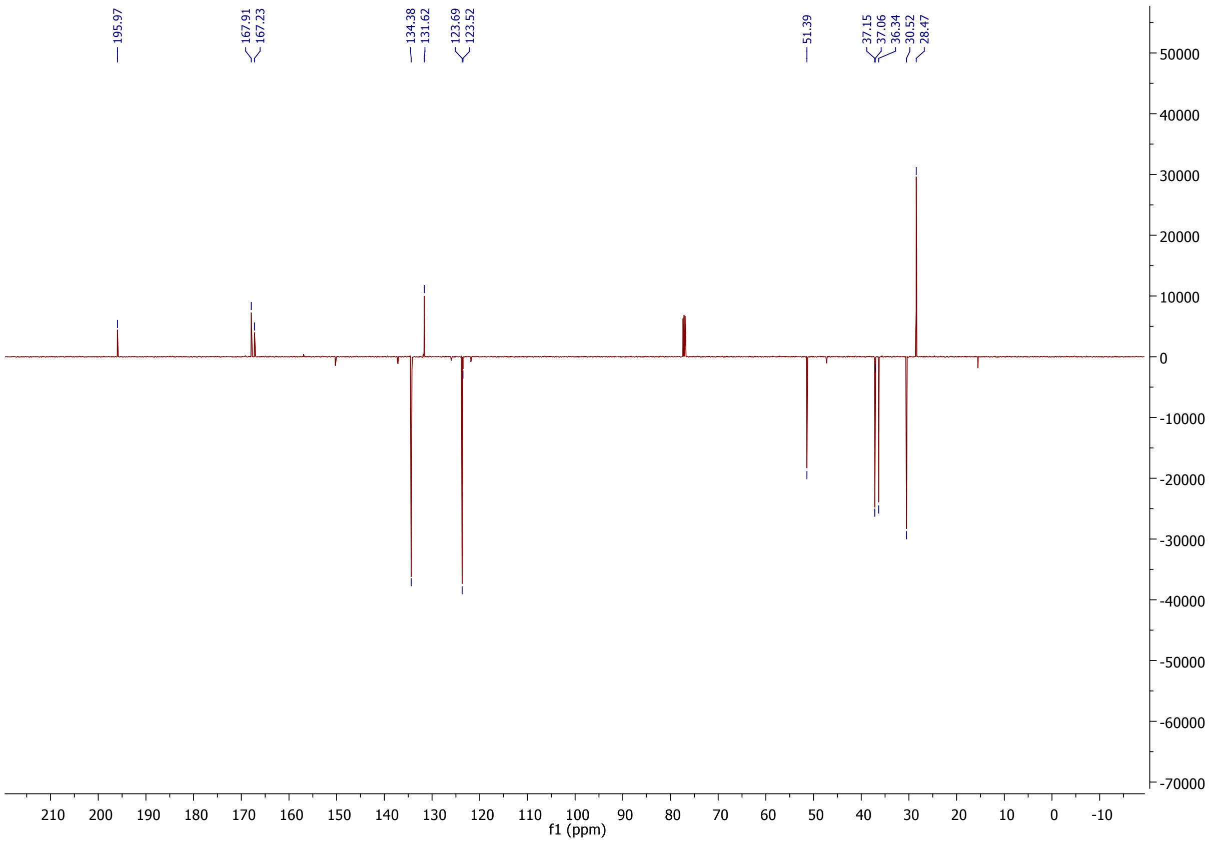
195.97
167.91
167.23
134.38
131.62
123.69
123.52
51.39
37.15
37.06
36.34
30.52
28.47
50000
40000
30000
20000
10000
0
-10000
-20000
-30000
-40000
-50000
-60000
-70000
210 200 190 180 170 160 150 140 130 120 110 100 90 80 70 60 50 40 30 20 10 0 -10
f1 (ppm)

Compound **8v**

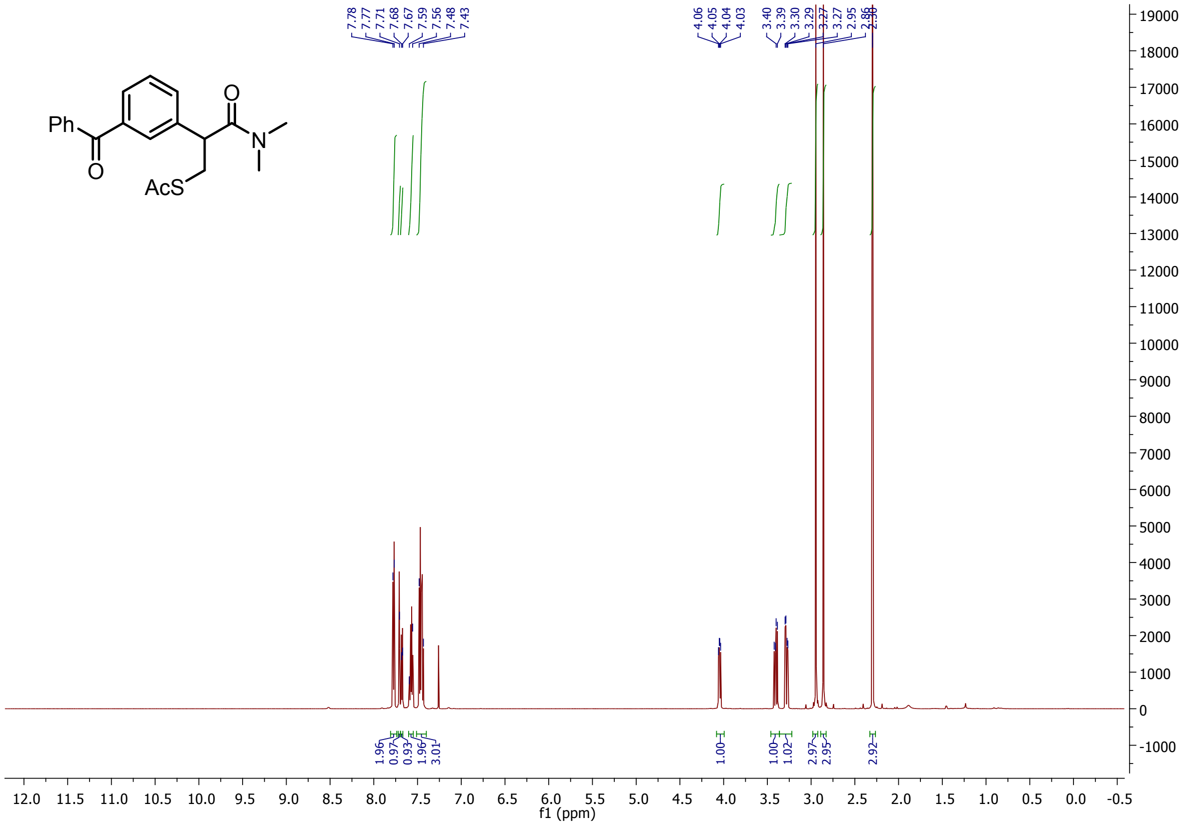

Ph
O
O
N
AcS
f1 (ppm)

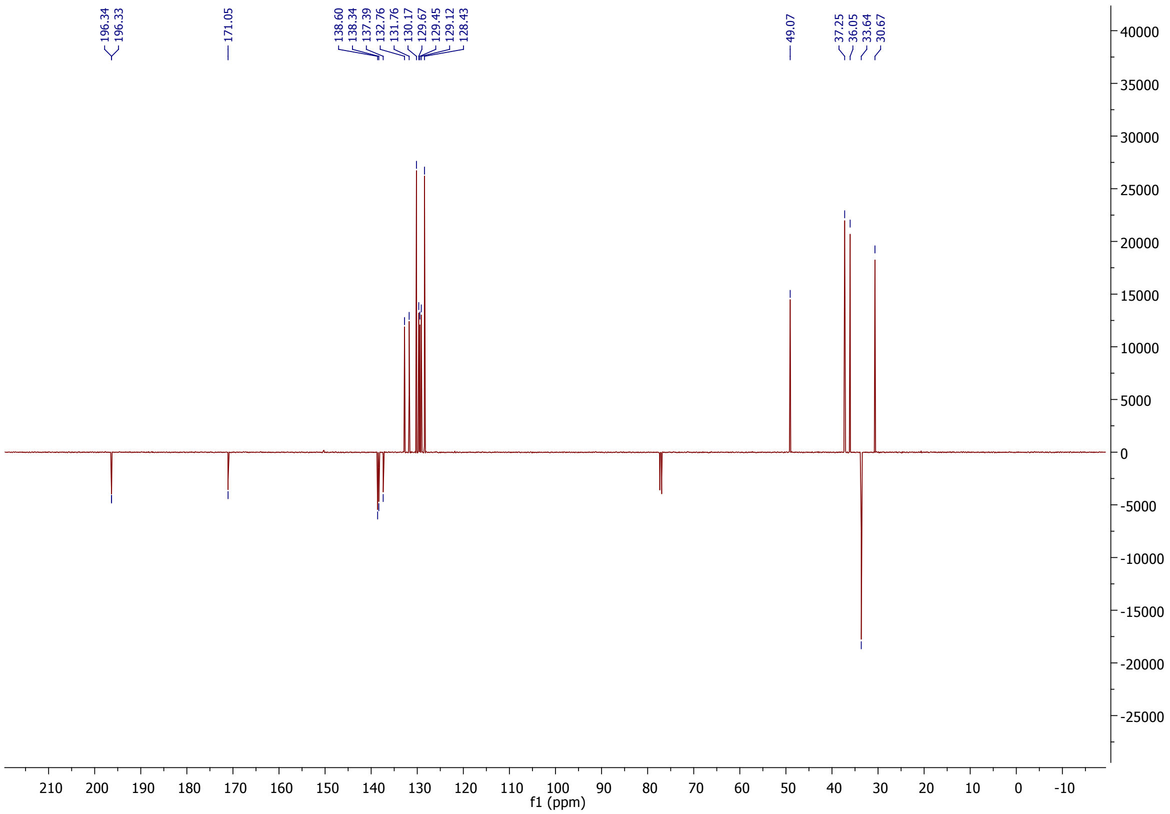
196.34
196.33
171.05
138.60
138.34
137.39
132.76
131.76
130.17
129.67
129.45
129.12
128.43
49.07
37.25
36.05
33.64
30.67
40000
35000
30000
25000
20000
15000
10000
5000
0
-5000
-10000
-15000
-20000
-25000
210
200
190
180
170
160
150
140
130
120
110
100
90
80
70
60
50
40
30
20
10
0
-10
f1 (ppm)

Compound **8w**

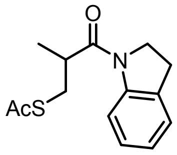

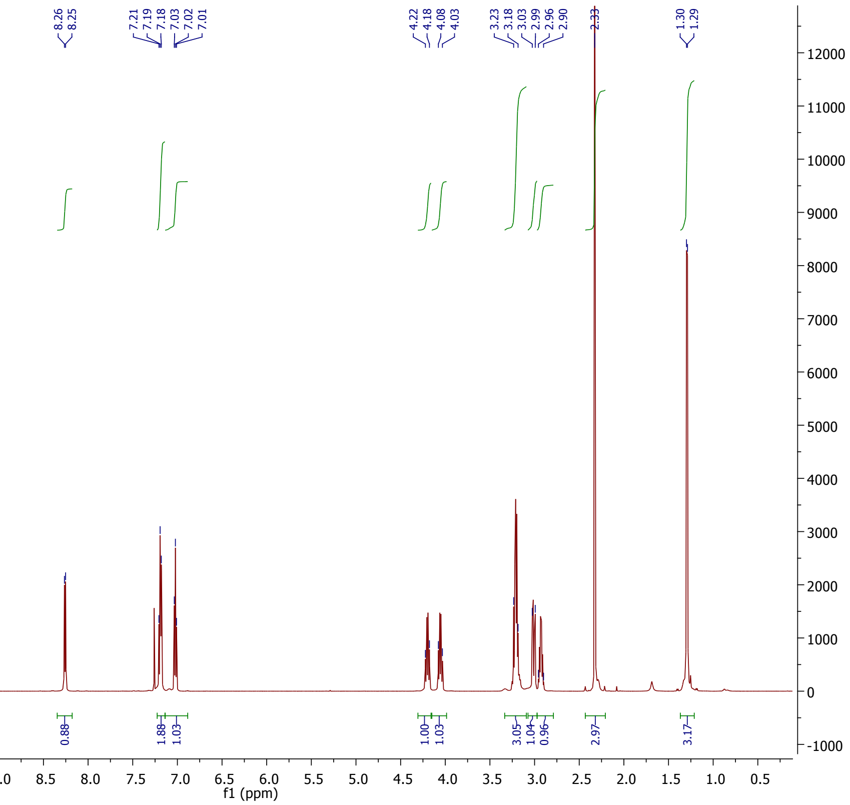

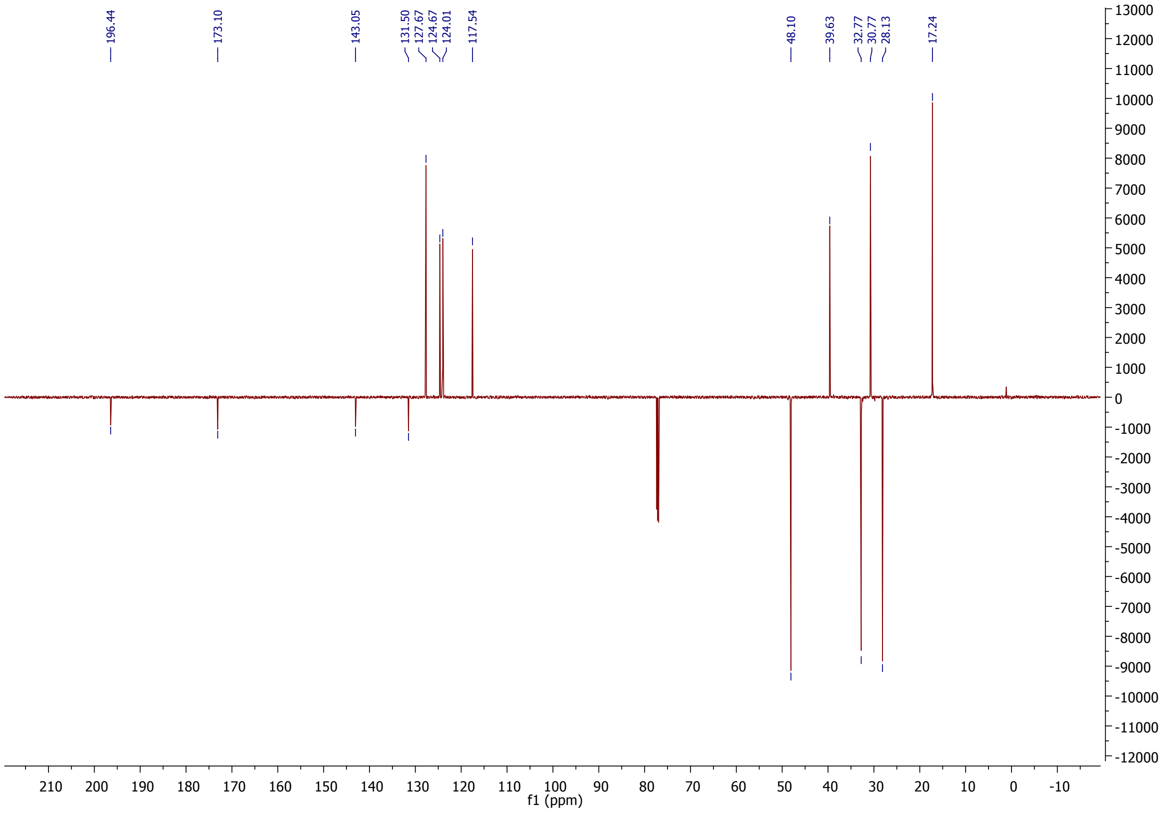

Compound **9a**

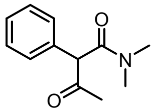

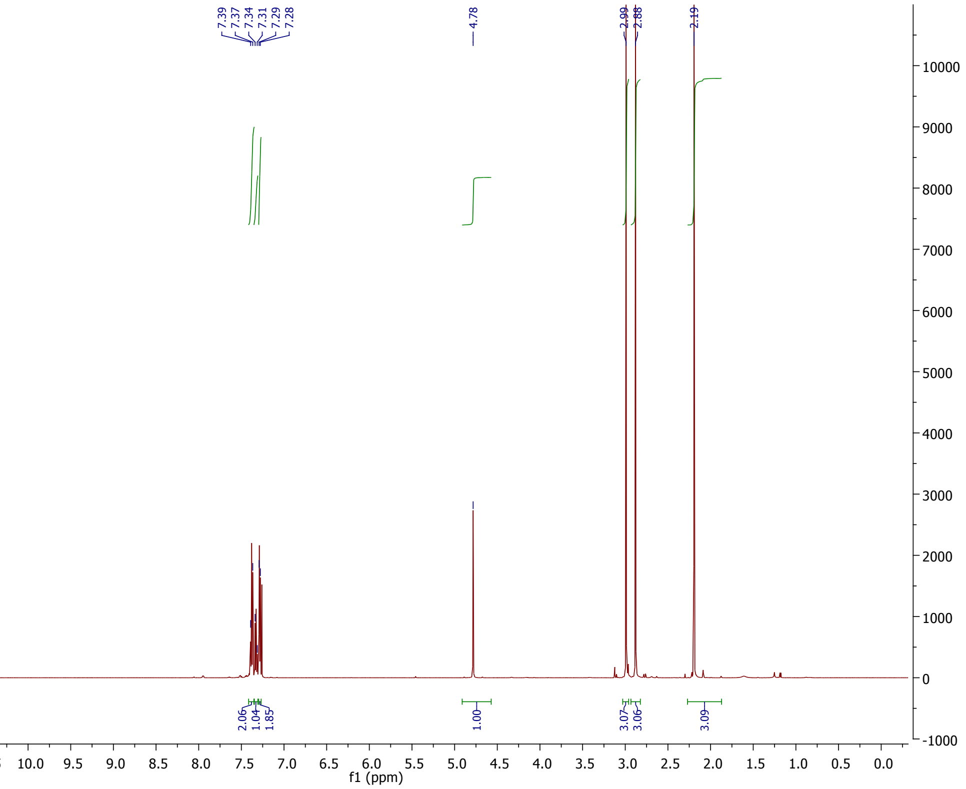
7.39
7.37
7.34
7.31
7.29
7.28
4.78
2.99
2.88
2.19
2.06
1.04
1.85
1.00
3.07
3.06
3.09
f1 (ppm)

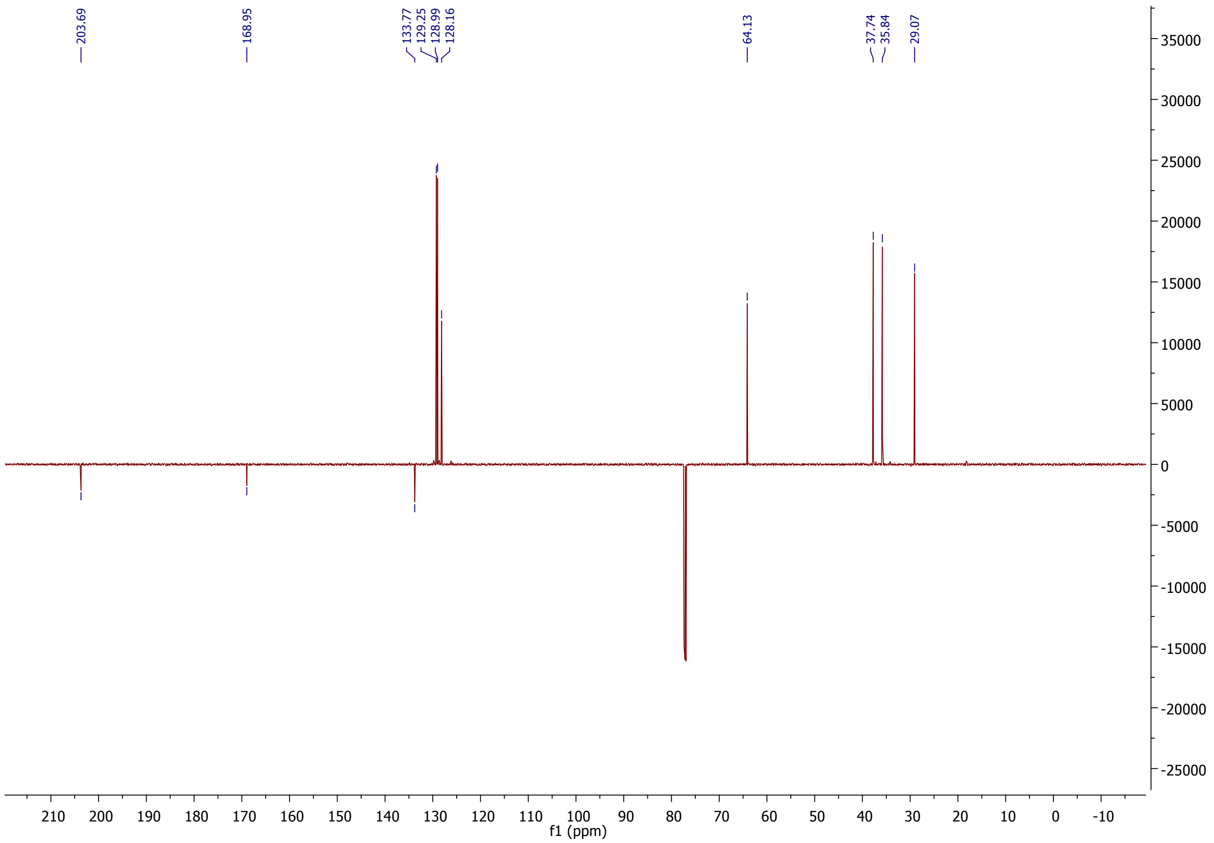

Compound **10a**

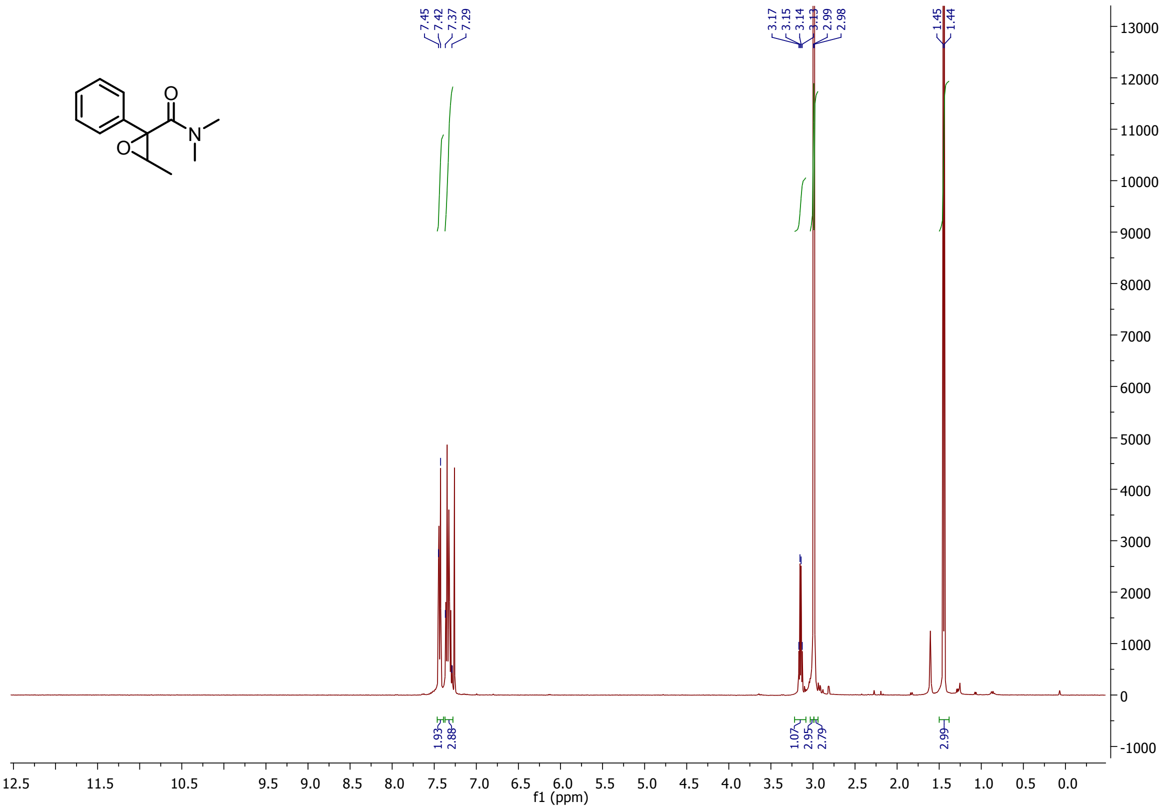
7.45
7.42
7.37
7.29
3.17
3.15
3.14
3.13
2.99
2.98
1.45
1.44
1.93
2.88
1.07
2.95
2.79
2.99
f1 (ppm)

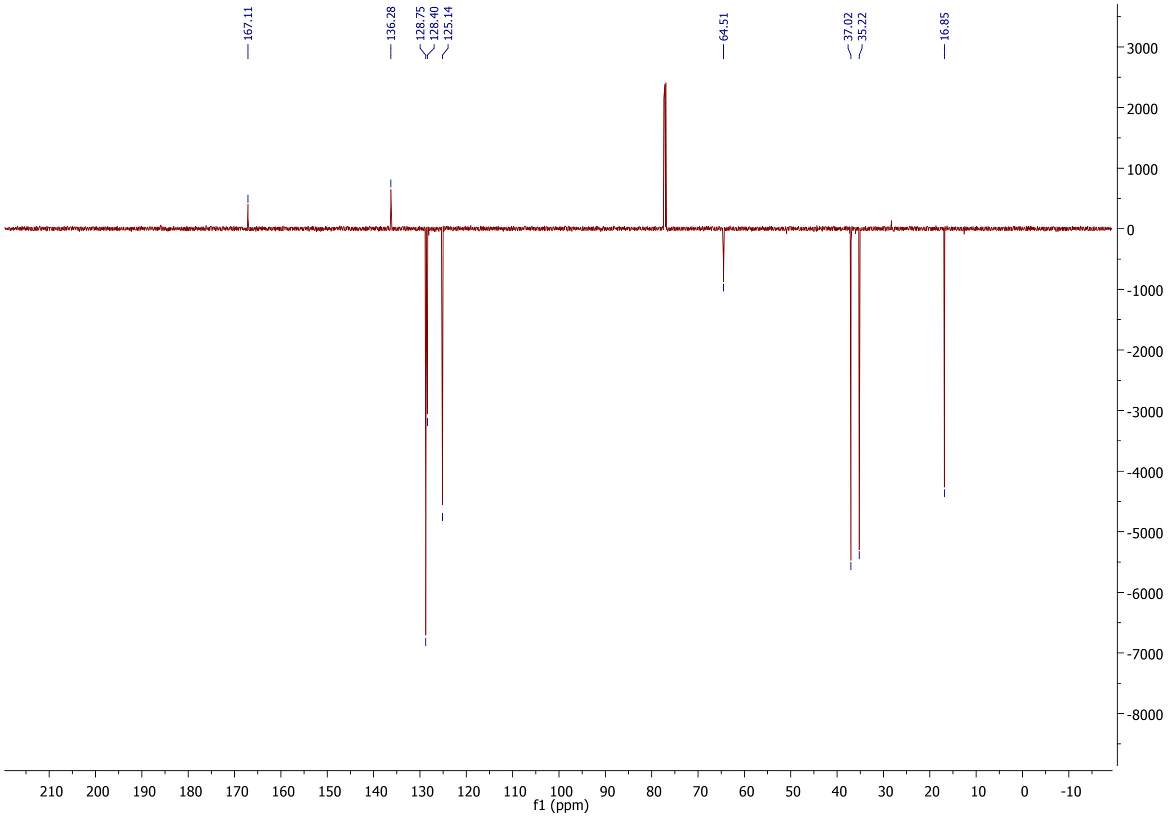

Compound **10b**

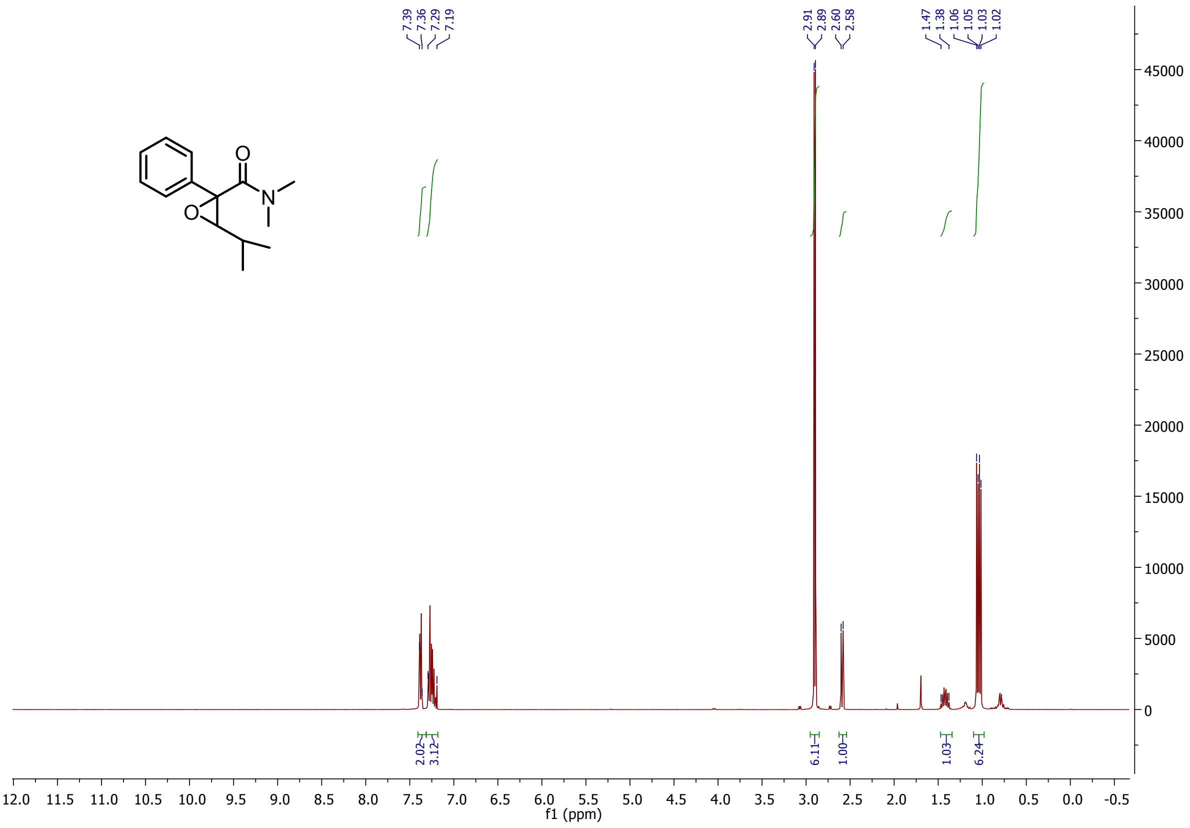
7.39
7.36
7.29
7.19
2.91
2.89
2.60
2.58
1.47
1.38
1.06
1.05
1.03
1.02
45000
40000
35000
30000
25000
20000
15000
10000
5000
0
2.02
3.12
6.11
1.00
1.03
6.24
12.0
11.5
11.0
10.5
10.0
9.5
9.0
8.5
8.0
7.5
7.0
6.5
6.0
5.5
5.0
4.5
4.0
3.5
3.0
2.5
2.0
1.5
1.0
0.5
0.0
-0.5
f1 (ppm)
O
N
O

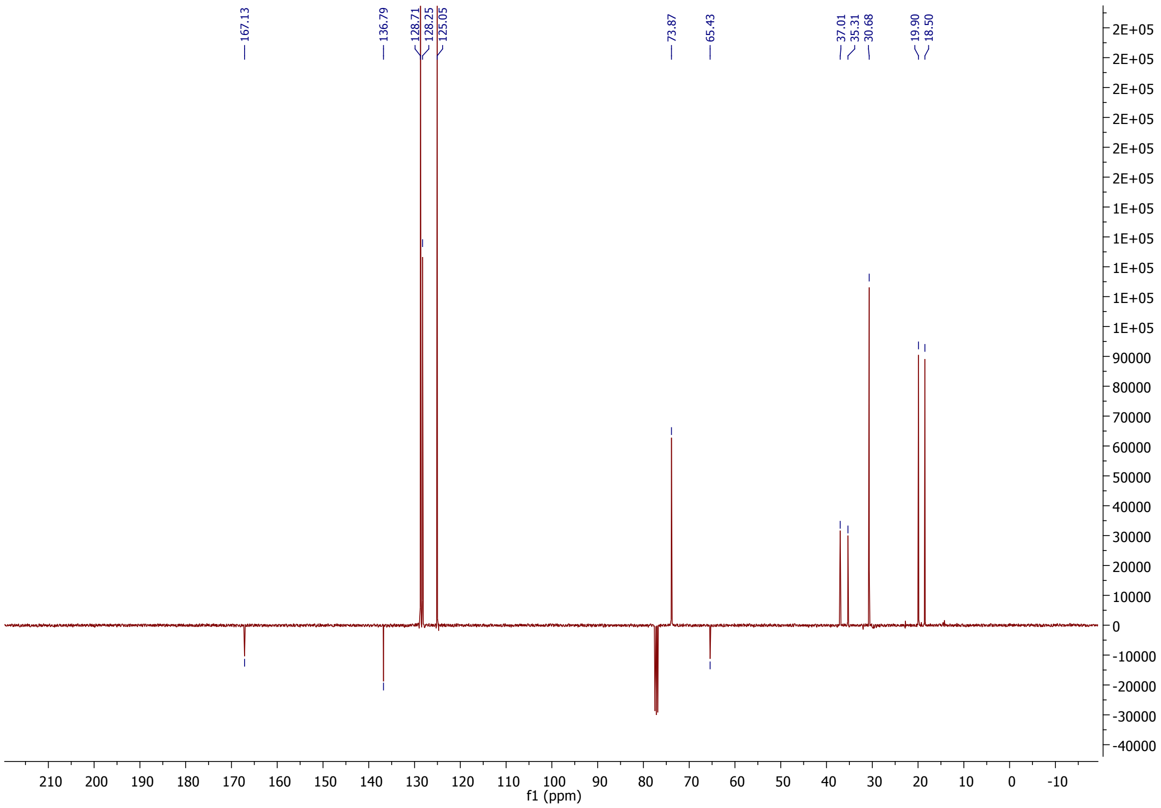

Compound **10c**

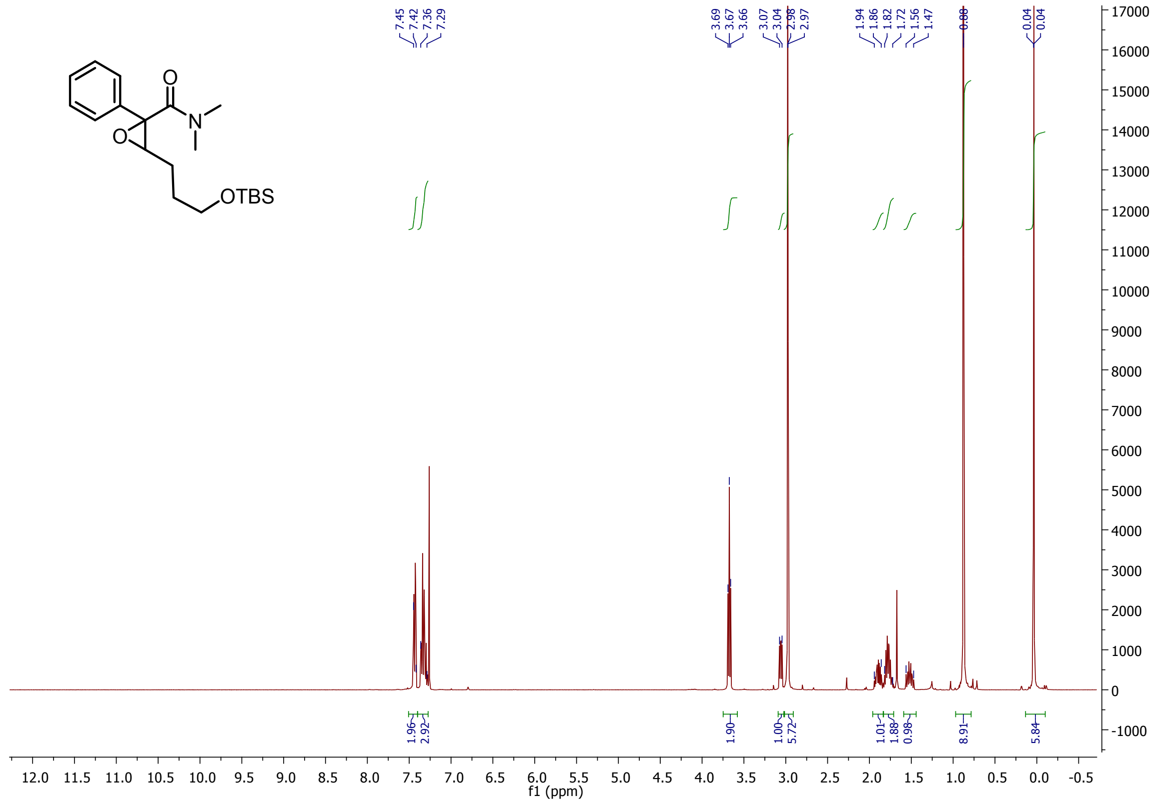
O
N
O
OTBS
7.45
7.42
7.36
7.29
3.69
3.67
3.66
3.07
3.04
2.98
2.97
1.94
1.86
1.82
1.72
1.56
1.47
0.88
0.04
0.04
1.96
2.92
1.90
1.00
5.72
1.01
1.88
0.98
8.91
5.84
f1 (ppm)

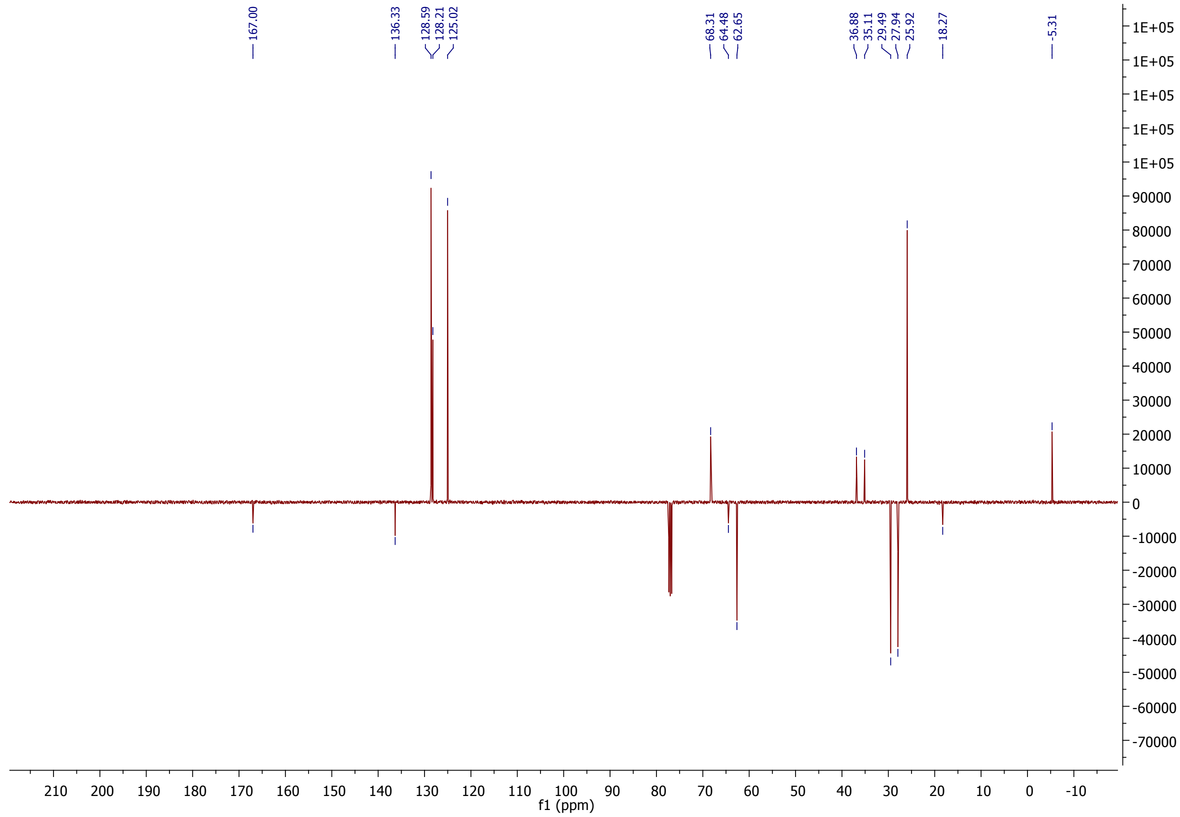
167.00
136.33
128.59
128.21
125.02
68.31
64.48
62.65
36.88
35.11
29.49
27.94
25.92
18.27
-5.31
f1 (ppm)

Compound **10d**

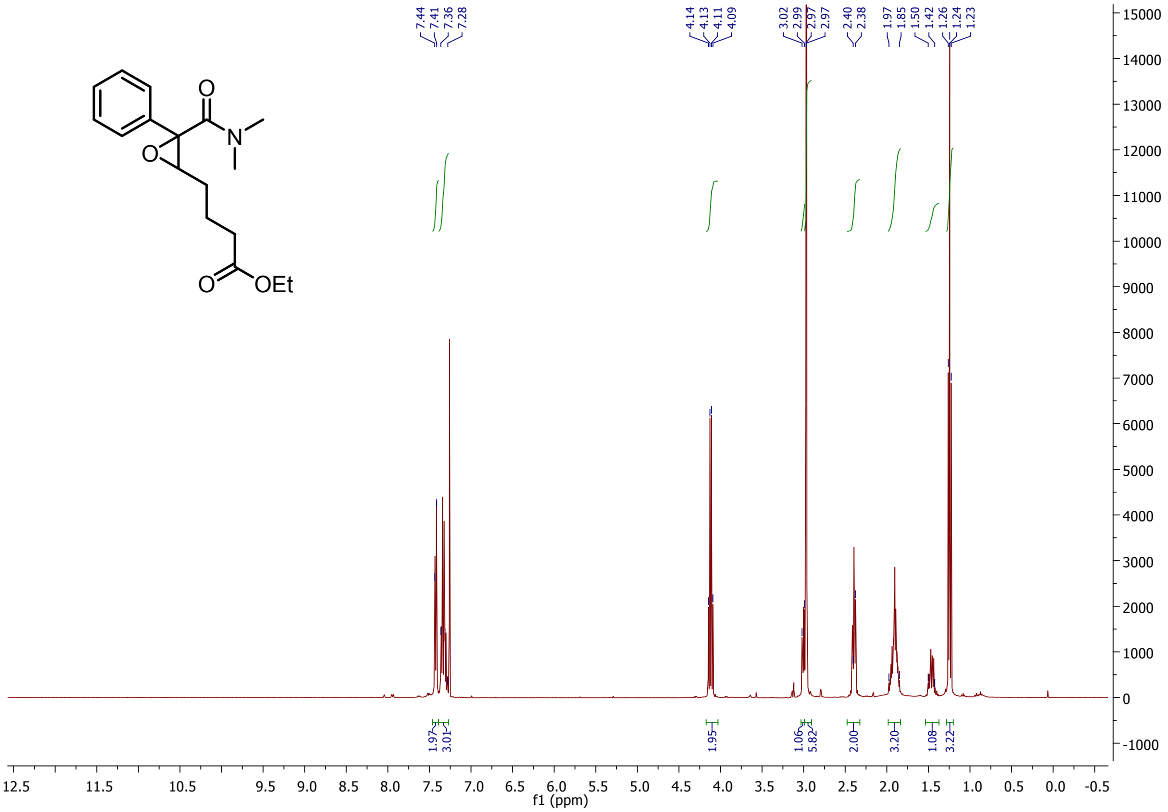

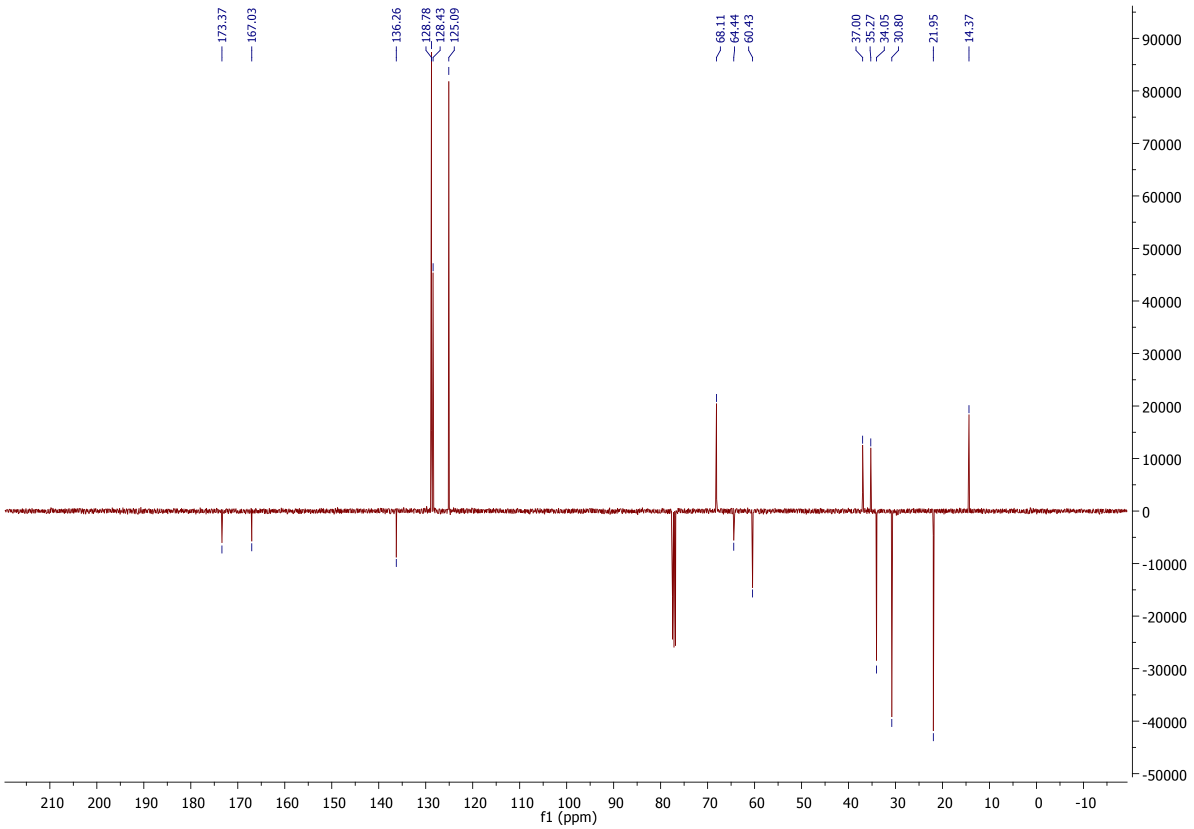

Compound **10e**

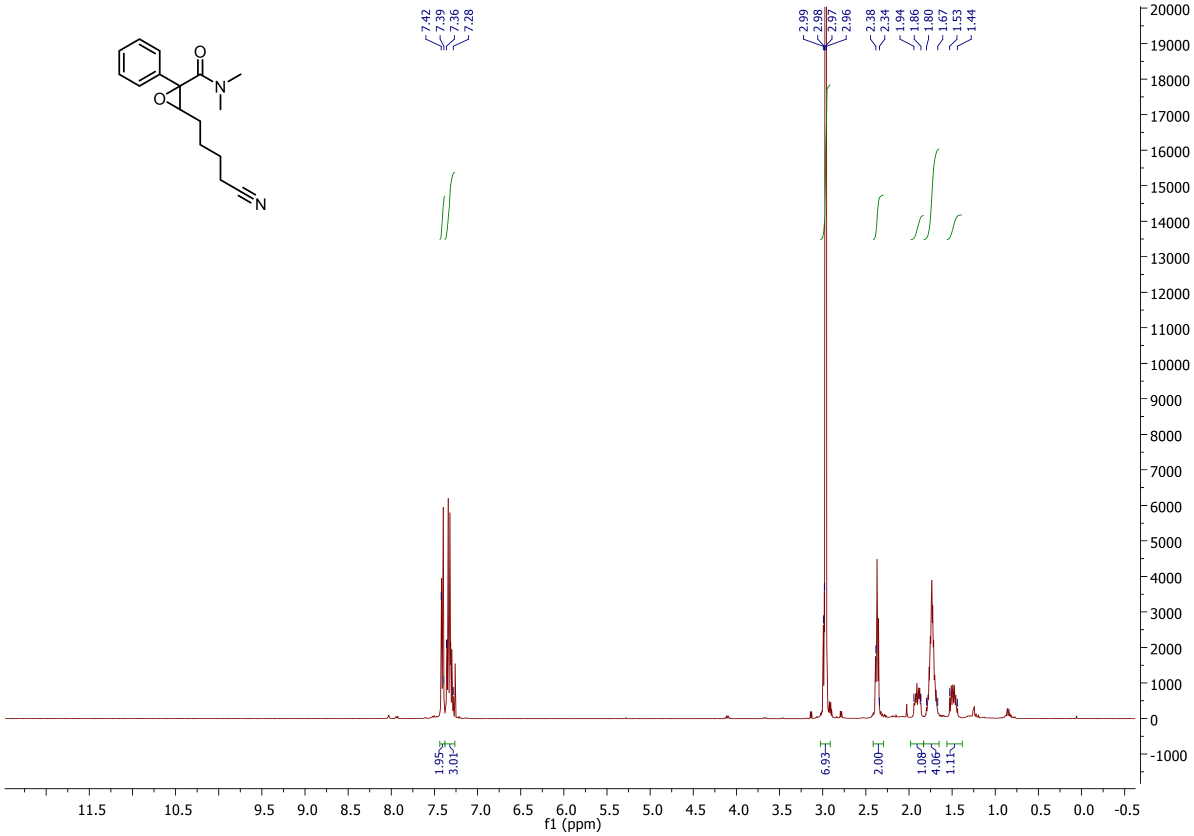
7.42
7.39
7.36
7.28
2.99
2.98
2.97
2.96
2.38
2.34
1.94
1.86
1.80
1.67
1.53
1.44
1.95
3.01
6.93
2.00
1.08
4.06
1.11
f1 (ppm)

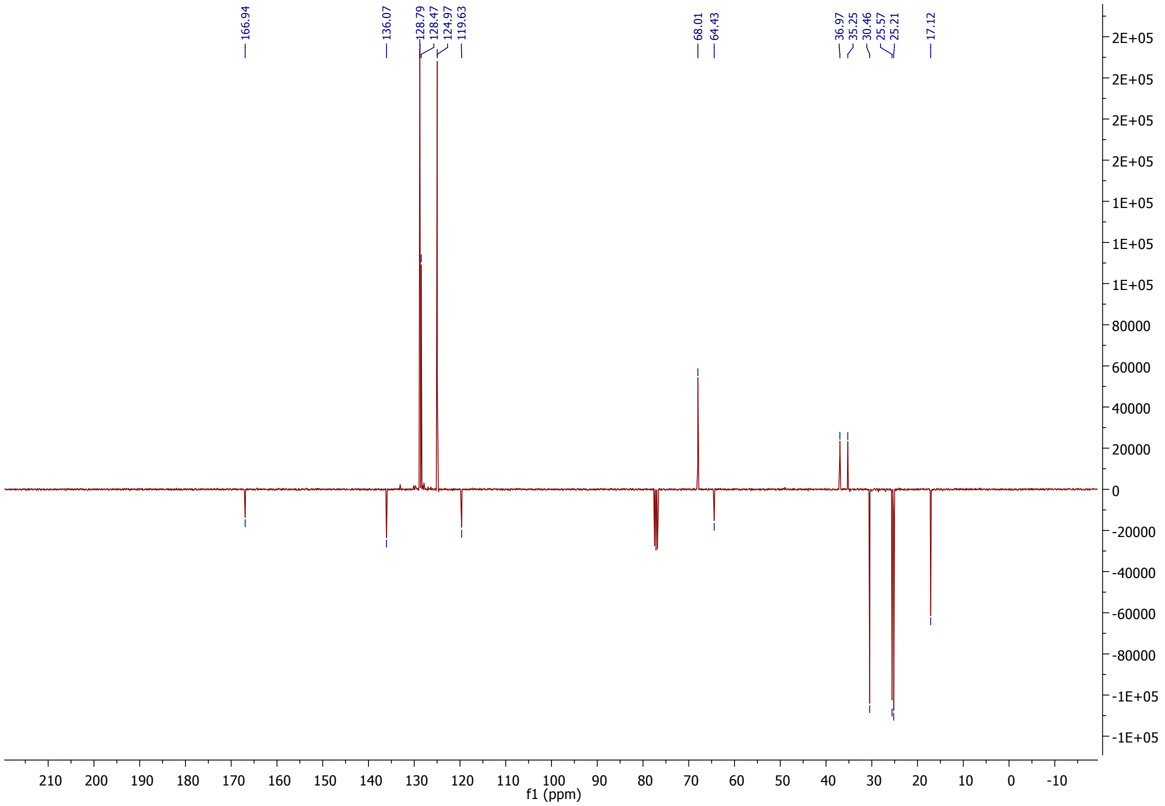

Compound **10f**

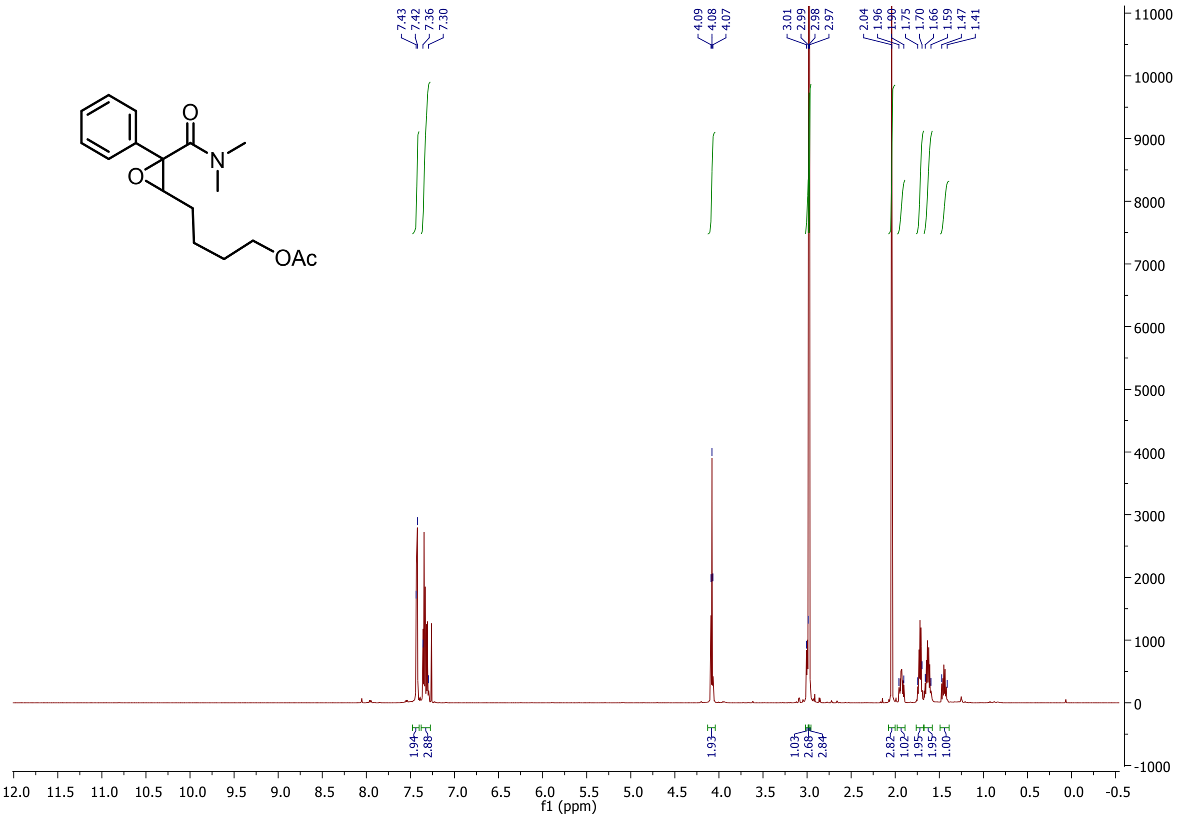
OAc
O
N
f1 (ppm)

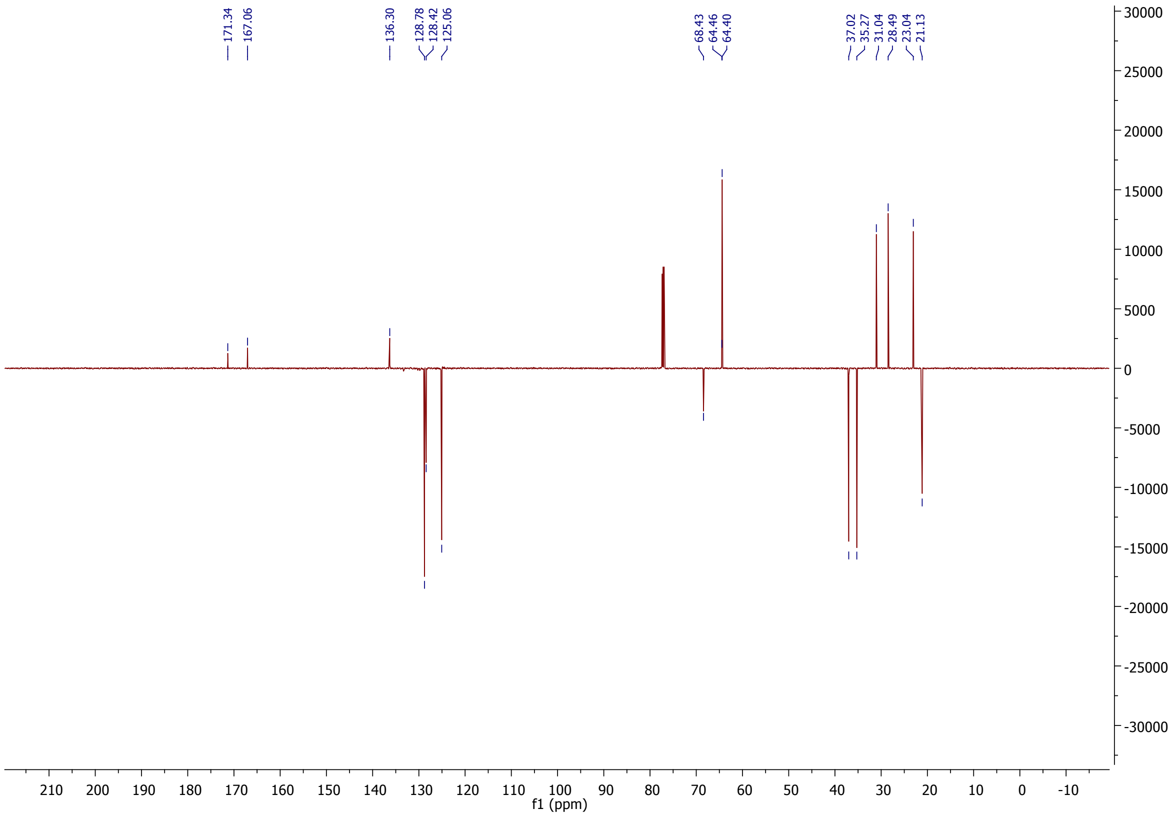

Compound **10g**

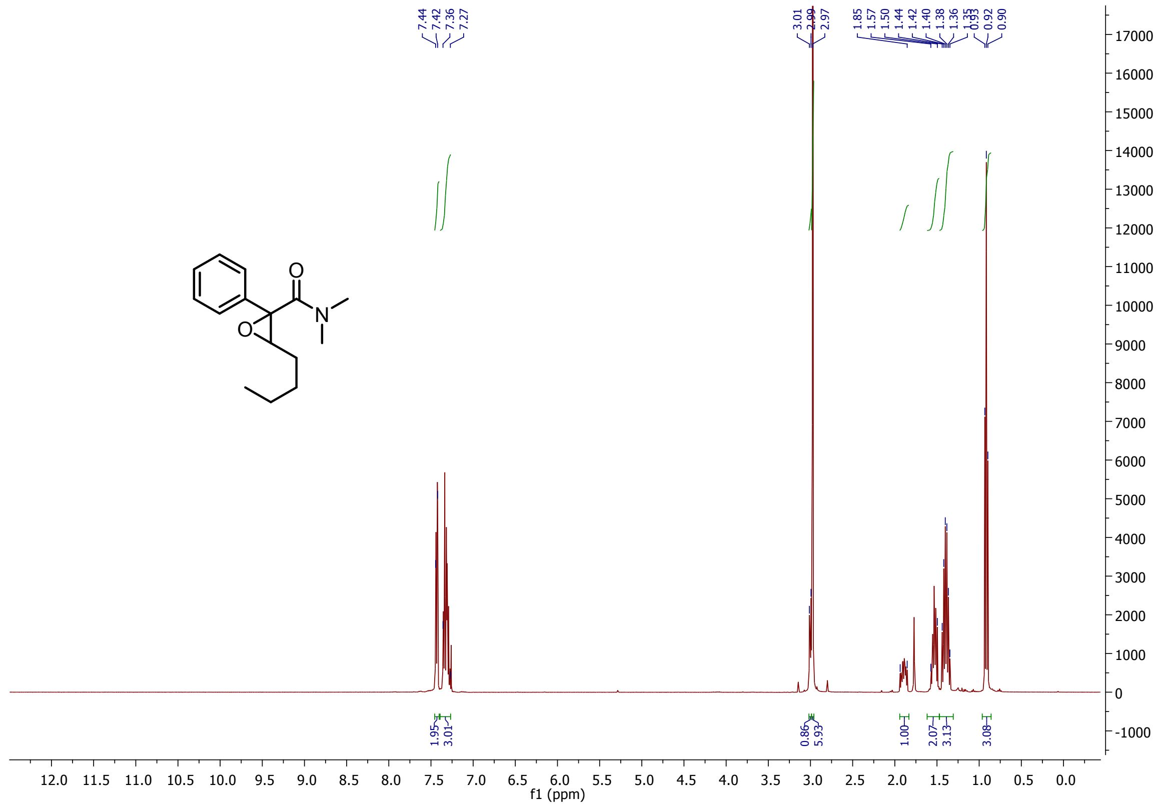
7.44
7.42
7.36
7.27
3.01
2.99
2.97
1.85
1.57
1.50
1.44
1.42
1.40
1.38
1.36
1.35
0.93
0.92
0.90
1.95
3.01
0.86
5.93
1.00
2.07
3.13
3.08
O
N
O
f1 (ppm)

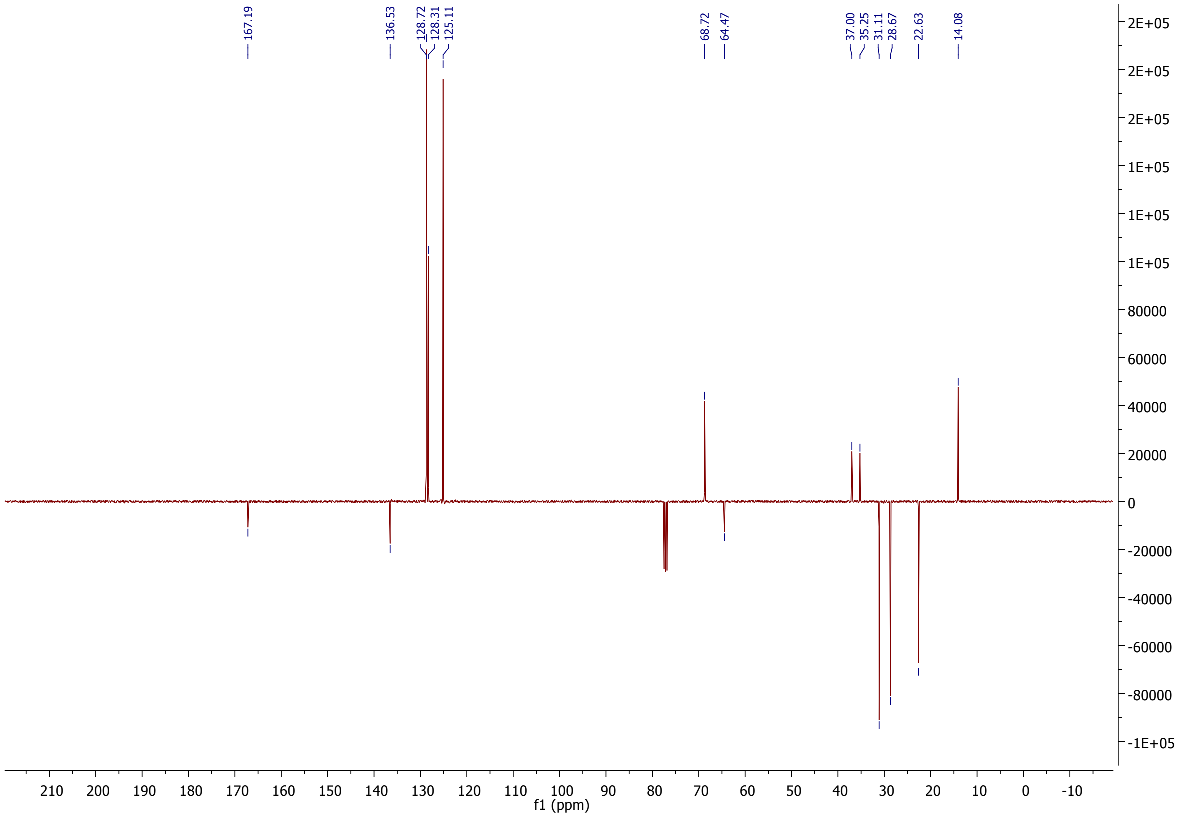

Compound **10h**

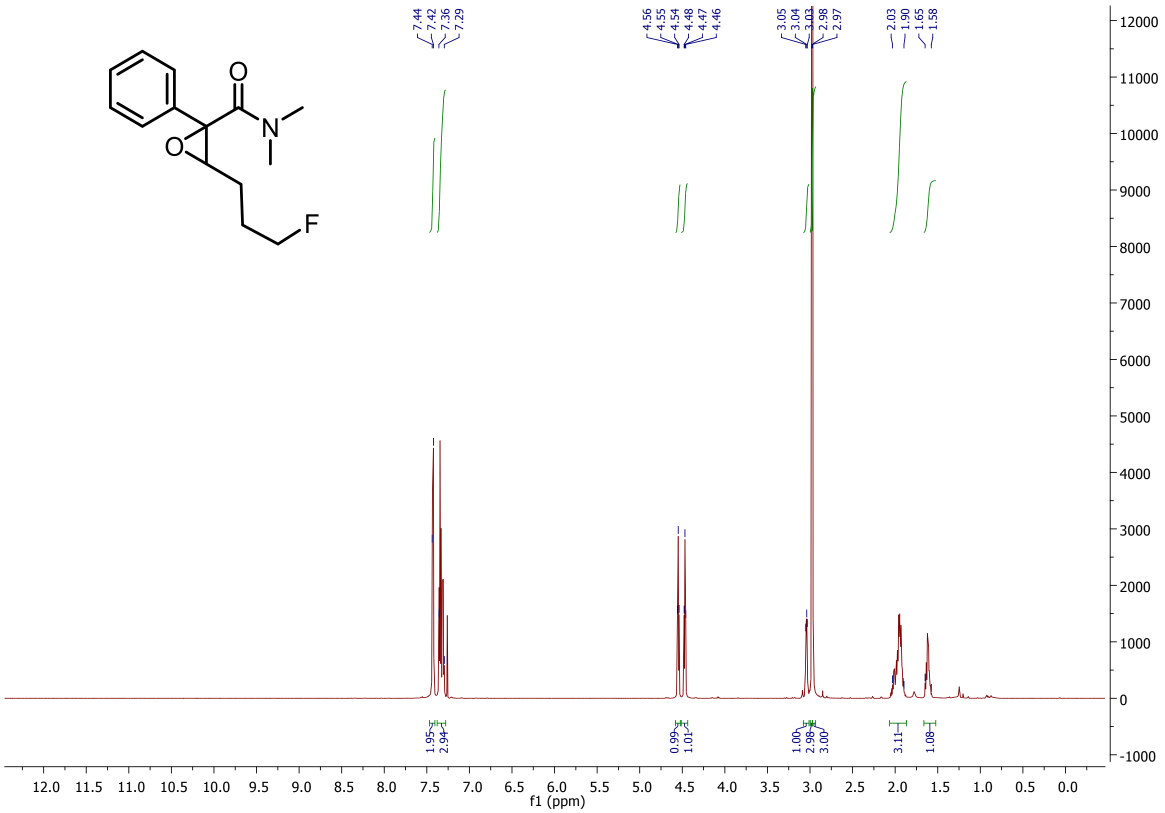
O
N
O
F
7.44
7.42
7.36
7.29
4.56
4.55
4.54
4.48
4.47
4.46
3.05
3.04
3.02
2.98
2.97
2.03
1.90
1.65
1.58
1.95
2.94
0.99
1.01
1.00
2.98
3.00
3.11
1.08
12000
11000
10000
9000
8000
7000
6000
5000
4000
3000
2000
1000
0
-1000
12.0 11.5 11.0 10.5 10.0 9.5 9.0 8.5 8.0 7.5 7.0 6.5 6.0 5.5 5.0 4.5 4.0 3.5 3.0 2.5 2.0 1.5 1.0 0.5 0.0
f1 (ppm)

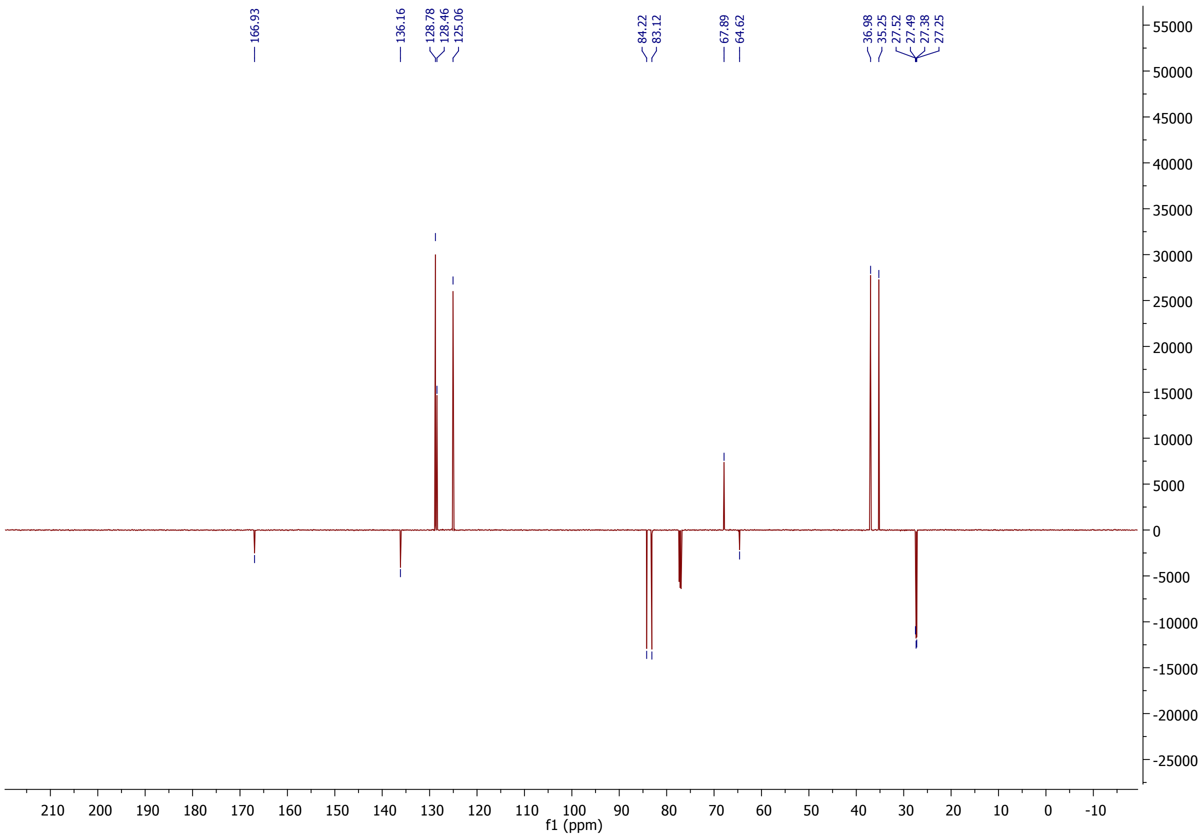

Compound **10i**

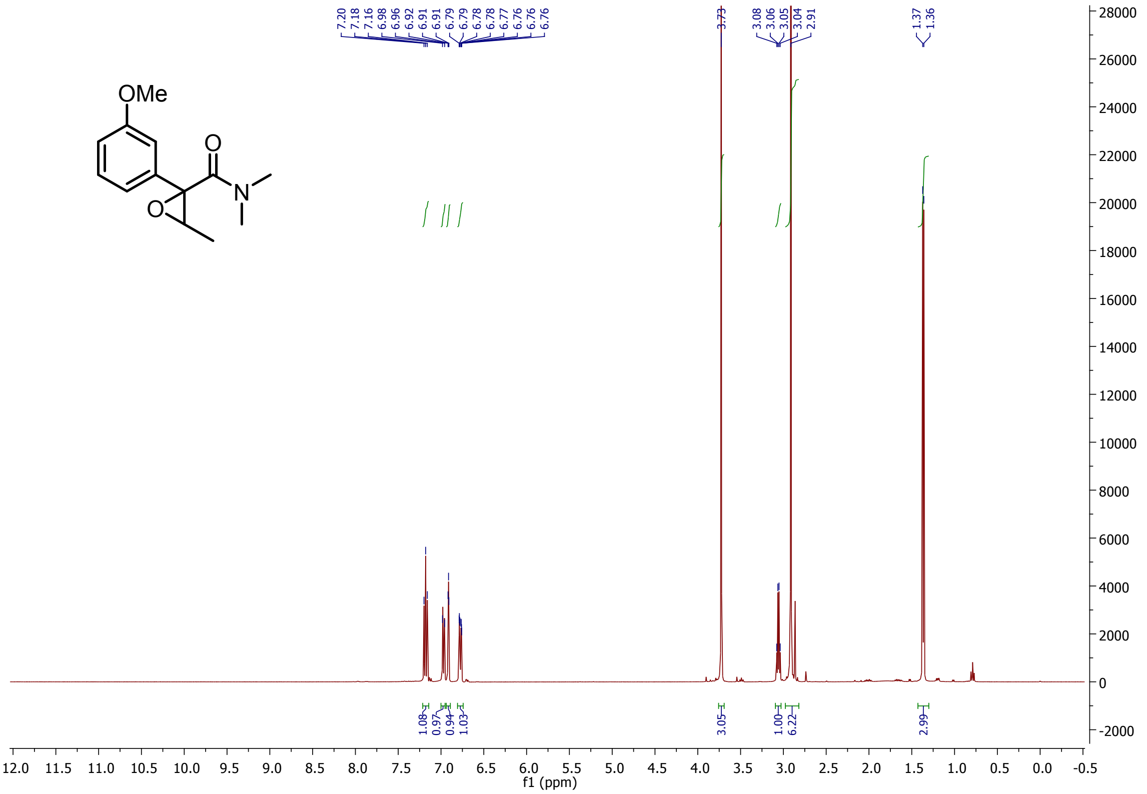
OMe
O
O
N
7.20 7.18 7.16 6.98 6.96 6.92 6.91 6.91 6.79 6.79 6.78 6.78 6.77 6.76 6.76 6.76
3.73
3.08 3.06 3.05 3.04 2.91
1.37 1.36
1.08 0.97 0.94 1.03
3.05
1.00 6.22
2.99
f1 (ppm)

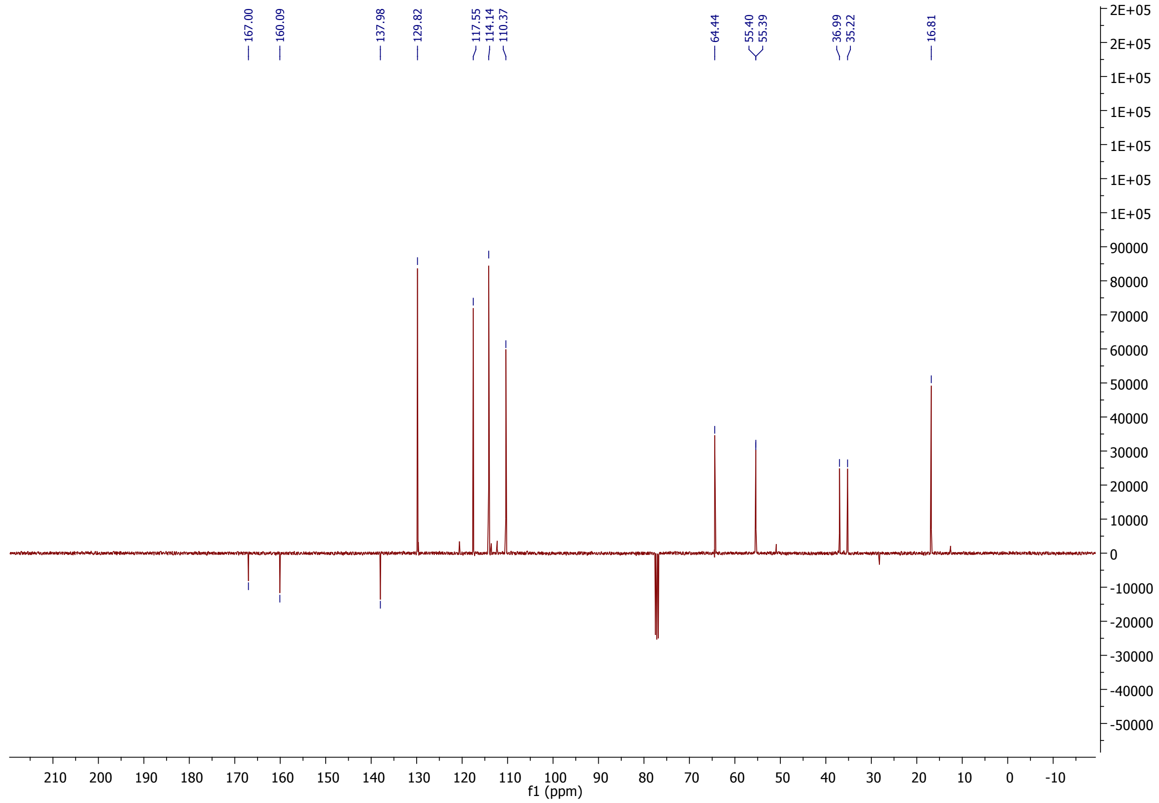
167.00
160.09
137.98
129.82
117.55 114.14 110.37
64.44
55.40 55.39
36.99 35.22
16.81
f1 (ppm)

Compound **10j**

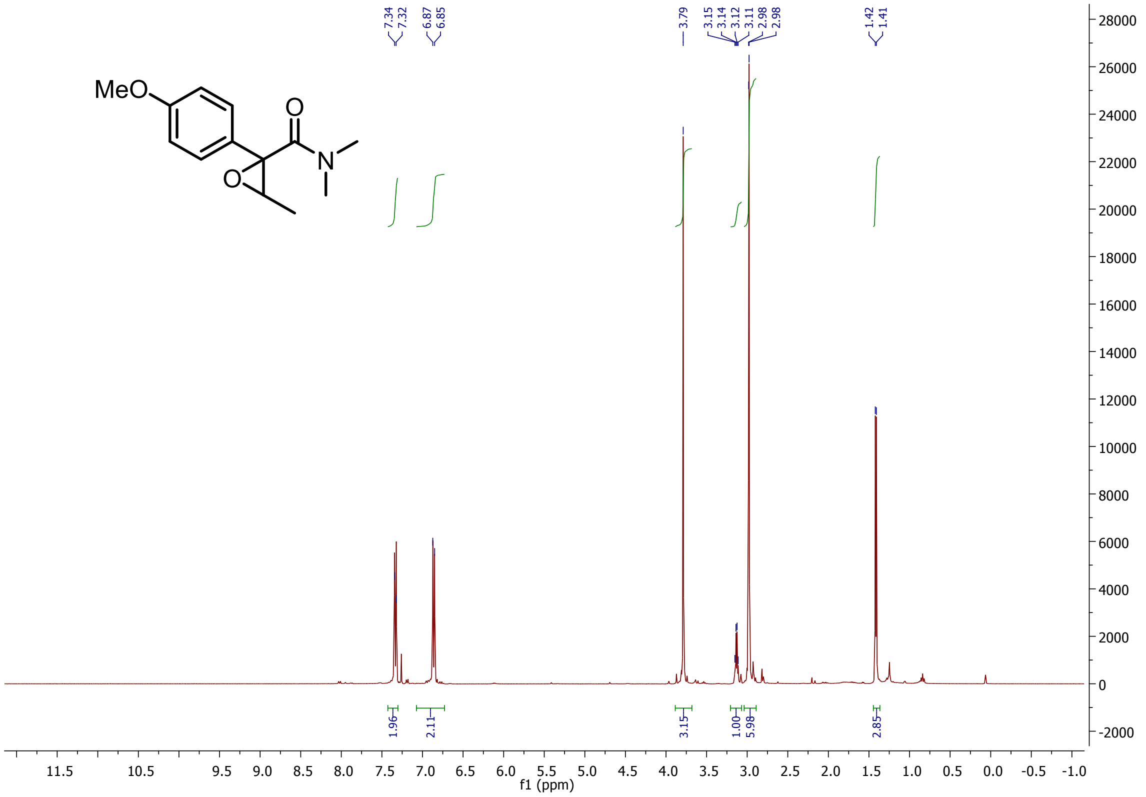

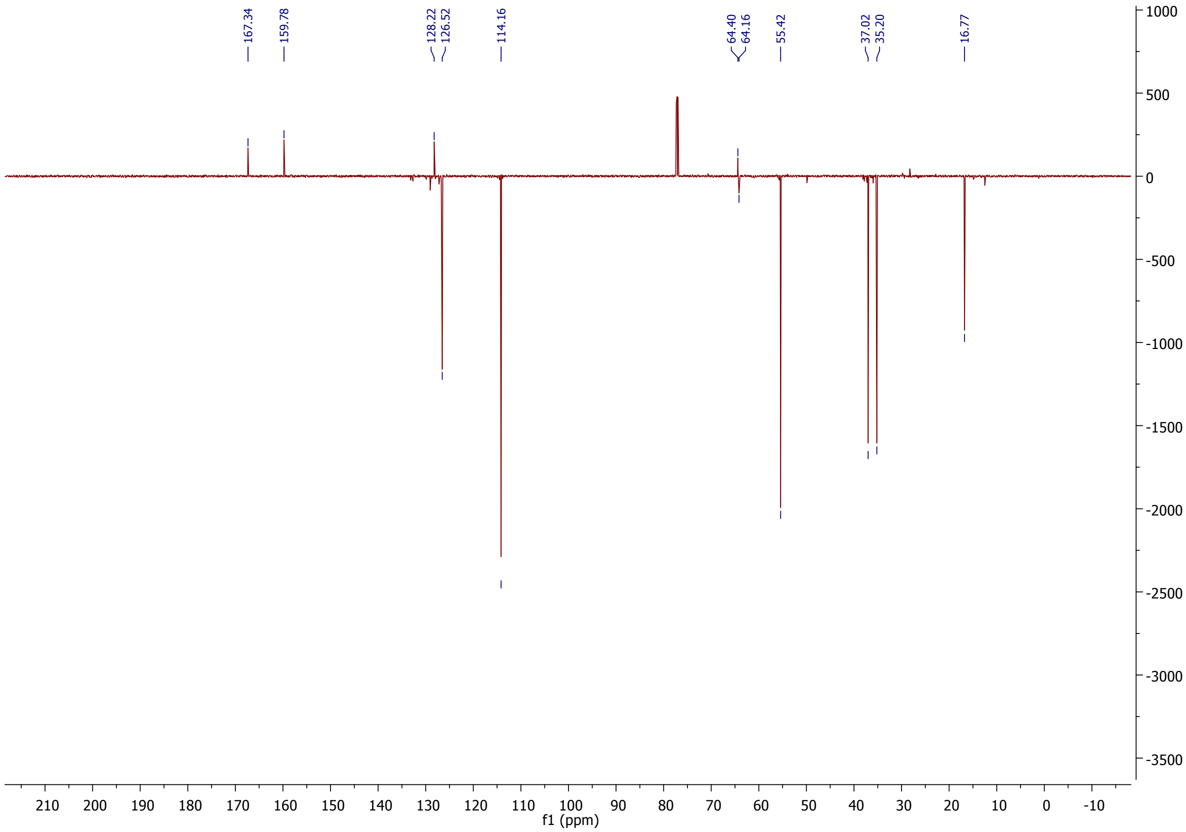
167.34
159.78
128.22
126.52
114.16
64.40
64.16
55.42
37.02
35.20
16.77
1000
500
0
-500
-1000
-1500
-2000
-2500
-3000
-3500
210
200
190
180
170
160
150
140
130
120
110
100
90
80
70
60
50
40
30
20
10
0
-10
f1 (ppm)

Compound **10k**

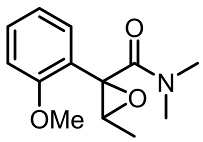

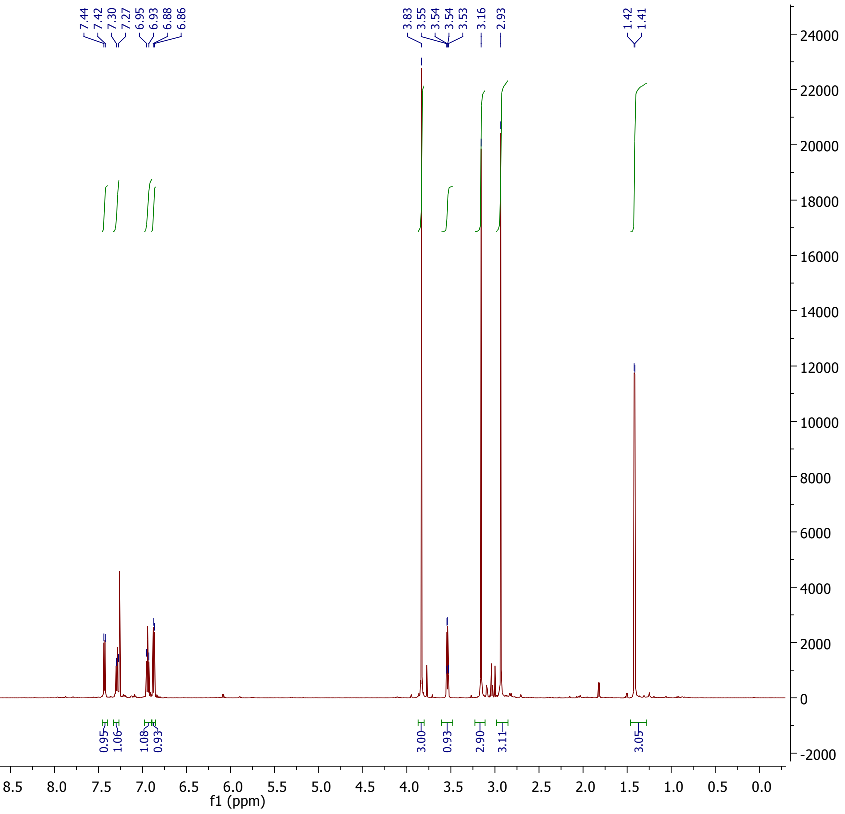

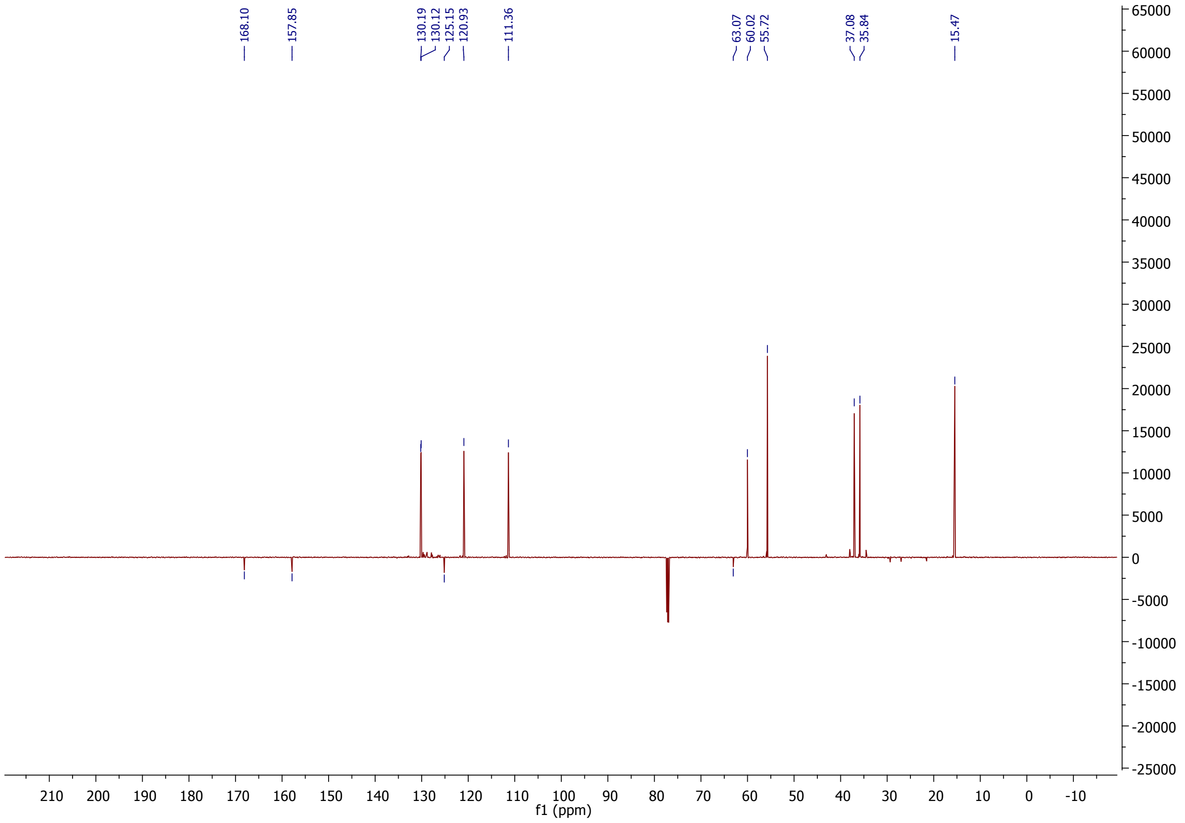

Compound **10l**

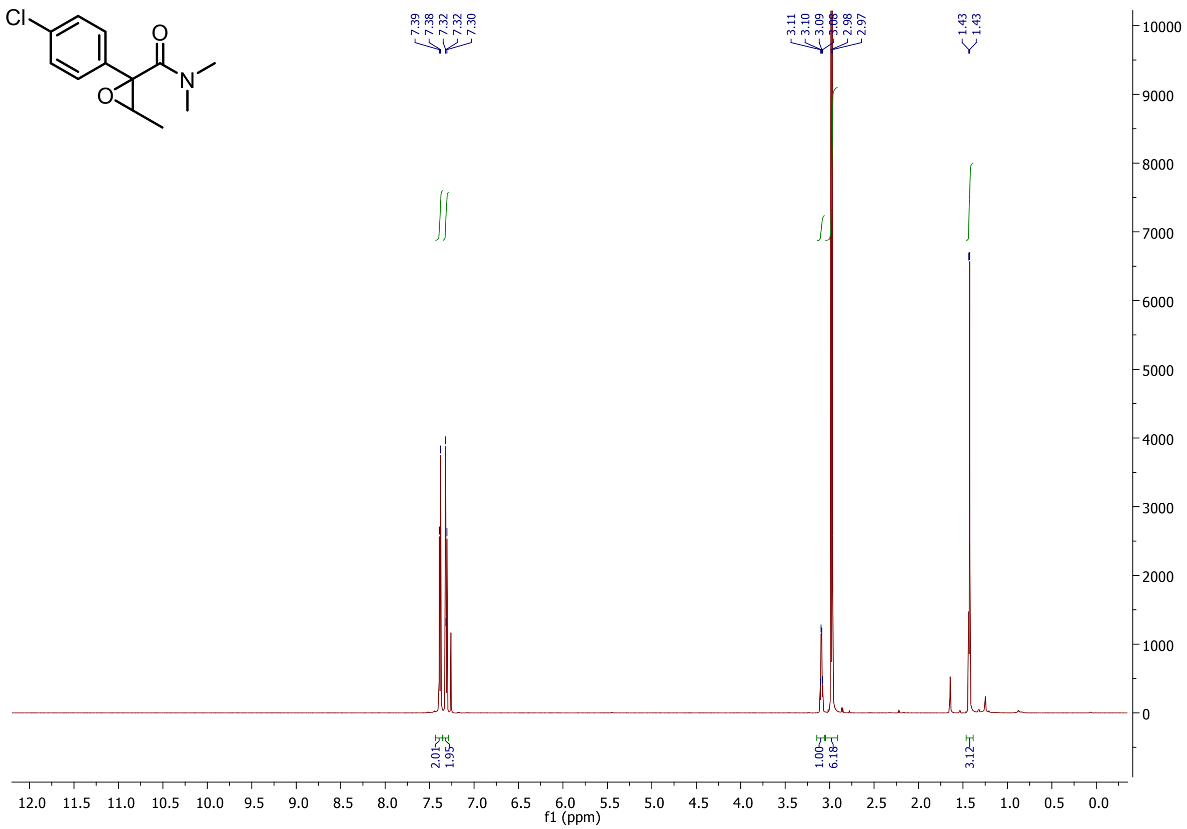
7.39
7.38
7.32
7.32
7.30
3.11
3.10
3.09
3.08
2.98
2.97
1.43
1.43
2.01
1.95
1.00
6.18
3.12
f1 (ppm)

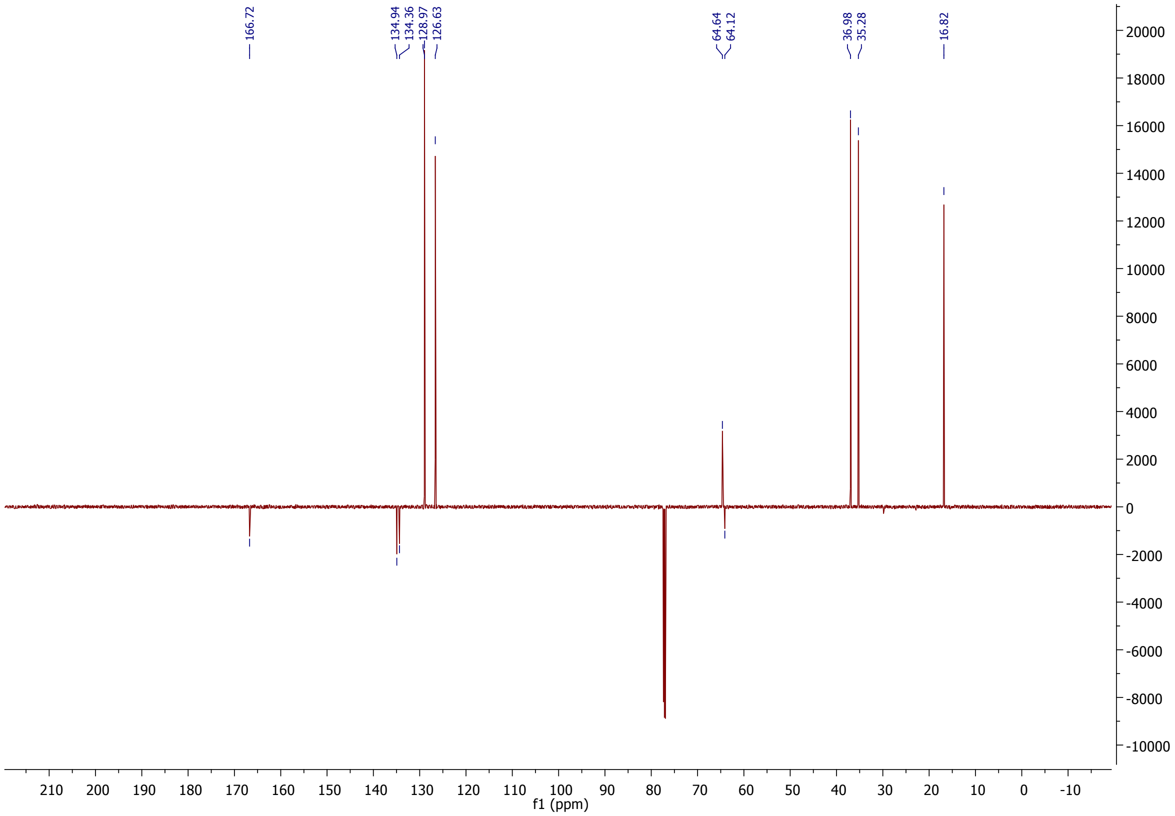

166.72
134.94
134.36
128.97
126.63
64.64
64.12
36.98
35.28
16.82
20000
18000
16000
14000
12000
10000
8000
6000
4000
2000
0
-2000
-4000
-6000
-8000
-10000
210 200 190 180 170 160 150 140 130 120 110 100 90 80 70 60 50 40 30 20 10 0 -10
f1 (ppm)

Compound **10m**

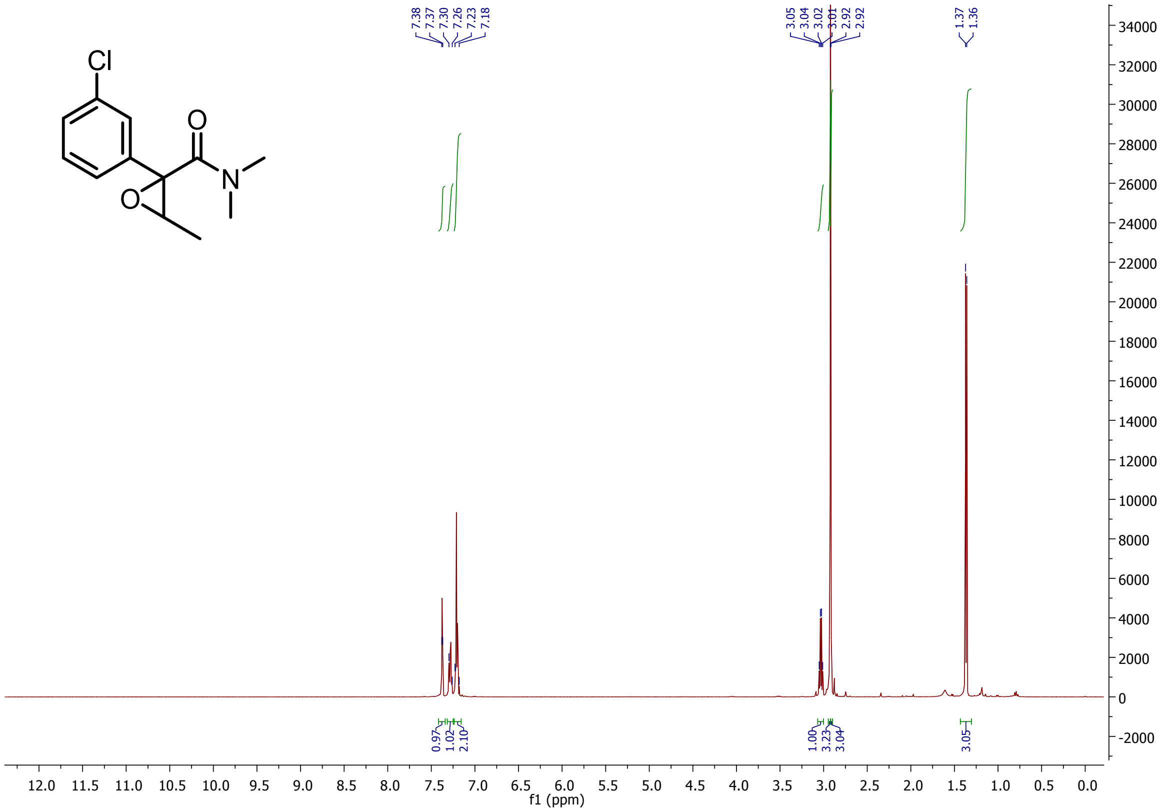

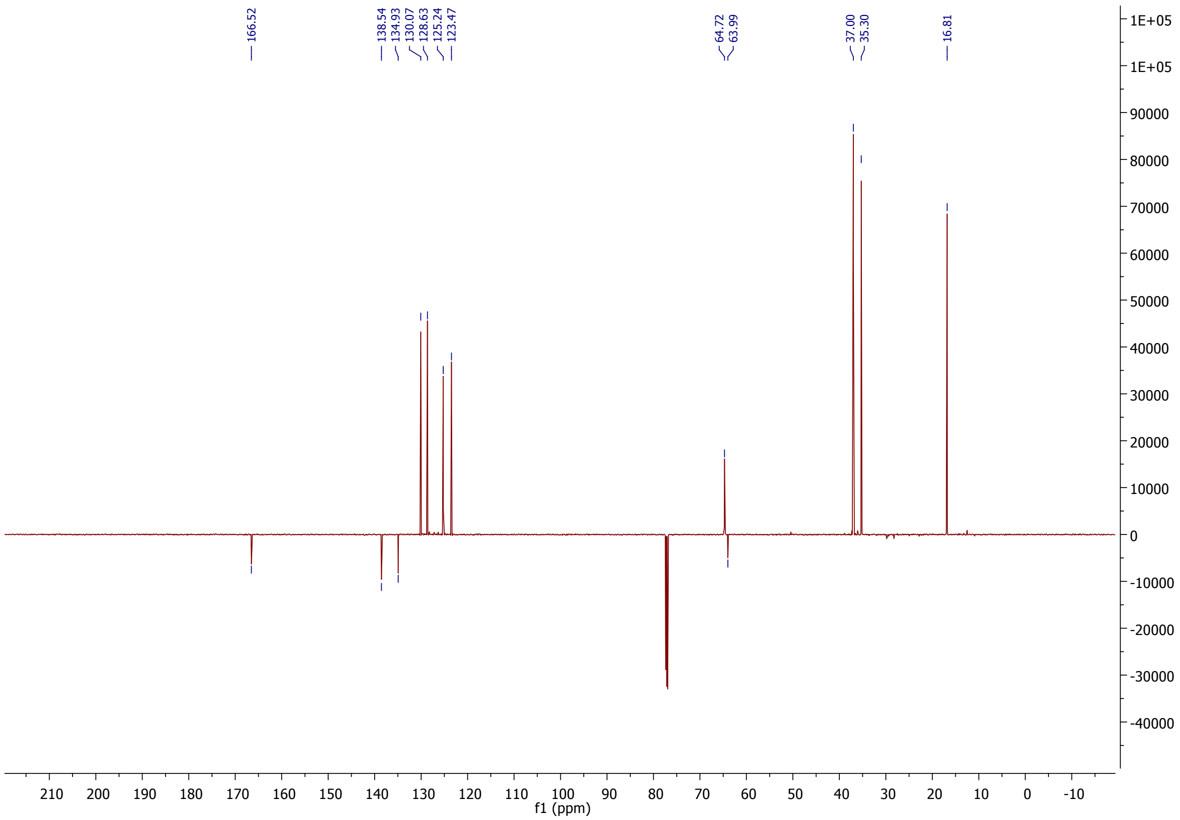

Compound **10n**

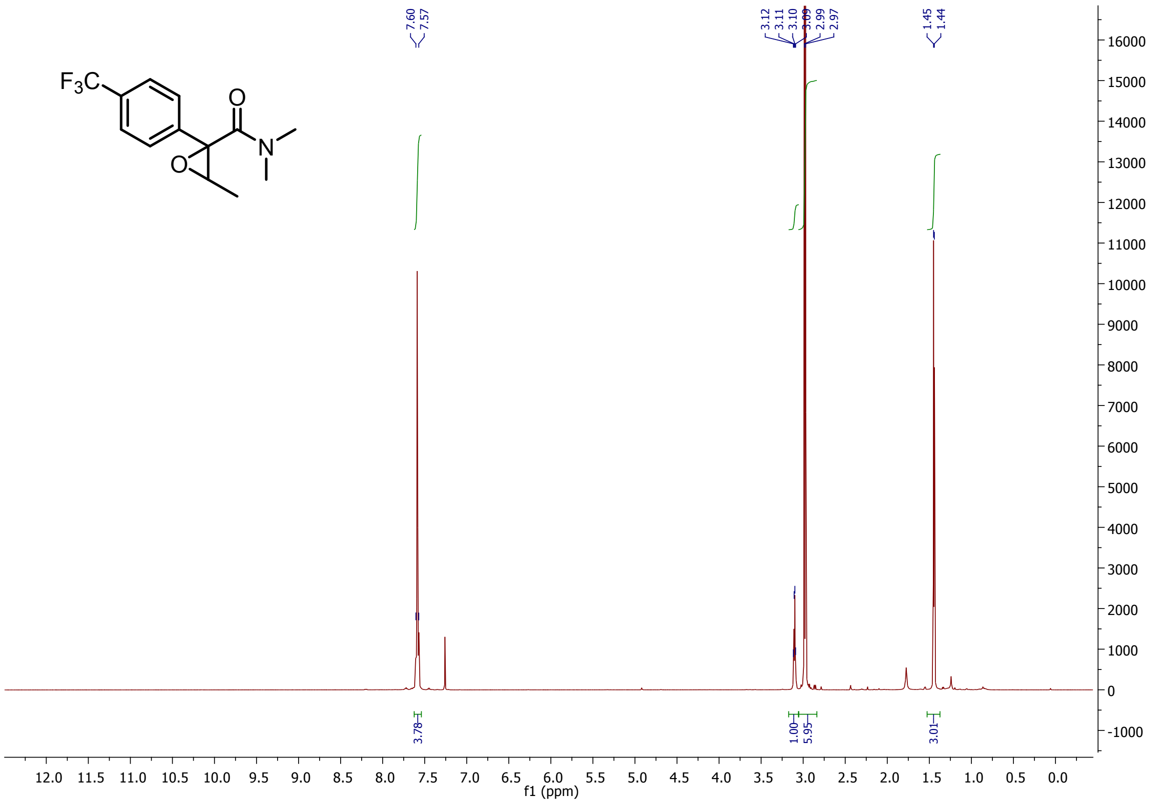
F3C
O
N
O
7.60
7.57
3.12
3.11
3.10
3.09
2.99
2.97
1.45
1.44
3.78
1.00
5.95
3.01
f1 (ppm)

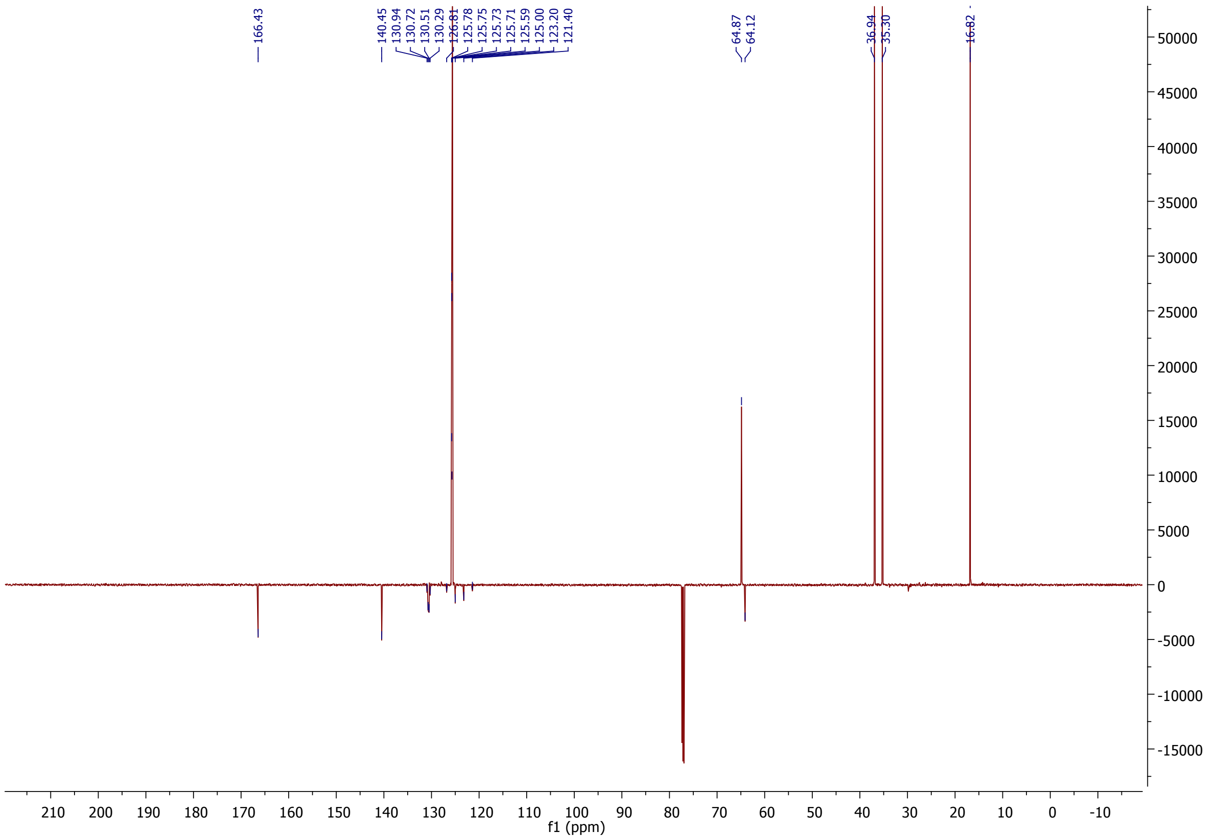

Compound **10o**

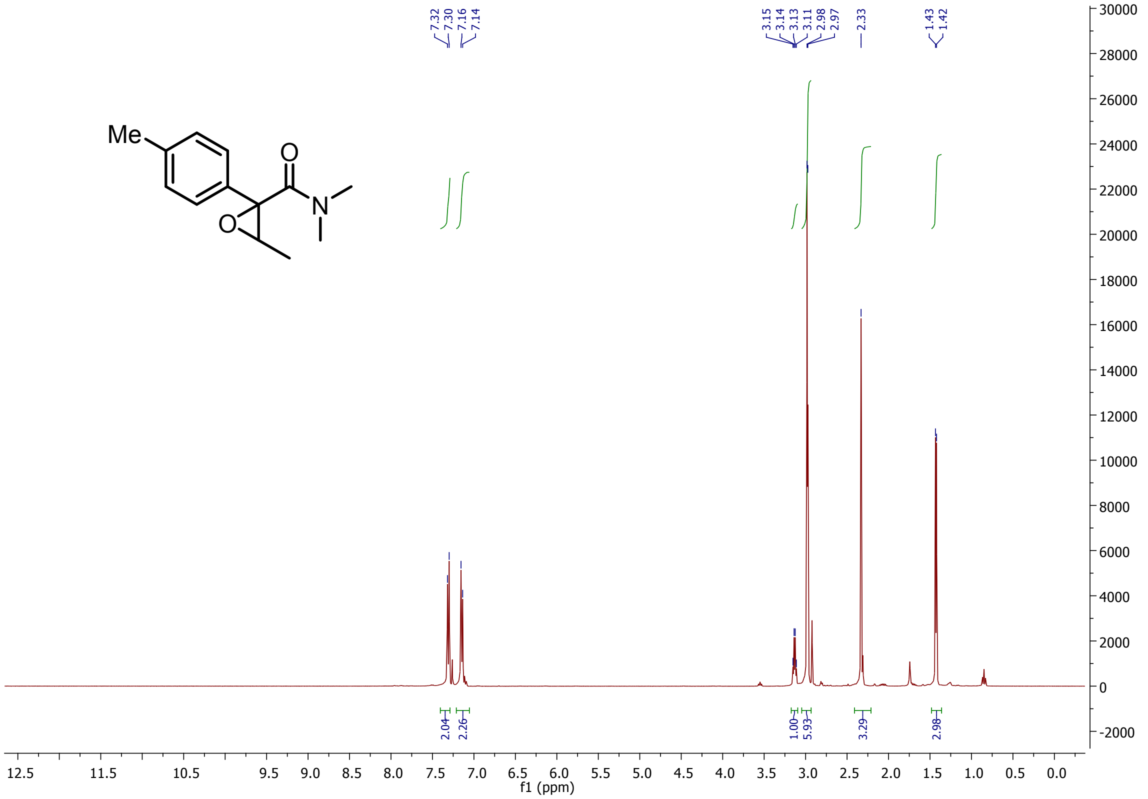
Me
O
N
O
7.32
7.30
7.16
7.14
3.15
3.14
3.13
3.11
2.98
2.97
2.33
1.43
1.42
2.04
2.26
1.00
5.93
3.29
2.98
f1 (ppm)

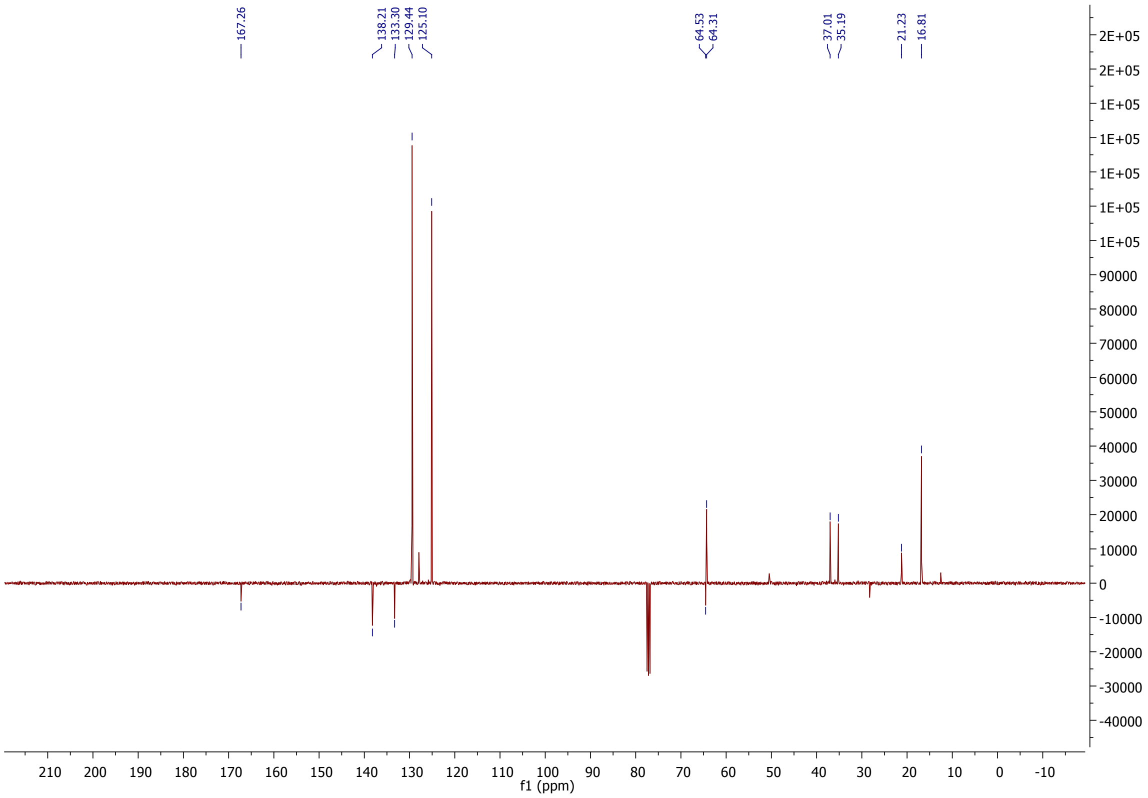
167.26
138.21
133.30
129.44
125.10
64.53
64.31
37.01
35.19
21.23
16.81
f1 (ppm)

Compound **10p**

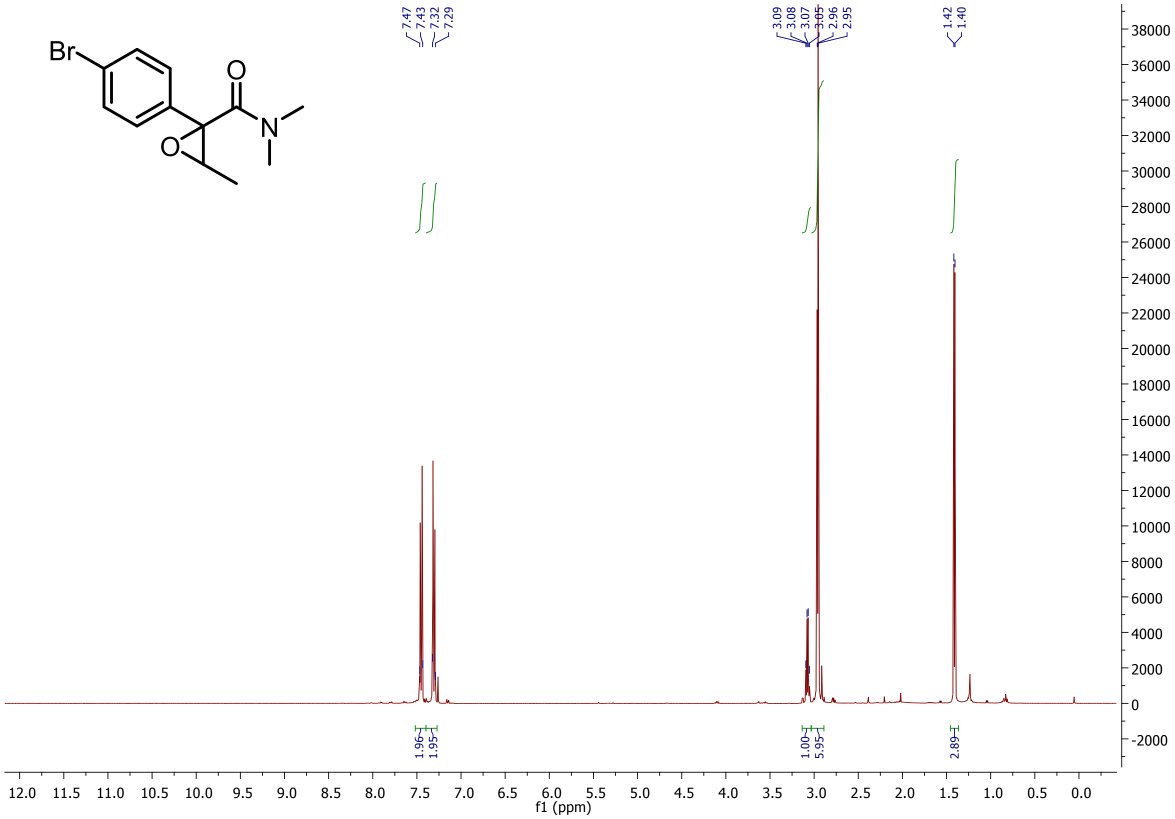

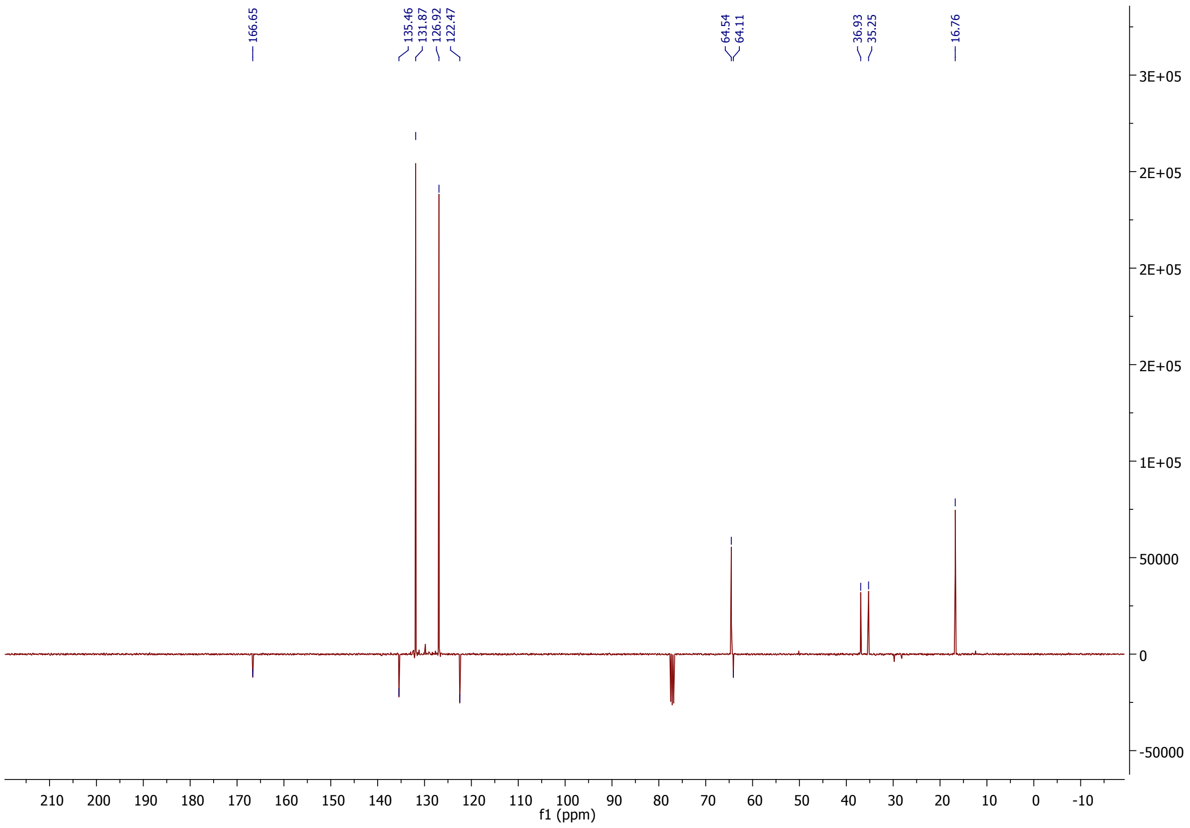

Compound **11g**

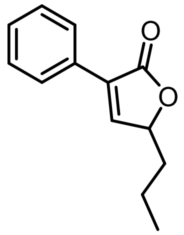

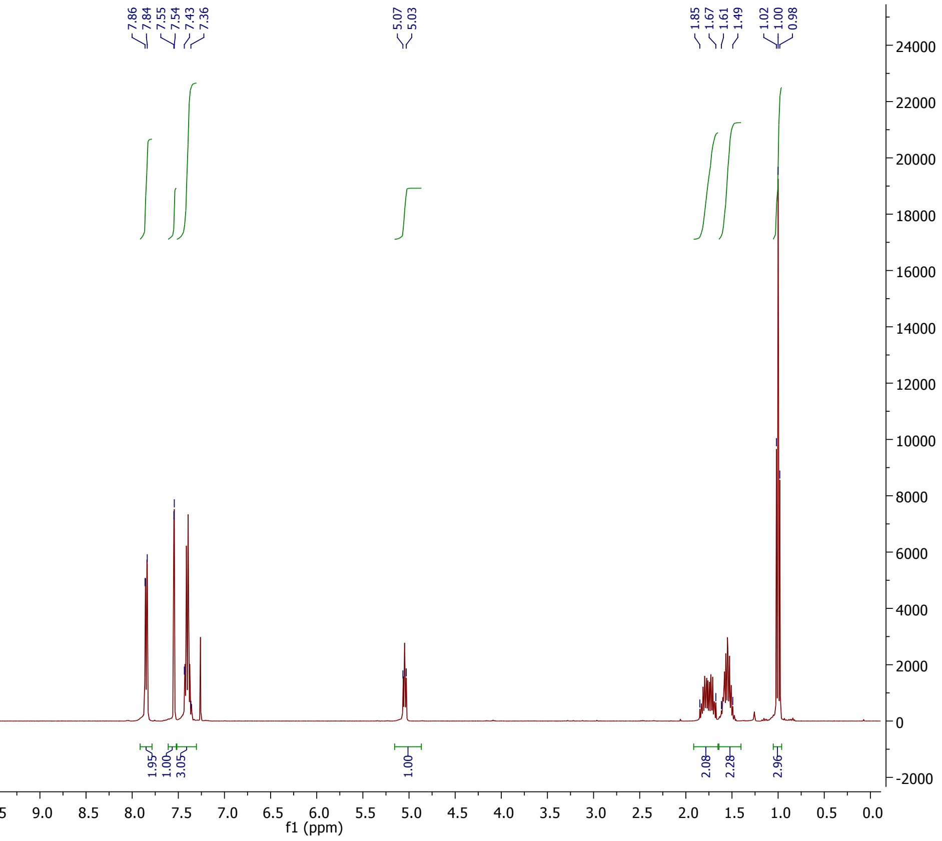
7.86
7.84
7.55
7.54
7.43
7.36
5.07
5.03
1.85
1.67
1.61
1.49
1.02
1.00
0.98
1.95
1.00
3.05
1.00
2.08
2.28
2.96
f1 (ppm)

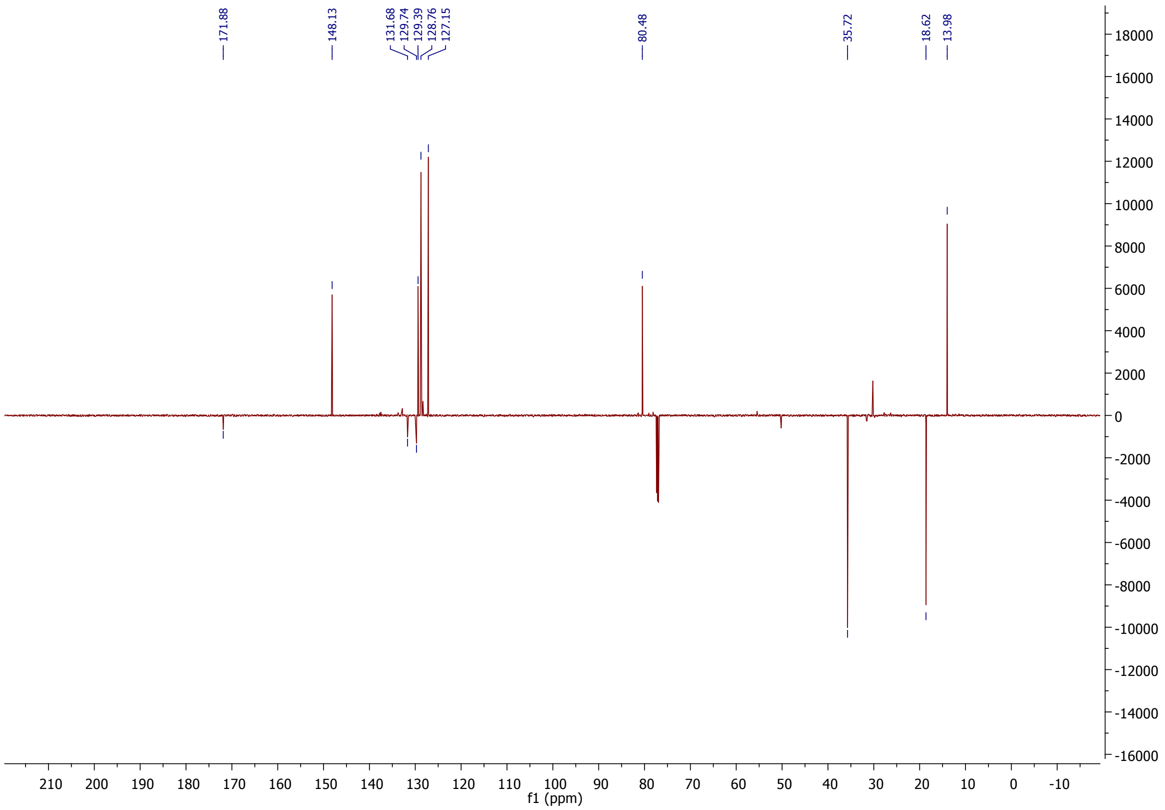

Compound **11q**

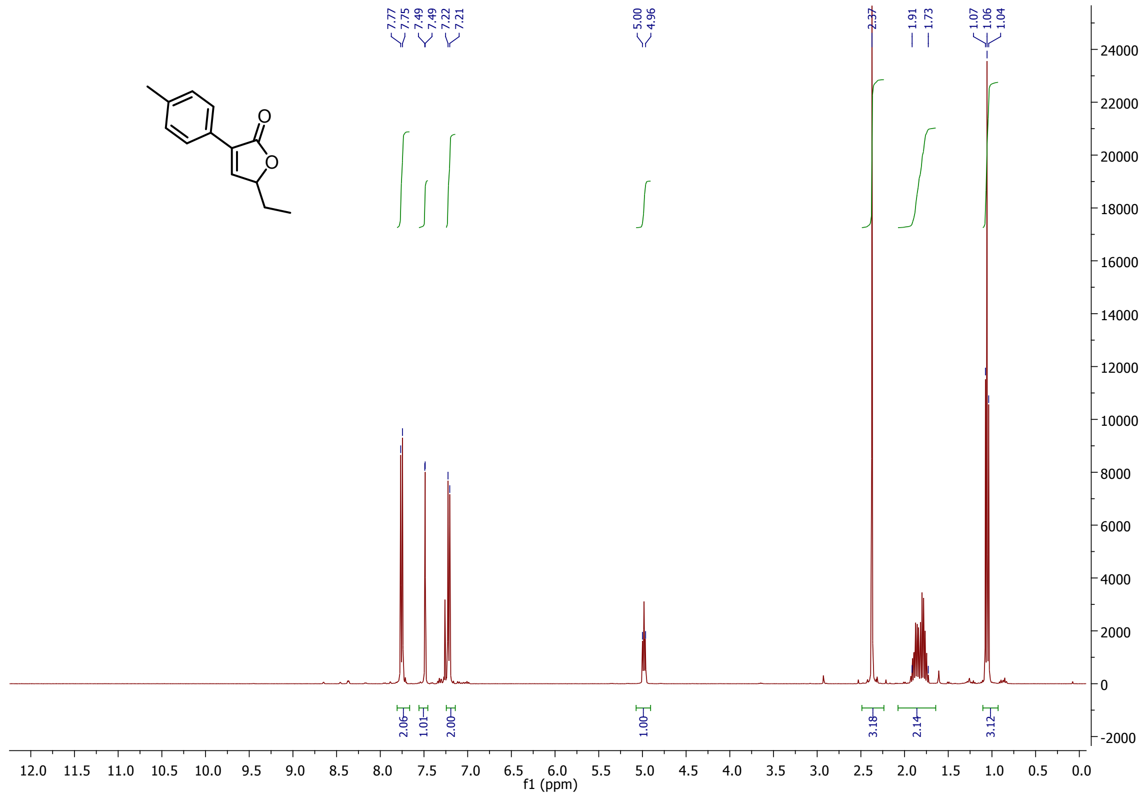

Compound **11a**

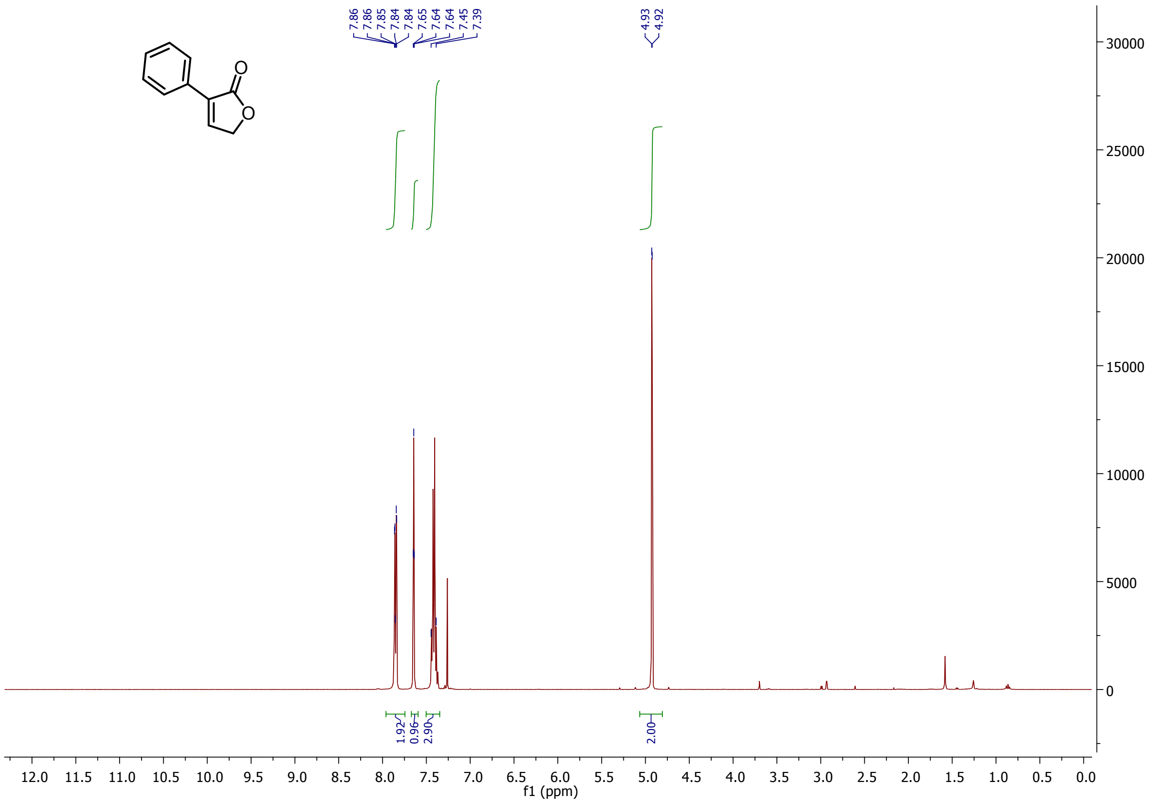
7.86
7.86
7.85
7.84
7.84
7.65
7.64
7.64
7.45
7.39
4.93
4.92
1.92
0.96
2.90
2.00
f1 (ppm)

Compound **22**

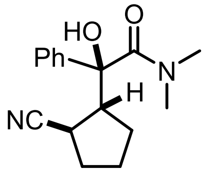
HO
O
Ph
N
H
NC

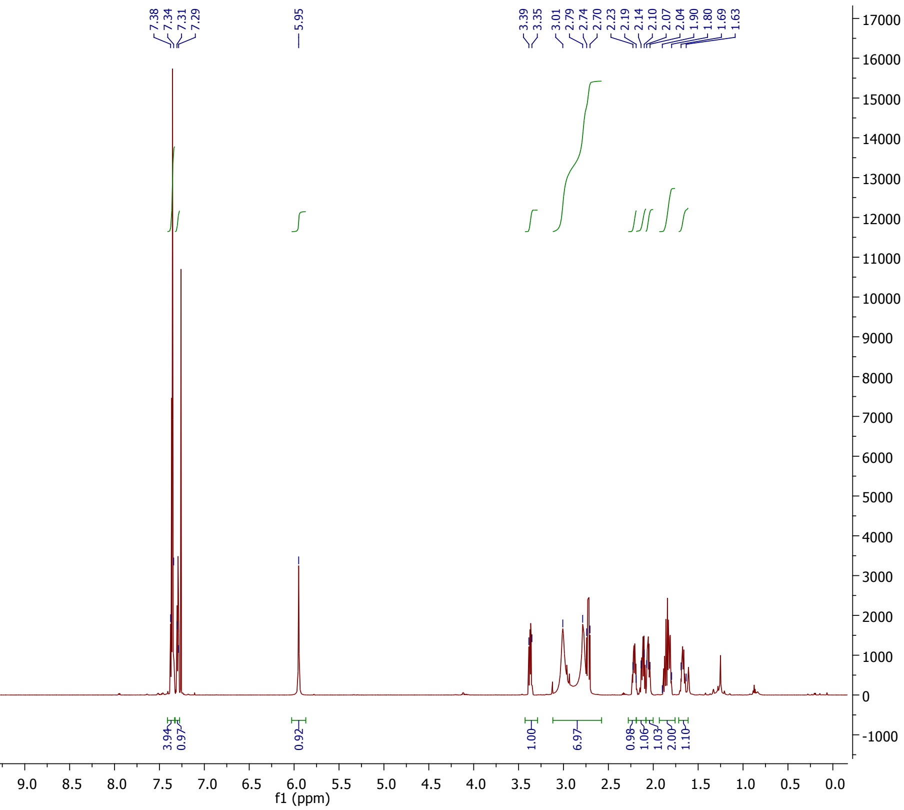
7.38
7.34
7.31
7.29
5.95
3.39
3.35
3.01
2.79
2.74
2.70
2.23
2.19
2.14
2.10
2.07
2.04
1.90
1.80
1.69
1.63
3.94
0.97
0.92
1.00
6.97
0.98
1.06
1.03
2.00
1.10
f1 (ppm)

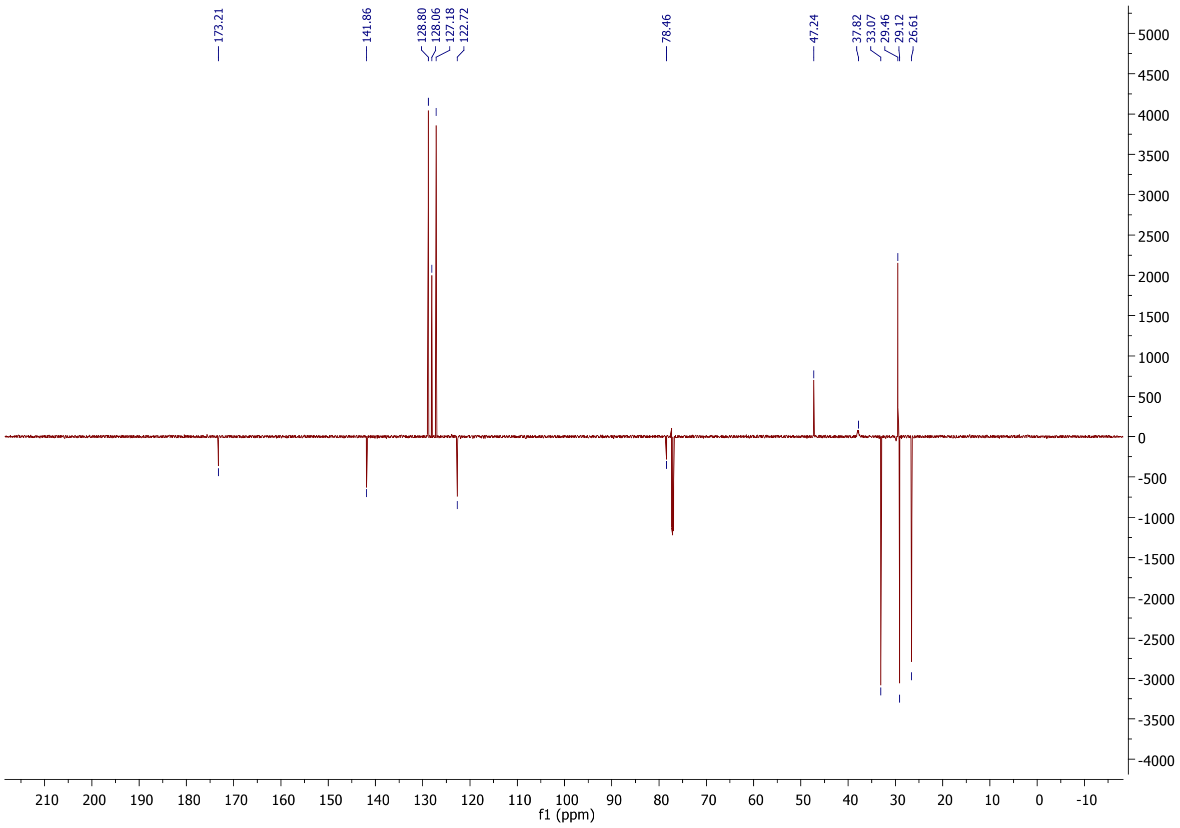
173.21
141.86
128.80
128.06
127.18
122.72
78.46
47.24
37.82
33.07
29.46
29.12
26.61
f1 (ppm)

Compound **23**

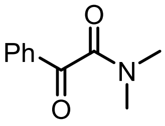
Ph
O
N
O

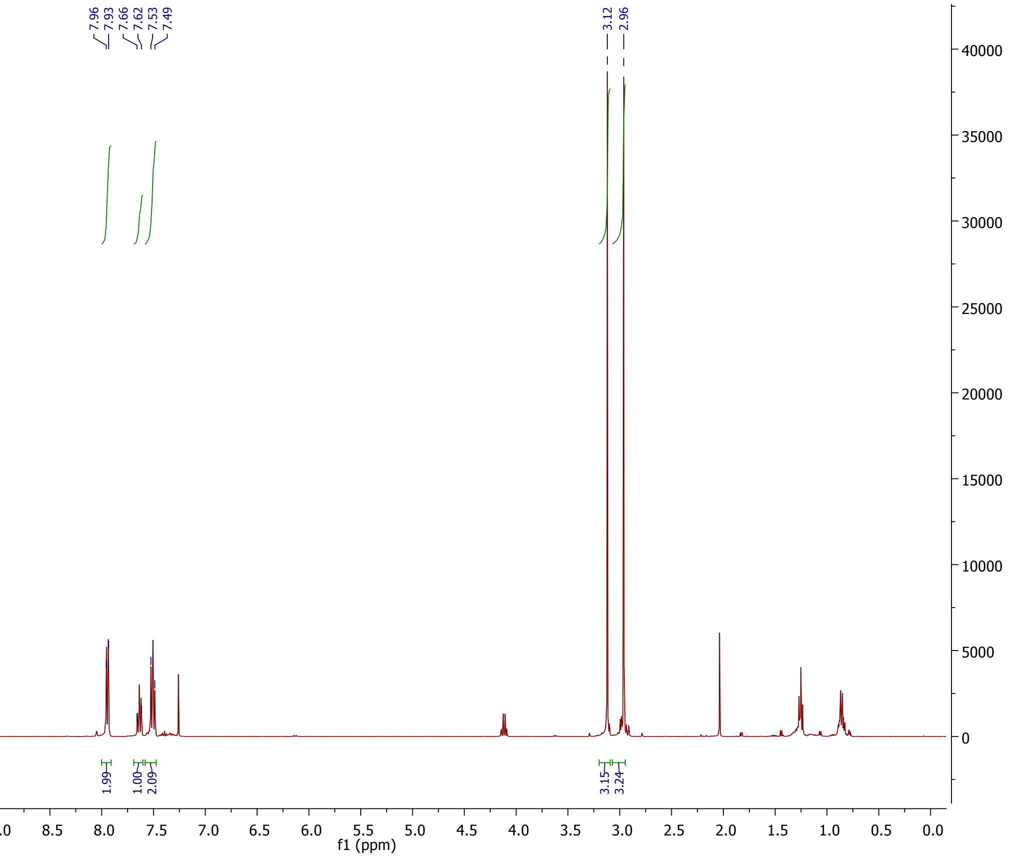
7.96
7.93
7.66
7.62
7.53
7.49
3.12
2.96
1.99
1.00
2.09
3.15
3.24
40000
35000
30000
25000
20000
15000
10000
5000
0
11.0 10.5 10.0 9.5 9.0 8.5 8.0 7.5 7.0 6.5 6.0 5.5 5.0 4.5 4.0 3.5 3.0 2.5 2.0 1.5 1.0 0.5 0.0
f1 (ppm)
